# Computational evidence for hundreds of non-conserved plant microRNAs

**DOI:** 10.1186/1471-2164-6-119

**Published:** 2005-09-13

**Authors:** Morten Lindow, Anders Krogh

**Affiliations:** 1 Bioinformatics Centre, Institute of Molecular Biology, University of Copenhagen, Denmark

## Abstract

**Background:**

MicroRNAs (miRNA) are small (20–25 nt) non-coding RNA molecules that regulate gene expression through interaction with mRNA in plants and metazoans. A few hundred miRNAs are known or predicted, and most of those are evolutionarily conserved. In general plant miRNA are different from their animal counterpart: most plant miRNAs show near perfect complementarity to their targets. Exploiting this complementarity we have developed a method for identification plant miRNAs that does not rely on phylogenetic conservation.

**Results:**

Using the presumed targets for the known miRNA as positive controls, we list and filter all segments of the genome of length ~20 that are complementary to a target mRNA-transcript. From the positive control we recover 41 (of 92 possible) of the already known miRNA-genes (representing 14 of 16 families) with only four false positives.

Applying the procedure to find possible new miRNAs targeting any annotated mRNA, we predict of 592 new miRNA genes, many of which are not conserved in other plant genomes. A subset of our predicted miRNAs is additionally supported by having more than one target that are not homologues.

**Conclusion:**

These results indicate that it is possible to reliably predict miRNA-genes without using genome comparisons. Furthermore it suggests that the number of plant miRNAs have been underestimated and points to the existence of recently evolved miRNAs in *Arabidopsis*.

## Background

MicroRNAs (miRNAs), 20–25 nucleotides in length, are involved in negative post transcriptional regulation in most multi-cellular organisms (for a review see e.g. [1,2]). The generality and importance of this recently discovered regulatory mechanism is gradually becoming apparent, and here we present computational evidence for new miRNAs indicating that their numbers are more abundant than previously believed, and argue that they play a major role in evolution.

Most of the miRNAs identified so far are conserved in other species, some remarkably well[3]. Previous computational screens for miRNA have relied on this evolutionary conservation to identify a few hundred putative miRNAs in vertebrates[4], *C. elegans*[5], and plants [6-8], and many have been experimentally confirmed (reviewed in [9]). However, these screens miss all miRNAs that have diverged since the last common ancestor of the genomes under comparison. A recent study using a combined bioinformatic and high-throughput experimental approach have identified 53 miRNAs not conserved beyond primates[10]. In plants, where comparisons have been between the distantly related *A. thaliana *(thale cress) and *O. sativa *(rice) genomes that diverged some 200 million years ago[11], it is probable that there are miRNAs which have escaped detection. Of the 112 *Arabidopsis *miRNA-genes currently registered[12], only 56 are conserved in the monocot rice (see methods section), indicating the existence of a substantial number of unconserved miRNA-genes. miRNA and short interfering RNAs (siRNA) are very similar in function, but different in biogenesis. According to the current nomenclature[13] both microRNAs (miRNAs) and short interfering RNAs (siRNAs) are 20–25 nucleotides long single stranded molecules that arise from processing of double stranded RNA (dsRNA) precursors. They are distinguished by the type of dsRNA they are excised from. While siRNAs come from long exogenous or endogenous dsRNA molecules (very long hairpins or RNA duplexes), mature miRNAs come from the stem region of shorter hairpins.

The mature miRNA or siRNA forms part of the RNA induced silencing complex (RISC) that binds to mRNAs. miRNA/siRNAs that bind with almost perfect complementarity to an mRNA often results in the cleavage of its target. Currently it seems that the higher the degree of complementarity to a target mRNA, the larger chance of that target being degraded. miRNAs with imperfect complementarity to a 3' untranslated region of a mRNA have been shown to inhibit translation of the mRNA[14,15]

When the base pairing between the miRNA and the target is incomplete it is non-trivial to identify targets for a miRNA [16-19]. In plants, however, most of the known miRNAs pair almost perfectly with one or more mRNAs, making it straightforward to identify likely plant targets (miRNAs often have more than one target). Using this observation it is possible to predict miRNA candidates in *Arabidopsis *that exhibit near perfect base pairing with the targets, without relying on homology to other organisms[20]. Here this idea is extended and refined to yield a highly specific screen that finds plant miRNAs in numbers much larger than previously thought.

## Results and discussion

### Identification of non-conserved miRNAs

**Figure 1:**
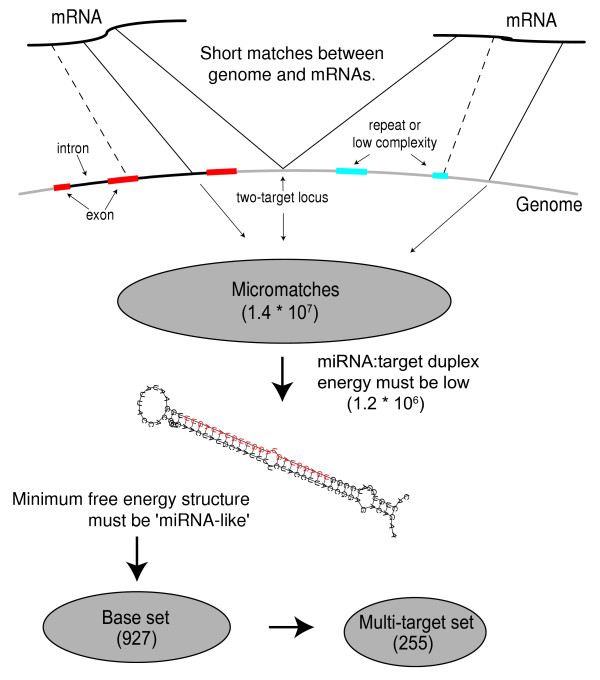
**Procedure for miRNA prediction. **The number of matches between a mRNA and a segment of the genome (micromatches) after each step is shown in parenthesis. mRNAs are compared with the genomic sequence to identify matching regions of 20–27 nucleotides with at most 2 mismatches. Matches overlapping annotated exons, repeats or low-complexity regions are discarded. Additionally, the miRNA:mRNA-duplexes must be stable and the potential miRNAs must have a structure similar to known miRNAs to be included in the base set predictions. The multi-target set is a more reliable subset of those that have more than one target. See text for more details.

The general approach is outlined in figure 1. Initially, a mRNA is compared with the genomic sequence to identify matching regions of 20–27 nucleotides with at most 2 mismatches (allowing 3 mismatches produced more than 10 000 matches per mRNA). These are called micromatches, and the genomic part is referred to as a genomic match. An average mRNA gives rise to about 1000 such micromatches, the vast majority (often all) of which we assume are spurious non-miRNA hits. However, it is possible, without comparing to other genomes, to filter the micromatches and achieve highly specific and fairly sensitive predictions of miRNA genes (Figure 1).

Six filters were used to identify a base set of genomic sequences as candidate miRNAs (with percentages of the initial micromatches that were remaining after each filter given in brackets): (1) they had high sequence complexity (26.9%); (2) they had no overlap with annotated exons on the same or the opposite strand (3.3%); (3) they had no overlap with repeat sequences defined by RepeatMasker (2.6%); (4) the putative miRNA:mRNA duplex should be relatively stable[17,21] with a calculated free energy of less than -34 kcal/mol (0.20%); (5) they had no more than identical 10 copies in the genome (0.19%), to eliminate repeated sequences not detected by standard repeat-masking; and (6) the miRNA was contained within a precursor structure that was similar to those observed in known *Arabidopsis *miRNA precursors, i.e. was predicted to be largely contained (at least 16 paired bases) within the stem of a double stranded stem-loop structure whose stem was predicted to have a free energy less than -60 kcal/mol, with at least 4 paired bases flanking the putative miRNA, and an intervening loop larger than 9 but less than 130 bases (0.0002%).

Although the base set predictions have a low number of false positives (see below), they can be even more refined to identify a subset of the predictions with extra confidence, because the probability of more than one mRNA matching a falsely predicted miRNA is minimal, unless the matching mRNA-targets are close homologs (in which case the multiple targets do not add much extra confidence). Most of the known miRNA in *Arabidopsis *are thought to have multiple targets often within the same family of homologous proteins[22]. If a known miRNA only has targets in a highly conserved protein family this filter can however be expected to falsely eliminate them.

In order to check the validity of our approach we took the mRNA targets of the known miRNAs and set out to see if using these as queries we would be able to correctly identify the known miRNA-genes. Of the 112 precursor sequences registered in RFAM (ver 5.1), we were able to map 92 perfectly to the current RefSeq assembly (TIGR ver 5.0) of the *Arabidopsis *genome; the remaining precursors were excluded from the positive control set. Likely targets for *Arabidopsis *miRNAs have previously been predicted allowing for up to 3 mismatches[23]. Repeating this procedure we find that our known miRNAs match 142 different annotated mRNA*. These are the positive control targets (refered to a 'known targets') and many have been experimentally confirmed[24,25]. Initially, the 142 mRNAs in the positive control set yielded 359,976 micromatches after removal of low complexity sequences. However, the filtering procedure reduces this dramatically to 45 different loci (41 of which are already known) representing 16 different families (14 known). Assuming that the 'unknown' loci we find are false positives the procedure has 91% specificity and 45% sensitivity on the level of loci identified. Using the refinement step requiring more than one non-homologouos target only true positives are found, but at the expense of halving the sensitivity to 22%. The validity of the estimates of specificity and sensitivity is discussed below.

### Hundreds of novel miRNAs

Applying the micromatcher procedure to all 28860 mRNAs annotated in *Arabidopsis *identifies 592 miRNA candidate loci (480 families) in the base set (Additional file 1). In the final step this is reduced to a set of 90 (70 new) when more than one non-homologouos target per miRNA is required. This is called the multi-target set and is a subset of the base set.

All miRNA gene predictions, their targets (with some basic annotation) and the predicted secondary structure of the precursor are available as supplementary data [Additional file 1], and at our website[26]

Using public databases we were able to acquire evidence for the expression of a small number of the predictions, 9 in the base set overlap with RNA molecules recently sequenced in a large scale cloning effort of *Arabidopsis *small RNA^4^, 109 have significant matches to *Arabidopsis *ESTs and 52 of the predicted precursors contain a 20-mer sequence tag from the *Arabidopsis MPSS database*[27].

### Evolutionary conservation of the predicted miRNA-genes

From an evolutionary point of view, it would seem to be a lot easier to adapt 20 bases in a miRNA for a new target than to evolve a protein for a specific regulatory task.

For mammals it has been suggested that the more targets a microRNA has the more likely it is to be conserved[28] because of the additional constraints of having to match multiple targets.

**Figure 2:**
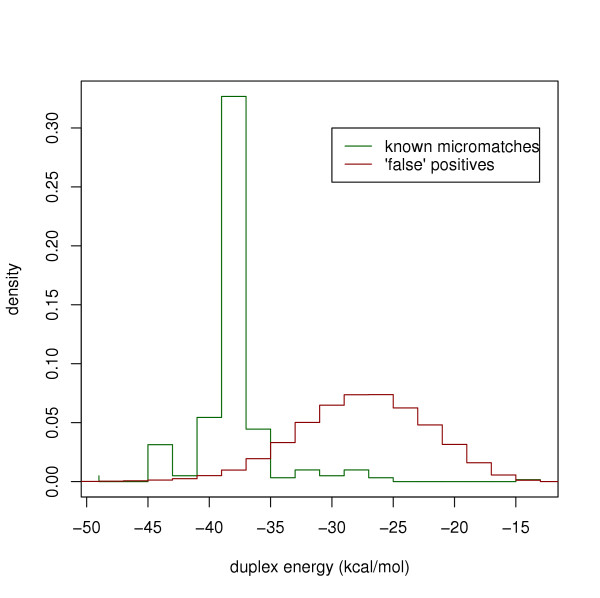
**Duplex energy is a strong discriminant between true and false micromatches. **The procedure was started with 142 mRNAs targeted by known miRNAs. Micromatches were filtered for low-complexity, overlap with exons and repeats. Then the remaining micromatches were divided in two bins: true positives (green trace) that overlap with known miRNA genes and false positives (red trace) that do not.

Indeed also for plants: comparison of our predictions in *Arabidopsis *to two other plant species reveals that the more targets a miRNA is predicted to have, the more likely it is to be conserved (Figure 2). Although no *Brassica *species is yet completely sequenced and we had to use a conjunction of all single sequence *Brassica *entries from GenBank, significantly more of the predicted miRNAs are conserved in *Brassica *than in rice, indicating that many miRNA-genes have diverged beyond recognition since the divergence of monocots and dicots approximately 200 million years ago.

Thus, we speculate that the highly conserved miRNAs are likely to be central regulators, often of many target mRNAs (imposing the evolutionary constraint to stay conserved), and are more likely to be highly expressed. Whereas more recently evolved miRNA would have fewer targets, and a more localized spatiotemporal expression, making them less likely to be detected by cloning efforts.

Since evolutionary conservation is part of many of the previous discovery procedures, it is likely that the set of known miRNAs is biased towards those that are conserved, and our data suggest that in fact, miRNAs evolve fast and are less conserved than e.g. protein-coding genes.

It has been proposed that some miRNAs originate from inverted duplication of target sequences, exemplified by the single locus miRNAs miR-161 and miR-163, which have precursors that show extended homology to the target mRNAs also outside the mature miRNA sequence[29]. However, our structural filters require that the match between miRNA and target is in the range 20–25, effectively eliminating such miRNA with extended homology.

### Comparison to other studies

Of the predicted 592 precursors in the base set, 29 overlap with the 92 predictions made by Bonnet et al.[30], and 4 of those by Wang et al.[8]. Thus, the different methods complement each other: The present method based on matching targets and miRNA is capable of finding non-conserved miRNAs, whereas the interspecies comparisons[8,31] can find miRNAs without obvious targets.

The idea to use potential targets to find miRNA-genes has recently been employed in two other studies. Xie et al. [32] started by finding frequently occurring subsequences of human 3' UTR sequences conserved in other mammals and successfully searched the genome for new miRNA genes.

Moreover Adai and coworkers[33] published results in *Arabidopsis *using potential targets to find new miRNA-genes. However, our approach differs significantly from theirs in the way the matches (that we term micromatches) are analysed and the kind of conclusions that can be drawn: Adai et al. looks for a 'cluster' of miRNA-genes that target the same sequence of a mRNA, and then aligns the candidates in such a cluster, scoring the alignment high if it shows a characteristic pattern where the miRNA and miRNA* are more conserved than the intertwining sequence. Thus, their method is limited to finding miRNAs that occur more than once in the genome, presumably as a result of duplication events. Moreover as a postfilter, Adai et al. require conservation in rice to generate their short-list used for experimental validation. Also, Adai et al. do not make any estimation of the specificity of their computational procedure and are consequently unable to speculate about the number of miRNAs.

In contrast our method is independent of whether a candidate has been duplicated in the genome or is conserved across species. Instead our aggressive filtering on the structural properties of the precursor enables us to make highly specific prediction (judging from the results using targets for known miRNAs as queries).

The multi-target miRNAs have a total of 528 different mRNA targets, which are involved in a variety of functions, but there is a notable over-representation of proteins with transcription factor activity and receptor binding activity as well as involvement in developmental processes (false discovery rate < 0.001, see Additional file 2). The predicted miRNA-genes are generally found scattered throughout the genome (Table 2). Unlike in mammals where 90 out of 232 miRNA-genes are within introns of protein coding genes [34], there is only one previously discovered *Arabidopsis *microRNA situated in an intron. This trend of plant microRNAs to be outside protein-coding genes also holds for our baseset predictions and even stronger for the multiple target predictions (Table 2).

**Table 2:** The distribution of predicted miRNA-genes in relation to genomic features. IGR, intergenic region. The ratio of the number of bases annotated as intergenic vs. intron is 3.1 in the genome as a whole.

**Position of predicted miRNA genes**		
	Base set	>1 target

Total number of loci	592	90
In introns (sense strand)	24	3
in introns (antisense)	18	2
In intergenic regions (both strands)	550	85
Within 500 bases upstream of gene	26	3
Within 500 bases downstream of gene	52	7
Ratio IGR/introns	14	18

Although estimating the sensitivity and specificity on the basis of the ability to correctly identify the small set of known miRNAs carries the danger of biasing, the presently most important concern must be not to massively overpredict new miRNA-genes. In constructing the filters we have therefore aim at high specificity at the expense of sensitivity. While false positives undoubtfully remain, the fact that the predictions share the properties of functional overrepresentation and bias of genomic location (properties not selected for in the filters) with known miRNAs provides independent indication that we indeed do not massively overpredict new miRNA-genes.

It is becoming evident that many regions between protein coding genes are transcribed (e.g. [35,36]). Indeed given the cases of miRNAs that have been suggested to regulate other miRNAs[37] or RNAs that guide methylation DNA[38], it would be interesting to extend our filtered intragenomic match approach to identify other possible miRNAs whose targets are not mRNAs.

## Conclusion

The present analysis predicts 71 new *Arabidopsis *miRNA genes with very few false positives (estimated specificity is 100%) and over five hundred with an estimate of 9% false predictions. The procedure misses some real miRNAs, such as those encoded in untranslated regions of genes, those with very many targets (classified as repeats by our method), and those not fulfilling our strict structural constraints, and we believe that the real number could be several thousands. Although, the predictions should eventually be confirmed in the lab, our data suggest that the *Arabidopsis *genome encodes substantially more miRNA genes than previously thought, and that the number of miRNAs is comparable to the number of protein transcription factors. Our results also indicate that many miRNA are specific to small groups of related species and we speculate that they could play a part in speciation. Finally we find it unlikely that these conclusions are specific to plants, and we hypothesize that they extend to most other multicellular organisms.

## Methods

### Sequences

*Arabidopsis *genome and annotation were the RefSeq sequences based on the 5.0 version released by TIGR. Known miRNAs were from the 5.1 release of the microRNA registry[39].

### The micromatcher procedure

#### Finding all micromatches

For each annotated spliced mRNA we exhaustively searched the genome for micromatches of length at least 20 with maximum 2 mismatches (no gaps allowed) using the suffixarray based program vmatch[40] (This search took 6 days on an Intel Xeon 2.2 Ghz machine running Linux).

Note about the positive control set of mRNAs: To select the positive control mRNA-targets we allow for 3 mismatches over the whole length of the mature miRNA; this potentially includes in the positive control set mRNAs that will be unable to recover the matching miRNA allowing only 2 mismatches over a length of 20 bases (the criterion used later). This discrepancy can lead to a too pessimistic estimation of the performance of procedure.

#### Lowcomplexity filter

Genomic micromatches not fulfilling a simple low complexity filter were discarded: 1) all four bases had to be present at least once, and 2) at most 11 of the three most frequent dinucleotides in the sequence were allowed.

#### Duplex stability

Using the program RNAcofold (Vienna RNA package[41]) the free energy change when a microRNA-candidate binds to a target site was calculated. Micromatches where this duplex energy is larger than -34 kcal/mol were discarded.

#### Long matches

Micromatches longer than 26 residues were discarded. To ascertain that a micromatch was not part of a longer match, the two parts of the micromatch extended by 50 bases to each side were aligned with bl2seq (two sequence NCBI blast), and those with a match longer than 26 were discarded.

#### Overlaps with known features and repeats

A micromatch was discarded if it had any bases in common with annotated exons (including matches to the reverse strand of the exon) or repeats as determined by RepeatMasker[42] run with *Arabidopsis *specific repeat libraries (RepBase Update 8.12, RM database version 20040306).

#### Copy number

Additionally to traditional repeat-masking that relies on the identification of *known *repeats, we made an additional pragmatic repeat filter: We simply determined the number of times all candidate sequences occurs in the entire genome, and removed candidates with a copy number higher than 10.

#### Filtering on properties of the possible precursor

In order to predict a possible precursor molecule, two genomic sequences around each micromatch were extracted: One starting 10 bases 5' of the micromatch and extending 240 bases 3' of the micromatch, and one with the extension lengths reversed. Each of these was treated independently in the following analysis. First the potential precursor sequence was folded with RNAfold[43] to find the minimum free energy structure These values are comparable, because all sequences are of almost equal length. Candidates with a folding free energy larger than -60 kcal/mol are discarded. This is a highly permissive filter. The mature miRNA has to be fully contained in a double stranded region of the precursor. The complementary part of the miRNA in this stem is denoted miRNA*. It is demanded that all base pairs between the miRNA and the miRNA* are pairing in the same direction opposite each other. The number of paired bases in the mature miRNA is required to be 16 or more.

In the known miRNA precursors, the stem is always longer than just the length of the mature miRNA. To find how far the stem of a candidate extends from the mature miRNA, we count how far inward towards the loop or outwards toward the ends of RNA-string the stem extends using the following algorithm: Moving out from the terminal basepair between miRNA and miRNA* a score of 1 is assigned for each base pair encountered and a score of -1 for each unpaired base. The extension is stopped when the current score is less than 5 lower than the maximum score so far. The last base pair is considered the terminus of the stem. Candidates with extensions less than 4 bases on either side of the mature miRNA were discarded. It was also required that the shortest number of bases between the miRNA and miRNA* were larger than 9 and less than 130.

Taken together these structural criteria constitute a highly selective, but somewhat conservative filter.

### Matches to ESTs and ASRP

BLASTN was used to search all *Arabidopsis *ESTs downloaded from GenBank on September 27, 2004. Hits longer than 70 nucleotides with more than 95% identity between a predicted precursor and an EST were considered positive. Sequences cloned and sequenced as part of the *Arabidopsis *Small RNA Project (ASRP)[44], were downloaded from [45]. All matches at least 15 long with at most one mismatch with our predicted mature miRNA-sequences were found using vmatch[46].

### Conservation in other genomes

To determine how many of our predictions were conserved in other plant genomes, we blasted the predicted *Arabidopsis *precursors against the rice-genome and *brassica *sequence downloaded from [47]. A miRNA prediction was taken to be conserved if it had a significant (e-value < 0.01) blast hit containing the mature miRNA with no more than 2 mismatches and the homolog had flanking sequence capable of folding back on the mature miRNA with at least 15 base pairs between the miRNA and miRNA*.

### The number of non-homologous targets for a putative miRNA

For all candidate microRNAs in the baseset matching more than one mRNA, we found the number of different non-homologous targets by performing single linkage clustering on the aminoacid sequences of the corresponding mRNAs using the program 'blastclust' from NCBI. Two proteins were considered homologous if they had more than 70% identity across at least 50% of the length.

### Clustering of micromatches into genomic loci

Micromatches with genomic start position within 4 nucleotides were logically grouped into the same locus.

### Clustering of similar miRNA sequences into families

We used the program vmatch[48] to align and perform single linkage clustering of the predicted mature miRNA sequences. Candidate pairs aligning over at least 17 bases, allowing an edit distance of 1 were grouped in the same family.

### Functional analysis of targets

We obtained gene ontology annotation (GOSLIM) from [49]. From each GOSLIM category we constructed a 2 × 2 contingency table counting the number of targets vs non-targets with or without the GOSLIM annotation. We used R[50] to calculate p-values with Fisher's Exact Test and employed the package 'qvalue'[51] to correct for multiple testing setting a false discovery rate level at 0.001. The results are included as [Additional file 2], along with the R-code used.

## Authors' contributions

ML and AK designed the study. ML wrote the programs. ML and AK drafted the manuscript. Both authors read and approved the final manuscript.

**Figure 3:**
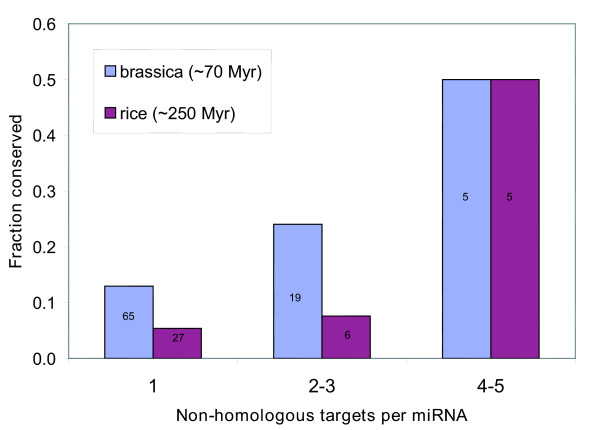
**Multi target predictions tend to be better conserved**. The precursor sequences of the predictions were used as queries for a blast search against rice (downloaded from tigr.org, March 2004) or *brassica *(downloaded from arabidopsis.org, August 2004), respectively. Columns show the proportion of miRNA predictions in *Arabidopsis *that were found to be conserved. Numbers refer to the actual number of conserved miRNA predictions.

**Table 1:** Summary of the results, starting with 136 mRNA targets to known miRNAs or all mRNAs, respectively. Numbers in parenthesis indicate the number of already known (RFAM) miRNA genes or families.

	**micromatches**	**miRNA genes found**	**distinct families**	**distinct targets**
***Query: known targets***				
Baseset	176	45(41)	16(14)	51
>1 non-homologous target	63	20(20)	12(12)	34
***Query: all mRNAs***				
Baseset	927	592	480	656
>1 target-homologous target	255	90	73	205

## Supplementary Material

Additional File 1 Predicted miRNA genes. List of predicted miRNA-genes, their predicted targets, genomic location and graphics showing predicted structure of the precursors. Click here for file

Additional File 2 Functional analysis of the predicted miRNA targets. Analysis of overrepresented Gene Ontology terms among the mRNAs predicted to be targeted by miRNAs. Click here for file
