# Supplementary material for: Computational evidence for hundreds of non-conserved plant microRNAs
Source: BMC Genomics. 2005 Sep 13;6:119. doi: 10.1186/1471-2164-6-119 (PMC1249568; doi:10.1186/1471-2164-6-119)
Supplement: Additional File 1 — Predicted miRNA genes. List of predicted miRNA-genes, their predicted targets, genomic location and graphics showing predicted structure of the precursors. [file 1471-2164-6-119-S1.html]

xml version="1.0" encoding="iso-8859-1"?

miRNA predictions in Arabidopsis

## Supplementary data for the paper: 'Computational evidence for hundreds of non-conserved plant miRNAs' by Morten Lindow & Anders Krogh

- Each predicted miRNA-gene appears between two horizontal lines and is uniquely identified by a locus\_id. Mature miRNAs with similar sequences (see methods) are assigned the same family\_id
- A note is added: (1) When a predicted miRNA-gene is identical to an already known one, and (2) when the predicted miRNA-gene have homolog in the brassica-family or in rice.
- Brief information about the targets are shown, please follow the link to Genbank for more information.
- A description of the genomic location of the prediction is given.
- An alignment between the mature miRNA (with 3 extra nucleotides on the flanks) and the reverse complement of the target sites (also with 3 extra flanking nucleotides).
- Link to picture of the predicted precursor structure, with the mature sequence shown in red (the actual picture files reside on a remote server, so internet accesss is required to view these).
- Sequence and structure(in dot-bracket format) of the predicted precursor stem-loop with the mature miRNA sequences shown in capital letters.

Please go to http://www.binf.ku.dk/users/morten/mimatcher/arabidopsis/ for future updates of this file.

---

## locus\_id: 209396

family\_id: 145

### **Targets:**

At4g21910(NM\_202859.1
): MATE efflux family protein  
At4g21910(NM\_202858.1
): MATE efflux family protein  
At4g21910(NM\_118312.2
): MATE efflux family protein

Location in genome: in an IGR, 3163 upstream of At3g44780, 2435 downstream of At3g44785

### Alignment between mature miRNA and predicted targets

```
        Extended mature miRNA:       ttatgtctatcactccatcaaacaca
        Target(rc):NM_202858.1       tatTGTCTATCACTCCACCAAACttg
        Target(rc):NM_118312.2       tatTGTCTATCACTCCACCAAACttg
        Target(rc):NM_202859.1       tatTGTCTATCACTCCACCAAACttg
```

### Precursor sequence and structure. Mature sequence in capital letters

```
ttggagtgtgtttgatggggcgacatgcatgttatcaaacacacttaTGTCTATCACTCCATCAAACacactccag   
 .(((((((((((((((((((.((((((..(((........)))..)))))).....))))))))))))))))))).
```

---

## locus\_id: 43755

family\_id: 133

### **Targets:**

(NM\_124435.2
): squamosa promoter-binding protein, putative  
At5g50570(NM\_180830.1
): squamosa promoter-binding protein, putative  
At5g50670(NM\_124445.1
): squamosa promoter-binding protein, putative  
(NM\_180791.1
): squamosa promoter-binding protein-like 2 (SPL2)  
At5g43270(NM\_123693.2
): squamosa promoter-binding protein-like 2 (SPL2)  
At1g69170(NM\_105584.4
): squamosa promoter-binding protein-like 6 (SPL6)  
At1g69170(NM\_202380.1
): squamosa promoter-binding protein-like 6 (SPL6)

Location in genome: in an IGR, 6040 upstream of At1g66770, 4717 downstream of At1g66790

2 homologs in brassica

1 homologs in rice

### Alignment between mature miRNA and predicted targets

```
        Extended mature miRNA:       tgttgacagaagatagagagcacaga
        Target(rc):NM_124435.2       ggaTGACAGAAGAGAGAGAGCACAatc
        Target(rc):NM_123693.2      aatTTGACAGAAGAGAGAGAGCACcat
        Target(rc):NM_180791.1      aatTTGACAGAAGAGAGAGAGCACcat
        Target(rc):NM_105584.4       agcTGACAGAAGAGAGAGAGCACgag
        Target(rc):NM_124445.1       ggaTGACAGAAGAGAGAGAGCACAatc
        Target(rc):NM_180830.1       ggaTGACAGAAGAGAGAGAGCACAatc
        Target(rc):NM_202380.1       agcTGACAGAAGAGAGAGAGCACgag
```

### Precursor sequence and structure. Mature sequence in capital letters

```
gtgtTGACAGAAGATAGAGAGCACagatgatgagatacaattcggagcatgttctttgcatcttactcctttgtgctctctagccttctgtcatcac   
 (((.(((((((((.(((((((((((((.((((((((.(((...((((....))))))).)))))).)).)))))))))))))..))))))))).)))
```

---

## locus\_id: 97160

family\_id: 133

### **Targets:**

(NM\_124435.2
): squamosa promoter-binding protein, putative  
At5g50670(NM\_124445.1
): squamosa promoter-binding protein, putative  
At5g50570(NM\_180830.1
): squamosa promoter-binding protein, putative  
At1g69170(NM\_105584.4
): squamosa promoter-binding protein-like 6 (SPL6)  
At1g27370(NM\_202192.1
): squamosa promoter-binding protein-like 10 (SPL10)  
At1g27360(NM\_102498.2
): squamosa promoter-binding protein-like 11 (SPL11)  
At1g27360(NM\_202191.1
): squamosa promoter-binding protein-like 11 (SPL11)  
At1g27370(NM\_102499.3
): squamosa promoter-binding protein-like 10 (SPL10)  
At5g43270(NM\_123693.2
): squamosa promoter-binding protein-like 2 (SPL2)  
(NM\_180791.1
): squamosa promoter-binding protein-like 2 (SPL2)  
At1g69170(NM\_202380.1
): squamosa promoter-binding protein-like 6 (SPL6)

Location in genome: in an IGR, 3689 upstream of At1g66800, 9620 downstream of At1g66780

2 homologs in brassica

1 homologs in rice

### Alignment between mature miRNA and predicted targets

```
        Extended mature miRNA:       tgttgacagaagatagagagcacaga
        Target(rc):NM_102498.2     gagGTTGACAGAAGAGAGAGAGCACggt
        Target(rc):NM_123693.2      aatTTGACAGAAGAGAGAGAGCACcat
        Target(rc):NM_202192.1     gtaGTTGACAGAAGAGAGAGAGCACtgt
        Target(rc):NM_202191.1     gagGTTGACAGAAGAGAGAGAGCACggt
        Target(rc):NM_202380.1       agcTGACAGAAGAGAGAGAGCACgag
        Target(rc):NM_102499.3     gtaGTTGACAGAAGAGAGAGAGCACtgt
        Target(rc):NM_124435.2       ggaTGACAGAAGAGAGAGAGCACAatc
        Target(rc):NM_180791.1      aatTTGACAGAAGAGAGAGAGCACcat
        Target(rc):NM_124445.1       ggaTGACAGAAGAGAGAGAGCACAatc
        Target(rc):NM_180830.1       ggaTGACAGAAGAGAGAGAGCACAatc
        Target(rc):NM_105584.4       agcTGACAGAAGAGAGAGAGCACgag
```

### Precursor sequence and structure. Mature sequence in capital letters

```
tgatagtgtTGACAGAAGATAGAGAGCACagatgataagatacaattcctcgcagcttctttgcatcttactcctttgtgctctctagccttctgtcatcacccgtta   
 .....(((.(((((((((.(((((((((((((.((((((((.(((...............))).)))))).)).)))))))))))))..))))))))).)))......
```

---

## locus\_id: 187603

family\_id: 128

### **Targets:**

At2g22740(NM\_201785.1
): SET domain-containing protein (SUVH6)  
At2g22740(NM\_179699.2
): SET domain-containing protein (SUVH6)

Location in genome: in an IGR, 4508 upstream of At3g16210, 6119 downstream of At3g16250

### Alignment between mature miRNA and predicted targets

```
        Extended mature miRNA:       acctctttctgcaaacgccttggattt
        Target(rc):NM_201785.1       gaaTCTTTCTGCAAACGCCATGGAaaa
        Target(rc):NM_179699.2       gaaTCTTTCTGCAAACGCCATGGAaaa
```

### Precursor sequence and structure. Mature sequence in capital letters

```
ttttataaccTCTTTCTGCAAACGCCTTGGAttttcttttggcttcatgttgttcattagaataatagccttttcatcttatttaaagaacatccaaggtgtttgtagaaaaaggtttcaaaa   
 .(((..(((((.((((((((((((((((((((.((((((.((((....(((((((....)))))))))))..............)))))).)))))))))))))))))))).)))))..))).
```

---

## locus\_id: 150313

family\_id: 124

### **Targets:**

At5g58140(NM\_180880.1
): protein kinase family protein / non phototropic hypocotyl 1-like protein (NPL1)  
(NM\_180879.1
): protein kinase family protein / non phototropic hypocotyl 1-like protein (NPL1)  
(NM\_180881.1
): protein kinase family protein / non phototropic hypocotyl 1-like protein (NPL1)

Location in genome: in an IGR, 12446 upstream of At2g09910, 88695 downstream of At2g09388

### Alignment between mature miRNA and predicted targets

```
        Extended mature miRNA:       cactcgggttcgatccccggcaacggc
        Target(rc):NM_180879.1       ctgTCTGGTTCGATCCCCGACAACact
        Target(rc):NM_180880.1       ctgTCTGGTTCGATCCCCGACAACact
        Target(rc):NM_180881.1       ctgTCTGGTTCGATCCCCGACAACact
```

### Precursor sequence and structure. Mature sequence in capital letters

```
ccgttgccggggatcgacccgggtcacccgcgtgacaggcgggaatacttaccacttagtacaacgacccaatatagtggtaagtattctcgcatgtcacTCGGGTTCGATCCCCGGCAACgg   
 (((((((((((((((((........(((((.((((((.((((((((((((((((((...................)))))))))))))))))).)))))).))))))))))))))))))))))
```

---

## locus\_id: 398850

family\_id: 120

### **Targets:**

At1g33140(NM\_103048.3
): 60S ribosomal protein L9 (RPL90A/C)  
At1g33120(NM\_103046.2
): 60S ribosomal protein L9 (RPL90B)

Location in genome: in an IGR, 8206 upstream of At5g33320, 9386 downstream of At5g33303

### Alignment between mature miRNA and predicted targets

```
        Extended mature miRNA:       ggttcaacttcttctcgccgaggattt
        Target(rc):NM_103048.3       tccTCACCTTCTTCTCGCCAAGGAagt
        Target(rc):NM_103046.2       tccTCACCTTCTTCTCGCCAAGGAagt
```

### Precursor sequence and structure. Mature sequence in capital letters

```
tctcggggtTCAACTTCTTCTCGCCGAGGAttttcactctgaccggtcttttcgctcccaaatttggaagaagatgcgatgactggaggagggggaccaattgagcgaggtgggggagacaatcgagg   
 .(((((...((..((((.(((((((((((.((..(.((((..((((((...(((((((((....))).....)).)))).)))))).)))))..))))..))).)))))).))))..))...))))).
```

---

## locus\_id: 215958

family\_id: 82

### **Targets:**

At2g10940(NM\_126828.2
): protease inhibitor/seed storage/lipid transfer protein (LTP) family protein  
At2g10940(NM\_179618.1
): protease inhibitor/seed storage/lipid transfer protein (LTP) family protein

Location in genome: in an IGR, 5035 upstream of At3g54790, 21721 downstream of At3g54830

### Alignment between mature miRNA and predicted targets

```
        Extended mature miRNA:       agggaaaatcggaggaaggggtattg
        Target(rc):NM_179618.1       cccTGAAATCGGAGGAAGGGGTAgct
        Target(rc):NM_126828.2       cccTGAAATCGGAGGAAGGGGTAgct
```

### Precursor sequence and structure. Mature sequence in capital letters

```
gcttaaggGAAAATCGGAGGAAGGGGTAttgagtgtttcacaagtaactagttcctctgtttttcctttttgc   
 ((..(((((((((.((((((((..((((((..(((...))).))).)))..)))))))).)))))))))..))
```

---

## locus\_id: 43776

family\_id: 79

### **Targets:**

At5g50530(NM\_124440.2
): CBS domain-containing protein / octicosapeptide/Phox/Bemp1 (PB1) domain-containing protein  
At5g50640(NM\_148107.1
): CBS domain-containing protein / octicosapeptide/Phox/Bemp1 (PB1) domain-containing protein

Location in genome: in an IGR, 2751 upstream of At1g66790, 6487 downstream of At1g66810

### Alignment between mature miRNA and predicted targets

```
        Extended mature miRNA:       catctgtgctctctatcttctgtcaa
        Target(rc):NM_148107.1       ctcCTGTGCCCTCTTTCTTCTGTgta
        Target(rc):NM_124440.2       ctcCTGTGCCCTCTTTCTTCTGTgta
```

### Precursor sequence and structure. Mature sequence in capital letters

```
ggtgatgacagaaggctagagagcacaaaggagtaagatgcaaagaagctgcgaggaattgtatcttatcatCTGTGCTCTCTATCTTCTGTcaacact   
 ((((.((((((((((.(((((((((((...((.((((((((((.....((....))..))))))))))))...))))))))))))))))))))).))))
```

---

## locus\_id: 97137

family\_id: 79

### **Targets:**

At5g50640(NM\_148107.1
): CBS domain-containing protein / octicosapeptide/Phox/Bemp1 (PB1) domain-containing protein  
At5g50530(NM\_124440.2
): CBS domain-containing protein / octicosapeptide/Phox/Bemp1 (PB1) domain-containing protein

Location in genome: in an IGR, 11520 upstream of At1g66800, 1789 downstream of At1g66780

### Alignment between mature miRNA and predicted targets

```
        Extended mature miRNA:       catctgtgctctctatcttctgtcaa
        Target(rc):NM_148107.1       ctcCTGTGCCCTCTTTCTTCTGTgta
        Target(rc):NM_124440.2       ctcCTGTGCCCTCTTTCTTCTGTgta
```

### Precursor sequence and structure. Mature sequence in capital letters

```
ggtgatgacagaaggctagagagcacaaaggagtaagatgcaaagaacatgctccgaattgtatctcatcatCTGTGCTCTCTATCTTCTGTcaacact   
 ((((.((((((((((.(((((((((((...((...((((((((.((......))....))))))))..))...))))))))))))))))))))).))))
```

---

## locus\_id: 236328

family\_id: 78

### **Targets:**

At4g31410(NM\_179149.1
): expressed protein  
At4g31410(NM\_119290.3
): expressed protein

Location in genome: in an IGR, 1416 upstream of At3g22400, 6014 downstream of At3g22380

### Alignment between mature miRNA and predicted targets

```
        Extended mature miRNA:       tggctggctacaagtaggatggtcaac
        Target(rc):NM_179149.1       acaCTGGCTACAAATAGGCTGGTCtgc
        Target(rc):NM_119290.3       acaCTGGCTACAAATAGGCTGGTCtgc
```

### Precursor sequence and structure. Mature sequence in capital letters

```
tgaatagttgaccatcctacatatgtggaactaaaatagaatggcagtttaaagttgatttatgatggCTGGCTACAAGTAGGATGGTCaactattga   
 ..((((((((((((((((((...(((((..(((..(((((.((((........)))).)))))..)))....))))).))))))))))))))))))..
```

---

## locus\_id: 286129

family\_id: 72

### **Targets:**

At2g32120(NM\_179852.2
): heat shock protein 70 family protein / HSP70 family protein  
At2g32120(NM\_128771.2
): heat shock protein 70 family protein / HSP70 family protein

Location in genome: in an IGR, 28 upstream of At4g21900, 1795 downstream of At4g21910

### Alignment between mature miRNA and predicted targets

```
        Extended mature miRNA:       cttctccggttagcacttccggctata
        Target(rc):NM_179852.2       cggCTCCGGTTAGCATTTCCTGCTcat
        Target(rc):NM_128771.2       cggCTCCGGTTAGCATTTCCTGCTcat
```

### Precursor sequence and structure. Mature sequence in capital letters

```
aagtcgggctcgagagcgtgttaacggagagtagcttgccttaccggaggcgcgtgtacttaggcatgtgcatagagttaaaactacttCTCCGGTTAGCACTTCCGGCTatactt   
 ((((..(((.((.(((.(((((((((((((((((...(((((....)))))..((((((........))))))..........)))).))))).))))))))))))))))..))))
```

---

## locus\_id: 412525

family\_id: 71

### **Targets:**

At3g62700(NM\_116135.2
): glutathione-conjugate transporter, putative  
At3g55970(NM\_115455.3
): oxidoreductase, 2OG-Fe(II) oxygenase family protein  
At5g50850(NM\_124463.2
): pyruvate dehydrogenase E1 component beta subunit, mitochondrial / PDHE1-B (PDH2)

Location in genome: in an IGR, 11010 upstream of At5g50550, 51599 downstream of At5g50375

### Alignment between mature miRNA and predicted targets

```
        Extended mature miRNA:       gtctagctccatcgatggctcgtgtt
        Target(rc):NM_115455.3      tcaCTAGCTCCTTCAATGGCTCGata
        Target(rc):NM_116135.2      caaCTAGCTCCATTGATGTCTCGTGcgc
        Target(rc):NM_124463.2       tcgAAGCTCCATCGATGGCTCTTtgc
```

### Precursor sequence and structure. Mature sequence in capital letters

```
gtgtgtcTAGCTCCATCGATGGCTCGTgttactcctgtgaacaatgaattagcatgaagtccatgaaacaacactagtcatcaatggagccggatactc   
 ..(((((..(((((((.(((((((.(((((..((.((.((...(((......)))....)))).))...))))).))))))).)))))))..)))))..
```

---

## locus\_id: 412591

family\_id: 71

### **Targets:**

At3g62700(NM\_116135.2
): glutathione-conjugate transporter, putative  
At3g55970(NM\_115455.3
): oxidoreductase, 2OG-Fe(II) oxygenase family protein  
At5g50850(NM\_124463.2
): pyruvate dehydrogenase E1 component beta subunit, mitochondrial / PDHE1-B (PDH2)

Location in genome: in an IGR, 11012 upstream of At5g50650, 4922 downstream of At5g50610

### Alignment between mature miRNA and predicted targets

```
        Extended mature miRNA:       gtctagctccatcgatggctcgtgtt
        Target(rc):NM_115455.3      tcaCTAGCTCCTTCAATGGCTCGata
        Target(rc):NM_116135.2      caaCTAGCTCCATTGATGTCTCGTGcgc
        Target(rc):NM_124463.2       tcgAAGCTCCATCGATGGCTCTTtgc
```

### Precursor sequence and structure. Mature sequence in capital letters

```
gtgtgtcTAGCTCCATCGATGGCTCGTgttactcctgtgaacaatgaattagcatgaagtccatgaaacaacactagtcatcaatggagccggatactc   
 ..(((((..(((((((.(((((((.(((((..((.((.((...(((......)))....)))).))...))))).))))))).)))))))..)))))..
```

---

## locus\_id: 238261

family\_id: 70

### **Targets:**

At5g36730(NM\_123030.1
): F-box family protein  
At5g36820(NM\_123040.1
): F-box family protein  
At3g17620(NM\_112643.1
): F-box family protein

Location in genome: in an IGR, 3785 upstream of At3g24630, 12426 downstream of At3g24590

### Alignment between mature miRNA and predicted targets

```
        Extended mature miRNA:       agtcctagagagtatctcttccacca
        Target(rc):NM_112643.1       aacCCTAGAGAGTCTCTCCTCCAaca
        Target(rc):NM_123040.1       aacCCTAGAGAGTACCTCCTCCAaca
        Target(rc):NM_123030.1       aacCCTAGAGAGTACCTCCTCCAaca
```

### Precursor sequence and structure. Mature sequence in capital letters

```
tcgaagtCCTAGAGAGTATCTCTTCCAccaaaaaaaaatgacgatcatctctgatctttcgtaggatttggtagaagagattctatctgggacttcga   
 (((((((((((((.((.((((((((.((((((.....((((.(((((....)))))..))))....)))))).)))))))).)).)))))))))))))
```

---

## locus\_id: 238262

family\_id: 70

### **Targets:**

At1g13560(NM\_101226.3
): aminoalcoholphosphotransferase (AAPT1)  
At1g13560(NM\_202088.1
): aminoalcoholphosphotransferase (AAPT1)  
At3g25585(NM\_113456.2
): aminoalcoholphosphotransferase, putative  
At3g25585(NM\_180413.1
): aminoalcoholphosphotransferase, putative

Location in genome: in an IGR, 3784 upstream of At3g24630, 12427 downstream of At3g24590

### Alignment between mature miRNA and predicted targets

```
        Extended mature miRNA:       gtcctagagagtatctcttccaccaa
        Target(rc):NM_113456.2       aaaCCAGAAAGTATCTCTTCCACaca
        Target(rc):NM_202088.1       gaaCCAGAAAGTATCTCTTCCACaca
        Target(rc):NM_180413.1       aaaCCAGAAAGTATCTCTTCCACaca
        Target(rc):NM_101226.3       gaaCCAGAAAGTATCTCTTCCACaca
```

### Precursor sequence and structure. Mature sequence in capital letters

```
tcgaagtcCTAGAGAGTATCTCTTCCACcaaaaaaaaatgacgatcatctctgatctttcgtaggatttggtagaagagattctatctgggacttcga   
 (((((((((((((.((.((((((((.((((((.....((((.(((((....)))))..))))....)))))).)))))))).)).)))))))))))))
```

---

## locus\_id: 272320

family\_id: 66

### **Targets:**

At1g04930(NM\_100371.2
): hydroxyproline-rich glycoprotein family protein  
At1g12380(NM\_101110.2
): expressed protein  
At4g28100(NM\_118950.2
): expressed protein  
(NM\_125593.2
): transcriptional factor B3 family protein / auxin-responsive factor, putative (ARF1)  
At4g14000(NM\_117475.2
): expressed protein  
At5g62000(NM\_180913.1
): transcriptional factor B3 family protein / auxin-responsive factor, putative (ARF1)

Location in genome: in an IGR, 97298 upstream of At4g06634, 24770 downstream of At4g06672

### Alignment between mature miRNA and predicted targets

```
        Extended mature miRNA:       gacggtggagcaggagctggctccgg
        Target(rc):NM_117475.2       ggaGGTGGAGGAAGAGCTGGCTCtga
        Target(rc):NM_125593.2       gcaGGAGGAGCAAGAGCTGGCTCtac
        Target(rc):NM_118950.2    tccGGCGTTGGAGCAGGAGCTGGtaa
        Target(rc):NM_180913.1       gcaGGAGGAGCAAGAGCTGGCTCtac
        Target(rc):NM_101110.2    atcGCCGGAGGAGCAGGAGCTGGagg
        Target(rc):NM_100371.2      tggCGGCGGAGCAGGAGGTGGCTgag
```

### Precursor sequence and structure. Mature sequence in capital letters

```
gaggacGGTGGAGCAGGAGCTGGCTCcggagggaaacgagatcgtgatggttcatgactacgttagtgtttcctcgagcagttctgctacaccatcctt   
 (((((.((((.(((((.(((((.(((..((((((((((((.((((((....)))))))).))).....)))))))))))))))))))).)))).)))))
```

---

## locus\_id: 254844

family\_id: 50

### **Targets:**

At1g06220(NM\_100503.2
): elongation factor Tu family protein  
At1g25470(NM\_102358.3
): AP2 domain-containing transcription factor, putative  
At1g06220(NM\_179269.1
): elongation factor Tu family protein

Location in genome: in an IGR, 8478 upstream of At3g48070, 8728 downstream of At3g48040

### Alignment between mature miRNA and predicted targets

```
        Extended mature miRNA:       caagccatcatcatgttcctgtgaag
        Target(rc):NM_179269.1       actGCCATCATCAAGTTCCTGTCcac
        Target(rc):NM_100503.2       actGCCATCATCAAGTTCCTGTCcac
        Target(rc):NM_102358.3         tttCTTCATCATGTTCCTGTGAAaca
```

### Precursor sequence and structure. Mature sequence in capital letters

```
gtcgagcttcattggaaccaaatggtggctcatccatctattactaaagctatacagcaggtggacaaGCCATCATCATGTTCCTGTGaagctcgat   
 ((((((((((((.(((((...(((((((((..(((((((.........(((....))))))))))..)))))))))...))))).))))))))))))
```

---

## locus\_id: 24649

family\_id: 41

### **Targets:**

At1g06170(NM\_100498.3
): basic helix-loop-helix (bHLH) family protein  
At1g06170(NM\_202040.1
): basic helix-loop-helix (bHLH) family protein

Location in genome: in an IGR, 5383 upstream of At1g36340, 5687 downstream of At1g36370

### Alignment between mature miRNA and predicted targets

```
        Extended mature miRNA:       aagattgattttcttcttctgcaccac
        Target(rc):NM_202040.1       acaATTGATTTTCTTCTTCTGCACgat
        Target(rc):NM_100498.3       acaATTGATTTTCTTCTTCTGCACgat
```

### Precursor sequence and structure. Mature sequence in capital letters

```
cagagatgtgttctgctgaaggagtaggctccatctttctcttcttagaggaagATTGATTTTCTTCTTCTGCACcactatctg   
 ((((...(((...(((.(((((((.(((.((.((((((((((....)))))))))).)).)))))))))).))).)))..))))
```

---

## locus\_id: 259051

family\_id: 31

### **Targets:**

(NM\_121392.2
): expressed protein  
At5g13890(NM\_180486.1
): expressed protein

Location in genome: in an IGR, 868 upstream of At3g54870, 5899 downstream of At3g54850

### Alignment between mature miRNA and predicted targets

```
        Extended mature miRNA:       cgcagtggaagaacacggcggagaag
        Target(rc):NM_180486.1       ggaAGAGGAAGAACACGGCGGCGgaa
        Target(rc):NM_121392.2       ggaAGAGGAAGAACACGGCGGCGgaa
```

### Precursor sequence and structure. Mature sequence in capital letters

```
tttctccgtttgtttttcctgcaccgtgtgatagcgacgatagtccaccgcAGTGGAAGAACACGGCGGAGaag   
 .((((((((((((((((((.((..((.(((.....(((....))))))))..))))))))))).))))))))).
```

---

## locus\_id: 127196

family\_id: 26

### **Targets:**

At1g47740(NM\_202258.1
): expressed protein  
At1g47740(NM\_103667.2
): expressed protein

Location in genome: in an IGR, 13169 upstream of At2g24970, 19613 downstream of At2g25050

### Alignment between mature miRNA and predicted targets

```
        Extended mature miRNA:       catagctccatatccttcaatggagg
        Target(rc):NM_103667.2       aaaAGCTCCATCTCCTCCAATGGcta
        Target(rc):NM_202258.1       aaaAGCTCCATCTCCTCCAATGGcta
```

### Precursor sequence and structure. Mature sequence in capital letters

```
ggaagcatAGCTCCATATCCTTCAATGGaggtgtggtccttcaacaaaaatacccccctcttgaaactctgtttcaccacacctccattgaaggacctgaagctatgcttcc   
 ((((((((((((.((..((((((((((((((((((((.........................(((((...)))))))))))))))))))))))))..)).))))))))))))
```

---

## locus\_id: 162691

family\_id: 24

### **Targets:**

At3g10550(NM\_111891.3
): expressed protein  
At5g25757(NM\_147918.2
): expressed protein  
At5g25754(NM\_147917.2
): expressed protein

Location in genome: in an IGR, 7633 upstream of At2g25110, 6473 downstream of At2g25080

### Alignment between mature miRNA and predicted targets

```
        Extended mature miRNA:       agagcagtgagcacgcaagagaagca
        Target(rc):NM_111891.3      tcaAGCAATAAGCACGCAAGAGAAaca
        Target(rc):NM_147918.2      ggaGGCAGTGAACACGCAAGAGAccg
        Target(rc):NM_147917.2       gagGCAGTGAACACGCAAGAGACcga
```

### Precursor sequence and structure. Mature sequence in capital letters

```
gacagaaagaGCAGTGAGCACGCAAGAGAAgcaagtgcaatgatatgcaaattgcctttgtgtgctcactctcttctgtc   
 (((((((.(((.((((((((((((((....((((.((((......))))..)))).))))))))))))))))))))))))
```

---

## locus\_id: 257945

family\_id: 20

### **Targets:**

At3g59360(NM\_180390.2
): nucleotide-sugar transporter family protein  
At3g59360(NM\_115798.2
): nucleotide-sugar transporter family protein

Location in genome: in an IGR, 4601 upstream of At3g52980, 421 downstream of At3g52960

### Alignment between mature miRNA and predicted targets

```
        Extended mature miRNA:       tggagattggagaccaacaagcaaca
        Target(rc):NM_115798.2       gacAGGCTGGAGACCAACAAGCAtgc
        Target(rc):NM_180390.2       gacAGGCTGGAGACCAACAAGCAtgc
```

### Precursor sequence and structure. Mature sequence in capital letters

```
atggAGATTGGAGACCAACAAGCAacaatcctgacttttggaaaggacgttggggttggttgctagttggatgccacgtctacgt   
 (((.(((((((...(((((.(((((((((((..((((((....)))).))..))))).)))))).)))))...))).)))).)))
```

---

## locus\_id: 123053

family\_id: 15

### **Targets:**

(NM\_124435.2
): squamosa promoter-binding protein, putative  
At1g27370(NM\_102499.3
): squamosa promoter-binding protein-like 10 (SPL10)  
At5g50670(NM\_124445.1
): squamosa promoter-binding protein, putative  
At1g27360(NM\_202191.1
): squamosa promoter-binding protein-like 11 (SPL11)  
At2g42200(NM\_180034.1
): squamosa promoter-binding protein-like 9 (SPL9)  
At1g27360(NM\_102498.2
): squamosa promoter-binding protein-like 11 (SPL11)  
At5g50570(NM\_180830.1
): squamosa promoter-binding protein, putative  
At3g57920(NM\_115654.1
): squamosa promoter-binding protein, putative  
At2g33810(NM\_128940.2
): squamosa promoter-binding protein-like 3 (SPL3)  
At1g69170(NM\_105584.4
): squamosa promoter-binding protein-like 6 (SPL6)  
At2g42200(NM\_129782.2
): squamosa promoter-binding protein-like 9 (SPL9)  
At1g27370(NM\_202192.1
): squamosa promoter-binding protein-like 10 (SPL10)  
(NM\_180791.1
): squamosa promoter-binding protein-like 2 (SPL2)  
At5g43270(NM\_123693.2
): squamosa promoter-binding protein-like 2 (SPL2)  
At1g69170(NM\_202380.1
): squamosa promoter-binding protein-like 6 (SPL6)

Location in genome: Contained by At2g19420 (NM\_127500: . hypothetical protein) in an intron on the reverse strand

### Alignment between mature miRNA and predicted targets

```
        Extended mature miRNA:       aggcgacagaagagagtgagcacaca
        Target(rc):NM_102498.2        gttGACAGAAGAGAGAGAGCACGgtg
        Target(rc):NM_123693.2       attTGACAGAAGAGAGAGAGCACcat
        Target(rc):NM_202192.1       agtTGACAGAAGAGAGAGAGCACtgt
        Target(rc):NM_202191.1        gttGACAGAAGAGAGAGAGCACGgtg
        Target(rc):NM_202380.1       agcTGACAGAAGAGAGAGAGCACgag
        Target(rc):NM_115654.1        tttGACAGAAGAGAGAGAGCACAgct
        Target(rc):NM_129782.2        tttGACAGAAGAGAGAGAGCACAgtt
        Target(rc):NM_102499.3        gttGACAGAAGAGAGAGAGCACTgta
        Target(rc):NM_124435.2        gatGACAGAAGAGAGAGAGCACAatc
        Target(rc):NM_180791.1       attTGACAGAAGAGAGAGAGCACcat
        Target(rc):NM_128940.2        actGACAGAAGAGAGTAAGCAAAgcc
        Target(rc):NM_105584.4        gctGACAGAAGAGAGAGAGCACGagt
        Target(rc):NM_180830.1        gatGACAGAAGAGAGAGAGCACAatc
        Target(rc):NM_124445.1        gatGACAGAAGAGAGAGAGCACAatc
        Target(rc):NM_180034.1        tttGACAGAAGAGAGAGAGCACAgtt
```

### Precursor sequence and structure. Mature sequence in capital letters

```
aggCGACAGAAGAGAGTGAGCACacatggctctttttctagcatgctcatgctcgaaagctctgcgtgcttactctcttcttgtctcct   
 (((.(((((((((((((((((((.((.((((....(((.(((((....))))).))))))).)).))))))))))))))).)))).)))
```

---

## locus\_id: 325365

family\_id: 15

### **Targets:**

At1g53160(NM\_104194.2
): squamosa promoter-binding protein-like 4 (SPL4)  
At2g42200(NM\_129782.2
): squamosa promoter-binding protein-like 9 (SPL9)  
(NM\_124435.2
): squamosa promoter-binding protein, putative  
At3g57920(NM\_115654.1
): squamosa promoter-binding protein, putative  
At5g50670(NM\_124445.1
): squamosa promoter-binding protein, putative  
At2g42200(NM\_180034.1
): squamosa promoter-binding protein-like 9 (SPL9)  
At5g50570(NM\_180830.1
): squamosa promoter-binding protein, putative  
(NM\_180791.1
): squamosa promoter-binding protein-like 2 (SPL2)  
At1g27360(NM\_202191.1
): squamosa promoter-binding protein-like 11 (SPL11)  
At1g27370(NM\_202192.1
): squamosa promoter-binding protein-like 10 (SPL10)  
At5g43270(NM\_123693.2
): squamosa promoter-binding protein-like 2 (SPL2)  
At1g27360(NM\_102498.2
): squamosa promoter-binding protein-like 11 (SPL11)  
At1g27370(NM\_102499.3
): squamosa promoter-binding protein-like 10 (SPL10)

Location in genome: in an IGR, 9721 upstream of At4g30990, 5935 downstream of At4g30960

1 homologs in rice

### Alignment between mature miRNA and predicted targets

```
        Extended mature miRNA:       aactgacagaagagagtgagcacatg
        Target(rc):NM_102498.2       ggtTGACAGAAGAGAGAGAGCACggt
        Target(rc):NM_123693.2       attTGACAGAAGAGAGAGAGCACcat
        Target(rc):NM_104194.2       agaTGACAGAAGAGAGAGAGCAGAcag
        Target(rc):NM_202192.1       agtTGACAGAAGAGAGAGAGCACtgt
        Target(rc):NM_202191.1       ggtTGACAGAAGAGAGAGAGCACggt
        Target(rc):NM_115654.1       gttTGACAGAAGAGAGAGAGCACAgct
        Target(rc):NM_129782.2       attTGACAGAAGAGAGAGAGCACAgtt
        Target(rc):NM_102499.3       agtTGACAGAAGAGAGAGAGCACtgt
        Target(rc):NM_124435.2       ggaTGACAGAAGAGAGAGAGCACAatc
        Target(rc):NM_180791.1       attTGACAGAAGAGAGAGAGCACcat
        Target(rc):NM_180830.1       ggaTGACAGAAGAGAGAGAGCACAatc
        Target(rc):NM_124445.1       ggaTGACAGAAGAGAGAGAGCACAatc
        Target(rc):NM_180034.1       attTGACAGAAGAGAGAGAGCACAgtt
```

### Precursor sequence and structure. Mature sequence in capital letters

```
aacTGACAGAAGAGAGTGAGCACatgcaggcactgttatgtgtctataactttgcgtgtgcgtgctcacctctctttctgtcagtt   
 (((((((((((((((((((((((..(((.(((..((((((....))))))..))).)))..))))))).))))).)))))))))))
```

---

## locus\_id: 337350

family\_id: 15

### **Targets:**

At2g42200(NM\_129782.2
): squamosa promoter-binding protein-like 9 (SPL9)  
At3g57920(NM\_115654.1
): squamosa promoter-binding protein, putative  
At2g33810(NM\_128940.2
): squamosa promoter-binding protein-like 3 (SPL3)  
(NM\_124435.2
): squamosa promoter-binding protein, putative  
At2g42200(NM\_180034.1
): squamosa promoter-binding protein-like 9 (SPL9)  
At5g50570(NM\_180830.1
): squamosa promoter-binding protein, putative  
At5g50670(NM\_124445.1
): squamosa promoter-binding protein, putative

Location in genome: in an IGR, 2522 upstream of At5g10940, 2541 downstream of At5g10950

4 homologs in brassica

### Alignment between mature miRNA and predicted targets

```
        Extended mature miRNA:       agttgacagaagagagtgagcacacaa
        Target(rc):NM_115654.1      agtTTGACAGAAGAGAGAGAGCACAgct
        Target(rc):NM_129782.2      gatTTGACAGAAGAGAGAGAGCACAgtt
        Target(rc):NM_124435.2       ggaTGACAGAAGAGAGAGAGCACAatc
        Target(rc):NM_128940.2       gacTGACAGAAGAGAGTAAGCAAAgcc
        Target(rc):NM_124445.1       ggaTGACAGAAGAGAGAGAGCACAatc
        Target(rc):NM_180830.1       ggaTGACAGAAGAGAGAGAGCACAatc
        Target(rc):NM_180034.1      gatTTGACAGAAGAGAGAGAGCACAgtt
```

### Precursor sequence and structure. Mature sequence in capital letters

```
gaaaagaagtTGACAGAAGAGAGTGAGCACAcaaaggggaagttgtataaaagttttgtatatggttgcttttgcgtgctcactctctttttgtcataacttctccttc   
 (((.((((((((((((((((((((((((((.((((((.((...(((((((.....)))))))...)).)))))).)))))))))))).))))))))..))))))..)))
```

---

## locus\_id: 384343

family\_id: 15

### **Targets:**

At2g33810(NM\_128940.2
): squamosa promoter-binding protein-like 3 (SPL3)  
At5g50570(NM\_180830.1
): squamosa promoter-binding protein, putative  
At3g57920(NM\_115654.1
): squamosa promoter-binding protein, putative  
(NM\_124435.2
): squamosa promoter-binding protein, putative  
At2g42200(NM\_180034.1
): squamosa promoter-binding protein-like 9 (SPL9)  
At2g42200(NM\_129782.2
): squamosa promoter-binding protein-like 9 (SPL9)  
At5g50670(NM\_124445.1
): squamosa promoter-binding protein, putative  
At5g43270(NM\_123693.2
): squamosa promoter-binding protein-like 2 (SPL2)  
At1g27360(NM\_102498.2
): squamosa promoter-binding protein-like 11 (SPL11)  
(NM\_180791.1
): squamosa promoter-binding protein-like 2 (SPL2)  
At1g27360(NM\_202191.1
): squamosa promoter-binding protein-like 11 (SPL11)

Location in genome: in an IGR, 17922 upstream of At5g12040, 10269 downstream of At5g11950

1 homologs in brassica

1 homologs in rice

### Alignment between mature miRNA and predicted targets

```
        Extended mature miRNA:       aggtgacagaagagagtgagcacaca
        Target(rc):NM_102498.2       ggtTGACAGAAGAGAGAGAGCACggt
        Target(rc):NM_123693.2       attTGACAGAAGAGAGAGAGCACcat
        Target(rc):NM_202191.1       ggtTGACAGAAGAGAGAGAGCACggt
        Target(rc):NM_115654.1       gttTGACAGAAGAGAGAGAGCACAgct
        Target(rc):NM_129782.2       attTGACAGAAGAGAGAGAGCACAgtt
        Target(rc):NM_124435.2       ggaTGACAGAAGAGAGAGAGCACAatc
        Target(rc):NM_180791.1       attTGACAGAAGAGAGAGAGCACcat
        Target(rc):NM_128940.2       gacTGACAGAAGAGAGTAAGCAAAgcc
        Target(rc):NM_124445.1       ggaTGACAGAAGAGAGAGAGCACAatc
        Target(rc):NM_180830.1       ggaTGACAGAAGAGAGAGAGCACAatc
        Target(rc):NM_180034.1       attTGACAGAAGAGAGAGAGCACAgtt
```

### Precursor sequence and structure. Mature sequence in capital letters

```
aggaggTGACAGAAGAGAGTGAGCACacatggtggtttcttgcatgcttttttgattagggtttcatgcttgaagctatgtgtgcttactctctctctgtcacccct   
 (((.(((((((((((((((((((((((((((((...(((..(((((((((.......))))...)))))..))))))))))))))))))))))).))))))))))))
```

---

## locus\_id: 354445

family\_id: 13

### **Targets:**

At1g60940(NM\_179503.1
): serine/threonine protein kinase, putative  
At1g60940(NM\_104774.3
): serine/threonine protein kinase, putative

Location in genome: in an IGR, 1923 upstream of At5g35530, 27908 downstream of At5g35560

### Alignment between mature miRNA and predicted targets

```
        Extended mature miRNA:       aggacgagttggagcttttgcctctaat
        Target(rc):NM_179503.1       aacACGAGCTGGAGTTTTTGCCTCTtcc
        Target(rc):NM_104774.3       aacACGAGCTGGAGTTTTTGCCTCTtcc
```

### Precursor sequence and structure. Mature sequence in capital letters

```
ggtggttggaggcaggcacatctcttctctgagaggaactatcctcagatatcctgaagattcttgaggcagaggACGAGTTGGAGCTTTTGCCTCTaatctcc   
 ((.((((((((((((((.((.((((((((((.(((((....)))))......(((.(((...))).)))))))))).))).))..)))..))))))))))).))
```

---

## locus\_id: 396158

family\_id: 12

### **Targets:**

At2g14120(NM\_126985.2
): dynamin-like protein 2b (ADL2b)  
At2g14120(NM\_126984.2
): dynamin-like protein 2b (ADL2b)

Location in genome: in an IGR, 1938 upstream of At5g28622, 2521 downstream of At5g28615

### Alignment between mature miRNA and predicted targets

```
        Extended mature miRNA:       gtcacgacagctacttgcggctgcgg
        Target(rc):NM_126985.2       ccgACGACAGCTACTTGCGGAAGagc
        Target(rc):NM_126984.2       ccgACGACAGCTACTTGCGGAAGagc
```

### Precursor sequence and structure. Mature sequence in capital letters

```
gtttggtcACGACAGCTACTTGCGGCTGcggcaaccgcaggttatgctgttcttttagtgaacgcaagacgatgcggccaaatgctacggcaaacgcaagtagctgcggtgaaaaac   
 ((((..((((..((((((((((((((((..(((.(((((.(((.(((.((((.......))))))).)))..))))).....)))..))))...))))))))))))..)))).))))
```

---

## locus\_id: 11657

family\_id: 7

### **Targets:**

At5g06510(NM\_120734.1
): CCAAT-binding transcription factor (CBF-B/NF-YA) family protein  
At3g05690(NM\_111443.3
): CCAAT-binding transcription factor (CBF-B/NF-YA) family protein  
At1g17590(NM\_101621.2
): CCAAT-binding transcription factor (CBF-B/NF-YA) family protein  
At1g17590(NM\_202121.1
): CCAAT-binding transcription factor (CBF-B/NF-YA) family protein  
At1g17590(NM\_202122.1
): CCAAT-binding transcription factor (CBF-B/NF-YA) family protein  
At1g72830(NM\_202405.1
): CCAAT-binding transcription factor (CBF-B/NF-YA) family protein  
At1g54160(NM\_104294.2
): CCAAT-binding transcription factor (CBF-B/NF-YA) family protein  
At1g72830(NM\_105941.4
): CCAAT-binding transcription factor (CBF-B/NF-YA) family protein

Location in genome: in an IGR, 612 upstream of At1g19370, 5204 downstream of At1g19390

1 homologs in brassica

### Alignment between mature miRNA and predicted targets

```
        Extended mature miRNA:       tgtgtggtagccaaggatgacttgcctgc
        Target(rc):NM_101621.2          gtaGCAGCCAAGGATGACTTCCCTttt
        Target(rc):NM_105941.4       ctaGTAGTAGCCAAGGATGACTTCCCctt
        Target(rc):NM_104294.2          taaGTAGCCAAGGATGACTTCCCgtc
        Target(rc):NM_120734.1            atgAGCCAAAGATGATTTGCCTGttt
        Target(rc):NM_111443.3            atgAGCCAAAGATGATTTGCCTGttc
        Target(rc):NM_202121.1          gtaGCAGCCAAGGATGACTTCCCTttt
        Target(rc):NM_202122.1          gtaGCAGCCAAGGATGACTTCCCTttt
        Target(rc):NM_202405.1       ctaGTAGTAGCCAAGGATGACTTCCCctt
```

### Precursor sequence and structure. Mature sequence in capital letters

```
gagaacttgtGTGGTAGCCAAGGATGACTTGCCtgcgttttagaccatatatatcaaagactcactcgatcgatagtcttagagttggttggtcgtcaggcagtctccttggctattcaaacaattctc   
 (((((.((((.(((((((((((((.((((.(((((((.....(((((......((.((((((...((....)).)))))).))..)))))...)).))))))))))))))))))).))).)))))))))
```

---

## locus\_id: 392208

family\_id: 7

### **Targets:**

(NM\_121287.3
): CCAAT-binding transcription factor (CBF-B/NF-YA) family protein  
At5g12840(NM\_180480.1
): CCAAT-binding transcription factor (CBF-B/NF-YA) family protein  
At1g54160(NM\_104294.2
): CCAAT-binding transcription factor (CBF-B/NF-YA) family protein  
At1g17590(NM\_101621.2
): CCAAT-binding transcription factor (CBF-B/NF-YA) family protein  
At1g17590(NM\_202121.1
): CCAAT-binding transcription factor (CBF-B/NF-YA) family protein  
At1g72830(NM\_105941.4
): CCAAT-binding transcription factor (CBF-B/NF-YA) family protein  
At1g72830(NM\_202405.1
): CCAAT-binding transcription factor (CBF-B/NF-YA) family protein  
At1g17590(NM\_202122.1
): CCAAT-binding transcription factor (CBF-B/NF-YA) family protein

Location in genome: in an IGR, 3449 upstream of At5g24830, 2213 downstream of At5g24820

2 homologs in rice

### Alignment between mature miRNA and predicted targets

```
        Extended mature miRNA:       aatgcagccaaggatgacttgccgga
        Target(rc):NM_180480.1         aaaAGCCAAGAATGATTTGCCGGttt
        Target(rc):NM_101621.2       gtaGCAGCCAAGGATGACTTCCCttt
        Target(rc):NM_105941.4       gtaGTAGCCAAGGATGACTTCCCctt
        Target(rc):NM_104294.2       taaGTAGCCAAGGATGACTTCCCGtca
        Target(rc):NM_121287.3         aaaAGCCAAGAATGATTTGCCGGttt
        Target(rc):NM_202121.1       gtaGCAGCCAAGGATGACTTCCCttt
        Target(rc):NM_202122.1       gtaGCAGCCAAGGATGACTTCCCttt
        Target(rc):NM_202405.1       gtaGTAGCCAAGGATGACTTCCCctt
```

### Precursor sequence and structure. Mature sequence in capital letters

```
agagtataatGCAGCCAAGGATGACTTGCCggaacgttgttaaccatgcatatgaataatgtgatgattaattatgtgatgaacatatttctggcaagttgtccttcggctacattttgctct   
 ((((((.((((.(((((((((..(((((((((((...((((...(((.(((((((.((((......)))).))))))))))))))...)))))))))))..))))).)))).)))).))))))
```

---

## locus\_id: 91578

family\_id: 1

### **Targets:**

At5g36730(NM\_123030.1
): F-box family protein  
At3g17570(NM\_112637.1
): F-box family protein  
At3g49520(NM\_114812.1
): F-box family protein  
At3g49510(NM\_114811.2
): F-box family protein  
At3g24580(NM\_113369.1
): F-box family protein  
At5g36820(NM\_123040.1
): F-box family protein  
At2g18780(NM\_127434.3
): F-box family protein  
At3g16820(NM\_112556.1
): F-box family protein  
At3g21170(NM\_113011.1
): F-box family protein  
At3g22350(NM\_113133.1
): F-box family protein  
At3g16880(NM\_112564.1
): F-box protein-related  
At3g22710(NM\_113170.1
): F-box family protein

Location in genome: in an IGR, 5824 upstream of At1g60090, 7943 downstream of At1g60060

### Alignment between mature miRNA and predicted targets

```
        Extended mature miRNA:       cgaaatctctctgttgtgaagtcaaacatgag
        Target(rc):NM_112556.1      tccAAATCTCTCTGTTGTAAAATCAAAaca
        Target(rc):NM_114811.2      tccAAATCTCTCTGTTGTAAAATCAAAaca
        Target(rc):NM_113011.1      tccAAATCTCTCTGTTGAGAAATCAAAact
        Target(rc):NM_127434.3      tctAAATCTCTCCGTTGTGAAATCAAAaca
        Target(rc):NM_123040.1      tccAAATCTCTCTGTTGTAAAATCAAAaca
        Target(rc):NM_123030.1      tccAAATCTCTCTGTTGTAAAATCAAAaca
        Target(rc):NM_112564.1      tccAAATCTCTCTGTTGTAAAATCAAAaga
        Target(rc):NM_113133.1      attAAATCTCTCTGTTGTAAAATCAAAact
        Target(rc):NM_113170.1       ttgAATCTCTCTGTTGTAAAATCAAACATtag
        Target(rc):NM_112637.1      tccAAATCTCTCTGTTGCGAAATCAAACctg
        Target(rc):NM_113369.1      cctAAATCTCTCTCTTGTGAAATCAAAaca
        Target(rc):NM_114812.1      tccAAATCTCTCGGTTGTGAAATCAAAaca
```

### Precursor sequence and structure. Mature sequence in capital letters

```
tagatgtcgaAATCTCTCTGTTGTGAAGTCAAACATgagtatgaattaacattaatggatctttttgattcataatactcatgtttggtttcacagtagatagatatcgacgtcta   
 ((((((((((.((((.(((((((((((..(((((((((((((............(((((((.....))))))).)))))))))))))..))))))))))).)))).))))))))))
```

---

## locus\_id: 71725

family\_id: 479

### **Targets:**

At2g13630(NM\_126942.1
): F-box family protein-related

Location in genome: in an IGR, 3731 upstream of At1g30160, 2950 downstream of At1g30140

### Alignment between mature miRNA and predicted targets

```
        Extended mature miRNA:       gccgacggatcctccggtgagcacga
        Target(rc):NM_126942.1       aaaGACGGATCCTCCTGAGAGCAgga
```

### Precursor sequence and structure. Mature sequence in capital letters

```
gattgtgcacacggtgaagaccccgatggctgctccacccaaaagagggatgccGACGGATCCTCCGGTGAGCAcgatc   
 ((((((((.(((.(.((.((..(((.((((...(((..(.....)..))).)))).))).)).))).))).))))))))
```

---

## locus\_id: 95402

family\_id: 478

### **Targets:**

At1g22550(NM\_102104.3
): proton-dependent oligopeptide transport (POT) family protein

Location in genome: in an IGR, 4393 upstream of At1g64590, 5195 downstream of At1g64570

### Alignment between mature miRNA and predicted targets

```
        Extended mature miRNA:       tgccacatgggttgtttccgccttctt
        Target(rc):NM_102104.3       gaaCACATGGCTTGTGTCCGCCTTgtc
```

### Precursor sequence and structure. Mature sequence in capital letters

```
tggaggagagcggcagcaacaatggcagcgtgagcgttatttctcgagtgaaattcgatcaggttgcCACATGGGTTGTTTCCGCCTTcttca   
 .(((((((.((((.((((((..(((((((.(((.((..(((((......))))).)).))).))))))).....)))))).))))))))))).
```

---

## locus\_id: 7452

family\_id: 477

### **Targets:**

At3g49200(NM\_114779.1
): hypothetical protein

Location in genome: Contained by At1g12430 (NM\_101115: . armadillo/beta-catenin repeat family protein / kinesin motor family protein) in an intron

### Alignment between mature miRNA and predicted targets

```
        Extended mature miRNA:       aggttcctcatctacactttgataat
        Target(rc):NM_114779.1       aatTTCCCCATCTACACTTTGATcct
```

### Precursor sequence and structure. Mature sequence in capital letters

```
aggTTCCTCATCTACACTTTGATaataaccatcaacttgaacttcaaagttgatggttattcaagttgtagtgaataactt   
 (((((..(((.(((((.(((((..((((((((((((((........))))))))))))))))))).))))))))..)))))
```

---

## locus\_id: 78526

family\_id: 476

### **Targets:**

At2g25370(NM\_128095.2
): zinc finger protein-related

Location in genome: in an IGR, 5040 upstream of At1g37000, 4036 downstream of At1g36980

### Alignment between mature miRNA and predicted targets

```
        Extended mature miRNA:       cttttggtcgctgaatccagcgacatgc
        Target(rc):NM_128095.2       taaTTGGTCGCTGAGTCCAGCGACAgac
```

### Precursor sequence and structure. Mature sequence in capital letters

```
ttgcttgtcgctggttcagcgatcaaaagtagcgacagtcgccagggaccagcgaccgtaattttttgtcgttaaaatttttagcgattagtcgctgcttTTGGTCGCTGAATCCAGCGACAtgcaa   
 .(((.(((((((((((((((((((((((((((((((((((((.(((((..((((((...........))))))....))))).)))))).)))))))))))))))))))))).))))))))).))).
```

---

## locus\_id: 82881

family\_id: 475

### **Targets:**

At2g25370(NM\_128095.2
): zinc finger protein-related

Location in genome: in an IGR, 9909 upstream of At1g47390, 15322 downstream of At1g47330

### Alignment between mature miRNA and predicted targets

```
        Extended mature miRNA:       ggattcagcgaccaaaagcagcgacta
        Target(rc):NM_128095.2       tggTTCAGCGACCAAAAATAGCGAtaa
```

### Precursor sequence and structure. Mature sequence in capital letters

```
tgtcgctggaTTCAGCGACCAAAAGCAGCGActaatcgctgaaaccagcgactgatcgctaaaaatttcagcgatcaaaaattgcggtcgctggtcgctacttttggtcgttgaaccagcgaca   
 .((((((((.(((((((((((((((.((((((((.((((((....)))))).((((((((.........))))))))..............)))))))).))))))))))))))))))))))).
```

---

## locus\_id: 3449

family\_id: 474

### **Targets:**

At2g37520(NM\_129307.3
): PHD finger family protein

Location in genome: in an IGR, 855 upstream of At1g06550, 4447 downstream of At1g06570

### Alignment between mature miRNA and predicted targets

```
        Extended mature miRNA:       gaataagcccatctcattcagagcttt
        Target(rc):NM_129307.3       tcgTAAGCCCTCCTCATTCAGAGCaga
```

### Precursor sequence and structure. Mature sequence in capital letters

```
ttgcaatgaaTAAGCCCATCTCATTCAGAGCtttaatggtagatgggccttgggttgcaaagaataagcccatatcattcagagctttaatgacagatgggccttgggttgcaa   
 .((((((...(((((((((((((((.(((((((((((((((..(((((.(((..((....))..))))))))))))))).)))))))))))))..)))))).)))).)))))).
```

---

## locus\_id: 99605

family\_id: 473

### **Targets:**

At5g40910(NM\_123456.1
): disease resistance protein (TIR-NBS-LRR class), putative

Location in genome: in an IGR, 35742 upstream of At1g69860, 4882 downstream of At1g69790

1 homologs in brassica

### Alignment between mature miRNA and predicted targets

```
        Extended mature miRNA:       ccccttgagttcccttaaacgcttcatt
        Target(rc):NM_123456.1       caaCTTGAGTTACCTTAAACGCTTCtgc
```

### Precursor sequence and structure. Mature sequence in capital letters

```
atgtcccCTTGAGTTCCCTTAAACGCTTCattgttcatactttgttatcatctatcgatcgatcaatcaatctgatgaacactgaagtgtttggggggactctaggtgacat   
 (((((.(((.((((((((((((((((((((.(((((((...(((..((((((....))).)))....)))....))))))).)))))))))))))))))))).))).)))))
```

---

## locus\_id: 103839

family\_id: 472

### **Targets:**

At4g29820(NM\_119128.2
): expressed protein

Location in genome: in an IGR, 2640 upstream of At1g76920, 1298 downstream of At1g76910

### Alignment between mature miRNA and predicted targets

```
        Extended mature miRNA:       ccttgaacgccgtcgtttcgtctccga
        Target(rc):NM_119128.2       tcaTGAACGACATCGTTTCGTCTCgtc
```

### Precursor sequence and structure. Mature sequence in capital letters

```
cctTGAACGCCGTCGTTTCGTCTCcgacgacattcacacgcgcacacttccacccttaccatctatccgtctcacacgtgcgtcggatgcgaaacggtggtgttcaggg   
 (((((((((((..((((((((.(((((((.(((......(((.........................))).......)))))))))).))))))))..)))))))))))
```

---

## locus\_id: 69392

family\_id: 471

### **Targets:**

At4g17490(NM\_117854.2
): ethylene-responsive element-binding protein, putative

Location in genome: in an IGR, 5742 upstream of At1g26920, 789 downstream of At1g26910

1 homologs in brassica

### Alignment between mature miRNA and predicted targets

```
        Extended mature miRNA:       gtgtttggagggaacagaggattgagac
        Target(rc):NM_117854.2       catTTTGGAGGAAACAGAGAATTGAaga
```

### Precursor sequence and structure. Mature sequence in capital letters

```
aggtggagtgTTTGGAGGGAACAGAGGATTGAgaccaataccaccagaaaagggtattttacctttggatcatttgcatcaatgtgacacggtattgtttcatccttccttctctccaacctaagcct   
 ((((..((.(((.((((((((..((((((.(((((.((((((....((.(((((((...)))))))...))..(..((....))..)...)))))))))))))))))..)))))))))))))..))))
```

---

## locus\_id: 92543

family\_id: 470

### **Targets:**

At5g23630(NM\_122268.2
): ATPase E1-E2 type family protein / haloacid dehalogenase-like hydrolase familiy protein

Location in genome: in an IGR, 4310 upstream of At1g61240, 1653 downstream of At1g61215

### Alignment between mature miRNA and predicted targets

```
        Extended mature miRNA:       cagggatcgaaacaccatcatggtcaga
        Target(rc):NM_122268.2       tccGGATCGATACACCATCACGGTCtga
```

### Precursor sequence and structure. Mature sequence in capital letters

```
gatgaatccgaccatgatggtgttgcagaaatttatgtatgatgtagttattgtccagGGATCGAAACACCATCATGGTCagatccgtc   
 ((((.(((.(((((((((((((((...((..((..((.(..(((....)))..).))..))))..))))))))))))))).))).))))
```

---

## locus\_id: 95649

family\_id: 469

### **Targets:**

At1g11620(NM\_101035.1
): F-box family protein

Location in genome: Contained by At1g64990 (NM\_105173: . expressed protein) in an intron on the reverse strand

### Alignment between mature miRNA and predicted targets

```
        Extended mature miRNA:       cagctgagcaaggaaccaagaccatgg
        Target(rc):NM_101035.1       tcaCTGAGCATGGGACCAAGACCAgtg
```

### Precursor sequence and structure. Mature sequence in capital letters

```
gtgcagCTGAGCAAGGAACCAAGACCAtggattgtttgctcgagaccgacaataatctctccggtcttaaataaattatccatagttttggttccatttacagcatcat   
 .....((((.....(((((((((((.((((((.(((((.(..((((((.............))))))..).))))).)))))).))))))))))).....)))).....
```

---

## locus\_id: 65311

family\_id: 468

### **Targets:**

At1g10200(NM\_100894.2
): transcription factor LIM, putative

Location in genome: in an IGR, 3436 upstream of At1g20380, 13617 downstream of At1g20350

### Alignment between mature miRNA and predicted targets

```
        Extended mature miRNA:       tttggcattctgtccacctccttctat
        Target(rc):NM_100894.2       ggcGGCATTCTCTCCTCCTCCTTCgag
```

### Precursor sequence and structure. Mature sequence in capital letters

```
gtcatctttGGCATTCTGTCCACCTCCTTCtatacatatatgcatgtgtatatatatatgcgtttcgtgtgaaagaaggaggtgggtatactgccaatagagat   
 (((.(((((((((.(.(..((((((((((((.(((((..((((((((((....))))))))))...)))))..))))))))))))..).).)))))).))))))
```

---

## locus\_id: 40842

family\_id: 467

### **Targets:**

At5g26860(NM\_122568.2
): Lon protease homolog 2, mitochondrial

Location in genome: in an IGR, 25163 upstream of At1g62960, 2219 downstream of At1g63010

### Alignment between mature miRNA and predicted targets

```
        Extended mature miRNA:       tagggcgcctctccattggcaggtcc
        Target(rc):NM_122568.2       gccGCCGCCTCTCCATTGGTAGGctc
```

### Precursor sequence and structure. Mature sequence in capital letters

```
ctagttttagGGCGCCTCTCCATTGGCAGGtcctttacttccaaatatacacatacatatatgaatatcgaaaatttccgatgatcgatttataaatgacctgccaaaggagagttgccctgaaactgg   
 ((((((((((((((.((((((.((((((((((.((((.......(((((........)))))((.(((((........))))).))......)))).)))))))))).)))))).))))))))))))))
```

---

## locus\_id: 12307

family\_id: 466

### **Targets:**

At5g15420(NM\_121546.2
): expressed protein

Location in genome: in an IGR, 11906 upstream of At1g20370, 6620 downstream of At1g20400

### Alignment between mature miRNA and predicted targets

```
        Extended mature miRNA:       cgatgagctggctcttccgggcgaactggctg
        Target(rc):NM_121546.2       tggTGAGCTGGCTCTTCTGGGCGAGCTGGatg
```

### Precursor sequence and structure. Mature sequence in capital letters

```
gatgagctggctcttctgggcgagctggatgatcttgacgagcagacttctctcgacgagctgacttgtatcgaTGAGCTGGCTCTTCCGGGCGAACTGGctgatc   
 (((.(((..(.((.((((((.((((..(.(.(((...((((((((.(((........)))))).)))))...))).).)..)))).)))))).)).)..))).)))
```

---

## locus\_id: 19791

family\_id: 465

### **Targets:**

At3g49270(NM\_114786.3
): expressed protein

Location in genome: in an IGR, 7766 upstream of At1g31350, 7729 downstream of At1g31370

### Alignment between mature miRNA and predicted targets

```
        Extended mature miRNA:       gctgcggtagcggtggcggcaaacact
        Target(rc):NM_114786.3       tccGCGGTGGCGGTGGCGACAAACgaa
```

### Precursor sequence and structure. Mature sequence in capital letters

```
ggcagcggctGCGGTAGCGGTGGCGGCAAACactaccgcaggttgttgttcgttttgttgccgcagacgctgccgcagccgctgcc   
 (((((((((((((((((((...(((((((.((..((.((((....))))..))..)))))))))...)))))))))))))))))))
```

---

## locus\_id: 27537

family\_id: 464

### **Targets:**

At5g49810(NM\_124359.2
): methionine S-methyltransferase

Location in genome: in an IGR, 3201 upstream of At1g43130, 40665 downstream of At1g43190

### Alignment between mature miRNA and predicted targets

```
        Extended mature miRNA:       agtagctgcgtcggcggcttgtttgata
        Target(rc):NM_124359.2       ataAGCTGCGTCGCCGGATTGTTTGcac
```

### Precursor sequence and structure. Mature sequence in capital letters

```
ctcttttctgacaagcacctgactagcacttgcatcttgattagtAGCTGCGTCGGCGGCTTGTTTGataaggg   
 (((((.((.(((((((..((((((((((((..(.....)...))).)))).)))))..))))))).)).)))))
```

---

## locus\_id: 75066

family\_id: 463

### **Targets:**

At5g11020(NM\_180475.1
): protein kinase family protein

Location in genome: in an IGR, 1749 upstream of At1g33860, 22710 downstream of At1g33811

### Alignment between mature miRNA and predicted targets

```
        Extended mature miRNA:       gacggccatgtgatcgacgaatcctggaa
        Target(rc):NM_180475.1       ataGGCCATGTGATCGCCGAACCCTGaga
```

### Precursor sequence and structure. Mature sequence in capital letters

```
ggtcgaggaattctcgatccggacagccgtatggtcgacgaatcctcgaactggacGGCCATGTGATCGACGAATCCTGgaacc   
 ((((.((((.(((((((((((....(((((.(((((((.......))).)))).)))))..)).)))))).))))))).).)))
```

---

## locus\_id: 104993

family\_id: 462

### **Targets:**

At3g11000(NM\_111937.3
): expressed protein

Location in genome: in an IGR, 3066 upstream of At1g78490, 2191 downstream of At1g78470

### Alignment between mature miRNA and predicted targets

```
        Extended mature miRNA:       tgaaaacaaacggaggagtttgtttgttgt
        Target(rc):NM_111937.3       tttAAACAAACGGAGCAGCTTGTTTGTctg
```

### Precursor sequence and structure. Mature sequence in capital letters

```
gatcgtgaAAACAAACGGAGGAGTTTGTTTGTtgtactcggtctagttcaaaccaaaacatctttgcaaatatgtatggtttgagctagaccgatgtcaacaaacaagctgcttcgtttgtattcaccatc   
 (((.(((((.((((((((((.(((((((((((((.((((((((((((((((((((..((((.((....)).)))).)))))))))))))))))).))))))))))))))).)))))))))).))))).)))
```

---

## locus\_id: 51517

family\_id: 461

### **Targets:**

At3g11000(NM\_111937.3
): expressed protein

Location in genome: in an IGR, 5963 upstream of At1g78460, 202 downstream of At1g78480

### Alignment between mature miRNA and predicted targets

```
        Extended mature miRNA:       aatacaaacgaagcagcttgtttgttga
        Target(rc):NM_111937.3       taaACAAACGGAGCAGCTTGTTTGTctg
```

### Precursor sequence and structure. Mature sequence in capital letters

```
gatggtgaatACAAACGAAGCAGCTTGTTTGTtgacatcggtctagctcaaaccatacatatttgcaaagatgttttggtttgaactagaccgagtacaacaaacaaactcctccgtttgttttcacgatc   
 (((.(((((.(((((((.((.((.((((((((((((.(((((((((.((((((((...((((((....))))))..)))))))).))))))))))).)))))))))).)).)).))))))).))))).)))
```

---

## locus\_id: 35441

family\_id: 460

### **Targets:**

At2g03550(NM\_126405.1
): expressed protein

Location in genome: in an IGR, 8970 upstream of At1g55030, 12260 downstream of At1g55080

### Alignment between mature miRNA and predicted targets

```
        Extended mature miRNA:       cgaccggtcctgttcctgtgacatatc
        Target(rc):NM_126405.1       tttCCGGTCCGGTTCCTGTGATATgag
```

### Precursor sequence and structure. Mature sequence in capital letters

```
ggagaagagaagtcaaaggaacatgaccgatcggtcttatgaaatttacagaaattgacgatgaagcatggatcgaCCGGTCCTGTTCCTGTGACATatcttcccc   
 ((.(((((...((((.(((((((.(((((.(((((((.(((...((((..(.......)..)))).)))))))))).))))).))))))).))))...))))))).
```

---

## locus\_id: 2472

family\_id: 459

### **Targets:**

At1g51310(NM\_104009.1
): tRNA methyl transferase family protein

Location in genome: in an IGR, 1520 upstream of At1g05065, 309 downstream of At1g05070

### Alignment between mature miRNA and predicted targets

```
        Extended mature miRNA:       atgggccagaaattctcaaagcccca
        Target(rc):NM_104009.1       tgaTTCCAGAAATTCTCAAAGCCttc
```

### Precursor sequence and structure. Mature sequence in capital letters

```
gatttggggctttgagattttctgggccatcgccacactttctctggccaatattgaaaggtgtggtcggcaacctttaacagatttgggttggatgGGCCAGAAATTCTCAAAGCCccaaatc   
 (((((((((((((((((.(((((((.(((((.(((((((((.((...........)))))))))))....((((((...........))))))))))).))))))).)))))))))))))))))
```

---

## locus\_id: 96159

family\_id: 458

### **Targets:**

At3g59770(NM\_115839.3
): sacI homology domain-containing protein / WW domain-containing protein

Location in genome: Contained by At1g65500 (NM\_105224: . expressed protein) in an intron

### Alignment between mature miRNA and predicted targets

```
        Extended mature miRNA:       gtcttgccctatctctttctgttttc
        Target(rc):NM_115839.3       acaGTGCCCTATCTCTTTCTGCTgca
```

### Precursor sequence and structure. Mature sequence in capital letters

```
tatgtcTTGCCCTATCTCTTTCTGTTttcttgtgtatatcatttttaattagaaaatagaaagagtgtaggaaagatata   
 ((((((((..((((((((((((((((((((.((..............)).))))))))))))))).))))).))))))))
```

---

## locus\_id: 33909

family\_id: 457

### **Targets:**

At5g09660(NM\_121003.2
): malate dehydrogenase, glyoxysomal

Location in genome: in an IGR, 1327 upstream of At1g52560, 2789 downstream of At1g52565

### Alignment between mature miRNA and predicted targets

```
        Extended mature miRNA:       caagggacagtagatgtcacaggttc
        Target(rc):NM_121003.2       atgGGGACAGTAGAGTTCACAGGgtt
```

### Precursor sequence and structure. Mature sequence in capital letters

```
gtcgtatctgtgacatctactgtctcttgaactattgtgttcaaGGGACAGTAGATGTCACAGGttcgat   
 ((((.(((((((((((((((((((((((((((......))))))))))))))))))))))))))).))))
```

---

## locus\_id: 103783

family\_id: 456

### **Targets:**

At1g21740(NM\_102023.1
): expressed protein

Location in genome: in an IGR, 6088 upstream of At1g76880, 1080 downstream of At1g76870

1 homologs in brassica

### Alignment between mature miRNA and predicted targets

```
        Extended mature miRNA:       catgcctctcccttcccatgtgattt
        Target(rc):NM_102023.1       acaGCCACTTCCTTCCCATGTGAaga
```

### Precursor sequence and structure. Mature sequence in capital letters

```
gtaaaaatcacatgggaagatgaaagtggcgtagataactcatcccacatGCCTCTCCCTTCCCATGTGAttttctat   
 ...((((((((((((((((.....((.(((((.(((.....)))....))))).))..))))))))))))))))....
```

---

## locus\_id: 25304

family\_id: 455

### **Targets:**

At5g18750(NM\_121880.2
): DNAJ heat shock N-terminal domain-containing protein

Location in genome: in an IGR, 479 upstream of At1g36990, 35922 downstream of At1g37037

### Alignment between mature miRNA and predicted targets

```
        Extended mature miRNA:       gcatgtcgctggattcagcgaccaaa
        Target(rc):NM_121880.2       agcTGTCACTGGATTCAACGACCttc
```

### Precursor sequence and structure. Mature sequence in capital letters

```
caTGTCGCTGGATTCAGCGACCaaaagcagcgactaatcgctaaaaattttaacgacaaaaaattacggtcgctggtccctggcgactgtcgctacttttgatcgctgaaccagcgacaag   
 ..(((((((((.((((((((.((((((.((((((...(((.(((.....))).)))...........((((((..(...)..)))))))))))).)))))).)))))))))))))))))..
```

---

## locus\_id: 45664

family\_id: 454

### **Targets:**

At4g15410(NM\_117629.2
): UBX domain-containing protein

Location in genome: in an IGR, 466 upstream of At1g69090, 3144 downstream of At1g69120

### Alignment between mature miRNA and predicted targets

```
        Extended mature miRNA:       tgtgtggcccggtattgtttggtttag
        Target(rc):NM_117629.2       gtgGTGGCCCGGTAGGGTTTGGTTcgg
```

### Precursor sequence and structure. Mature sequence in capital letters

```
aaatgtGTGGCCCGGTATTGTTTGGTTtagtctgagtaaatcgaagtctaaactgagaccatatgacttgtatttcatggttatatgattaaatttggtaaaaccaaactaccgggtcatacattt   
 (((((((((((((((((..(((((((((...(..(((........((((......))))(((((((((((.....)).))))))))).....)))..)..))))))))))))))))))))))))))
```

---

## locus\_id: 24650

family\_id: 453

### **Targets:**

At2g47820(NM\_130349.2
): expressed protein

Location in genome: in an IGR, 5422 upstream of At1g36340, 5648 downstream of At1g36370

### Alignment between mature miRNA and predicted targets

```
        Extended mature miRNA:       ctgctgaaggagtaggctccatctttc
        Target(rc):NM_130349.2       catCTGAATGAATAGGCTCCATCTgag
```

### Precursor sequence and structure. Mature sequence in capital letters

```
gtgttctgCTGAAGGAGTAGGCTCCATCTttctcttcttagaggaagattgattttcttcttctgcaccac   
 (((...(((.(((((((.(((.((.((((((((((....)))))))))).)).)))))))))).))).)))
```

---

## locus\_id: 14323

family\_id: 452

### **Targets:**

At1g74850(NM\_106143.2
): pentatricopeptide (PPR) repeat-containing protein

Location in genome: in an IGR, 748 upstream of At1g23350, 174 downstream of At1g23360

### Alignment between mature miRNA and predicted targets

```
        Extended mature miRNA:       attgtggaatgtttcagtgcttggtga
        Target(rc):NM_106143.2       tgaGTGGAATGTTTCAATACTTGGatt
```

### Precursor sequence and structure. Mature sequence in capital letters

```
tggctgttcaccaggctctcaatcacttccacaatccgcatatcccgtcaccggtttaggcgacctaatgctcaatgcctccatgttcgtctcggattGTGGAATGTTTCAGTGCTTGGtgaagtcca   
 .((((..(((((((((.((.((.((.((((((((((((......(((....)))...((((((.(....((.....))......).)))))))))))))))))))).)).)).)))))))))))))..
```

---

## locus\_id: 2946

family\_id: 451

### **Targets:**

At1g15470(NM\_101416.2
): transducin family protein / WD-40 repeat family protein

Location in genome: Contained by At1g05780 (NM\_100458: . hypothetical protein) in an intron on the reverse strand

### Alignment between mature miRNA and predicted targets

```
        Extended mature miRNA:       gaaacccaatctctacttcagtctttc
        Target(rc):NM_101416.2       tacACCCAATCTCTTCTCCAGTCTgga
```

### Precursor sequence and structure. Mature sequence in capital letters

```
ggtatcggaaACCCAATCTCTACTTCAGTCTttcccttttggaaagagagagactgaagaagaggttgagttttcgaaacc   
 (((.((((((((.((((((((.((((((((((((.(((.....))).)))))))))))).)))))))).)))))))).)))
```

---

## locus\_id: 52750

family\_id: 450

### **Targets:**

At5g13130(NM\_121316.1
): hypothetical protein

Location in genome: Contained by At1g80460 (NM\_106694: . glycerol kinase, putative) in an intron

### Alignment between mature miRNA and predicted targets

```
        Extended mature miRNA:       ttgctgagggatttggtttctgatga
        Target(rc):NM_121316.1       aacCTGAGGGATTTGGTTTCTTCcag
```

### Precursor sequence and structure. Mature sequence in capital letters

```
caggattgCTGAGGGATTTGGTTTCTGAtgaaagctttctttggtgatccttaattacaggagactagagaaagaactctcgggaggaagatcccattttgtggagtcttg   
 (((((((.(.(((((((((..(((((((.((...(((((((((((..((((.......)))).)))))))))))...)))))))))..))))))).))....).)))))))
```

---

## locus\_id: 69393

family\_id: 449

### **Targets:**

At3g60860(NM\_115950.2
): guanine nucleotide exchange family protein

Location in genome: in an IGR, 5738 upstream of At1g26920, 795 downstream of At1g26910

### Alignment between mature miRNA and predicted targets

```
        Extended mature miRNA:       ggagggaacagaggattgagaccaat
        Target(rc):NM_115950.2       accGGGAACAGAGGATTCAGACTcgg
```

### Precursor sequence and structure. Mature sequence in capital letters

```
ttggaGGGAACAGAGGATTGAGACCaataccaccagaaaagggtattttacctttggatcatttgcatcaatgtgacacggtattgtttcatccttccttctctccaa   
 .(((((((((..((((((.(((((.((((((....((.(((((((...)))))))...))..(..((....))..)...)))))))))))))))))..))))))))).
```

---

## locus\_id: 1612

family\_id: 448

### **Targets:**

At3g62190(NM\_116084.2
): DNAJ heat shock N-terminal domain-containing protein

Location in genome: in an IGR, 904 upstream of At1g03730, 4388 downstream of At1g03743

### Alignment between mature miRNA and predicted targets

```
        Extended mature miRNA:       tgcggtgttgccaatttgccatcgcg
        Target(rc):NM_116084.2       cttAGTGTTGCCAACTTGCCATCatc
```

### Precursor sequence and structure. Mature sequence in capital letters

```
cggaccatgcGGTGTTGCCAATTTGCCATCgcgaccgtgttgtatggcaaaattggactccgtttccg   
 ((((....((((.((..((((((((((((.(((((...)))))))))).))))))))).)))).))))
```

---

## locus\_id: 97832

family\_id: 447

### **Targets:**

At5g46540(NM\_124024.1
): ABC transporter family protein

Location in genome: in an IGR, 28612 upstream of At1g67530, 309 downstream of At1g67480

### Alignment between mature miRNA and predicted targets

```
        Extended mature miRNA:       ataacaccgttttgcacaaccgcaat
        Target(rc):NM_124024.1       atcACACCGTTCTTCACAACCGCgat
```

### Precursor sequence and structure. Mature sequence in capital letters

```
gcaataACACCGTTTTGCACAACCGCaatggtcctgagccgatgagtctataatttcattagaggctcatcggctcagcactatcacggttttgcaaaacagcgttattgc   
 ((((((((.(.((((((((.(((((..(((((.((((((((((((((((.((((...)))).)))))))))))))))).)))))..))))).)))))))).).))))))))
```

---

## locus\_id: 94976

family\_id: 446

### **Targets:**

At3g62570(NM\_116122.2
): DNAJ heat shock N-terminal domain-containing protein

Location in genome: in an IGR, 5662 upstream of At1g64050, 7633 downstream of At1g64000

### Alignment between mature miRNA and predicted targets

```
        Extended mature miRNA:       aagagcaacgtcgttttgcttcttca
        Target(rc):NM_116122.2       tgaAGCAACGTCGTTTTGCTCCTcac
```

### Precursor sequence and structure. Mature sequence in capital letters

```
gatgcaggagagaagacgacgttagagtttttgtctgcggcagagattacttcccccgcaaggaaaagAGCAACGTCGTTTTGCTTCTtcatc   
 ((((.(((((..(((((((((((.(..(((((.(.(((((..(((.....)))..))))).).)))))..)))))))))))).))))).))))
```

---

## locus\_id: 29984

family\_id: 445

### **Targets:**

At5g52310(NM\_124610.2
): low-temperature-responsive protein 78 (LTI78) / desiccation-responsive protein 29A (RD29A)

Location in genome: in an IGR, 30703 upstream of At1g47540, 21228 downstream of At1g47620

### Alignment between mature miRNA and predicted targets

```
        Extended mature miRNA:       aatgttgtcagcttctccgcgacacct
        Target(rc):NM_124610.2       tcaGTTGTCAGTTTCTCCGCCACAtaa
```

### Precursor sequence and structure. Mature sequence in capital letters

```
tctaatGTTGTCAGCTTCTCCGCGACAcctgtagcgaacattttcgttgtgttggcgtttcggtttatggatgacgacattgga   
 (((((((((((((.((...(((.(((.((..(((((((....)))))))....)).))).))).....)).)))))))))))))
```

---

## locus\_id: 75249

family\_id: 444

### **Targets:**

At4g19380(NM\_118058.3
): alcohol oxidase-related

Location in genome: in an IGR, 14179 upstream of At1g34050, 3116 downstream of At1g34040

### Alignment between mature miRNA and predicted targets

```
        Extended mature miRNA:       agttcggatcgaggattcctcggaccc
        Target(rc):NM_118058.3       tgaTCGGATGGAGCATTCCTCGGAata
```

### Precursor sequence and structure. Mature sequence in capital letters

```
gtcggaatcgatgattcctcggacccgatagatttttctcgaagatttaaagtTCGGATCGAGGATTCCTCGGAcccgat   
 (((((..((((.((.(((((((..(((((((((((((...)))))))))....)))).))))))).)).))))..)))))
```

---

## locus\_id: 113

family\_id: 443

### **Targets:**

At3g25660(NM\_113465.2
): glutamyl-tRNA(Gln) amidotransferase, putative

Location in genome: in an IGR, 1310 upstream of At1g01100, 4758 downstream of At1g01120

### Alignment between mature miRNA and predicted targets

```
        Extended mature miRNA:       tgctggtggagagataccggaagact
        Target(rc):NM_113465.2       ttgTGGTGGAGAGATAACGGAAGtgt
```

### Precursor sequence and structure. Mature sequence in capital letters

```
gaggtgcTGGTGGAGAGATACCGGAAGacttcaccggagattcttgagtcgccggttttctaaaccaccgtctcttc   
 (((..(.((((..(((((.(((((..((((.((..((.....)))))))).))))))))))..)))).)..)))...
```

---

## locus\_id: 26386

family\_id: 442

### **Targets:**

At2g20190(NM\_179666.2
): CLIP-associating protein (CLASP) -related

Location in genome: in an IGR, 8531 upstream of At1g40390, 78787 downstream of At1g41750

### Alignment between mature miRNA and predicted targets

```
        Extended mature miRNA:       gcggtgctgcatgggcgaccttgggc
        Target(rc):NM_179666.2       acaGTGCTGCTTGTGCGACCTTGtgg
```

### Precursor sequence and structure. Mature sequence in capital letters

```
cgtccgagacacctgtcgcctgtgggcgcagcagctgtctggacatttggcagcgggcgGTGCTGCATGGGCGACCTTGggcg   
 ((((((((......(((((((((((((((.((.((((((.........))))))..)).))))).))))))))))))))))))
```

---

## locus\_id: 71480

family\_id: 441

### **Targets:**

At5g58640(NM\_125251.2
): selenoprotein-related

Location in genome: in an IGR, 2265 upstream of At1g29850, 349 downstream of At1g29830

### Alignment between mature miRNA and predicted targets

```
        Extended mature miRNA:       cgcataagccaagtagaagccatgttt
        Target(rc):NM_125251.2       ccgATAAGCCAAGTGGAAGCCATGgat
```

### Precursor sequence and structure. Mature sequence in capital letters

```
gtaatcgcATAAGCCAAGTAGAAGCCATGtttgtcatcatataggacttcttaacttagatgacaaacaaggactactacttgacttatgcgattat   
 (((((((((((((.(((((((.((((.(((((((((((...((((....)))).....))))))))))).)).)).))))))).)))))))))))))
```

---

## locus\_id: 99469

family\_id: 440

### **Targets:**

At3g11850(NM\_112018.3
): expressed protein

Location in genome: in an IGR, 3916 upstream of At1g69680, 9545 downstream of At1g69620

### Alignment between mature miRNA and predicted targets

```
        Extended mature miRNA:       acccccaactccaagattcccaaaaaga
        Target(rc):NM_112018.3       attCCCAATTCCAAGATTCCCTAAAtca
```

### Precursor sequence and structure. Mature sequence in capital letters

```
tctctttttttggtaaatatatatagaagaatcttggagttgggggtattcaagaaaaaacattaataccCCCAACTCCAAGATTCCCAAAAagaaga   
 (((.(((((((((...............((((((((((((((((((((((..............))))))))))))))))))))))))))))))))))
```

---

## locus\_id: 36771

family\_id: 439

### **Targets:**

At5g62370(NM\_125631.1
): pentatricopeptide (PPR) repeat-containing protein

Location in genome: in an IGR, 12507 upstream of At1g56590, 6339 downstream of At1g56630

### Alignment between mature miRNA and predicted targets

```
        Extended mature miRNA:       taaatttgtgacaagagcaagtccaaa
        Target(rc):NM_125631.1       cgcAGTTGTGACAACAGCAAGTCCcac
```

### Precursor sequence and structure. Mature sequence in capital letters

```
ttttagataaATTTGTGACAAGAGCAAGTCCaaatgaaaatttattgatttgctcttgtcacaaatttatctaaag   
 .(((((((((((((((((((((((((((((..(((((....)))))))))))))))))))))))))))))))))).
```

---

## locus\_id: 10851

family\_id: 438

### **Targets:**

At5g02280(NM\_120306.1
): synbindin, putative

Location in genome: in an IGR, 2430 upstream of At1g18070, 6426 downstream of At1g18100

### Alignment between mature miRNA and predicted targets

```
        Extended mature miRNA:       taagtgaaagctcctgagatatggcatc
        Target(rc):NM_120306.1       cagGTGAAAGCTGCTGAGAAATGGCgtg
```

### Precursor sequence and structure. Mature sequence in capital letters

```
ggggtaaGTGAAAGCTCCTGAGATATGGCatccataaaaaaaaccttatcatcaattatattgaatttgataaggcttatgggatccatagcttagcagctttacttcaccct   
 ((((((((((((.(((.(((((.(((((..(((((((......(((((((((((((...)))))...)))))))).)))))))..))))).))))).)))))))))).)))))
```

---

## locus\_id: 96816

family\_id: 437

### **Targets:**

At1g66320(NM\_105303.2
): F-box family protein

Location in genome: in an IGR, 12905 upstream of At1g66340, 25719 downstream of At1g66250

### Alignment between mature miRNA and predicted targets

```
        Extended mature miRNA:       aaaattcaggagaacatgatcgtttggtacga
        Target(rc):NM_105303.2       atgATTCAGGAAAAGATGATCGTTTGGTAaga
```

### Precursor sequence and structure. Mature sequence in capital letters

```
ttgtcggaaaATTCAGGAGAACATGATCGTTTGGTAcgaatacaagatctggtgagaatggacgaagaaggaaagagagacgtgttcgtaccaaacgatcatgttctcctgaatcttccgacaa   
 .((((((((.(((((((((((((((((((((((((((((((((....(((....))).....(......)...........))))))))))))))))))))))))))))))))).)))))))).
```

---

## locus\_id: 63677

family\_id: 436

### **Targets:**

At1g07380(NM\_100612.1
): ceramidase family protein

Location in genome: in an IGR, 12004 upstream of At1g17950, 3976 downstream of At1g17910

1 homologs in brassica

### Alignment between mature miRNA and predicted targets

```
        Extended mature miRNA:       tggcgtttgacagaaagcagaagagagag
        Target(rc):NM_100612.1       gttCGTTTGACAGAAAGCAGAAACGAagc
```

### Precursor sequence and structure. Mature sequence in capital letters

```
aatctctctctcttcttacctgtctgccttgagaaattgcgtaactgatactgatgggtttttaatggCGTTTGACAGAAAGCAGAAGAGAgagaggtt   
 ((((((((((((((((...((((((((((((((((....((((.....))).).....))))))).))))...))))).....))))))))))))))))
```

---

## locus\_id: 4963

family\_id: 435

### **Targets:**

At1g54940(NM\_104367.1
): glycogenin glucosyltransferase (glycogenin)-related

Location in genome: in an IGR, 2400 upstream of At1g09020, 1377 downstream of At1g09030

### Alignment between mature miRNA and predicted targets

```
        Extended mature miRNA:       cagcgatgcctcgggaaccatgagcg
        Target(rc):NM_104367.1       tgcCGATGGCTCGAGAACCATGAttc
```

### Precursor sequence and structure. Mature sequence in capital letters

```
ttggctcaaaccatggaatcgcggcattgagctcctgcacaggcaggcagCGATGCCTCGGGAACCATGAgcgcga   
 ...(((((.....(((..(((.((((((..((((((((....))))).))))))))).)))...))))))))....
```

---

## locus\_id: 91580

family\_id: 434

### **Targets:**

At3g19890(NM\_112879.1
): F-box family protein

Location in genome: in an IGR, 5555 upstream of At1g60090, 8216 downstream of At1g60060

### Alignment between mature miRNA and predicted targets

```
        Extended mature miRNA:       tattggttacccatatggccatctcaaa
        Target(rc):NM_112879.1       tagTGGTTACCCATATGGCCATCTCgcc
```

### Precursor sequence and structure. Mature sequence in capital letters

```
tttcagatggctgtttgggtaactaatatttaagattttggtcaatttaagttaccaatttatatatTGGTTACCCATATGGCCATCTCaaa   
 ....((((((((((.((((((((((((((.((((...(((((.(((....)))))))))))).)))))))))))))).))))))))))....
```

---

## locus\_id: 78524

family\_id: 433

### **Targets:**

At1g47625(NM\_202257.1
): hypothetical protein

Location in genome: in an IGR, 5050 upstream of At1g37000, 4026 downstream of At1g36980

### Alignment between mature miRNA and predicted targets

```
        Extended mature miRNA:       ttagtcgctgcttttggtcgctgaatcc
        Target(rc):NM_202257.1       actGTCGCTACTTTTGATCGCTGAAcca
```

### Precursor sequence and structure. Mature sequence in capital letters

```
gtcgctggttcagcgatcaaaagtagcgacagtcgccagggaccagcgaccgtaattttttgtcgttaaaatttttagcgattaGTCGCTGCTTTTGGTCGCTGAAtccagcgac   
 ((((((((((((((((((((((((((((((((((((.(((((..((((((...........))))))....))))).)))))).)))))))))))))))))))))).))))))))
```

---

## locus\_id: 23688

family\_id: 432

### **Targets:**

At1g62380(NM\_104918.3
): 1-aminocyclopropane-1-carboxylate oxidase, putative / ACC oxidase, putative

Location in genome: in an IGR, 10232 upstream of At1g35610, 3225 downstream of At1g35625

### Alignment between mature miRNA and predicted targets

```
        Extended mature miRNA:       cactggctccttctgctgaaacttgtac
        Target(rc):NM_104918.3       ccgTGGCTCCTTGGGCTGAAACTTGacc
```

### Precursor sequence and structure. Mature sequence in capital letters

```
ccatcgtaaagttcttaggaggagttggcgtaggtggaaaaatcttcgccacTGGCTCCTTCTGCTGAAACTTGtacggtgg   
 ((((((((((((((.(((((((((((((((.((((......)))).)))))...))))))))))..).))))).))))))))
```

---

## locus\_id: 104245

family\_id: 431

### **Targets:**

At5g09820(NM\_121019.2
): plastid-lipid associated protein PAP / fibrillin family protein

Location in genome: in an IGR, 1563 upstream of At1g77400, 9438 downstream of At1g77370

### Alignment between mature miRNA and predicted targets

```
        Extended mature miRNA:       agagagatcttgaaggagacgacgagaa
        Target(rc):NM_121019.2       gacGAGATCTTGAAGGAAGCGACGAttc
```

### Precursor sequence and structure. Mature sequence in capital letters

```
ggcgttctcgttttccggtgagatttagggtttctcttctgcgagagtgagagagaGAGATCTTGAAGGAGACGACGAgaaac   
 ....((((((((((((..(((((((.....((((((((..((....))..)))))))))))))))..))))..))))))))..
```

---

## locus\_id: 83800

family\_id: 430

### **Targets:**

At5g08120(NM\_120894.2
): myosin heavy chain-related

Location in genome: in an IGR, 2927 upstream of At1g47990, 6892 downstream of At1g47970

### Alignment between mature miRNA and predicted targets

```
        Extended mature miRNA:       ctctccccaaacgcagaattgcttagc
        Target(rc):NM_120894.2       tcgTCCCCAAACCCAGAATCGCTTtgc
```

### Precursor sequence and structure. Mature sequence in capital letters

```
ggtgggctagtggttctagtctggtggaagagtggccctgttggtctaggtcagggatcgactcTCCCCAAACGCAGAATTGCTTagcaacatc   
 ((((.(((((..(((((.((.(((.(((.(((((((((((...........))))).)).)))))))))).))..)))))..).))))..))))
```

---

## locus\_id: 80446

family\_id: 429

### **Targets:**

At2g22870(NM\_127852.2
): expressed protein

Location in genome: in an IGR, 28199 upstream of At1g42960, 29983 downstream of At1g42700

### Alignment between mature miRNA and predicted targets

```
        Extended mature miRNA:       gtggaagctcgtctctgcctactccttg
        Target(rc):NM_127852.2       gaaGAAGCTCGTCTCTGCCTAAACCaga
```

### Precursor sequence and structure. Mature sequence in capital letters

```
ggggctaaagttgaggcgggagggttttgctcgttctgttcggtcgagtagttggtcgagcgtgtgGAAGCTCGTCTCTGCCTACTCCttggctct   
 ((((((((.(...(((((((((((((((.(.((...((((((..(((....)))..)))))))).)))))))).)).))))))...).))))))))
```

---

## locus\_id: 93662

family\_id: 428

### **Targets:**

At1g47990(NM\_103695.1
): gibberellin 2-oxidase, putative / GA2-oxidase, putative

Location in genome: in an IGR, 17926 upstream of At1g62440, 4 downstream of At1g62410

### Alignment between mature miRNA and predicted targets

```
        Extended mature miRNA:       accacacttggagtcctcccacatgt
        Target(rc):NM_103695.1       tggACACTTGGAGCCCTCCTACAccg
```

### Precursor sequence and structure. Mature sequence in capital letters

```
aaacatgtgggaggactccaagtgtggttatatcctcggtattatctcgatgtgaaccACACTTGGAGTCCTCCCACAtgttt   
 ((((((((((((((((((((((((((((((((((...((......)).)))))).))))))))))))))))))))))))))))
```

---

## locus\_id: 35536

family\_id: 427

### **Targets:**

At5g25100(NM\_122419.2
): endomembrane protein 70, putative

Location in genome: in an IGR, 6405 upstream of At1g55110, 2837 downstream of At1g55140

### Alignment between mature miRNA and predicted targets

```
        Extended mature miRNA:       agaggaagtagctgagagcaatggaaa
        Target(rc):NM_122419.2       gctGGAAGTAGCAGAGAACAATGGtga
```

### Precursor sequence and structure. Mature sequence in capital letters

```
tcttcttcgttgctttcttcttcctcggacctggactattctctcagatagaGGAAGTAGCTGAGAGCAATGGaaaga   
 ((((.((((((((((((..(((((((....(((((......)).)))...))))))).....))))))))))))))))
```

---

## locus\_id: 92815

family\_id: 426

### **Targets:**

At1g80650(NM\_106713.2
): double-stranded RNA-binding domain (DsRBD)-containing protein

Location in genome: in an IGR, 10513 upstream of At1g61520, 10457 downstream of At1g61470

### Alignment between mature miRNA and predicted targets

```
        Extended mature miRNA:       ccagaataggagatgggtcgagaacc
        Target(rc):NM_106713.2       aggGAATAGAAGAAGGGTCGAGAgga
```

### Precursor sequence and structure. Mature sequence in capital letters

```
cccaGAATAGGAGATGGGTCGAGAaccactgaatgagccaacatataggtcgaaggatccatcgactcttctggg   
 (((((((.((..((((((((..((....(((.(((......))).))).))....))))))))..)).)))))))
```

---

## locus\_id: 19792

family\_id: 425

### **Targets:**

At4g36870(NM\_119851.2
): BEL1-like homeobox 2 protein (BLH2)

Location in genome: in an IGR, 7773 upstream of At1g31350, 7723 downstream of At1g31370

2 homologs in rice

### Alignment between mature miRNA and predicted targets

```
        Extended mature miRNA:       gcagcggctgcggtagcggtggcggc
        Target(rc):NM_119851.2       atgGCGGCTGCGGTGGCGGTGGCtga
```

### Precursor sequence and structure. Mature sequence in capital letters

```
ggttgcggcaGCGGCTGCGGTAGCGGTGGCggcaaacactaccgcaggttgttgttcgttttgttgccgcagacgctgccgcagccgctgccgcaacc   
 (((((((((((((((((((((((((...(((((((.((..((.((((....))))..))..)))))))))...)))))))))))))))))))))))))
```

---

## locus\_id: 95401

family\_id: 424

### **Targets:**

At2g45540(NM\_130116.2
): WD-40 repeat family protein / beige-related

Location in genome: in an IGR, 4452 upstream of At1g64590, 5137 downstream of At1g64570

### Alignment between mature miRNA and predicted targets

```
        Extended mature miRNA:       agagcggcagcaacaatggcagcgtg
        Target(rc):NM_130116.2       gctGCGGCAGCAGCAATTGCAGCtgc
```

### Precursor sequence and structure. Mature sequence in capital letters

```
tggaggagaGCGGCAGCAACAATGGCAGCgtgagcgttatttctcgagtgaaattcgatcaggttgccacatgggttgtttccgccttcttca   
 .(((((((.((((.((((((..(((((((.(((.((..(((((......))))).)).))).))))))).....)))))).))))))))))).
```

---

## locus\_id: 80257

family\_id: 423

### **Targets:**

At1g33030(NM\_103036.2
): O-methyltransferase family 2 protein

Location in genome: in an IGR, 24534 upstream of At1g42630, 20791 downstream of At1g42550

### Alignment between mature miRNA and predicted targets

```
        Extended mature miRNA:       catatttctgaaacaaagcaaaagcaaa
        Target(rc):NM_103036.2       tgaATCTCTGAAACAAAGCAAAAGCttt
```

### Precursor sequence and structure. Mature sequence in capital letters

```
tgcattgcatATTTCTGAAACAAAGCAAAAGCaaaaaaaaatgtattatgctcttgtattgtggtcgttgtaatttttattttttttgctttgtttcagaaatatgcaatgca   
 .((((((((((((((((((((((((((((((.(((.((((((.(((.(((..(.........)..))).))))))))).))))))))))))))))))))))))))))))))).
```

---

## locus\_id: 20629

family\_id: 422

### **Targets:**

At5g57550(NM\_125136.3
): xyloglucan:xyloglucosyl transferase / xyloglucan endotransglycosylase / endo-xyloglucan transferase (XTR3)

Location in genome: in an IGR, 3637 upstream of At1g32350, 16762 downstream of At1g32400

1 homologs in brassica

### Alignment between mature miRNA and predicted targets

```
        Extended mature miRNA:       gctcttgtagcgcactggtctgcgatg
        Target(rc):NM_125136.3       cccCTTGTAGCCCACTGGTCCGCGttc
```

### Precursor sequence and structure. Mature sequence in capital letters

```
cgcccggctCTTGTAGCGCACTGGTCTGCGatggtacattaatgtgcaacgtgtgattagttcgaagctcgagctgggcg   
 ((((((((((..((..((.(((((((((((...(((((....)))))..)))).))))))).))..))..))))))))))
```

---

## locus\_id: 27422

family\_id: 421

### **Targets:**

At3g31350(NM\_113998.2
): hypothetical protein

Location in genome: in an IGR, 56106 upstream of At1g42990, 20405 downstream of At1g43080

### Alignment between mature miRNA and predicted targets

```
        Extended mature miRNA:       cctggatcgatcggtcccctcctgggat
        Target(rc):NM_113998.2       ttgGGATCGATCGATCCCATCCTGGcag
```

### Precursor sequence and structure. Mature sequence in capital letters

```
cgatcgatctggccctgggatcgatcgatcccatcctggcgggtctgaaccttcatctggcagacgacctGGATCGATCGGTCCCCTCCTGGgatcgatcg   
 ((((((((((.(....(((((((((((((((..(((((.((((..(((....))))))).)))..))...)))))))))))))))....).))))))))))
```

---

## locus\_id: 53928

family\_id: 420

### **Targets:**

At3g05950(NM\_111469.2
): germin-like protein, putative

Location in genome: in an IGR, 5504 upstream of At1g02350, 2321 downstream of At1g02320

1 homologs in brassica

### Alignment between mature miRNA and predicted targets

```
        Extended mature miRNA:       tcggtggcgcgtgggtgagtgtgcgg
        Target(rc):NM_111469.2       tcaGTGGCTCGTGGGTGCGTGTGtgg
```

### Precursor sequence and structure. Mature sequence in capital letters

```
cgacttcgGTGGCGCGTGGGTGAGTGTGcggtgggtttaaacctcccggcgcgtagtctatacgtgctaaggagacg   
 ...((((..(((((((((...((.((((((.((((.........)))).)))))).))..))))))))).))))...
```

---

## locus\_id: 36077

family\_id: 419

### **Targets:**

At4g31805(NM\_119330.1
): WRKY family transcription factor

Location in genome: in an IGR, 13946 upstream of At1g55880, 7337 downstream of At1g55940

### Alignment between mature miRNA and predicted targets

```
        Extended mature miRNA:       tatctactttcaaaggtgactctcgat
        Target(rc):NM_119330.1       ttcCCACTTCCAAAGGTGACTCTCtgc
```

### Precursor sequence and structure. Mature sequence in capital letters

```
ttataaagagtcacttttgaaatagatacactatcatactcatccggacgttctcatggacaaagatgatagtgtatCTACTTTCAAAGGTGACTCTCgataa   
 .(((..(((((((((((((((((((((((((((((((.....((((((.....)).)))).....))))))))))))))).))))))))))))))))..))).
```

---

## locus\_id: 42713

family\_id: 418

### **Targets:**

At4g27657(NM\_118904.1
): expressed protein

Location in genome: in an IGR, 6326 upstream of At1g65340, 400 downstream of At1g65350

4 homologs in brassica

### Alignment between mature miRNA and predicted targets

```
        Extended mature miRNA:       tgcgaagaaccccggaggtgactgca
        Target(rc):NM_118904.1       tcgGAAGAACCCCGGCGGTGAGTtta
```

### Precursor sequence and structure. Mature sequence in capital letters

```
gttatgggtcataggtcgggcttcgggcgggcagctttatctgatcaagggccggggcacacgggtcctggtactatccaggtgcGAAGAACCCCGGAGGTGACTgc   
 ......((((((...((((((((((.((.(((((......)))....((..(((((((......)))))))..))..))..)).)))))...)))))..))))))..
```

---

## locus\_id: 54116

family\_id: 417

### **Targets:**

At5g45390(NM\_123907.2
): ATP-dependent Clp protease proteolytic subunit (ClpP4)

Location in genome: in an IGR, 1501 upstream of At1g02690, 509 downstream of At1g02680

### Alignment between mature miRNA and predicted targets

```
        Extended mature miRNA:       tcatcatcatcaggaatctcgggatat
        Target(rc):NM_123907.2       atcTCATCATCAGGTATCTCGGGAgtc
```

### Precursor sequence and structure. Mature sequence in capital letters

```
ttcatcttcaTCATCATCAGGAATCTCGGGAtatggatcagccatgtccttatatgatgatgatattgatgaggaagaagatgaa   
 .((((((((.((.(((((((..((((((((((((((.....))))))))).....)).)))....))))))).)).)))))))).
```

---

## locus\_id: 63601

family\_id: 416

### **Targets:**

At3g14810(NM\_112342.1
): mechanosensitive ion channel domain-containing protein / MS ion channel domain-containing protein

Location in genome: in an IGR, 411 upstream of At1g17745, 215 downstream of At1g17730

### Alignment between mature miRNA and predicted targets

```
        Extended mature miRNA:       ctccctctctcccatgtcttgttcattgca
        Target(rc):NM_112342.1       ataCCTCTCTCCCATGTCTTGATGATTcat
```

### Precursor sequence and structure. Mature sequence in capital letters

```
ggattgttgcagttgtcaacttgggagagaggatgatatggtgaagtctcctcCCTCTCTCCCATGTCTTGTTCATTgcaaagtct   
 (((((.(((((((...((((.(((((((((((..((...((.(....).))))))))))))))).))..))...))))))))))))
```

---

## locus\_id: 3783

family\_id: 415

### **Targets:**

At1g15570(NM\_101426.3
): cyclin, putative

Location in genome: in an IGR, 421 upstream of At1g07050, 1427 downstream of At1g07060

### Alignment between mature miRNA and predicted targets

```
        Extended mature miRNA:       gaggagtgaaaggccgagatacagatc
        Target(rc):NM_101426.3       aacGAGTGAATGGCCGAGACACAGcat
```

### Precursor sequence and structure. Mature sequence in capital letters

```
gaagaagagGAGTGAAAGGCCGAGATACAGatcgaaacatagatatcacttaccaattgataattgggtgacgcctctgtttcagtttagatttcggcttcccattcctcttcatc   
 ...(((((((((((..((((((((((..((((.((((((.((...((((...(((((.....)))))))))...)).)))))).))))..))))))))))..)))))))))))...
```

---

## locus\_id: 94969

family\_id: 414

### **Targets:**

At4g25890(NM\_118722.2
): 60S acidic ribosomal protein P3 (RPP3A)

Location in genome: in an IGR, 5830 upstream of At1g64050, 7465 downstream of At1g64000

### Alignment between mature miRNA and predicted targets

```
        Extended mature miRNA:       gttgagctcgtcggtggaagaagacc
        Target(rc):NM_118722.2       ctgGAGCTCGTAGGTAGAAGAAGctg
```

### Precursor sequence and structure. Mature sequence in capital letters

```
cctgttttgcttccgtcgacgaggacgacggaagctatttcgcggaagatgtcgttGAGCTCGTCGGTGGAAGAAGacctaagg   
 ((((((((.(((((..((((((((((((((...((......))......)))))))...)))))))..))))))))))...)))
```

---

## locus\_id: 91475

family\_id: 413

### **Targets:**

At2g48110(NM\_130378.1
): expressed protein

Location in genome: in an IGR, 54727 upstream of At1g60060, 786 downstream of At1g59980

### Alignment between mature miRNA and predicted targets

```
        Extended mature miRNA:       tgtatagagcgagaaaggcatcataac
        Target(rc):NM_130378.1       accATAGAGCTAGAAAGGCATCATgcc
```

### Precursor sequence and structure. Mature sequence in capital letters

```
tcgaacatgtATAGAGCGAGAAAGGCATCATaactaaggttccaaagactctaaaaaacgttagagagttaagatgcctatcttgttctatttttgtgcga   
 (((.(((...(((((((((((.(((((((.(((((..(....).....((((((......))))))))))).))))))).)))))))))))...))).)))
```

---

## locus\_id: 84497

family\_id: 412

### **Targets:**

At1g48910(NM\_103784.1
): flavin-containing monooxygenase family protein / FMO family protein

Location in genome: in an IGR, 3264 upstream of At1g48920, 1051 downstream of At1g48910

### Alignment between mature miRNA and predicted targets

```
        Extended mature miRNA:       tgctttggcatcggtgctttgggaat
        Target(rc):NM_103784.1       tgtTTTGGCATCGGTGCTTTGGGgaa
```

### Precursor sequence and structure. Mature sequence in capital letters

```
tttccaatgcTTTGGCATCGGTGCTTTGGGaatccatcctatttttgtgatattatacaggactatgaatacgtggtgaagaagaatggattccccaaagcaccgatgccagatcattggaaa   
 .((((((((.(((((((((((((((((((((((((((.((....(((((......))))).((((((....)))))).....)).))))))).)))))))))))))))))))).)))))))).
```

---

## locus\_id: 86839

family\_id: 411

### **Targets:**

At1g32440(NM\_102979.1
): pyruvate kinase, putative

Location in genome: in an IGR, 4580 upstream of At1g52170, 100 downstream of At1g52155

### Alignment between mature miRNA and predicted targets

```
        Extended mature miRNA:       ttgtgttcttgaattagtcaatctatgtt
        Target(rc):NM_102979.1       actTGTTCTTGAATCAGCCAATCTATatg
```

### Precursor sequence and structure. Mature sequence in capital letters

```
ttttagtttgTGTTCTTGAATTAGTCAATCTATgttattgaaaggattactacgttgtggtatttaggattttggttagttaaggattggccaaatcaagaacacaaactagag   
 .((((((((((((((((((.((.((((((((.((..((..(((.((.((((((...)))))).))....)))..))....)).)))))))).)).)))))))))))))))))).
```

---

## locus\_id: 88660

family\_id: 410

### **Targets:**

At1g17070(NM\_101567.2
): D111/G-patch domain-containing protein

Location in genome: in an IGR, 1944 upstream of At1g55050, 1706 downstream of At1g55040

### Alignment between mature miRNA and predicted targets

```
        Extended mature miRNA:       cctttgacttctcttctccacgtctg
        Target(rc):NM_101567.2       ctcTTGACCTCTCTTCTCCACCTttg
```

### Precursor sequence and structure. Mature sequence in capital letters

```
ttacttatgtggggaagatatgtcacaggaacaggaccggtcgatccatgcttcatcgtcaatttctgtaaatttcataagaccgatcggtcatgttcctTTGACTTCTCTTCTCCACGTctgtaa   
 .(((..((((((((((((...((((.(((((((.(((((((((.((.(((.........((.....)).......)))..)).))))))))).))))))).))))...))))))))))))..))).
```

---

## locus\_id: 75065

family\_id: 409

### **Targets:**

At1g04930(NM\_100371.2
): hydroxyproline-rich glycoprotein family protein

Location in genome: in an IGR, 1770 upstream of At1g33860, 22689 downstream of At1g33811

### Alignment between mature miRNA and predicted targets

```
        Extended mature miRNA:       ggtcgacgaatcctcgaactggacggcca
        Target(rc):NM_100371.2       ctgCGACGAATCCTCGAACAGGTCGGgct
```

### Precursor sequence and structure. Mature sequence in capital letters

```
ggccgagtggtcgaggaattctcgatccggacagccgtatggtCGACGAATCCTCGAACTGGACGGcc   
 (((((..((((((((((.((((((((((((....)))...)))))).))))))))).))))..)))))
```

---

## locus\_id: 38895

family\_id: 408

### **Targets:**

At4g24020(NM\_118534.3
): RWP-RK domain-containing protein

Location in genome: in an IGR, 3121 upstream of At1g60780, 4738 downstream of At1g60790

### Alignment between mature miRNA and predicted targets

```
        Extended mature miRNA:       gcagaggaagagaggctaatcggcttc
        Target(rc):NM_118534.3       aggGAGGAAGAGAGGATACTCGGCgag
```

### Precursor sequence and structure. Mature sequence in capital letters

```
ggagtcgttggacagagatcttcgtcttccgtgttggatacggcaGAGGAAGAGAGGCTAATCGGCttc   
 (((((((((((.(.....(((((.(((.((((((...)))))).))).)))))...))))).)))))))
```

---

## locus\_id: 70987

family\_id: 407

### **Targets:**

At1g04250(NM\_100306.2
): auxin-responsive protein / indoleacetic acid-induced protein 17 (IAA17)

Location in genome: in an IGR, 3203 upstream of At1g29270, 892 downstream of At1g29260

2 homologs in brassica

### Alignment between mature miRNA and predicted targets

```
        Extended mature miRNA:       ctctattggcaggaaaccattactta
        Target(rc):NM_100306.2       gatTTTTGGCAGGAAACCATCACgtt
```

### Precursor sequence and structure. Mature sequence in capital letters

```
agatctcTATTGGCAGGAAACCATTACttagatctttgcatctctttatgcattgcttttaattagtgagttatctgccaaaggagattt   
 ((((((((.((((((((.((((((((.(((((.(..(((((......)))))..)..))))).))))).))).)))))))).))))))))
```

---

## locus\_id: 25400

family\_id: 406

### **Targets:**

At2g11090(NM\_126833.1
): expressed protein

Location in genome: in an IGR, 28578 upstream of At1g36990, 7823 downstream of At1g37037

### Alignment between mature miRNA and predicted targets

```
        Extended mature miRNA:       ataggtcgagtgaaggtgatgaatgt
        Target(rc):NM_126833.1       agtGATCGAGTGAAGGTGATGAAggt
```

### Precursor sequence and structure. Mature sequence in capital letters

```
tcgagtgaatgatgatggagttactcggcctgttggtagagtgattggtcgagtgaatgatgatggtgttactcggccatgttggtagagtgataGGTCGAGTGAAGGTGATGAAtgtactcgg   
 .((((((..(.((.((....(((((((((((((((.(..(.(((((((((((((((............))))))))))).)))).)..).)))))))))))))))..)).)).)...)))))).
```

---

## locus\_id: 1108

family\_id: 405

### **Targets:**

At5g09350(NM\_120971.2
): phosphatidylinositol 4-kinase, putative

Location in genome: in an IGR, 1192 upstream of At1g02710, 3382 downstream of At1g02730

### Alignment between mature miRNA and predicted targets

```
        Extended mature miRNA:       gtttgctgattagagagagcaacatta
        Target(rc):NM_120971.2       tgcTGCTGATTAGAGAGAGCACCAaag
```

### Precursor sequence and structure. Mature sequence in capital letters

```
tgttTGCTGATTAGAGAGAGCAACAttatgattaaacaaatcttagcgttaaaagtaagaacttttaagctctttttaaatcacgataaattaggctctctctaatcagcaaaca   
 .((((((((((((((((((((...((..(((((....(((....(((.((((((((....)))))))))))...))).)))))..)).......)))))))))))))))))))).
```

---

## locus\_id: 309223

family\_id: 404

### **Targets:**

At5g19290(NM\_121934.2
): esterase/lipase/thioesterase family protein

Location in genome: in an IGR, 6862 upstream of At4g09750, 121 downstream of At4g09730

### Alignment between mature miRNA and predicted targets

```
        Extended mature miRNA:       aggggtggttgacggctacggcactc
        Target(rc):NM_121934.2       gacGGTGTTTGACGGCTACGGAAgtc
```

### Precursor sequence and structure. Mature sequence in capital letters

```
atcagaggGGTGGTTGACGGCTACGGCActcttagagtttctggaggttgaggattgtctatgccgctctatttgat   
 ((((((((((((((.(((((((.((((.((((.((.....)))))))))).)).)))))...)))))))).))))))
```

---

## locus\_id: 304628

family\_id: 403

### **Targets:**

At4g28250(NM\_118965.2
): beta-expansin, putative (EXPB3)

Location in genome: in an IGR, 116982 upstream of At4g06603, 204547 downstream of At4g06536

### Alignment between mature miRNA and predicted targets

```
        Extended mature miRNA:       caggggccgaagttgaggcgggaggg
        Target(rc):NM_118965.2       acaGGGGAGAAGTTGAGGCGGGAact
```

### Precursor sequence and structure. Mature sequence in capital letters

```
tgcagGGGCCGAAGTTGAGGCGGGAgggtgttgctcgttctgtccggtcgagcgtgtggaagctcgtctctgccttctccttggctctctca   
 .(..((((((((.(..(((((((((((((.(..(.(((((.........))))).)..)..)))).)).)))))))..).))))))))..).
```

---

## locus\_id: 399917

family\_id: 402

### **Targets:**

At2g41740(NM\_129738.3
): villin 2 (VLN2)

Location in genome: in an IGR, 31837 upstream of At5g34870, 11418 downstream of At5g34861

### Alignment between mature miRNA and predicted targets

```
        Extended mature miRNA:       agggttcaatgatgttgccactgtgcc
        Target(rc):NM_129738.3       agaGTTCAAAGATGTTGCCACCGTttc
```

### Precursor sequence and structure. Mature sequence in capital letters

```
tggtggcagctggtgacagaatggcaaaccccagccagtttgaggGTTCAATGATGTTGCCACTGTgccca   
 .((..((((.(((..(((..((....((((((((.....))).)))))..))..)))..)))))))..)).
```

---

## locus\_id: 388068

family\_id: 401

### **Targets:**

At2g15690(NM\_127130.2
): pentatricopeptide (PPR) repeat-containing protein

Location in genome: in an IGR, 7821 upstream of At5g18500, 972 downstream of At5g18470

### Alignment between mature miRNA and predicted targets

```
        Extended mature miRNA:       gtgagatagaggattctggtggtggg
        Target(rc):NM_127130.2       tccAGATTGAGGATTCTGGTGGTaat
```

### Precursor sequence and structure. Mature sequence in capital letters

```
atcaagcacaccctgaggatctctctctcgtttcatcaaactgacagcagattcgtgagagataaacttggttttggggtttgtttccacaggtgAGATAGAGGATTCTGGTGGTgggcttggt   
 (((((((.((((.(.((((((.((((...((((((((...(((....)))....(((.(((((((((((.......)))))))))))))).)))))))))))))))))).).)))).)))))))
```

---

## locus\_id: 348615

family\_id: 400

### **Targets:**

At4g22580(NM\_118384.3
): exostosin family protein

Location in genome: in an IGR, 7095 upstream of At5g27960, 15908 downstream of At5g27980

### Alignment between mature miRNA and predicted targets

```
        Extended mature miRNA:       aaatgggtggatgaagcgacgtcgttt
        Target(rc):NM_118384.3       agcTGTGTGGATGAAACGACGTCGgat
```

### Precursor sequence and structure. Mature sequence in capital letters

```
tcaaaTGGGTGGATGAAGCGACGTCGttttccttatgtttcgaagagttgaaaaaatgagaaaacgatgtcgtttctcaatcgggtcatgggtga   
 (((.((((.(.((((((((((((((((((((.((((.((((((....))))))..)))))))))))))))))))))...))).).))))...)))
```

---

## locus\_id: 385986

family\_id: 399

### **Targets:**

At5g15980(NM\_121603.3
): pentatricopeptide (PPR) repeat-containing protein

Location in genome: Contained by At5g15070 (NM\_121511: . expressed protein) in an intron

### Alignment between mature miRNA and predicted targets

```
        Extended mature miRNA:       ctgtcattgtgtcttgcagagttctat
        Target(rc):NM_121603.3       gctTCACTGTGTCTTGCAGACTTCatc
```

### Precursor sequence and structure. Mature sequence in capital letters

```
ggcgagagaagtttgcattgcatttaggcaagcggtatgtggttattccagttatctgactgTCATTGTGTCTTGCAGAGTTCtatcgcc   
 (((((.((((.((((((..((((...((((..(((((..(((.....)))..)).)))..))))...))))..)))))).)))).)))))
```

---

## locus\_id: 305548

family\_id: 398

### **Targets:**

At5g02580(NM\_120336.2
): expressed protein

Location in genome: in an IGR, 12866 upstream of At4g06676, 200806 downstream of At4g06603

### Alignment between mature miRNA and predicted targets

```
        Extended mature miRNA:       gaggtggaagtgttgataggctggga
        Target(rc):NM_120336.2       cctGTGGAAGTGATGATAGGCTTaat
```

### Precursor sequence and structure. Mature sequence in capital letters

```
cgagGTGGAAGTGTTGATAGGCTGggaagggctgccagcatcagaggatacttgggaaccggcaacaccattttcagctcagtaacccaatttccaccttg   
 ((((((((((((((((...(((((..(((((.((((.(..((.(((....))).))..).)))).).)).))..)))))...))))...))))))))))))
```

---

## locus\_id: 305876

family\_id: 397

### **Targets:**

At4g17280(NM\_117834.3
): auxin-responsive family protein

Location in genome: in an IGR, 77885 upstream of At4g07390, 8287 downstream of At4g07310

### Alignment between mature miRNA and predicted targets

```
        Extended mature miRNA:       cgtcgctggcacggtgggtgcttcgg
        Target(rc):NM_117834.3       tccCGATGGCACGGTGGGTGCTGtac
```

### Precursor sequence and structure. Mature sequence in capital letters

```
gaaccccggagtctgaaccatcatcgtccgcgatgaccactgtctccgagttcgtggatggaggaagttctggcggggtggctaccgcttccggaatcgtCGCTGGCACGGTGGGTGCTTcgggttc   
 ((((((.(((((....(((..(((((((((((((((...(((((..((....))..))))).((((((..((((......))))..))))))....))))))).)).))))))))))))))))))))
```

---

## locus\_id: 328860

family\_id: 396

### **Targets:**

At1g64720(NM\_105147.2
): expressed protein

Location in genome: Contained by At4g36170 (NM\_119784: . hypothetical protein) in an intron

### Alignment between mature miRNA and predicted targets

```
        Extended mature miRNA:       cggcggaactcatcatccctaagtgg
        Target(rc):NM_105147.2       gaaCGGAACTCATCATCCCAAAAgaa
```

### Precursor sequence and structure. Mature sequence in capital letters

```
tccacgtaggacggtggtgggtcggccggcggtcactaatcaagcggCGGAACTCATCATCCCTAAGtggg   
 .((((.((((..(((((((((((.((((..(((.....)))...)))).).)))))))))))))).)))).
```

---

## locus\_id: 369981

family\_id: 395

### **Targets:**

At5g58900(NM\_125280.2
): myb family transcription factor

Location in genome: in an IGR, 2714 upstream of At5g55893, 11221 downstream of At5g55910

### Alignment between mature miRNA and predicted targets

```
        Extended mature miRNA:       ccaagtccgtttggttcaaaggagct
        Target(rc):NM_125280.2       tgtTGTCCGTTTGGTTCCAAGGAaac
```

### Precursor sequence and structure. Mature sequence in capital letters

```
gttgggccaAGTCCGTTTGGTTCAAAGGAgctgtccccaagcatgcttttaacttttggactgcccagc   
 (((((((..((((((...((((.((((.((((.......))).).)))).))))..)))))))))))))
```

---

## locus\_id: 308717

family\_id: 394

### **Targets:**

At2g22940(NM\_127859.1
): expressed protein

Location in genome: in an IGR, 935 upstream of At4g09310, 978 downstream of At4g09300

### Alignment between mature miRNA and predicted targets

```
        Extended mature miRNA:       ccgagccgatatagatgtgtcccgcagcg
        Target(rc):NM_127859.1       caaAGCCGATATATATGTTTCCCGCAccg
```

### Precursor sequence and structure. Mature sequence in capital letters

```
tgagaggtggcttcggggcaaacggtatgtaccggctcggggcgcgcttgatgactgtccattaagctcagttggctgtctcccgAGCCGATATAGATGTGTCCCGCAgcgttctca   
 ...((((..(((.(((((((.......((((.((((((((((...((((((((......)))))))).(((....)))..)))))))))).))))....))))))).)))..)))).
```

---

## locus\_id: 331206

family\_id: 393

### **Targets:**

At2g46650(NM\_130230.1
): cytochrome b5, putative

Location in genome: in an IGR, 4298 upstream of At4g39920, 3848 downstream of At4g39890

### Alignment between mature miRNA and predicted targets

```
        Extended mature miRNA:       ctgactgatacggcccattaagaaca
        Target(rc):NM_130230.1       cctACTGATACGGCCCATTAACAtaa
```

### Precursor sequence and structure. Mature sequence in capital letters

```
cccaatttttcttaatgggtcgtatcagactttatatttattccatcatactgACTGATACGGCCCATTAAGAacaattggg   
 (((((((.((((((((((((((((((((.(..(((............)))..).)))))))))))))))))))).)))))))
```

---

## locus\_id: 355970

family\_id: 392

### **Targets:**

At2g26890(NM\_128246.3
): DNAJ heat shock N-terminal domain-containing protein

Location in genome: in an IGR, 1959 upstream of At5g36890, 13749 downstream of At5g36910

### Alignment between mature miRNA and predicted targets

```
        Extended mature miRNA:       gcggctgcttggggtgacggaggaga
        Target(rc):NM_128246.3       gctGCTGCTTTGGGTGAAGGAGGtgg
```

### Precursor sequence and structure. Mature sequence in capital letters

```
gctcgcttccctgtccaagcaaccctagcggtgctccgtcggaattccctctctaggcgGCTGCTTGGGGTGACGGAGGagagt   
 ((((.(((((..(((......((((((((((....((((((((........))).))))))))).)))))))))))))).))))
```

---

## locus\_id: 292287

family\_id: 391

### **Targets:**

At1g59950(NM\_104687.1
): aldo/keto reductase, putative

Location in genome: in an IGR, 343 upstream of At4g30970, 4292 downstream of At4g30980

### Alignment between mature miRNA and predicted targets

```
        Extended mature miRNA:       tcttctgtcagttttctctgttgcatt
        Target(rc):NM_104687.1       aacTCCCTCAGTTTTCTCTGTTGCcat
```

### Precursor sequence and structure. Mature sequence in capital letters

```
gaggtcaagcaggcagagataggcaactgacagaaagagaggtgagcacgcacacgcaaagttatagacacataacagtgcctgcatgtgctcactctctTCTGTCAGTTTTCTCTGTTGCattcctc   
 ((((....((((.((((((.....(((((((((((.(((((.((((((((..((.(((..(((((......)))))..))).))..)))))))))))))))))))))))).))))))))))...))))
```

---

## locus\_id: 278105

family\_id: 390

### **Targets:**

At1g05820(NM\_100463.1
): protease-associated (PA) domain-containing protein

Location in genome: in an IGR, 8523 upstream of At4g11920, 1351 downstream of At4g11950

### Alignment between mature miRNA and predicted targets

```
        Extended mature miRNA:       tgaatcatgttgtaaccacccaataac
        Target(rc):NM_100463.1       ccgATCATGTTGTAACCACCCCATgga
```

### Precursor sequence and structure. Mature sequence in capital letters

```
atgggttaatggattgttacggtatgattcgggtgacaccaccctaactggtttcccctaaaaccggtttgggtagtgacccttgaATCATGTTGTAACCACCCAATaacccat   
 ((((((((.(((...(((((..(((((((((((.(.(((.((((.(((((((((......))))))))).)))).))).).)))))))))))..)))))...))).))))))))
```

---

## locus\_id: 322713

family\_id: 389

### **Targets:**

At1g22870(NM\_102133.2
): protein kinase family protein

Location in genome: in an IGR, 3449 upstream of At4g26700, 3769 downstream of At4g26680

### Alignment between mature miRNA and predicted targets

```
        Extended mature miRNA:       ggaggagccgagattttggtcgggtcg
        Target(rc):NM_102133.2       cttGGAGCAGAAATTTTGGTCGGGagg
```

### Precursor sequence and structure. Mature sequence in capital letters

```
gtggaggaGGAGCCGAGATTTTGGTCGGGtcggcttcagctgatgttgttgttgacctctttgttcctcggactcttaatttcac   
 ((((((.((((((((((....(((..((((((((..((((....))))..))))))))..)))...))))).)))))..))))))
```

---

## locus\_id: 373738

family\_id: 388

### **Targets:**

At3g50180(NM\_114878.1
): hypothetical protein

Location in genome: in an IGR, 7194 upstream of At5g62140, 10393 downstream of At5g62170

### Alignment between mature miRNA and predicted targets

```
        Extended mature miRNA:       agtgacagggcaactctcctttggcaa
        Target(rc):NM_114878.1       caaGACAGTGCAACTCTCCATTGGata
```

### Precursor sequence and structure. Mature sequence in capital letters

```
cggaagcagtGACAGGGCAACTCTCCTTTGGcaagtgacatagccaatagtcaagaacacaaaagatacaaatagccaagtcgcctgccaatagaaagatgccctattactgctccg   
 ((((.(((((((.((((((.((.((..((((((.(((((...((.....(((............))).......))...))))).))))))..)).)).)))))).)))))))))))
```

---

## locus\_id: 395402

family\_id: 387

### **Targets:**

At4g16630(NM\_117764.3
): DEAD/DEAH box helicase, putative (RH28)

Location in genome: in an IGR, 14947 upstream of At5g28320, 24737 downstream of At5g28280

### Alignment between mature miRNA and predicted targets

```
        Extended mature miRNA:       cggttgtttgccttctctagggaacc
        Target(rc):NM_117764.3       gcaTTGTTTGCCTTCTCTTGGGAcat
```

### Precursor sequence and structure. Mature sequence in capital letters

```
gtgcctaattgaaggaagcaccgtccattaccggtcataggcttgtgaccggtgggaagcgcctcatggaaccggtcacaacccacatgatcggTTGTTTGCCTTCTCTAGGGAac   
 ...((((...((((((((((.........(((((((((.((.(((((((((((........((....)).))))))))))).))..)))))))))))))).)))))..))))....
```

---

## locus\_id: 394369

family\_id: 386

### **Targets:**

At3g04310(NM\_111302.1
): expressed protein

Location in genome: in an IGR, 1854 upstream of At5g27820, 1795 downstream of At5g27810

### Alignment between mature miRNA and predicted targets

```
        Extended mature miRNA:       ttaatctcatcctccatagcgagacc
        Target(rc):NM_111302.1       atcATCCCATCCTCCATAGCCAGtac
```

### Precursor sequence and structure. Mature sequence in capital letters

```
attcactttaATCTCATCCTCCATAGCGAGaccctactacacggccatgtattgttttttgaaactctatccatgatcgtgtagtagagtctcattagggggatgtgattgaagtggat   
 (((((((((((((.((((((((.(((.(((((.((((((((((..((((.((.((((....))))...)).))))..)))))))))).))))).))))))))))).)))))))))))))
```

---

## locus\_id: 271838

family\_id: 385

### **Targets:**

At5g35604(NM\_148034.1
): hypothetical protein

Location in genome: in an IGR, 40234 upstream of At4g06599, 56553 downstream of At4g06634

### Alignment between mature miRNA and predicted targets

```
        Extended mature miRNA:       tcgagcctttgatttgtccctccgagt
        Target(rc):NM_148034.1       cctAGCCCTTGCTTTGTCCCTCCGcgg
```

### Precursor sequence and structure. Mature sequence in capital letters

```
tcggggtggaggagatgatcgaggggagctattagttcgAGCCTTTGATTTGTCCCTCCGagtaga   
 ((((((.(((.((....((((((((((((.....))))...)))))))))).))))))))).....
```

---

## locus\_id: 340003

family\_id: 384

### **Targets:**

At4g36640(NM\_119827.2
): SEC14 cytosolic factor family protein / phosphoglyceride transfer family protein

Location in genome: in an IGR, 5327 upstream of At5g15520, 4157 downstream of At5g15540

### Alignment between mature miRNA and predicted targets

```
        Extended mature miRNA:       tgcctagctcttgaagctcttcctgttaa
        Target(rc):NM_119827.2       aaaCTAGCTCTTGAAGCTTTTCCGGTctc
```

### Precursor sequence and structure. Mature sequence in capital letters

```
tcctctctgcCTAGCTCTTGAAGCTCTTCCTGTtaacgaaggcacatcattgtgcaatgcttgatgggtcgaggctcgagcaaggcgagaaaagga   
 (((((((.((((.((((..((..(((..((((((((((...(((((....)))))..)).))))))))..)))..)))))).))))))))...)))
```

---

## locus\_id: 311059

family\_id: 383

### **Targets:**

At4g38070(NM\_119968.2
): bHLH family protein

Location in genome: in an IGR, 8436 upstream of At4g11970, 3012 downstream of At4g11940

### Alignment between mature miRNA and predicted targets

```
        Extended mature miRNA:       tcacccgaatcataccgtaacaatccatt
        Target(rc):NM_119968.2       tcgCCCGAATCATGCCGTAATAATCCttt
```

### Precursor sequence and structure. Mature sequence in capital letters

```
tgggttattgggtggttacaacatgattcaagggtcactacccaaaccggttttaggggaaaccagttagggtggtgtcaCCCGAATCATACCGTAACAATCCattaaccca   
 .((((((.(((((.(((((...(((((((..((((((((((((.(((.((((((....)))))).))).))))))))..)))))))))))...))))).))))).)))))).
```

---

## locus\_id: 418674

family\_id: 382

### **Targets:**

At3g12020(NM\_112036.2
): kinesin motor protein-related

Location in genome: in an IGR, 1655 upstream of At5g60410, 2195 downstream of At5g60390

2 homologs in brassica

### Alignment between mature miRNA and predicted targets

```
        Extended mature miRNA:       cggtatctctcctacgtagcaatcctt
        Target(rc):NM_112036.2       atcTGTCTCTCCAACGTAGCAATCtgg
```

### Precursor sequence and structure. Mature sequence in capital letters

```
aagatttgcttcgcaggagagatagcgccatcacctcttctaagaagttaactagtggtgacggTATCTCTCCTACGTAGCAATCctt   
 (((..(((((.((.((((((((((.((.((((((..((((...)))).......)))))).)).)))))))))).)).)))))..)))
```

---

## locus\_id: 379073

family\_id: 381

### **Targets:**

At1g26250(NM\_102389.1
): proline-rich extensin, putative

Location in genome: Contained by At5g03560 (NM\_120437: . expressed protein) in an intron on the reverse strand

### Alignment between mature miRNA and predicted targets

```
        Extended mature miRNA:       agggtcatacggaggaggtggaggctc
        Target(rc):NM_102389.1       ataGACATAAGGAGGAGGTGGAGGaga
```

### Precursor sequence and structure. Mature sequence in capital letters

```
taggatcctctggttcctcaatcgccggtttcttgctgaaaggGTCATACGGAGGAGGTGGAGGctcctg   
 (((((.((((((.((((((.((.(((..((((.....)))).))).))...)))))).)))))).)))))
```

---

## locus\_id: 385708

family\_id: 380

### **Targets:**

At5g26340(NM\_122535.2
): hexose transporter, putative

Location in genome: in an IGR, 2832 upstream of At5g14560, 17142 downstream of At5g14495

2 homologs in brassica

### Alignment between mature miRNA and predicted targets

```
        Extended mature miRNA:       gttctcaggtcacccctgctgagctc
        Target(rc):NM_122535.2       ctgCTCCGGTCACCACTGCTGAGtaa
```

### Precursor sequence and structure. Mature sequence in capital letters

```
gtagtggatctcgacagggttgatatgagaacacacgagtaatcaacggctgtaatgacgctacgtcattgttacagctctcgttttcatgtgttCTCAGGTCACCCCTGCTGAGctctttctc   
 ..((.(((.((((.(((((.((((.((((((((((.(((.(((....(((((((((((((...))))))...)))))))...)))))).)))))))))).)))).))))).)))).)))..)).
```

---

## locus\_id: 418672

family\_id: 379

### **Targets:**

At1g34270(NM\_103149.3
): exostosin family protein

Location in genome: in an IGR, 1717 upstream of At5g60410, 2134 downstream of At5g60390

### Alignment between mature miRNA and predicted targets

```
        Extended mature miRNA:       aagatttgcttcgcaggagagatagc
        Target(rc):NM_103149.3       cgaTTTTGCTTCGCAGGAAAGATctc
```

### Precursor sequence and structure. Mature sequence in capital letters

```
gcaaataaagATTTGCTTCGCAGGAGAGATagcgccatcacctcttctaagaagttaactagtggtgacggtatctctcctacgtagcaatccttatatatgc   
 (((.((((((..(((((.((.((((((((((.((.((((((..((((...)))).......)))))).)).)))))))))).)).)))))..))).))).)))
```

---

## locus\_id: 336448

family\_id: 378

### **Targets:**

At5g59970(NM\_125390.2
): histone H4

Location in genome: in an IGR, 27988 upstream of At5g09380, 3293 downstream of At5g09460

### Alignment between mature miRNA and predicted targets

```
        Extended mature miRNA:       ccgtaaccgccggatccgtagatataa
        Target(rc):NM_125390.2       gatTAACCGCCGAATCCGTAGAGAgtc
```

### Precursor sequence and structure. Mature sequence in capital letters

```
agtatagtgtctagtaggcctgttacggatccggatatccgggtttttttggagtatccggatccggtccgTAACCGCCGGATCCGTAGATAtaatatt   
 (((((.(((((((((.(((..((((((((.(((((..((((((((((....))).)))))))))))))))))))).)))..))...))))))).)))))
```

---

## locus\_id: 281389

family\_id: 377

### **Targets:**

At5g56230(NM\_125006.2
): prenylated rab acceptor (PRA1) family protein

Location in genome: in an IGR, 22801 upstream of At4g15640, 6362 downstream of At4g15730

### Alignment between mature miRNA and predicted targets

```
        Extended mature miRNA:       ggccgctgaagacgtggagacgattga
        Target(rc):NM_125006.2       gaaCGCTGCACACGTGGAGACGATgat
```

### Precursor sequence and structure. Mature sequence in capital letters

```
ggcCGCTGAAGACGTGGAGACGATtgatttcgtcctgaaaagtgacgagcgtgtctagtcgtctgcatgtctttgtttcc   
 ((..((.(((((((((.(((((((((((((((((((....)).))))))...))).)))))))).)))))))))))..))
```

---

## locus\_id: 343356

family\_id: 376

### **Targets:**

At5g08630(NM\_120950.2
): DDT domain-containing protein

Location in genome: in an IGR, 3426 upstream of At5g20630, 19 downstream of At5g20635

### Alignment between mature miRNA and predicted targets

```
        Extended mature miRNA:       catgctgcagctgcaactgcaagatc
        Target(rc):NM_120950.2       atgGCTGCAACTGCAACTACAAGctt
```

### Precursor sequence and structure. Mature sequence in capital letters

```
ccagctgtttccgcagttgcagttgcactcgaccgtcgtgtctgaataaaaagaagagctcatGCTGCAGCTGCAACTGCAAGatcagatgg   
 (((.(((.((..((((((((((((((((..((.(.((...(((........))).))).))..).)))))))))))))))..)).))).)))
```

---

## locus\_id: 267182

family\_id: 375

### **Targets:**

At1g23780(NM\_102226.2
): F-box family protein

Location in genome: in an IGR, 4363 upstream of At4g02710, 727 downstream of At4g02715

### Alignment between mature miRNA and predicted targets

```
        Extended mature miRNA:       agtgatcttcctcttcgcctcgcttc
        Target(rc):NM_102226.2       tacGATCTTCTTCTTCGCCTCACaaa
```

### Precursor sequence and structure. Mature sequence in capital letters

```
agtGATCTTCCTCTTCGCCTCGCttcctgtttttttgaaatggaaaaagggaagaggaagttttact   
 (((((.((((((((((.(((...((((.(((((...)))))))))..)))))))))))))..)))))
```

---

## locus\_id: 396303

family\_id: 374

### **Targets:**

At3g02660(NM\_111134.3
): tRNA synthetase class I (W and Y) family protein

Location in genome: in an IGR, 33029 upstream of At5g28690, 6058 downstream of At5g28660

### Alignment between mature miRNA and predicted targets

```
        Extended mature miRNA:       agtaacaacccgtcccgtgggacccgc
        Target(rc):NM_111134.3       gtcACCAACCCGTCCCGTGGCACCacc
```

### Precursor sequence and structure. Mature sequence in capital letters

```
ggcagagtAACAACCCGTCCCGTGGGACCcgcccacgtggccacactctgccacgtggcatcctgttggcgggtcccacgggacaggttgttacagaatgtc   
 ((((..(((((((((.(((((((((((((((((((((((((........))))))))..........))))))))))))))))).)))))))))....))))
```

---

## locus\_id: 352823

family\_id: 373

### **Targets:**

At5g41140(NM\_123479.2
): expressed protein

Location in genome: in an IGR, 37023 upstream of At5g33420, 4197 downstream of At5g33431

### Alignment between mature miRNA and predicted targets

```
        Extended mature miRNA:       taccagagcactccttggatggctttcg
        Target(rc):NM_123479.2       actCAGAGCACTCCTTGTATTGCTTctt
```

### Precursor sequence and structure. Mature sequence in capital letters

```
tacCAGAGCACTCCTTGGATGGCTTtcgccacaagagcactccctggatggctttcgccaccaaagcactccctggacggctgtcgaccaacggactgcgcttggtg   
 ((((((.(((.(((((((.((((..((((((...(((..((...(((.((((....))))))).))..)))..))).)))..)))).)))).))).)))..))))))
```

---

## locus\_id: 363785

family\_id: 372

### **Targets:**

At4g30080(NM\_119154.3
): transcriptional factor B3 family protein

Location in genome: in an IGR, 18416 upstream of At5g46800, 2838 downstream of At5g46860

### Alignment between mature miRNA and predicted targets

```
        Extended mature miRNA:       tgtcgttatgcctggctccctgtatgc
        Target(rc):NM_119154.3       gagCATTATGCCTGGCTCCCTGTAaac
```

### Precursor sequence and structure. Mature sequence in capital letters

```
gtttgtCGTTATGCCTGGCTCCCTGTAtgccacgagtggataccgattttggttttaaaatcggctgccggtggcgtacaaggagtcaagcatgaccagaagc   
 ((((.(.(((((((.(((((((.((((((((((..(..(...(((((((((....))))))))))..)..)))))))))).))))))).))))))).).))))
```

---

## locus\_id: 276204

family\_id: 371

### **Targets:**

At1g17745(NM\_101636.2
): D-3-phosphoglycerate dehydrogenase / 3-PGDH

Location in genome: in an IGR, 22229 upstream of At4g09670, 11344 downstream of At4g09740

### Alignment between mature miRNA and predicted targets

```
        Extended mature miRNA:       accctgtccgatagaccacccaaggc
        Target(rc):NM_101636.2       gagCTGACCGATAGACCACCCTAatg
```

### Precursor sequence and structure. Mature sequence in capital letters

```
gggaggtcctatgggagtttgcggagaggacggtgatagccacgcttcagtccaaacattgatgctatcaccCTGTCCGATAGACCACCCAAggccttttc   
 ((((((.(((.((((.(((((((((.(((...((((((((......(((((......))))).))))))))))).)))).)))))..))))))))))))).
```

---

## locus\_id: 292289

family\_id: 370

### **Targets:**

At1g15230(NM\_101391.2
): expressed protein

Location in genome: in an IGR, 416 upstream of At4g30970, 4219 downstream of At4g30980

1 homologs in brassica

### Alignment between mature miRNA and predicted targets

```
        Extended mature miRNA:       aactgacagaaagagaggtgagcacgc
        Target(rc):NM_101391.2       gttTGACAGAAGGAGAGCTGAGCAgtc
```

### Precursor sequence and structure. Mature sequence in capital letters

```
gataggcaacTGACAGAAAGAGAGGTGAGCAcgcacacgcaaagttatagacacataacagtgcctgcatgtgctcactctcttctgtcagttttctctgtt   
 ((((((.(((((((((((.(((((.((((((((..((.(((..(((((......)))))..))).))..))))))))))))))))))))))))...))))))
```

---

## locus\_id: 415511

family\_id: 369

### **Targets:**

At3g57880(NM\_115650.2
): C2 domain-containing protein

Location in genome: in an IGR, 14927 upstream of At5g55050, 525 downstream of At5g55010

### Alignment between mature miRNA and predicted targets

```
        Extended mature miRNA:       tggtctccagaaagttttcttccaagg
        Target(rc):NM_115650.2       ttaTCTCCAGAGAGTTTTCCTCCAcct
```

### Precursor sequence and structure. Mature sequence in capital letters

```
ttggTCTCCAGAAAGTTTTCTTCCAaggttcaagaacggatcttcacaattcaaattggtgagaaacagtttgaattttgaggatccgttcttgaaccttgcaagaaaactttctgaagaccaa   
 .((((((.((((((((((((((.(((((((((((((((((((((((.(((((((((((........))))))))))).))))))))))))))))))))))).)))))))))))))).)))))).
```

---

## locus\_id: 282348

family\_id: 368

### **Targets:**

At5g10650(NM\_121102.3
): zinc finger (C3HC4-type RING finger) family protein

Location in genome: in an IGR, 484 upstream of At4g16790, 1059 downstream of At4g16800

### Alignment between mature miRNA and predicted targets

```
        Extended mature miRNA:       ttggcgccgtctgtgggaattcaaaa
        Target(rc):NM_121102.3       tctGCGCCGTCTGTGAGAATTAAcaa
```

### Precursor sequence and structure. Mature sequence in capital letters

```
ttgGCGCCGTCTGTGGGAATTCAaaaaactttgctaattcaaaaagctcaaagcttaaagattttttgattacccacagagagcgtcaa   
 .((((((..((((((((...(((((((((((((...........(((.....)))))))).))))))))...))))))))..)))))).
```

---

## locus\_id: 272000

family\_id: 367

### **Targets:**

At3g43470(NM\_114215.1
): hypothetical protein

Location in genome: in an IGR, 62015 upstream of At4g06599, 34773 downstream of At4g06634

3 homologs in brassica

### Alignment between mature miRNA and predicted targets

```
        Extended mature miRNA:       cccaatgttgagcatttggtggtgtt
        Target(rc):NM_114215.1       gaaATTGTTGAGCATTCGGTGGTtct
```

### Precursor sequence and structure. Mature sequence in capital letters

```
gtatcccccAATGTTGAGCATTTGGTGGTgttcgccaatgttgtgcatttggtggtgttccccaatgttgaacatttggtggtgtgc   
 (((..((((((((((.((((((.((.((...(((((((((.....)).)))))))....)))))))))).)))).)))).))..)))
```

---

## locus\_id: 287090

family\_id: 366

### **Targets:**

At2g22730(NM\_127838.2
): transporter-related

Location in genome: Contained by At4g22830 (NM\_118412: . expressed protein) in an intron

### Alignment between mature miRNA and predicted targets

```
        Extended mature miRNA:       attatgagagaagcttcatctacatat
        Target(rc):NM_127838.2       ctaATGAAAGAAGCTTCACCTACAcca
```

### Precursor sequence and structure. Mature sequence in capital letters

```
cattATGAGAGAAGCTTCATCTACAtataaagtctcgatctttcactcacaatgtagattaagcttctctcattatg   
 (((.(((((((((((((.((((((((....(((...((....)))))....)))))))).))))))))))))).)))
```

---

## locus\_id: 382445

family\_id: 365

### **Targets:**

At3g46620(NM\_114529.4
): zinc finger (C3HC4-type RING finger) family protein

Location in genome: in an IGR, 4022 upstream of At5g08750, 19272 downstream of At5g08690

### Alignment between mature miRNA and predicted targets

```
        Extended mature miRNA:       ttcctgtaacaacaatacgacgttgttttg
        Target(rc):NM_114529.4       caaCTGTAACACCAATACGACGTCGTTaga
```

### Precursor sequence and structure. Mature sequence in capital letters

```
acatcgtttcCTGTAACAACAATACGACGTTGTTttgtcgtaacgatgaaactaaattccaaatttcaaagtttcgttgttattaccaaacgacgtcgttttgttgttagagaaaacaatgt   
 ((((.((((.((.(((((((((.((((((((((((.((.((((((((((((((................)))))))))))))).)).)))))))))))).))))))))).)).)))).))))
```

---

## locus\_id: 285594

family\_id: 364

### **Targets:**

At5g04820(NM\_120564.1
): ovate family protein

Location in genome: in an IGR, 2547 upstream of At4g21350, 24050 downstream of At4g21370

### Alignment between mature miRNA and predicted targets

```
        Extended mature miRNA:       atactttttccccattgttaccacatg
        Target(rc):NM_120564.1       cttCTTCTTCCCCATTGATACCACcaa
```

### Precursor sequence and structure. Mature sequence in capital letters

```
tgcataCTTTTTCCCCATTGTTACCACatgctccattgaagaggttcatgataatccatgcaatttttcataggagcatgtggaaattaataacggaaacaaagaatgca   
 .((((.((((((((...(((((((((((((((((..(((((((...((((......))))...)))))))..)))))))))))....)))))).))))..)))).)))).
```

---

## locus\_id: 347042

family\_id: 363

### **Targets:**

At4g22080(NM\_118329.2
): pectate lyase family protein

Location in genome: in an IGR, 4783 upstream of At5g26130, 4055 downstream of At5g26150

### Alignment between mature miRNA and predicted targets

```
        Extended mature miRNA:       ttatgggttatgaccatatctttctt
        Target(rc):NM_118329.2       tctAGGGTTATGACCATATCCTTagc
```

### Precursor sequence and structure. Mature sequence in capital letters

```
gtgatggttaTGGGTTATGACCATATCTTTctttccactttcggcaagaaagcatgtggagaatgcccagcctgtcac   
 (((((((...(((((.....(((((((((((((.((......)).))))))).)))))).....)))))..)))))))
```

---

## locus\_id: 363032

family\_id: 362

### **Targets:**

At1g20960(NM\_101949.1
): U5 small nuclear ribonucleoprotein helicase, putative

Location in genome: in an IGR, 2057 upstream of At5g45650, 14060 downstream of At5g45690

### Alignment between mature miRNA and predicted targets

```
        Extended mature miRNA:       acagtcgccaacgaccagcgaccgca
        Target(rc):NM_101949.1       ggtGTCGCCCACGACCAGCCACCatc
```

### Precursor sequence and structure. Mature sequence in capital letters

```
gcgacaGTCGCCAACGACCAGCGACCgcaatttttggtcgctgaaatttttagcgatcagttactggtttcagcgattagtcgactgtttttggtcgctggatcagcgacatgc   
 ......(((((....((((((((((((((.((.(((((((((((((((..((((.....))))..)))))))))))))))..)).)))....))))))))).)).)))))....
```

---

## locus\_id: 306635

family\_id: 361

### **Targets:**

At4g12330(NM\_117303.2
): cytochrome P450 family protein

Location in genome: in an IGR, 27823 upstream of At4g07868, 18726 downstream of At4g07825

### Alignment between mature miRNA and predicted targets

```
        Extended mature miRNA:       gcggtccggtctgccaaagaccgcag
        Target(rc):NM_117303.2       cgaGCCCGGTCTGCCAAATACCGgac
```

### Precursor sequence and structure. Mature sequence in capital letters

```
cgggcggtctatgacggaccttgcttatgttcattccgaggcgGTCCGGTCTGCCAAAGACCGcagacg   
 ((.(((((((.((.((((((((((((..(......)..))))))...)))))).)).)))))))...))
```

---

## locus\_id: 397267

family\_id: 360

### **Targets:**

At2g06908(NM\_147262.1
): hypothetical protein

Location in genome: in an IGR, 47687 upstream of At5g29591, 47992 downstream of At5g29570

### Alignment between mature miRNA and predicted targets

```
        Extended mature miRNA:       tggagccaaagctgatggggcggggg
        Target(rc):NM_147262.1       tgaAGCCGAAGCTGATGGGGCAGtgg
```

### Precursor sequence and structure. Mature sequence in capital letters

```
ctcttggtgccgtgcggctaaagttactagtcttgaagccgaggtcggctgagtcttggAGCCAAAGCTGATGGGGCGGgggg   
 (((((.((.((((.(((((...(((...((.(((..(((((....)))))))).))...)))...))))))))).)).)))))
```

---

## locus\_id: 413229

family\_id: 359

### **Targets:**

At1g36990(NM\_103354.3
): expressed protein

Location in genome: in an IGR, 3374 upstream of At5g51670, 738 downstream of At5g51660

### Alignment between mature miRNA and predicted targets

```
        Extended mature miRNA:       gaaagaagaaatgattggagtcagaagg
        Target(rc):NM_103354.3       tgtAGAAGAAATAATTGGAATCAGAtag
```

### Precursor sequence and structure. Mature sequence in capital letters

```
gaatgaaAGAAGAAATGATTGGAGTCAGAaggagaatgttggtgatatgcttagcagcgtgactgcgtctgtactgttgcaagtcagcgatttctccttttgcctccttcctttctctttagatc   
 .....(((((.((((.((..((((.(((((((((((.((((.((((.(((...(((((((....))).))))......))).)))).))))))))))))))).)))).)).))))))))).....
```

---

## locus\_id: 420158

family\_id: 358

### **Targets:**

At1g29320(NM\_102672.3
): transducin family protein / WD-40 repeat family protein

Location in genome: in an IGR, 3125 upstream of At5g62960, 9683 downstream of At5g62930

### Alignment between mature miRNA and predicted targets

```
        Extended mature miRNA:       cttcgtttgcaacagctgtctctctg
        Target(rc):NM_102672.3       ctgTGTTTGCTACAGCTGTCTCTtct
```

### Precursor sequence and structure. Mature sequence in capital letters

```
gtgaagagaacagcagttttgttgcaaacgcgtttacttagacgcgtcgctctcgtatcttgcacgctcttttggcttCGTTTGCAACAGCTGTCTCTctgtcac   
 ((((((((((((((.....(((((((((((.(((.....(((.((((.((...........)))))))))...)))..)))))))))))))))).))))).))))
```

---

## locus\_id: 353291

family\_id: 357

### **Targets:**

At2g09388(NM\_179617.1
): hypothetical protein

Location in genome: in an IGR, 5898 upstream of At5g34780, 6837 downstream of At5g34820

### Alignment between mature miRNA and predicted targets

```
        Extended mature miRNA:       tgctcttgcggtccagtgacagatcgtggg
        Target(rc):NM_179617.1       tgtTCTTGCGGTCTGGTGACAGATCGTagg
```

### Precursor sequence and structure. Mature sequence in capital letters

```
gaagccttgatttatgtcatggaccctcatgagacattggagatggagtgagtcctatcaatgcTCTTGCGGTCCAGTGACAGATCGTgggtttc   
 ((((((((((((..(((((((((((..((.(((.((((((....(((.....)))..))))))))).)).)))))).)))))))))).)))))))
```

---

## locus\_id: 353290

family\_id: 356

### **Targets:**

At2g09388(NM\_179617.1
): hypothetical protein

Location in genome: in an IGR, 5891 upstream of At5g34780, 6848 downstream of At5g34820

### Alignment between mature miRNA and predicted targets

```
        Extended mature miRNA:       tccagtgacagatcgtgggtttcgcc
        Target(rc):NM_179617.1       tctGGTGACAGATCGTAGGTTTCccc
```

### Precursor sequence and structure. Mature sequence in capital letters

```
cctaaggaagccttgatttatgtcatggaccctcatgagacattggagatggagtgagtcctatcaatgctcttgcggtccAGTGACAGATCGTGGGTTTCgccgg   
 ......((((((((((((..(((((((((((..((.(((.((((((....(((.....)))..))))))))).)).)))))).)))))))))).))))))).....
```

---

## locus\_id: 290119

family\_id: 355

### **Targets:**

At1g04600(NM\_100339.1
): myosin, putative

Location in genome: in an IGR, 2347 upstream of At4g27585, 492 downstream of At4g27595

### Alignment between mature miRNA and predicted targets

```
        Extended mature miRNA:       attcgctgattctgcaacggcgatgg
        Target(rc):NM_100339.1       gtaCGCAGAATCTGCAACGGCGAaag
```

### Precursor sequence and structure. Mature sequence in capital letters

```
ttccgttgttgtagctaccgagtcattgtccgattctggattCGCTGATTCTGCAACGGCGAtggga   
 ((((((((((((.((....((((((..(((((....)))))....)))))).)).))))))))))))
```

---

## locus\_id: 271558

family\_id: 354

### **Targets:**

At3g62570(NM\_116122.2
): DNAJ heat shock N-terminal domain-containing protein

Location in genome: in an IGR, 331258 upstream of At4g06526, 13734 downstream of At4g06599

### Alignment between mature miRNA and predicted targets

```
        Extended mature miRNA:       cgaggtgagtcacggcgaggtgatcgc
        Target(rc):NM_116122.2       aagGATGAGTCACGGCGAGGAGATgag
```

### Precursor sequence and structure. Mature sequence in capital letters

```
caggtcagcaattggctcgctgctgagtccagatcgggaagtgggccgaGGTGAGTCACGGCGAGGTGATcgctccttg   
 ((((..(((.((((.(((((((.(((.(((...((((........)))).).)).)))))))))).)))).))).))))
```

---

## locus\_id: 348195

family\_id: 353

### **Targets:**

At1g50480(NM\_103931.2
): formate--tetrahydrofolate ligase / 10-formyltetrahydrofolate synthetase (THFS)

Location in genome: in an IGR, 15979 upstream of At5g27790, 4923 downstream of At5g27840

### Alignment between mature miRNA and predicted targets

```
        Extended mature miRNA:       gggtctcgctatggaggatgagatta
        Target(rc):NM_103931.2       tgaTCTCGCGTTGGAGGATGAGAaga
```

### Precursor sequence and structure. Mature sequence in capital letters

```
cacttcaatcacatccccctaatgagactctactacacgatcatggatagagtttcaaaaaacaatacatggccgtgtagtagggTCTCGCTATGGAGGATGAGAttaaagtg   
 (((((.((((.(((((((.((.(((((((((((((((((..((((......((((....))))....))))..))))))))))))))))).)).)).))))).)))).)))))
```

---

## locus\_id: 413436

family\_id: 352

### **Targets:**

At5g47780(NM\_124152.2
): glycosyl transferase family 8 protein

Location in genome: in an IGR, 9861 upstream of At5g52030, 4846 downstream of At5g51970

### Alignment between mature miRNA and predicted targets

```
        Extended mature miRNA:       ctgtctagccacggtttggggtttcg
        Target(rc):NM_124152.2       atcTCTAGCCATGGTTTGAGGTTgcc
```

### Precursor sequence and structure. Mature sequence in capital letters

```
ggagtgtaaactgctctctgtggttcgaaaggtgagccacgtttgaagggacgggttcgtccgctgTCTAGCCACGGTTTGGGGTTtcgcttt   
 .(((((.(((((.(...((((((((.((..((((((((.((((......)))))))))).))....)).))))))))...).)))))))))).
```

---

## locus\_id: 352831

family\_id: 351

### **Targets:**

At4g07932(NM\_148273.1
): hypothetical protein

Location in genome: in an IGR, 37415 upstream of At5g33420, 3806 downstream of At5g33431

### Alignment between mature miRNA and predicted targets

```
        Extended mature miRNA:       gtggtacatccgcggtatgcctgtcgt
        Target(rc):NM_148273.1       gtaGTACACCCGCAGTATGCCTGTagt
```

### Precursor sequence and structure. Mature sequence in capital letters

```
cgtgGTACATCCGCGGTATGCCTGTcgtacgtccgcggtatgcatgtggtacgtctgcggtatgcatgtggtacgtctgcggtatgcccgtggtacgtctgtggtatgcccgtaaatgaccgtg   
 ...(((.(((..((((((((((...((.(((((((((((((((.((..(.(((((..((.......))..).)))))..))))))).)))))).)))).)).)))))).))))..))))))...
```

---

## locus\_id: 371869

family\_id: 350

### **Targets:**

At3g18550(NM\_112741.1
): TCP family transcription factor, putative

Location in genome: in an IGR, 1180 upstream of At5g58930, 3268 downstream of At5g58950

### Alignment between mature miRNA and predicted targets

```
        Extended mature miRNA:       gggtcgatctcatcgatctcccaata
        Target(rc):NM_112741.1       tctTCCCTCTCATCGATCTCCCAtcg
```

### Precursor sequence and structure. Mature sequence in capital letters

```
gtattgggagatatctgagatcgacccaaaccaaatcgactcggttagggTCGATCTCATCGATCTCCCAatat   
 ((((((((((((...((((((((((((.((((..........)))).))))))))))))...))))))))))))
```

---

## locus\_id: 270567

family\_id: 349

### **Targets:**

At1g23710(NM\_102219.2
): expressed protein

Location in genome: in an IGR, 109623 upstream of At4g05633, 204222 downstream of At4g06526

### Alignment between mature miRNA and predicted targets

```
        Extended mature miRNA:       ttgtggtgaatttcatggagatctcttct
        Target(rc):NM_102219.2       attTGGTGAAATCCATGGAGATCTCTgtt
```

### Precursor sequence and structure. Mature sequence in capital letters

```
gggatttttgTGGTGAATTTCATGGAGATCTCTtctccgtcgtggatcccctctccggcgtggagagatagatgtttggcatggagagatcaagctatcagagacacaccggaggattcct   
 ((((.((((.(((((..((((((((.(((((((((...((((.(.(((..((((((.....))))))...))).).))))..)))))))))...))))..))))..))))).)))).))))
```

---

## locus\_id: 302345

family\_id: 348

### **Targets:**

At5g51690(NM\_124548.3
): 1-aminocyclopropane-1-carboxylate synthase, putative / ACC synthase, putative

Location in genome: in an IGR, 17660 upstream of At4g04423, 17760 downstream of At4g04404

### Alignment between mature miRNA and predicted targets

```
        Extended mature miRNA:       ttgaccatatttgagttatcaaatgacagtg
        Target(rc):NM_124548.3       ataACCATATTTGAGGGATCAAATGACAcat
```

### Precursor sequence and structure. Mature sequence in capital letters

```
ttctaggttgACCATATTTGAGTTATCAAATGACAgtggcaattaggctaatgagaatgtgtcactgtcatttgataatccaaatatggtatacatagaa   
 .((((.((..((((((((((..(((((((((((((((((((......(....)......)))))))))))))))))))..))))))))))..)).)))).
```

---

## locus\_id: 277404

family\_id: 347

### **Targets:**

At3g10040(NM\_111839.2
): expressed protein

Location in genome: in an IGR, 20756 upstream of At4g11090, 7774 downstream of At4g11140

### Alignment between mature miRNA and predicted targets

```
        Extended mature miRNA:       cttactttctttcttgctcctatcat
        Target(rc):NM_111839.2       ctcTCTCTCTTTCTTGCTCCTATatc
```

### Precursor sequence and structure. Mature sequence in capital letters

```
tgagtgatggtcatggcatgaaagaaggtgagaattgcatcgtgaatcaaaacctccacaagttggatatcttgattttctgatcaacgcaattcttACTTTCTTTCTTGCTCCTATcatcactga   
 ..(((((((((...((((.(((((((((((((((((((((((.(((((((....((((.....))))....)))))))..))))....))))))))))))))))))).))))...)))))))))..
```

---

## locus\_id: 344773

family\_id: 346

### **Targets:**

At1g13130(NM\_101183.3
): glycosyl hydrolase family 5 protein / cellulase family protein

Location in genome: in an IGR, 14174 upstream of At5g23240, 4673 downstream of At5g23300

### Alignment between mature miRNA and predicted targets

```
        Extended mature miRNA:       ggagggagctggtggagacaggaagg
        Target(rc):NM_101183.3       aacGGGAGCTTGTGGAGAGAGGAtaa
```

### Precursor sequence and structure. Mature sequence in capital letters

```
gagaaggaGGGAGCTGGTGGAGACAGGAaggagaagcacatgggtgtggtggggtccttttcacacagctcaccatctc   
 ((((.((...((((((((((((..((((....(..((((....))))..)....)))))))))).)))))).)).))))
```

---

## locus\_id: 352263

family\_id: 345

### **Targets:**

At1g06270(NM\_100509.3
): pentatricopeptide (PPR) repeat-containing protein

Location in genome: in an IGR, 4290 upstream of At5g33240, 67345 downstream of At5g33300

### Alignment between mature miRNA and predicted targets

```
        Extended mature miRNA:       aaaaaaaaaaatgttccccagttcgggatc
        Target(rc):NM_100509.3       aacAACAAAAATGTTCCCAAGTTCGGGttg
```

### Precursor sequence and structure. Mature sequence in capital letters

```
caaagccaaaAAAAAAAATGTTCCCCAGTTCGGGatcgatcgatccaaaactcatccacgaagtaaaatcgtctgctagaacgggaacgatcgatcccgagctggcggacattgttttttttttgctttg   
 ((((((.((((((((((((((((.((((((((((((((((((.(((...........((((.......))))...........))).)))))))))))))))))).))))))).))))))))).))))))
```

---

## locus\_id: 314329

family\_id: 344

### **Targets:**

At2g35070(NM\_129059.2
): expressed protein

Location in genome: in an IGR, 1717 upstream of At4g15660, 524 downstream of At4g15650

### Alignment between mature miRNA and predicted targets

```
        Extended mature miRNA:       tttcgtcggtagtataccatcgagaatcc
        Target(rc):NM_129059.2       taaCGTCGCTAGTATATCATCGAGAAaag
```

### Precursor sequence and structure. Mature sequence in capital letters

```
tcgactaattttcgatggatttctgacggctatattcacgactggttagtatttCGTCGGTAGTATACCATCGAGAAtccgtcga   
 (((((..(((((((((((((..(((((((..(((((.((.....)).))))).)))))))....)).)))))))))))..)))))
```

---

## locus\_id: 281656

family\_id: 343

### **Targets:**

At3g10360(NM\_111871.2
): pumilio/Puf RNA-binding domain-containing protein

Location in genome: in an IGR, 22562 upstream of At4g16000, 118 downstream of At4g16040

### Alignment between mature miRNA and predicted targets

```
        Extended mature miRNA:       acggggccgttaaatccaccggcaccgg
        Target(rc):NM_111871.2       aatGGGCTGTCAAATCCACCGGCACtga
```

### Precursor sequence and structure. Mature sequence in capital letters

```
tgagcaccgacaatggtggacccataatggtcgacggaacggcggtgacgGGGCCGTTAAATCCACCGGCACcggtggtga   
 ..(.(((((....(((((((....((((((((..((..((....))..)).))))))))..)))))))....))))).)..
```

---

## locus\_id: 324326

family\_id: 342

### **Targets:**

At3g50270(NM\_114887.2
): transferase family protein

Location in genome: in an IGR, 4275 upstream of At4g29330, 8239 downstream of At4g29285

### Alignment between mature miRNA and predicted targets

```
        Extended mature miRNA:       aggatcgtcggcgggagagaaggagga
        Target(rc):NM_114887.2       gaaATCGTCGGCGGGAAAGAAGTActt
```

### Precursor sequence and structure. Mature sequence in capital letters

```
cgccgtcaggATCGTCGGCGGGAGAGAAGGAggagcgacgtggcatttcgctggctgtttttttgtcgttttccgtccgatttgttcggcg   
 (((((...((((((..(((((((((((..((((((((.(.((((.....))))).))))))))..)).)))))))))))))))...)))))
```

---

## locus\_id: 410692

family\_id: 341

### **Targets:**

At3g19890(NM\_112879.1
): F-box family protein

Location in genome: in an IGR, 1855 upstream of At5g48290, 5886 downstream of At5g48240

### Alignment between mature miRNA and predicted targets

```
        Extended mature miRNA:       ggtaacgcacaacactaagccatcgcagtt
        Target(rc):NM_112879.1       tggAACGCACAACACTAAACCATCGCAttg
```

### Precursor sequence and structure. Mature sequence in capital letters

```
gtctttcactacgatgacttattgttgtgttaccaaagaaaacataaggcttgtgttgtctttggtAACGCACAACACTAAGCCATCGCAgttacagac   
 ((((.(.(((.(((((.((((.(((((((((((((((((.((((((.....)))))).)))))))))..)))))))).)))).))))).))).).))))
```

---

## locus\_id: 306241

family\_id: 340

### **Targets:**

At1g79220(NM\_106573.3
): mitochondrial transcription termination factor family protein / mTERF family protein

Location in genome: in an IGR, 56368 upstream of At4g07670, 65447 downstream of At4g07523

### Alignment between mature miRNA and predicted targets

```
        Extended mature miRNA:       tctttgctcttcccggtcgagtgttg
        Target(rc):NM_106573.3       aaaTTTCTCTTCCCGGTCGAATGcat
```

### Precursor sequence and structure. Mature sequence in capital letters

```
tcgattgtggcactcgactgcagcggcgtagaggtggtcgagtacggcgagttgtgctcaggtgtctTTGCTCTTCCCGGTCGAGTGttgtggcga   
 (((.(((..(((((((((((..(.((.((((((..(.(.((((((((....)))))))).).)..)))))).)).).)))))))))))..))))))
```

---

## locus\_id: 361406

family\_id: 339

### **Targets:**

At3g51770(NM\_115036.2
): tetratricopeptide repeat (TPR)-containing protein

Location in genome: in an IGR, 4841 upstream of At5g43590, 16857 downstream of At5g43650

2 homologs in brassica

### Alignment between mature miRNA and predicted targets

```
        Extended mature miRNA:       agattgaatggtctcgagccaggcatc
        Target(rc):NM_115036.2       actTTGAATGGTTTCGAGCCAGCCtta
```

### Precursor sequence and structure. Mature sequence in capital letters

```
gggaatgaagcctggtccgacatcatcatcatcatcgttataatcatgagatcatgagaTTGAATGGTCTCGAGCCAGGCatcattcac   
 ..((((((.((((((..(((.(((((((((((.(((.((((....))))))).))))...)).))))).)))..)))))).))))))..
```

---

## locus\_id: 337348

family\_id: 338

### **Targets:**

At2g23350(NM\_127899.2
): polyadenylate-binding protein, putative / PABP, putative

Location in genome: in an IGR, 2460 upstream of At5g10940, 2604 downstream of At5g10950

3 homologs in brassica

### Alignment between mature miRNA and predicted targets

```
        Extended mature miRNA:       tttgcgtgctcactctctttttgtca
        Target(rc):NM_127899.2       gcaGCGTGCTCACTCTCTATCTGgtc
```

### Precursor sequence and structure. Mature sequence in capital letters

```
aagttgacagaagagagtgagcacacaaaggggaagttgtataaaagttttgtatatggttgcttttGCGTGCTCACTCTCTTTTTGtcataactt   
 ((((((((((((((((((((((((.((((((.((...(((((((.....)))))))...)).)))))).)))))))))))).))))))))..))))
```

---

## locus\_id: 390809

family\_id: 337

### **Targets:**

At1g64960(NM\_105170.2
): expressed protein

Location in genome: in an IGR, 1196 upstream of At5g23070, 12978 downstream of At5g23030

### Alignment between mature miRNA and predicted targets

```
        Extended mature miRNA:       accgctgcctccagcgacttcactctt
        Target(rc):NM_105170.2       cttGCAGCCTCCAGCGTCTTCACTtca
```

### Precursor sequence and structure. Mature sequence in capital letters

```
gaagagcaagcagcgctggatgcagaggtttatcgatcgattcttgcttttgtaaacaaaaataaatattcacaggaattgatcgatgaaccGCTGCCTCCAGCGACTTCACTcttt   
 ((((((.(((...(((((((.((((.((((((((((((((((((((..(((((........)))))......)))))))))))))))))))).)))).))))))).)))..))))))
```

---

## locus\_id: 379984

family\_id: 336

### **Targets:**

At1g09940(NM\_100868.2
): glutamyl-tRNA reductase 2 / GluTR (HEMA2)

Location in genome: in an IGR, 62 upstream of At5g04910, 289 downstream of At5g04900

### Alignment between mature miRNA and predicted targets

```
        Extended mature miRNA:       ttcgcaactttctctttgcttcttcg
        Target(rc):NM_100868.2       gctGCAACTTTCTCTTCGCTTCGatt
```

### Precursor sequence and structure. Mature sequence in capital letters

```
gttcGCAACTTTCTCTTTGCTTCTtcgtctctctctctctgtcgtgtttgagaagacgaaaagagtcggagagaaagatcggac   
 (((((...(((((((((((.((((((((((.((((...(......)...))))))))))..)))).)))))))))))..)))))
```

---

## locus\_id: 347360

family\_id: 335

### **Targets:**

At5g54390(NM\_124819.2
): inositol monophosphatase family protein

Location in genome: in an IGR, 8475 upstream of At5g26640, 10027 downstream of At5g26620

### Alignment between mature miRNA and predicted targets

```
        Extended mature miRNA:       ggattaccgctgcttcctcaaccatgta
        Target(rc):NM_124819.2       cttTTACCGCTGCTTCCTCGACTATatt
```

### Precursor sequence and structure. Mature sequence in capital letters

```
tgcaatggttgaggcagcgcggcactatgatctcggctccaaggagtggggaTTACCGCTGCTTCCTCAACCATgta   
 ((((.(((((((((.(((((((.....(((((((.((((....)))).))))))))))).))).)))))))))))))
```

---

## locus\_id: 366054

family\_id: 334

### **Targets:**

At5g32426(NM\_147974.1
):

Location in genome: in an IGR, 2618 upstream of At5g49870, 12935 downstream of At5g49890

### Alignment between mature miRNA and predicted targets

```
        Extended mature miRNA:       cagtccacagatggtccgctgcggtt
        Target(rc):NM_147974.1       tccTTGACAGATGGTCCGCTGCGcca
```

### Precursor sequence and structure. Mature sequence in capital letters

```
aaaccgttgcggaccaagtttatttgtatgtcaaaacagTCCACAGATGGTCCGCTGCGgttt   
 (((((((.((((((((......(((((.(((....)))....))))))))))))).)))))))
```

---

## locus\_id: 369912

family\_id: 333

### **Targets:**

At5g19020(NM\_121907.1
): pentatricopeptide (PPR) repeat-containing protein

Location in genome: in an IGR, 10729 upstream of At5g55810, 15593 downstream of At5g55870

### Alignment between mature miRNA and predicted targets

```
        Extended mature miRNA:       gagtgtgctctctttccttctgccacca
        Target(rc):NM_121907.1       tcaTGTGCTCTCTTCCCTTCTTCCAacg
```

### Precursor sequence and structure. Mature sequence in capital letters

```
atgttgacagaagaaagagagcacaacctgggattagcaaaaagatagttttgcccttgtcgggagTGTGCTCTCTTTCCTTCTGCCAccat   
 (((.((.((((((((((((((((((.(((((.(...((((((......))))))...).)))))..)))))))))))).)))))).)).)))
```

---

## locus\_id: 372677

family\_id: 332

### **Targets:**

At4g21770(NM\_118297.3
): pseudouridine synthase family protein

Location in genome: in an IGR, 1634 upstream of At5g60400, 15094 downstream of At5g60450

2 homologs in brassica

### Alignment between mature miRNA and predicted targets

```
        Extended mature miRNA:       tgctacgtaggagagataccgtcacc
        Target(rc):NM_118297.3       aagTACGCAGGAGAGATACCGGCgca
```

### Precursor sequence and structure. Mature sequence in capital letters

```
taaggattgcTACGTAGGAGAGATACCGTCaccactagttaacttcttagaagaggtgatggcgctatctctcctgcgaagcaaatcttta   
 (((((((((((.(((((((((((((((((((((.((..((((....)))).)).))))))))...))))))))))))).))).))))))))
```

---

## locus\_id: 355401

family\_id: 331

### **Targets:**

At3g28380(NM\_113758.1
): P-glycoprotein, putative

Location in genome: in an IGR, 4400 upstream of At5g36100, 514 downstream of At5g36120

### Alignment between mature miRNA and predicted targets

```
        Extended mature miRNA:       agagtttgctaccgccgctgccgcctg
        Target(rc):NM_113758.1       tatGTTTGATACCGCCTCTGCCGCtaa
```

### Precursor sequence and structure. Mature sequence in capital letters

```
tcacttgcggcagcggcgacgaaacgaacatatgcacctgcggcaatggcagcggtagaGTTTGCTACCGCCGCTGCCGCctgcggtga   
 (((((.((((((((((((......(((((...(((..((((.......))))..)))..)))))....))))))))))))....)))))
```

---

## locus\_id: 395616

family\_id: 330

### **Targets:**

At4g38570(NM\_120018.2
): CDP-diacylglycerol--inositol 3-phosphatidyltransferase, putative / phosphatidylinositol synthase, putative

Location in genome: in an IGR, 9051 upstream of At5g28410, 22412 downstream of At5g28350

### Alignment between mature miRNA and predicted targets

```
        Extended mature miRNA:       tctagggattgggaacgaagggagga
        Target(rc):NM_120018.2       aagAGAAATTGGGAACGAAGGGAtcc
```

### Precursor sequence and structure. Mature sequence in capital letters

```
gtctctAGGGATTGGGAACGAAGGGAggagactgcgaattttgtggcgtacaagagcgccaagtcgaaaccttccttttgctcaaatcccacgtggc   
 (((.(..(((((((((...(((((((((.((((..........((((((......))))))))))....)))))))))..))).))))))..).)))
```

---

## locus\_id: 379071

family\_id: 329

### **Targets:**

At3g14067(NM\_112261.2
): subtilase family protein

Location in genome: Contained by At5g03560 (NM\_120437: . expressed protein) in an intron on the reverse strand

### Alignment between mature miRNA and predicted targets

```
        Extended mature miRNA:       tcctctggttcctcaatcgccggttt
        Target(rc):NM_112261.2       caaTCTGCTTCCGCAATCGCCGGaat
```

### Precursor sequence and structure. Mature sequence in capital letters

```
taggatccTCTGGTTCCTCAATCGCCGGtttcttgctgaaagggtcatacggaggaggtggaggctcctg   
 (((((.((((((.((((((.((.(((..((((.....)))).))).))...)))))).)))))).)))))
```

---

## locus\_id: 271624

family\_id: 328

### **Targets:**

At3g32180(NM\_114040.1
): hypothetical protein

Location in genome: in an IGR, 333592 upstream of At4g06526, 11400 downstream of At4g06599

### Alignment between mature miRNA and predicted targets

```
        Extended mature miRNA:       caagcgtgtggaaagaggacgccggcg
        Target(rc):NM_114040.1       acgGCGTGGGGAAAGAGAACGCCGaca
```

### Precursor sequence and structure. Mature sequence in capital letters

```
gaaacaaGCGTGTGGAAAGAGGACGCCGgcgggctcgaagccatcgccgggcaaaaacaagggactcccggggtcatgggggcagacggtggttgctccttcgcgttccattctataggtgttgttc   
 ..(((((((.(((((((.(.((((((.(((((((.....)))..))))((((((..((....(.((((((......))))))).....))..))))))....))))))).))))))).)).))))).
```

---

## locus\_id: 344956

family\_id: 327

### **Targets:**

At2g31900(NM\_128748.2
): myosin family protein

Location in genome: in an IGR, 556 upstream of At5g23530, 4597 downstream of At5g23550

### Alignment between mature miRNA and predicted targets

```
        Extended mature miRNA:       cggacaacaaaccgatgcggacttagttt
        Target(rc):NM_128748.2       gcaACAGCAAACAGATGCGGACTTAGctc
```

### Precursor sequence and structure. Mature sequence in capital letters

```
ggactaatccgtagcggtttagtccataccatttttgggtcggACAACAAACCGATGCGGACTTAGttt   
 .((((((((((((.((((((.((((...(((....)))...))))...)))))).)))))).)))))).
```

---

## locus\_id: 406752

family\_id: 326

### **Targets:**

At5g15630(NM\_121567.2
): phytochelatin synthetase family protein / COBRA cell expansion protein COBL4

Location in genome: Contained by At5g42620 (NM\_123626: . expressed protein) in an intron

### Alignment between mature miRNA and predicted targets

```
        Extended mature miRNA:       atgcctcaggctttttctttttcgtg
        Target(rc):NM_121567.2       taaCCTCAGGCTTTTTCTTCTTCagg
```

### Precursor sequence and structure. Mature sequence in capital letters

```
gagatgaaaggaaaagaagacgcactgaggtatgttatgagtatctaatgtcctcctaatccagagaagaagttagctattattaacatgCCTCAGGCTTTTTCTTTTTCgtgtttc   
 ((((((...(((((((((((.((.(((((((((((((..((((.(((((.((.((((.....)).)).)).))))).))))..))))))))))))))).))))))))))).))))))
```

---

## locus\_id: 305575

family\_id: 325

### **Targets:**

At4g17150(NM\_117819.1
): expressed protein

Location in genome: in an IGR, 190281 upstream of At4g07031, 14402 downstream of At4g06676

### Alignment between mature miRNA and predicted targets

```
        Extended mature miRNA:       ctcgactgcgggggcgaagaggtggtc
        Target(rc):NM_117819.1       taaGACTGCGGGCGCGAAGAGTTGtga
```

### Precursor sequence and structure. Mature sequence in capital letters

```
actcGACTGCGGGGGCGAAGAGGTGgtcgagtacggtgagttgtgctcatgtgtctttgctctttgcggtcgagt   
 (((((((((((((((((((((..((...((((((((....)))))))).))..))))))).))))))))))))))
```

---

## locus\_id: 276194

family\_id: 324

### **Targets:**

At4g11680(NM\_117237.3
): zinc finger (C3HC4-type RING finger) family protein

Location in genome: in an IGR, 18886 upstream of At4g09670, 14686 downstream of At4g09740

### Alignment between mature miRNA and predicted targets

```
        Extended mature miRNA:       gagtactggtttctcctctatccttag
        Target(rc):NM_117237.3       gatTACTAGTCTCTCCTCTATCCTcca
```

### Precursor sequence and structure. Mature sequence in capital letters

```
gaggtattagtaggaggagggggtggtaaaagctccgggagggagtgaccaattctctctaaagcaggtcttctagacctaggagTACTGGTTTCTCCTCTATCCTtagaccc   
 ..(((...((..(((((((..(.((((....(((((.(((((((((.....))))))))).....(((((.....))))).))))))))).)..)))))))...))...))).
```

---

## locus\_id: 292091

family\_id: 323

### **Targets:**

At2g24530(NM\_128014.1
): expressed protein

Location in genome: Contained by At4g30710 (NM\_202913: . expressed protein) in an intron

### Alignment between mature miRNA and predicted targets

```
        Extended mature miRNA:       gtgattttctctcggagagtgggcacc
        Target(rc):NM_128014.1       gaaATTCTCTCTCGGAGAGTTGGCcag
```

### Precursor sequence and structure. Mature sequence in capital letters

```
ggtgATTTTCTCTCGGAGAGTGGGCaccggttcttgattcttcaatccacgtggtcccatttttggagaaaacacc   
 ((((.((((((((..(((((((((.((((((..((((....))))...)).)))))))))))))))))))))))))
```

---

## locus\_id: 352028

family\_id: 322

### **Targets:**

At1g75140(NM\_106170.3
): expressed protein

Location in genome: in an IGR, 19717 upstream of At5g32775, 81274 downstream of At5g33200

### Alignment between mature miRNA and predicted targets

```
        Extended mature miRNA:       tgggatgacccgtggtacggacgact
        Target(rc):NM_106170.3       gccGAGGACCCGTGGTTCGGACGtag
```

### Precursor sequence and structure. Mature sequence in capital letters

```
gtctgagtggGATGACCCGTGGTACGGACGactagggtgatcgaattcgttttggtaaccccggttgtctgtctgcgatcgtcctcaagtagc   
 ..(((..((((((((..((..(.(((((((((..((((.((((((.....)))))).))))..))))))))))..)).)))))).))..))).
```

---

## locus\_id: 388067

family\_id: 321

### **Targets:**

At5g24915(NM\_147913.1
):

Location in genome: in an IGR, 7910 upstream of At5g18500, 882 downstream of At5g18470

### Alignment between mature miRNA and predicted targets

```
        Extended mature miRNA:       caagcacaccctgaggatctctctctc
        Target(rc):NM_147913.1       cccGCAGCCCCTGAGGATCTCTCTgct
```

### Precursor sequence and structure. Mature sequence in capital letters

```
atcaaGCACACCCTGAGGATCTCTCTctcgtttcatcaaactgacagcagattcgtgagagataaacttggttttggggtttgtttccacaggtgagatagaggattctggtggtgggcttggt   
 (((((((.((((.(.((((((.((((...((((((((...(((....)))....(((.(((((((((((.......)))))))))))))).)))))))))))))))))).).)))).)))))))
```

---

## locus\_id: 306940

family\_id: 320

### **Targets:**

At5g18530(NM\_121858.1
): beige/BEACH domain-containing protein

Location in genome: in an IGR, 100201 upstream of At4g08097, 14937 downstream of At4g08040

### Alignment between mature miRNA and predicted targets

```
        Extended mature miRNA:       acgtcgcaagtaacacttccggattc
        Target(rc):NM_121858.1       tgcTCGCAAGTAACACTTCCACAagt
```

### Precursor sequence and structure. Mature sequence in capital letters

```
cagacgTCGCAAGTAACACTTCCGGAttcgttcttagtcctagaacggttcgggggcgttacagatagttctg   
 ((((((((....(((((.(((((((((.((((((.......))))))))))))))).))))).))).).))))
```

---

## locus\_id: 304982

family\_id: 319

### **Targets:**

At2g39720(NM\_129529.3
): zinc finger (C3HC4-type RING finger) family protein

Location in genome: in an IGR, 194656 upstream of At4g06676, 19016 downstream of At4g06603

1 homologs in brassica

### Alignment between mature miRNA and predicted targets

```
        Extended mature miRNA:       tcgatctgaggagcagatcgatcaat
        Target(rc):NM_129529.3       tacATCTGAAAAGCAGATCGATCtaa
```

### Precursor sequence and structure. Mature sequence in capital letters

```
ctcagattgatcgatctcggagtcggattgatcgatcccagtatacatgattgttttcaatcacgatgttgatgttcagatcgatcgATCTGAGGAGCAGATCGATCaatctgtag   
 ..((((((((((((((((....(((((((((((((((.((..((((.((((((....)))))).).)))...))....)))))))))))))))...).)))))))))))))))...
```

---

## locus\_id: 323834

family\_id: 318

### **Targets:**

At2g34060(NM\_128957.2
): peroxidase, putative

Location in genome: in an IGR, 20 upstream of At4g28580, 1363 downstream of At4g28570

### Alignment between mature miRNA and predicted targets

```
        Extended mature miRNA:       aggactcggcggagagatctctgacc
        Target(rc):NM_128957.2       agtAATCGGCGGAGAGCTCTCTGtgg
```

### Precursor sequence and structure. Mature sequence in capital letters

```
gcggcaggACTCGGCGGAGAGATCTCTGaccagctgcaggtaagtcaatgggatcacttcgccgtagtacgagc   
 ((....(.(((((((((((.((((((((((..((.....))..))))..)))))).)))))))).))).)..))
```

---

## locus\_id: 401711

family\_id: 317

### **Targets:**

At2g17860(NM\_127340.1
): pathogenesis-related thaumatin family protein

Location in genome: in an IGR, 41043 upstream of At5g36180, 501 downstream of At5g36110

### Alignment between mature miRNA and predicted targets

```
        Extended mature miRNA:       ttcgtcgccgctgccgcaagtgaaatc
        Target(rc):NM_127340.1       gcaGTCGCCGGTGACGCAAGTGAAtat
```

### Precursor sequence and structure. Mature sequence in capital letters

```
ttcgtttcatcaccgcaggcggcagcggcggtagcaaactctaccgctgccattgccgcaggtgcatatgttcgtttcGTCGCCGCTGCCGCAAGTGAAatcgaa   
 .(((((((((........(((((((((((((.(((.(((..((((((.((....)).)).)))).....))).)))...)))))))))))))..)))))).))).
```

---

## locus\_id: 349926

family\_id: 316

### **Targets:**

At5g16730(NM\_121679.2
): expressed protein

Location in genome: in an IGR, 2104 upstream of At5g28620, 32444 downstream of At5g28640

### Alignment between mature miRNA and predicted targets

```
        Extended mature miRNA:       gtttttcaccgcagctacttgcgttt
        Target(rc):NM_121679.2       tccTTTCACCGCTGCTACTCGCGctt
```

### Precursor sequence and structure. Mature sequence in capital letters

```
gttTTTCACCGCAGCTACTTGCGtttgccgtagcatttggccgcatcgtcttgcgttcactaaaagaacagcataacctgcggttgccgcagccgcaagtagctgtcgtgaccaaac   
 ((((.((((.(((((((((((((.((((....(((....((((((..((..(((((((.......)))).)))..)).))))))))).)))).))))))))))))).))))..))))
```

---

## locus\_id: 277863

family\_id: 315

### **Targets:**

At3g42640(NM\_114131.2
): ATPase, plasma membrane-type, putative / proton pump, putative

Location in genome: in an IGR, 2898 upstream of At4g11560, 237 downstream of At4g11580

### Alignment between mature miRNA and predicted targets

```
        Extended mature miRNA:       gagccgcatgaacccgaagaatttgtt
        Target(rc):NM_114131.2       attCCACATGAACCCCAAGAATTTcaa
```

### Precursor sequence and structure. Mature sequence in capital letters

```
agaacaaattctccaggttcatgcggctcagcttggtgatctcaacctccgtttgagCCGCATGAACCCGAAGAATTTgttct   
 ((((((((((((.(.(((((((((((((((((..(((.......)))...).)))))))))))))))).).))))))))))))
```

---

## locus\_id: 410213

family\_id: 314

### **Targets:**

At5g64190(NM\_125815.1
): expressed protein

Location in genome: in an IGR, 20687 upstream of At5g47630, 2379 downstream of At5g47530

### Alignment between mature miRNA and predicted targets

```
        Extended mature miRNA:       gaggaggcttcacgtccagcagcacc
        Target(rc):NM_125815.1       gttGAGGCTTCTCGTCCAGCAACtct
```

### Precursor sequence and structure. Mature sequence in capital letters

```
gaggtggtgctgcgacggaagcctcctcgccgatgcggaggagGAGGCTTCACGTCCAGCAGCacctc   
 ((((((.(((((.(((((((((((((((.(((...)))..))))))))))).))))))))).))))))
```

---

## locus\_id: 289077

family\_id: 313

### **Targets:**

At1g53770(NM\_104254.2
): expressed protein

Location in genome: in an IGR, 19073 upstream of At4g25790, 7309 downstream of At4g25870

### Alignment between mature miRNA and predicted targets

```
        Extended mature miRNA:       gtccctggctgcatctcccactcgaa
        Target(rc):NM_104254.2       ccaCCAGGCTGCATCACCCACTCtct
```

### Precursor sequence and structure. Mature sequence in capital letters

```
ttcgatggagatggatggcttggggaggccgaggctgagcatagctggaggaggacttaatggaggaacaacctcccgtggttgagtattaatgggagtcCCTGGCTGCATCTCCCACTCgaa   
 .((((.(((((((...((((.(((((..((.(.(((.(((...((.(((((..(.(((....)))...)..))))).)).))).))).....).))..))))))))).)))))))...)))).
```

---

## locus\_id: 301336

family\_id: 312

### **Targets:**

At5g07330(NM\_120815.2
): expressed protein

Location in genome: in an IGR, 19826 upstream of At4g03750, 12459 downstream of At4g03690

### Alignment between mature miRNA and predicted targets

```
        Extended mature miRNA:       ccgaaccttaaccgacccatttgggt
        Target(rc):NM_120815.2       taaAACCTTAACCGACCCATTTGaaa
```

### Precursor sequence and structure. Mature sequence in capital letters

```
aaccgAACCTTAACCGACCCATTTGggttgagacaaaatgcaactgacaggtttgagtctacgcttaagtttggtttgggtcagtttgaattcgagtgggtcaggtcggttggtt   
 ((((.((((...(((((((((((((((((.((((...((.(((....((..(((((((....)))))))..))..))).))..)))).)))))))))))))).))).))))))))
```

---

## locus\_id: 298202

family\_id: 311

### **Targets:**

At4g37380(NM\_119901.1
): pentatricopeptide (PPR) repeat-containing protein

Location in genome: in an IGR, 11795 upstream of At4g00030

### Alignment between mature miRNA and predicted targets

```
        Extended mature miRNA:       acttggctgttgcggtggtggaaatcg
        Target(rc):NM_119901.1       atcTGGCGGTTGCGGTGGTGGATAact
```

### Precursor sequence and structure. Mature sequence in capital letters

```
gattccaccacatcagttacaatcggaggctgatccggactTGGCTGTTGCGGTGGTGGAAAtc   
 ..(((((((((..((((((...(((((......)))))...)))))).....)))))))))...
```

---

## locus\_id: 306239

family\_id: 310

### **Targets:**

At4g05633(NM\_148246.1
): hypothetical protein

Location in genome: in an IGR, 56421 upstream of At4g07670, 65393 downstream of At4g07523

### Alignment between mature miRNA and predicted targets

```
        Extended mature miRNA:       cactcgactgcagcggcgtagaggtgg
        Target(rc):NM_148246.1       aaaTCGACTGCGGCGGCGCAGAGGgat
```

### Precursor sequence and structure. Mature sequence in capital letters

```
ttgtggcacTCGACTGCAGCGGCGTAGAGGtggtcgagtacggcgagttgtgctcaggtgtctttgctcttcccggtcgagtgttgtgg   
 ..(..(((((((((((..(.((.((((((..(.(.((((((((....)))))))).).)..)))))).)).).)))))))))))..)..
```

---

## locus\_id: 304757

family\_id: 309

### **Targets:**

At4g05633(NM\_148246.1
): hypothetical protein

Location in genome: in an IGR, 53054 upstream of At4g06603, 268470 downstream of At4g06536

### Alignment between mature miRNA and predicted targets

```
        Extended mature miRNA:       ggggaactcgactgcgacggcgcagaggtgg
        Target(rc):NM_148246.1       gtcGAAATCGACTGCGGCGGCGCAGAGGgat
```

### Precursor sequence and structure. Mature sequence in capital letters

```
gatgggGAACTCGACTGCGACGGCGCAGAGGtggtcgagtatggagagtggtgatcgggtgtctttgctcttcgtggtagagtaattcgtt   
 ((((((..((((.((..(((.((.((((((..(.((((.(((.(....).))).)))).)..)))))).)))))..)).))))..))))))
```

---

## locus\_id: 398559

family\_id: 308

### **Targets:**

At3g31350(NM\_113998.2
): hypothetical protein

Location in genome: in an IGR, 53990 upstream of At5g33280, 18135 downstream of At5g33230

### Alignment between mature miRNA and predicted targets

```
        Extended mature miRNA:       tttggatcgatcgatcccgaactgggga
        Target(rc):NM_113998.2       atgGGATCGATCGATCCCGAGCTGGcgg
```

### Precursor sequence and structure. Mature sequence in capital letters

```
aatgtccgccagctcgggatcgatcgttcccgttctagcagacgattttacttcgtggatgagttttGGATCGATCGATCCCGAACTGGggaacatt   
 (((((((.((((.(((((((((((((.(((.(..((..(..((((.......)))).)...))..).))).))))))))))))).)))))).)))))
```

---

## locus\_id: 340002

family\_id: 307

### **Targets:**

At3g42680(NM\_114135.1
): hypothetical protein

Location in genome: in an IGR, 5269 upstream of At5g15520, 4217 downstream of At5g15540

### Alignment between mature miRNA and predicted targets

```
        Extended mature miRNA:       gtcgaggctcgagcaaggcgagaaaag
        Target(rc):NM_114135.1       attGAGACTCGAGGAAGGCGAGAAtgt
```

### Precursor sequence and structure. Mature sequence in capital letters

```
tcctctctgcctagctcttgaagctcttcctgttaacgaaggcacatcattgtgcaatgcttgatgggtcGAGGCTCGAGCAAGGCGAGAAaagga   
 (((((((.((((.((((..((..(((..((((((((((...(((((....)))))..)).))))))))..)))..)))))).))))))))...)))
```

---

## locus\_id: 283311

family\_id: 306

### **Targets:**

At2g31215(NM\_179830.1
): basic helix-loop-helix (bHLH) family protein

Location in genome: in an IGR, 1326 upstream of At4g17980, 3123 downstream of At4g17990

### Alignment between mature miRNA and predicted targets

```
        Extended mature miRNA:       tattgtacaggtctaagtgtacgaatcaacag
        Target(rc):NM_179830.1       tggTGTACAAATCTAAGTGTACGAATCAAaca
```

### Precursor sequence and structure. Mature sequence in capital letters

```
ctgtttgattcgtacacttagatctgtacaacattttttagtgtacagatgtaagtgtacgttgtgtatatttatatatatatatatacactaaaaatatTGTACAGGTCTAAGTGTACGAATCAAcag   
 (((((.(((((((((((((((((((((((((...(((((((((((...((((((((((((.....)))))))))))).........)))))))))))..))))))))))))))))))))))))))))))
```

---

## locus\_id: 344690

family\_id: 305

### **Targets:**

At2g20780(NM\_127643.2
): mannitol transporter, putative

Location in genome: Contained by At5g23150 (NM\_122221: . PWWP domain-containing protein) in an intron on the reverse strand

### Alignment between mature miRNA and predicted targets

```
        Extended mature miRNA:       agcatttcagggaaggaagtgaaaattt
        Target(rc):NM_127643.2       aagATCTCAGGGAACGAAGTGAAAAatc
```

### Precursor sequence and structure. Mature sequence in capital letters

```
gaattttttacttccctgaaatgctaataaatacggtattattagcATTTCAGGGAAGGAAGTGAAAAttt   
 ((((((((..((((((((((((((((((((........)))))))))))))))))))).....))))))))
```

---

## locus\_id: 332206

family\_id: 304

### **Targets:**

At2g03000(NM\_126352.1
): zinc finger (C3HC4-type RING finger) family protein

Location in genome: Contained by At5g02310 (NM\_120309: . eceriferum3 protein, putative) in an intron on the reverse strand

### Alignment between mature miRNA and predicted targets

```
        Extended mature miRNA:       cgttctgcatgctccttgttgatgctt
        Target(rc):NM_126352.1       acgTTTGCATGCTCCTCGTTGATGtcg
```

### Precursor sequence and structure. Mature sequence in capital letters

```
tgtctacgtTCTGCATGCTCCTTGTTGATGcttatcaacaaggagatacattaacagggagcatgcaggacccagaca   
 .((((..(((((((((((((((((((((((..((((........)))))))))))))))))))))))))))..)))).
```

---

## locus\_id: 355660

family\_id: 303

### **Targets:**

At1g02150(NM\_100095.3
): pentatricopeptide (PPR) repeat-containing protein

Location in genome: in an IGR, 23485 upstream of At5g36280, 57300 downstream of At5g36650

### Alignment between mature miRNA and predicted targets

```
        Extended mature miRNA:       ttctctctagcagacaaccgaaaccacg
        Target(rc):NM_100095.3       gcaTCACTTGCAGACAACCGAAACCttt
```

### Precursor sequence and structure. Mature sequence in capital letters

```
gtgggtcattccctggttgatggtaattgagaaaacgattttgggaaccctcttctctctagcggagggttcccaattgaaaacgacgtcgttttcTCTCTAGCAGACAACCGAAACCac   
 ..(((.....))).(((((.((.((...(((((((((((.(((((((((((((.((....)).)))))))))))))...........)))))))))))..)).))..)))))........
```

---

## locus\_id: 318564

family\_id: 302

### **Targets:**

At5g14840(NM\_121488.1
):

Location in genome: in an IGR, 1471 upstream of At4g20960, 4176 downstream of At4g20940

### Alignment between mature miRNA and predicted targets

```
        Extended mature miRNA:       cgtgggcgataacgaggtcggtacaa
        Target(rc):NM_121488.1       cgcGAGTGATAACGAGGTCGGTAtgt
```

### Precursor sequence and structure. Mature sequence in capital letters

```
ggcgtGGGCGATAACGAGGTCGGTAcaatagatgacgccgagtagtattctttatttgagttttcccgtgcgctgacgttatcgtccgagctgcc   
 (((.(((((((((((...(((((..((...((.(((..(((((((......))))))).))).))...))..)))))))))))))))).)))...
```

---

## locus\_id: 357220

family\_id: 301

### **Targets:**

At1g53380(NM\_104217.3
): expressed protein

Location in genome: in an IGR, 1008 upstream of At5g38100, 15761 downstream of At5g38130

### Alignment between mature miRNA and predicted targets

```
        Extended mature miRNA:       ccgatcttcttcaagcttgtagatgc
        Target(rc):NM_104217.3       ataCTCTTCCTCAAGCTTGTAGAacg
```

### Precursor sequence and structure. Mature sequence in capital letters

```
tacggaggaggctgcgagataagaaatattgaagaagcttcggtaaccatcactccttggaggtatggttataatgccgATCTTCTTCAAGCTTGTAGAtgcttggta   
 (((.(((.(..(((((((..........(((((((((..(((((((((((..(((....)))..))))).....)))))).))))))))).))))))).).))).)))
```

---

## locus\_id: 398852

family\_id: 300

### **Targets:**

At5g32590(NM\_122838.1
): myosin heavy chain-related

Location in genome: in an IGR, 8135 upstream of At5g33320, 9456 downstream of At5g33303

### Alignment between mature miRNA and predicted targets

```
        Extended mature miRNA:       cgatgactggaggagggggaccaattga
        Target(rc):NM_122838.1       cggTGACTGGAGGAGGAGGACCCATaga
```

### Precursor sequence and structure. Mature sequence in capital letters

```
tctcgccgaggattttcactctgaccggtcttttcgctcccaaatttggaagaagatgcgaTGACTGGAGGAGGGGGACCAATtgagcgagg   
 .((((((((((.((..(.((((..((((((...(((((((((....))).....)).)))).)))))).)))))..))))..))).))))).
```

---

## locus\_id: 305951

family\_id: 299

### **Targets:**

At5g29040(NM\_122788.1
):

Location in genome: in an IGR, 76444 upstream of At4g07390, 9722 downstream of At4g07310

### Alignment between mature miRNA and predicted targets

```
        Extended mature miRNA:       accggccgctatggcttttgcggtgaggagtg
        Target(rc):NM_122788.1       cttGGCCGCTATGGCCTTCGCGGTGAGGAatg
```

### Precursor sequence and structure. Mature sequence in capital letters

```
gcggctcgctgcttagtccatttcgggaggcaggccgaggtgaatcacgacggggtgatcgttctctgcgggatgaccGGCCGCTATGGCTTTTGCGGTGAGGAgt   
 ((..((((((((..((.((((..(((..((((..(((.((.(((((((......)))))...)).)).)))..)).))..)))..))))))...))))))))..))
```

---

## locus\_id: 305872

family\_id: 298

### **Targets:**

At3g32190(NM\_114041.1
): hypothetical protein

Location in genome: in an IGR, 77929 upstream of At4g07390, 8237 downstream of At4g07310

### Alignment between mature miRNA and predicted targets

```
        Extended mature miRNA:       gagttcgtggatggaggaagttctggcggggt
        Target(rc):NM_114041.1       gtaTTCGTGGATGGAAGAATTTCTGGCGGagt
```

### Precursor sequence and structure. Mature sequence in capital letters

```
gtatcacccggctcgccagtagtagactgctcgctaggaaccccggagtctgaaccatcatcgtccgcgatgaccactgtctccgagTTCGTGGATGGAGGAAGTTCTGGCGGggtggctac   
 .......(((.((((((((.....((((.(((.(((.((((..(((((.(.......((((((....)))))).....).))))).)))).)))...)))..)))))))))))).)))....
```

---

## locus\_id: 351492

family\_id: 297

### **Targets:**

At4g24890(NM\_118621.2
): calcineurin-like phosphoesterase family protein

Location in genome: in an IGR, 49311 upstream of At5g30520, 3292 downstream of At5g31719

### Alignment between mature miRNA and predicted targets

```
        Extended mature miRNA:       gaggggcttcgggttggagagagtgt
        Target(rc):NM_118621.2       agtGGGCTTGGGGTTGGAGATAGcaa
```

### Precursor sequence and structure. Mature sequence in capital letters

```
gttgagGGGCTTCGGGTTGGAGAGAGtgttgtacgaccaaacgtctcccgagccgaacctaaacaac   
 ((((..(((.(((((.((((.((((.((((.........)))))))))))).))))))))...))))
```

---

## locus\_id: 270836

family\_id: 296

### **Targets:**

At4g00230(NM\_116240.2
): subtilisin-like serine endopeptidase (XSP1)

Location in genome: Contained by At4g06526 (NM\_178979: . hypothetical protein) in an intron

### Alignment between mature miRNA and predicted targets

```
        Extended mature miRNA:       gtcgcccatggtcgaggaaactctact
        Target(rc):NM_116240.2       ctaGACCATGGTCTAGGAAACTCTctg
```

### Precursor sequence and structure. Mature sequence in capital letters

```
gaatcgtcGCCCATGGTCGAGGAAACTCTactcgtgactagaaattggtcgcctgtggtcgagagaacttgaccgtgagtaaaggatcgtcgactgtggtctaggagattc   
 (((((..(..((((((((((.((..((.(((((((((((((...))))))))....(((((((....)))))))..))))).))..)).))))))))))....)..)))))
```

---

## locus\_id: 351716

family\_id: 295

### **Targets:**

At3g32190(NM\_114041.1
): hypothetical protein

Location in genome: in an IGR, 10250 upstream of At5g32550, 13468 downstream of At5g32590

### Alignment between mature miRNA and predicted targets

```
        Extended mature miRNA:       actggttcaggagttttcaccgatgt
        Target(rc):NM_114041.1       accGGTTCAGGAGTTACCACCGAcat
```

### Precursor sequence and structure. Mature sequence in capital letters

```
gttgttcggtggagacccttggttctgaatcatcgtcatcctctatggtcaccgtttcggtgttcgtggacaaagggacctcgggcggaatgactactGGTTCAGGAGTTTTCACCGAtgtggcagc   
 ((..((((((((((((......((((((((((..(((((((.((..((((.((.......((((....))))..))))))...)).)).)))))...))))))))))))))))))))).)..))...
```

---

## locus\_id: 361446

family\_id: 294

### **Targets:**

At3g22800(NM\_113179.3
): leucine-rich repeat family protein / extensin family protein

Location in genome: in an IGR, 930 upstream of At5g43650, 592 downstream of At5g43660

### Alignment between mature miRNA and predicted targets

```
        Extended mature miRNA:       cgtgtggtggcgtcggcgatggacta
        Target(rc):NM_113179.3       gagGTGGTGGCGGCGGCGAAGGAtac
```

### Precursor sequence and structure. Mature sequence in capital letters

```
gtgacgcgtGTGGTGGCGTCGGCGATGGActagcggcgctggtgattggttgtgcggtttctttaacgccggagattacttgattcgccaccattgttgc   
 (..(((...(((((((((((((((.((((..(((.((((............)))).)))..)))).)))))).............))))))))))))..)
```

---

## locus\_id: 405919

family\_id: 293

### **Targets:**

At3g61760(NM\_116041.2
): dynamin-like protein B (DL1B)

Location in genome: in an IGR, 2628 upstream of At5g41530, 687 downstream of At5g41510

### Alignment between mature miRNA and predicted targets

```
        Extended mature miRNA:       atgatgatgctgttgggtttctcttt
        Target(rc):NM_116041.2       agtATGATGCAGTTGGGCTTCTCaat
```

### Precursor sequence and structure. Mature sequence in capital letters

```
aacgtaaaaaagaaacccaacagcatcatcatttgaaaagaaaccatacgtatgATGATGCTGTTGGGTTTCTCttttacgtt   
 (((((((((.((((((((((((((((((((((.((.............)).)))))))))))))))))))))).)))))))))
```

---

## locus\_id: 290915

family\_id: 292

### **Targets:**

At4g28810(NM\_119025.1
): expressed protein

Location in genome: in an IGR, 8010 upstream of At4g28770, 8994 downstream of At4g28820

### Alignment between mature miRNA and predicted targets

```
        Extended mature miRNA:       attggaggagggtctctgtgtttgact
        Target(rc):NM_119025.1       atcGGAGGAGGGTATCTGTGTCTGgcc
```

### Precursor sequence and structure. Mature sequence in capital letters

```
ggaagtggaagcggggcattttcttctccgttgccgcttccgctaccacgaagaatgggtggtggaggaagagatggtggtgtattGGAGGAGGGTCTCTGTGTTTGactggttct   
 ...(((.(((((((((.((((((((((.((..(((.(((((.(((((((.........))))))).)))))....)))..))....)))))))))))))))).))).)))......
```

---

## locus\_id: 351779

family\_id: 291

### **Targets:**

At2g23140(NM\_127878.2
): armadillo/beta-catenin repeat family protein / U-box domain-containing protein

Location in genome: in an IGR, 17117 upstream of At5g32550, 6601 downstream of At5g32590

### Alignment between mature miRNA and predicted targets

```
        Extended mature miRNA:       agcatcttctctagacattgcgactt
        Target(rc):NM_127878.2       ttaATCTTCTCTAGACATTGCTCaag
```

### Precursor sequence and structure. Mature sequence in capital letters

```
agtttgttgcatggtcttgagggtttcagctatcgaagcgttggtagcATCTTCTCTAGACATTGCGActtact   
 (((..((((((..((((.((((((....((((((((....))))))))))))))...))))..))))))..)))
```

---

## locus\_id: 315762

family\_id: 290

### **Targets:**

At4g27510(NM\_118886.1
): expressed protein

Location in genome: in an IGR, 27428 upstream of At4g17150, 7351 downstream of At4g17080

1 homologs in brassica

### Alignment between mature miRNA and predicted targets

```
        Extended mature miRNA:       caagcttctgagagacctgagagctc
        Target(rc):NM_118886.1       ttgGCTTCTGAGAAACCTGAGAGgac
```

### Precursor sequence and structure. Mature sequence in capital letters

```
gttgcaaGCTTCTGAGAGACCTGAGAGctctagttgtgagggttctaggtttccataagtttcaac   
 ((((.((((((.((.(((((((.((((((((.......))))))))))))))))).))))))))))
```

---

## locus\_id: 264971

family\_id: 289

### **Targets:**

At1g18485(NM\_101706.2
): pentatricopeptide (PPR) repeat-containing protein

Location in genome: in an IGR, 2859 downstream of At4g00010

### Alignment between mature miRNA and predicted targets

```
        Extended mature miRNA:       tcgaccattcctcaaccgggtcgagga
        Target(rc):NM_101706.2       gtcATCATTCCTTAACCGGGTCGAccc
```

### Precursor sequence and structure. Mature sequence in capital letters

```
gaattctcagactcggtcgacgattccaacacctggtcgacgaatcctcgaacccgatagattttcgtcgACCATTCCTCAACCGGGTCGAggattc   
 ((((((((.((((((((.((.((..........(((((((((((((.(((....)))..)))..)))))))))).)).)).))))))))))))))))
```

---

## locus\_id: 397826

family\_id: 288

### **Targets:**

At3g42990(NM\_114167.1
): hypothetical protein

Location in genome: in an IGR, 573 upstream of At5g31685, 40314 downstream of At5g30500

### Alignment between mature miRNA and predicted targets

```
        Extended mature miRNA:       cctggagtatgcgtcctggaacgattc
        Target(rc):NM_114167.1       cccGGAGTACGCGTCTTGGAACGActc
```

### Precursor sequence and structure. Mature sequence in capital letters

```
ggctcgggatgattccttgatgcgatttccaaggtacttgtcatcggcctGGAGTATGCGTCCTGGAACGAttccgagcc   
 ((((((((((..((((..((((((..(((((.(((...........))))))))..))))))..))))..))))))))))
```

---

## locus\_id: 423107

family\_id: 287

### **Targets:**

At1g17620(NM\_101625.3
): expressed protein

Location in genome: in an IGR, 11030 upstream of At5g67430, 2892 downstream of At5g67400

### Alignment between mature miRNA and predicted targets

```
        Extended mature miRNA:       tcgggtgaagcgaggttgttgagcac
        Target(rc):NM_101625.3       gacGGTGAAGCTAGGTCGTTGAGgac
```

### Precursor sequence and structure. Mature sequence in capital letters

```
cgccgtcgGGTGAAGCGAGGTTGTTGAGcacccaaatgatctgttgagccaacgtggcgtcgtttgattcgatggcg   
 (((((((((((.((((((.(((((((.((...(((........))).)))))))..)).)))))).)))))))))))
```

---

## locus\_id: 370006

family\_id: 286

### **Targets:**

At5g16440(NM\_121649.3
): isopentenyl-diphosphate delta-isomerase I / isopentenyl diphosphate:dimethylallyl diphosphate isomerase I (IPP1)

Location in genome: in an IGR, 9075 upstream of At5g55893, 4860 downstream of At5g55910

### Alignment between mature miRNA and predicted targets

```
        Extended mature miRNA:       agacagatcggatcggaggaagagag
        Target(rc):NM_121649.3       ccgGAGATCGGATCGGAGAAAGAcgg
```

### Precursor sequence and structure. Mature sequence in capital letters

```
tttccaatctctgccttttctctggtctctatatcgtcgtttttgctacatttgattgggagtagtaaagatgaagagaCAGATCGGATCGGAGGAAGAgaggaag   
 .((((..(((((.(((((..((((((((.(...((.(((((((((((((.(((....))).))))))))))))).)).).))))))))..))))).))))))))).
```

---

## locus\_id: 348614

family\_id: 285

### **Targets:**

At2g47440(NM\_130312.3
): DNAJ heat shock N-terminal domain-containing protein

Location in genome: in an IGR, 7088 upstream of At5g27960, 15916 downstream of At5g27980

### Alignment between mature miRNA and predicted targets

```
        Extended mature miRNA:       ggatgaagcgacgtcgttttccttat
        Target(rc):NM_130312.3       agcTGAAGCGACGTCGTTTGCCTcct
```

### Precursor sequence and structure. Mature sequence in capital letters

```
atgggtggaTGAAGCGACGTCGTTTTCCTtatgtttcgaagagttgaaaaaatgagaaaacgatgtcgtttctcaatcgggtcat   
 ((((.(.((((((((((((((((((((.((((.((((((....))))))..)))))))))))))))))))))...))).).))))
```

---

## locus\_id: 376517

family\_id: 284

### **Targets:**

At1g76910(NM\_106342.1
): hypothetical protein

Location in genome: in an IGR, 5714 upstream of At5g66550, 8192 downstream of At5g66600

### Alignment between mature miRNA and predicted targets

```
        Extended mature miRNA:       ctgaagcccattgttacggcccattcgg
        Target(rc):NM_106342.1       gcaAAGCCCATTGATAAGGCCCATTgat
```

### Precursor sequence and structure. Mature sequence in capital letters

```
ggcaactgAAGCCCATTGTTACGGCCCATTcggaggaagtcgagaaaagggctcaattcgaaacgtttaacgatgggctttgtgcc   
 ((((...((((((((((((((.(((...((((((...((((........))))...))))))..))))))))))))))))).))))
```

---

## locus\_id: 172008

family\_id: 283

### **Targets:**

At5g55600(NM\_124942.2
): agenet domain-containing protein / bromo-adjacent homology (BAH) domain-containing protein

Location in genome: in an IGR, 9593 upstream of At2g39190, 6993 downstream of At2g39140

### Alignment between mature miRNA and predicted targets

```
        Extended mature miRNA:       ggagccatgcatatcctcatacatatata
        Target(rc):NM_124942.2       tagGCCACGCTTATCCTCATACATATctt
```

### Precursor sequence and structure. Mature sequence in capital letters

```
tgtagatgtatatgtattatatatgtatgcctggctccctgtatgccatatgctgagcccatcgagtatcgatgacctccgtggatggcgtatgaggaGCCATGCATATCCTCATACATATataattatg   
 .((((.(((((((((((.......((((((.(((((((..(((((((((.(((.(((..(((((.....)))))..))).))).)))))))))..))))))).))))))....))))))))))).)))).
```

---

## locus\_id: 171565

family\_id: 282

### **Targets:**

At3g57320(NM\_115592.3
): expressed protein

Location in genome: in an IGR, 2473 upstream of At2g38330, 4052 downstream of At2g38320

### Alignment between mature miRNA and predicted targets

```
        Extended mature miRNA:       ccatcctgagtttcattggctcttct
        Target(rc):NM_115592.3       cgcTCCTGAGCTTCAGTGGCTCTgtg
```

### Precursor sequence and structure. Mature sequence in capital letters

```
gtagagaagaatctgtaaagctcaggagggatagcgccatgatgatcacattcgttatctattttttggcgctatccaTCCTGAGTTTCATTGGCTCTtcttactac   
 ((((((((((..(.((.((((((((((.(((((((((((.((((((.((....)).))).)))...))))))))))).)))))))))).)).)..))))))..))))
```

---

## locus\_id: 126615

family\_id: 281

### **Targets:**

At3g17265(NM\_180271.1
): F-box family protein

Location in genome: in an IGR, 450 upstream of At2g24500, 24518 downstream of At2g24550

### Alignment between mature miRNA and predicted targets

```
        Extended mature miRNA:       cccaacaactgatagagctgcaatatc
        Target(rc):NM_180271.1       tctAACAACTGATAGAACCGCAATttg
```

### Precursor sequence and structure. Mature sequence in capital letters

```
tctttcccAACAACTGATAGAGCTGCAATatcttcacgattaaaactcttaaacgaaagaggaaaacgtggttctatcagttgttggaaaaga   
 (((((.(((((((((((((((((..(....(((((.((.((((.....)))).))...)))))....)..))))))))))))))))).)))))
```

---

## locus\_id: 206190

family\_id: 280

### **Targets:**

At1g59940(NM\_104686.2
): two-component responsive regulator / response regulator 3 (ARR3)

Location in genome: in an IGR, 58247 upstream of At3g42680, 16726 downstream of At3g42723

### Alignment between mature miRNA and predicted targets

```
        Extended mature miRNA:       gctgaggcggcgtcggtcgagtgcgg
        Target(rc):NM_104686.2       gcgGAGGCGGCGGCGGTGGAGTGgtc
```

### Precursor sequence and structure. Mature sequence in capital letters

```
tcgatagcggcggcactggaaaggtcgagtgagttgttgaaatcggctgaggcggcgtcggtagagtgcgttgttgaaatcggctGAGGCGGCGTCGGTCGAGTGcggctacga   
 (((.((((.((..((((.((....)).))))...........((((((((.((.((.(((((.((...((....))...)).))))).)).)).))))))))..)).)))))))
```

---

## locus\_id: 200547

family\_id: 279

### **Targets:**

At1g79950(NM\_106644.3
): helicase-related

Location in genome: in an IGR, 64970 upstream of At3g30390, 67490 downstream of At3g30470

### Alignment between mature miRNA and predicted targets

```
        Extended mature miRNA:       cactctgcaagttgtggttgttctac
        Target(rc):NM_106644.3       acaTCTGCAAGTTGTGATTGTTCatc
```

### Precursor sequence and structure. Mature sequence in capital letters

```
tggccgttctagaggttattgccgctctgcaggttgtggccgctctaggttgtggcagctcagcaggttgtggccacTCTGCAAGTTGTGGTTGTTCtacaggtta   
 (((((....(((((...((..(.(((.((((((..((((((((.....((((.(....).)))).....))))))))))))))))).)..))..)))))..)))))
```

---

## locus\_id: 212929

family\_id: 278

### **Targets:**

At5g38340(NM\_123195.1
): disease resistance protein (TIR-NBS-LRR class), putative

Location in genome: in an IGR, 6322 upstream of At3g49920, 10044 downstream of At3g49960

### Alignment between mature miRNA and predicted targets

```
        Extended mature miRNA:       gaaagagagattccaaggtgatgggt
        Target(rc):NM_123195.1       tatCGAGAGATTCCAAGCTGATGttg
```

### Precursor sequence and structure. Mature sequence in capital letters

```
agaggaagaaAGAGAGATTCCAAGGTGATGggtgcgaacggctccgtgtccccgcgaatagttgcctcctcaaccattggttctttttttctttcttt   
 (((((((((((((((((..(((((((...(((.((.(((.....((((....))))....))))).)))...))).)))).)))))))))))))))))
```

---

## locus\_id: 140321

family\_id: 277

### **Targets:**

At3g53370(NM\_115197.2
): DNA-binding S1FA family protein

Location in genome: Contained by At2g44735 (NM\_180092: . expressed protein) in an intron on the reverse strand

### Alignment between mature miRNA and predicted targets

```
        Extended mature miRNA:       tggtggcttcatcagccgctttttct
        Target(rc):NM_115197.2       cctTGGCTTCAGCAGCCGCCTTTccg
```

### Precursor sequence and structure. Mature sequence in capital letters

```
ggcaagagcggttcgctaagtccaccaacgccacctaccggtcccgtataacacggctcacgttagcctccaaccatgatgactacggtggcttggTGGCTTCATCAGCCGCTTTTtct   
 ((.((((((((((....(((.(((((((.((((((....((((.(((.......((((......)))).......)))..))))..))))))))))))))))....)))))))))).))
```

---

## locus\_id: 124717

family\_id: 276

### **Targets:**

At3g48780(NM\_114737.2
): serine C-palmitoyltransferase, putative

Location in genome: in an IGR, 253 upstream of At2g21950, 2065 downstream of At2g21970

### Alignment between mature miRNA and predicted targets

```
        Extended mature miRNA:       tcgtcgtcttcttcttctcccaattgg
        Target(rc):NM_114737.2       tgtTCGTCTTCTTCTTCTCCCTCTatc
```

### Precursor sequence and structure. Mature sequence in capital letters

```
gcttcatcgTCGTCTTCTTCTTCTCCCAATtggaaacgtcggaggacgaagggaagacgaggaagc   
 (((((.(((((.(((.((((((((((....((....))..)))))).))))))).))))).)))))
```

---

## locus\_id: 132310

family\_id: 275

### **Targets:**

At2g37660(NM\_129322.2
): expressed protein

Location in genome: in an IGR, 4170 upstream of At2g32140, 11025 downstream of At2g32180

### Alignment between mature miRNA and predicted targets

```
        Extended mature miRNA:       gcggttgcggcagcggcaacggtaacact
        Target(rc):NM_129322.2       gttGTTGCGGCGGCGGAAACGGTAACcga
```

### Precursor sequence and structure. Mature sequence in capital letters

```
ctgcgGTTGCGGCAGCGGCAACGGTAACactgccgcaggttgttgttcgtttcgtcgccgccaacgttgccgcagccgctgtcgcaaccgcag   
 (((((((((((((((((((..(((((((..((..((.(((...((.......))..)))))))..)))))))..)))))))))))))))))))
```

---

## locus\_id: 153122

family\_id: 274

### **Targets:**

At2g27610(NM\_128320.1
): pentatricopeptide (PPR) repeat-containing protein

Location in genome: in an IGR, 5808 upstream of At2g13290, 11914 downstream of At2g13270

### Alignment between mature miRNA and predicted targets

```
        Extended mature miRNA:       tttagcatctagaagagcagttcatt
        Target(rc):NM_128320.1       ataAGCATCTAAAAGAGCAGTTCcaa
```

### Precursor sequence and structure. Mature sequence in capital letters

```
atgaactgcacttttaaagctttatcaaacttgatatttAGCATCTAGAAGAGCAGTTCat   
 (((((((((.((((((..(((.(((((....)))))...)))...)))))).)))))))))
```

---

## locus\_id: 155471

family\_id: 273

### **Targets:**

At1g46696(NM\_103606.1
): hypothetical protein

Location in genome: in an IGR, 9856 upstream of At2g15130, 11120 downstream of At2g15090

### Alignment between mature miRNA and predicted targets

```
        Extended mature miRNA:       gcgaacttctcctcatcagcccggactga
        Target(rc):NM_103606.1       gcaAACTTCTCCTCGTCAGCTCGGACcga
```

### Precursor sequence and structure. Mature sequence in capital letters

```
cgaagtgagttcagtcgggtcgggacgaggagtcttcggctccgcagcatgtctagcgAACTTCTCCTCATCAGCCCGGACtgattcaag   
 .....(((((.(((((((((...((.((((((..((((.((..((.....))..))))))...)))))).)).)))).))))))))))..
```

---

## locus\_id: 155468

family\_id: 272

### **Targets:**

At1g46696(NM\_103606.1
): hypothetical protein

Location in genome: in an IGR, 9912 upstream of At2g15130, 11067 downstream of At2g15090

### Alignment between mature miRNA and predicted targets

```
        Extended mature miRNA:       aagtgagttcagtcgggtcgggacga
        Target(rc):NM_103606.1       aaaTGAGTTCAGTAGGGTCAGGAtga
```

### Precursor sequence and structure. Mature sequence in capital letters

```
aggagcgaagTGAGTTCAGTCGGGTCGGGAcgaggagtcttcggctccgcagcatgtctagcgaacttctcctcatcagcccggactgattcaagctcct   
 ((((((....(((((.(((((((((...((.((((((..((((.((..((.....))..))))))...)))))).)).)))).)))))))))).))))))
```

---

## locus\_id: 119717

family\_id: 271

### **Targets:**

At5g40420(NM\_123406.2
): glycine-rich protein / oleosin

Location in genome: in an IGR, 40938 upstream of At2g15045, 33656 downstream of At2g15180

### Alignment between mature miRNA and predicted targets

```
        Extended mature miRNA:       agtccgggctgatgaggagaagttcg
        Target(rc):NM_123406.2       taaCCGGGCTGAAGAGGAGAAATagt
```

### Precursor sequence and structure. Mature sequence in capital letters

```
tcagtCCGGGCTGATGAGGAGAAGTtcgctagacatgctgcggagccgaagactcctcgtcccgacccgactga   
 ((((((.((((.(((((((((..((((((.((.....)))).))))......)))))))))..).)))))))))
```

---

## locus\_id: 118481

family\_id: 270

### **Targets:**

At5g22760(NM\_122182.2
): PHD finger family protein

Location in genome: in an IGR, 12389 upstream of At2g14260, 1001 downstream of At2g14290

### Alignment between mature miRNA and predicted targets

```
        Extended mature miRNA:       agatttacttctcggtgccaaattgacca
        Target(rc):NM_122182.2       gtgTTTACTTTTCGGTGCCAAATTGAaat
```

### Precursor sequence and structure. Mature sequence in capital letters

```
cgagggtggtccactttggtgggaagatgtcaagagtgtggcaaagcgctcaagaTTTACTTCTCGGTGCCAAATTGAccaactttg   
 (((((.(((((...((((((((((((..(((..((((((......))))))..)))...))))))...))))))..))))).)))))
```

---

## locus\_id: 127334

family\_id: 269

### **Targets:**

At3g47730(NM\_114641.3
): ABC transporter family protein

Location in genome: in an IGR, 3895 upstream of At2g25090, 2959 downstream of At2g25100

### Alignment between mature miRNA and predicted targets

```
        Extended mature miRNA:       aagagaaacgcaaagaaactgacagaag
        Target(rc):NM_114641.3       accAGAAACACAAAGAAACCGACAGttg
```

### Precursor sequence and structure. Mature sequence in capital letters

```
caagAGAAACGCAAAGAAACTGACAGaagagagtgagcacacaaaggcaatttgcatatcattgcacttgcttctcttgcgtgctcactgctctttctgtcagattccggtgctgatctctttg   
 .((((((...(((..(((.(((((((((((((((((((((.(((((((((..((((......)))).))))))...))).))))))))).))).))))))))).)))...)))...))))))..
```

---

## locus\_id: 114300

family\_id: 268

### **Targets:**

At3g13940(NM\_112248.3
): expressed protein

Location in genome: in an IGR, 70534 upstream of At2g07760, 55214 downstream of At2g09840

### Alignment between mature miRNA and predicted targets

```
        Extended mature miRNA:       tagactcttgcggctggacgactcag
        Target(rc):NM_112248.3       tcaACTCTTGCGCCTGGAGGACTtac
```

### Precursor sequence and structure. Mature sequence in capital letters

```
gctagACTCTTGCGGCTGGACGACTcaggcgaacgaattcattttggaaacctggattggccgactgcgggcgtctaac   
 ..(((((.(((((((.(((.(((..((((....((((.....))))....))))..))).))).))))))).)))))..
```

---

## locus\_id: 112701

family\_id: 267

### **Targets:**

At1g72960(NM\_105954.1
): root hair defective 3 GTP-binding (RHD3) family protein

Location in genome: in an IGR, 426 upstream of At2g07687, 15574 downstream of At2g07755

### Alignment between mature miRNA and predicted targets

```
        Extended mature miRNA:       aatccatctcggtcgaagagctgatt
        Target(rc):NM_105954.1       ataCCATCTCCGTCGATGAGCTGtac
```

### Precursor sequence and structure. Mature sequence in capital letters

```
cgacaaatCCATCTCGGTCGAAGAGCTGattccattcgggatcggcaaatcgccgagtggaggggaag   
 ...(...((((.(((((.(((...(((((((((....)))))))))...))))))))))))...)...
```

---

## locus\_id: 158902

family\_id: 266

### **Targets:**

At4g30000(NM\_119146.3
): dihydropterin pyrophosphokinase, putative / dihydropteroate synthase, putative / DHPS, putative

Location in genome: Contained by At2g20010 (NM\_127559: . expressed protein) in an intron on the reverse strand

### Alignment between mature miRNA and predicted targets

```
        Extended mature miRNA:       agatctatgaagcgacggagacggagat
        Target(rc):NM_119146.3       ggtTCTAAGAAGCGACGGAGAAGGAatc
```

### Precursor sequence and structure. Mature sequence in capital letters

```
gtctccgtctccgattcgtttcttcatccctaacgccttcttcaccttactcgcagctgtagaagtcagagaTCTATGAAGCGACGGAGACGGAgat   
 (((((((((((((..(((((((...(((.((...(.(((((.((.((.......)).)).))))).))).)))....))))))))))))))))))))
```

---

## locus\_id: 169768

family\_id: 265

### **Targets:**

At3g33530(NM\_114071.2
): transducin family protein / WD-40 repeat family protein

Location in genome: in an IGR, 7006 upstream of At2g35790, 347 downstream of At2g35760

### Alignment between mature miRNA and predicted targets

```
        Extended mature miRNA:       gctttggtgatggctttgatgttgctta
        Target(rc):NM_114071.2       cggTTGGTGATGGCTTCGATGCTGCcat
```

### Precursor sequence and structure. Mature sequence in capital letters

```
ggtggctTTGGTGATGGCTTTGATGTTGCttatgtccgtgttggtttctgccgaggaaacaccaaccatcggacagaggatagactcagccgcaaccgactttacc   
 (((((..(((((..(((((..((.(((.(((.((((((.((((((((((.....))))..))))))...)))))).)))...))))))))))..)))))..)))))
```

---

## locus\_id: 254322

family\_id: 264

### **Targets:**

At3g25950(NM\_113499.1
): hypothetical protein

Location in genome: in an IGR, 533 upstream of At3g47030, 490 downstream of At3g47020

### Alignment between mature miRNA and predicted targets

```
        Extended mature miRNA:       gtgaacggcgtgagtgccgtagatta
        Target(rc):NM_113499.1       tatAACGGCGGGAGTGCCGTGGAaga
```

### Precursor sequence and structure. Mature sequence in capital letters

```
agatcatcatccatggcactgacgccgttcacggcaactgccgtagacgttgttgttgccgtgAACGGCGTGAGTGCCGTAGAttattggct   
 ...(((..(((.((((((((.(((((((((((((((((.((((....))..)).))))))))))))))))).)))))))).)))...)))..
```

---

## locus\_id: 243447

family\_id: 263

### **Targets:**

At1g50280(NM\_103912.2
): phototropic-responsive NPH3 family protein

Location in genome: in an IGR, 62973 upstream of At3g30180, 1171 downstream of At3g29970

### Alignment between mature miRNA and predicted targets

```
        Extended mature miRNA:       gaattatggagggatctttgggtttctttt
        Target(rc):NM_103912.2       gatTTCTGGAGGGATCTTAGGGTTTCTggc
```

### Precursor sequence and structure. Mature sequence in capital letters

```
gtggagggaaTTATGGAGGGATCTTTGGGTTTCTtttcagaagaagaagaagaagaattgtagatcccgccacaattccgtcaac   
 ...((.((((((.(((.(((((((..(..(((((((((.......).))))))))..)...))))))).))).)))))).))...
```

---

## locus\_id: 185439

family\_id: 262

### **Targets:**

At1g04230(NM\_100304.3
): expressed protein

Location in genome: in an IGR, 146 upstream of At3g13080, 1581 downstream of At3g13090

### Alignment between mature miRNA and predicted targets

```
        Extended mature miRNA:       tcttcttcctcttggcttcgtgagcgtg
        Target(rc):NM_100304.3       ttcTCTTCCTCTTGGGTTTGTGAGCatc
```

### Precursor sequence and structure. Mature sequence in capital letters

```
agtgctctggtggggtttgagaggagaggaacggagcgttgtctctttctccttctTCTTCCTCTTGGCTTCGTGAGCgtgct   
 ((..(.((..(((((.(..((((((((((((.((((.(........).)))))))))).))))))..))))))..)).)..))
```

---

## locus\_id: 140322

family\_id: 261

### **Targets:**

At1g24450(NM\_102290.2
): ribonuclease III family protein

Location in genome: Contained by At2g44735 (NM\_180092: . expressed protein) in an intron on the reverse strand

### Alignment between mature miRNA and predicted targets

```
        Extended mature miRNA:       tggcttggtggcttcatcagccgctt
        Target(rc):NM_102290.2       aacCTTGATGGCTTCATCAACCGttc
```

### Precursor sequence and structure. Mature sequence in capital letters

```
ggcaagagcggttcgctaagtccaccaacgccacctaccggtcccgtataacacggctcacgttagcctccaaccatgatgactacggtggCTTGGTGGCTTCATCAGCCGctttttct   
 ((.((((((((((....(((.(((((((.((((((....((((.(((.......((((......)))).......)))..))))..))))))))))))))))....)))))))))).))
```

---

## locus\_id: 168285

family\_id: 260

### **Targets:**

At5g23190(NM\_122225.2
): cytochrome P450 family protein

Location in genome: in an IGR, 498 upstream of At2g33480, 6157 downstream of At2g33450

### Alignment between mature miRNA and predicted targets

```
        Extended mature miRNA:       gggtggaagtcgttggttagcaaaca
        Target(rc):NM_122225.2       gaaTGGAAGTCGTTAGTTAGCTAgtg
```

### Precursor sequence and structure. Mature sequence in capital letters

```
gattcaagggTGGAAGTCGTTGGTTAGCAAacattagtgtctcaggacaagatagctcaggacaagatgtccgagcggttcagaatcagtaatgtttttttccggagactctactcgttgagtt   
 ((((((((((((((.(((.((((..((.((((((((.((..((.((((......((((.((((.....)))))))).)))).))..)).))))))))))..)))).)))))))))).)))))))
```

---

## locus\_id: 225251

family\_id: 259

### **Targets:**

At4g30320(NM\_119178.1
): allergen V5/Tpx-1-related family protein

Location in genome: in an IGR, 786 upstream of At3g06360, 8172 downstream of At3g06330

### Alignment between mature miRNA and predicted targets

```
        Extended mature miRNA:       tgggctgcgttttttgggcccatagg
        Target(rc):NM_119178.1       cttGCTGCGTTTTGTGGACCCATgaa
```

### Precursor sequence and structure. Mature sequence in capital letters

```
tagtggGCTGCGTTTTTTGGGCCCATaggaagcctctttagtgggctcgaattgcatagcaaacta   
 ((((..((((.((..(((((((((((((((....))))..)))))))))))..)).))))..))))
```

---

## locus\_id: 248528

family\_id: 258

### **Targets:**

At2g13500(NM\_126932.1
): hypothetical protein

Location in genome: in an IGR, 29771 upstream of At3g42070, 28162 downstream of At3g41979

### Alignment between mature miRNA and predicted targets

```
        Extended mature miRNA:       gagaacactatctctgaaggcagccct
        Target(rc):NM_126932.1       caaAACACTATCTCTGAAAACAGCtct
```

### Precursor sequence and structure. Mature sequence in capital letters

```
ggaggaggagctactcttggaggtggtgtcactggagctggaaacgaagtctcagtcatctgagactgagaattcaaacttgatctgagAACACTATCTCTGAAGGCAGCccttgctct   
 ((((((((.(((.(..(..((((((((((..(((((...((....(((.((((((((......)))))))).)))...))...))).)).))))))))))..)..).))))))).))))
```

---

## locus\_id: 167445

family\_id: 257

### **Targets:**

At1g61040(NM\_104784.3
): plus-3 domain-containing protein

Location in genome: in an IGR, 987 upstream of At2g32160, 449 downstream of At2g32150

### Alignment between mature miRNA and predicted targets

```
        Extended mature miRNA:       ttgccgctgccgcaaccgcagccgct
        Target(rc):NM_104784.3       caaCCGCTGCTGCAACCGCTGCCtcg
```

### Precursor sequence and structure. Mature sequence in capital letters

```
ggcggcatcgaaacgaacacaaaacctgcggttgcgacagcggctgcggcaacgttggcggcgacgaaacgaacaacaacctgcggcagtgttaccgttgCCGCTGCCGCAACCGCAGCCgctgccgcc   
 (((((((.((....(....).....(((((((((((.(((((((.((((.((((...((.(((..................))).))..)))).)))).))))))).))))))))))).)).)))))))
```

---

## locus\_id: 180197

family\_id: 256

### **Targets:**

At5g48680(NM\_124245.2
): sterile alpha motif (SAM) domain-containing protein

Location in genome: in an IGR, 860 upstream of At3g04760, 2535 downstream of At3g04770

### Alignment between mature miRNA and predicted targets

```
        Extended mature miRNA:       cagcatgatcttgtcttcctctcttagg
        Target(rc):NM_124245.2       tatCATCATCTTGCCTTCCTCTCTTcct
```

### Precursor sequence and structure. Mature sequence in capital letters

```
gccagCATGATCTTGTCTTCCTCTCTTaggtttcatatatagttaataaatattttatatatttcttgttcttacaagattatatgatcatagcttagagagagagagagactaggtcatgctggt   
 (((((((((((((.((((..(((((((((((((((((((.........(((((.....)))))((((((....)))))).)))))))....)))))...)))))))..)))).)))))))))))))
```

---

## locus\_id: 233080

family\_id: 255

### **Targets:**

At5g43660(NM\_123732.1
): expressed protein

Location in genome: in an IGR, 887 upstream of At3g18150, 12988 downstream of At3g18100

### Alignment between mature miRNA and predicted targets

```
        Extended mature miRNA:       caagggaagcgtacttgtgcaaaatgcgg
        Target(rc):NM_123732.1       cgcGGGAAATGTACTTGTGCAAAATGtct
```

### Precursor sequence and structure. Mature sequence in capital letters

```
ggatctggtattttgctcaagtatggttttcttggcccatccacttctaatgtgttttgtaactaatgcaaaatgtgcattagaagtggattggtcaaGGGAAGCGTACTTGTGCAAAATGcggcagtcc   
 (((.((((((((((((.((((((((.((..(((((((.(((((((((((((((((((((((.....))))))))..))))))))))))))).)))))))..)).)))))))).)))))))))..))))))
```

---

## locus\_id: 168729

family\_id: 254

### **Targets:**

At3g59180(NM\_115780.1
): hypothetical protein

Location in genome: in an IGR, 2330 upstream of At2g34210, 1583 downstream of At2g34200

### Alignment between mature miRNA and predicted targets

```
        Extended mature miRNA:       caaaggagatttgcctcgcaatgcttc
        Target(rc):NM_115780.1       ctcAGGAGAATTGCCTTGCAATGCcaa
```

### Precursor sequence and structure. Mature sequence in capital letters

```
ttggtagaaagcattacagggcgaatcctctattggcagtggaagttgatgacccttatatgttattttctcatcattttcctctgccaaAGGAGATTTGCCTCGCAATGCttcactaa   
 .((((.(((.(((((.(..((((((((.(((.(((((((.(((((.((((((....((.....)).....)))))).))))).))))))).)))))))))))..).)))))))))))).
```

---

## locus\_id: 246688

family\_id: 253

### **Targets:**

At2g35860(NM\_179922.1
): beta-Ig-H3 domain-containing protein / fasciclin domain-containing protein

Location in genome: in an IGR, 33937 upstream of At3g32394, 39573 downstream of At3g32330

### Alignment between mature miRNA and predicted targets

```
        Extended mature miRNA:       cggcgggagctgggacaggagctgcg
        Target(rc):NM_179922.1       gtcCGGGAGCTGGAGCAGGAGCTagg
```

### Precursor sequence and structure. Mature sequence in capital letters

```
ttcggaagttcttgcatcctgtaagtcgagtcggtcgaggaaagatcggatgacttcgtccagattggttcctcgtggagttgtctctccgagttcggCGGGAGCTGGGACAGGAGCTgcggttgga   
 .(((..((((((((..((((((..((((((((((.(((((((.(((((((((....))))).))))..))))))).((((....)))))))).))))))....)).)))))))))))).))).....
```

---

## locus\_id: 124666

family\_id: 252

### **Targets:**

At4g38390(NM\_120001.2
): expressed protein

Location in genome: in an IGR, 2870 upstream of At2g21880, 4938 downstream of At2g21900

### Alignment between mature miRNA and predicted targets

```
        Extended mature miRNA:       gtcggcatagtacaagttggtcgacta
        Target(rc):NM_120001.2       gcaGGCATAGTACAAGTTGGACAAtct
```

### Precursor sequence and structure. Mature sequence in capital letters

```
gatttgtagtccacaaacttgtattatgccgatttgtaagcagtttgtcGGCATAGTACAAGTTGGTCGActacaaatc   
 (((((((((((.((.(((((((((((((((((((((....)))...)))))))))))))))))).)).)))))))))))
```

---

## locus\_id: 113736

family\_id: 251

### **Targets:**

At4g12640(NM\_117334.3
): RNA recognition motif (RRM)-containing protein

Location in genome: in an IGR, 10622 upstream of At2g07724, 14947 downstream of At2g07734

### Alignment between mature miRNA and predicted targets

```
        Extended mature miRNA:       ctctggacccggaatttgtgggtgca
        Target(rc):NM_117334.3       tgtTGGACCCGGAATACGTGGGTttc
```

### Precursor sequence and structure. Mature sequence in capital letters

```
ccgcctgctcTGGACCCGGAATTTGTGGGTgcaaggatgcaagtcttacgggaccgcattcgcggcctgcaacaaaggatttctggtctcctgcaggagcgg   
 ((((((((...(((((.((((((((..(((((..((((((..((((....)))).)))))))).)))..))......)))))).)))))...))))..))))
```

---

## locus\_id: 233056

family\_id: 250

### **Targets:**

At1g11220(NM\_100994.2
): expressed protein

Location in genome: in an IGR, 3417 upstream of At3g18150, 10460 downstream of At3g18100

### Alignment between mature miRNA and predicted targets

```
        Extended mature miRNA:       ttgaaccgtcgcggaggagttaggctc
        Target(rc):NM_100994.2       accAACCGTCGCGGAGGAGCTTGGgtg
```

### Precursor sequence and structure. Mature sequence in capital letters

```
ggctgagactccttggaagcttttagagacggcaacaatggcggctacgaaagctctggaatttgttttgccgacgggcttcttcatccagccgtcattgAACCGTCGCGGAGGAGTTAGGct   
 ((((..((((((((.(...........(((((...(((((((((((..(((((((((((((......)).)))..)))))..)))....)))))))))))..))))).).)))))))).))))
```

---

## locus\_id: 199777

family\_id: 249

### **Targets:**

At4g34990(NM\_119665.2
): myb family transcription factor (MYB32)

Location in genome: in an IGR, 21796 upstream of At3g29790, 66719 downstream of At3g30150

### Alignment between mature miRNA and predicted targets

```
        Extended mature miRNA:       ggggagacggtgggagaaagagattt
        Target(rc):NM_119665.2       ggaGAGACGGTTTGAGAAAGAGAgag
```

### Precursor sequence and structure. Mature sequence in capital letters

```
gggGAGACGGTGGGAGAAAGAGAtttggttagcggcggagctggctggcattctcttcttctttgatcttcgc   
 ..((((((((.(((((((.((((.(..((((((......))))))..)...)))))))))))))).)))))..
```

---

## locus\_id: 118860

family\_id: 248

### **Targets:**

At3g05210(NM\_111394.3
): nucleotide repair protein, putative

Location in genome: in an IGR, 3652 upstream of At2g14540, 18703 downstream of At2g14570

### Alignment between mature miRNA and predicted targets

```
        Extended mature miRNA:       agttggaagagagatggtggcgtctt
        Target(rc):NM_111394.3       ggcTTGAAGAGAGATGGAGGCGTgga
```

### Precursor sequence and structure. Mature sequence in capital letters

```
ggaagtTGGAAGAGAGATGGTGGCGTcttggttcctctcagtggcgtgttggccgagaagattagctcatacaccaggagttattcgagtcgtcatcttcttctctttgtttcc   
 (((((...(((((((((.(((((((.(((((.....(((..(((.((((.(((..(.....)..))).)))).))).)))....))))).))))))).)).))))))).)))))
```

---

## locus\_id: 200473

family\_id: 247

### **Targets:**

At1g45165(NM\_202244.1
): Expressed protein

Location in genome: in an IGR, 20525 upstream of At3g30390, 111935 downstream of At3g30470

12 homologs in brassica

### Alignment between mature miRNA and predicted targets

```
        Extended mature miRNA:       ccgacggatccggatccggatgcgga
        Target(rc):NM_202244.1       gttACGGATACGGATCCGGATACctc
```

### Precursor sequence and structure. Mature sequence in capital letters

```
gttatccgtatccggaaccgatccgaaaaaacgattatccgagatttccggatccagatacggataacgaaattacggatccgACGGATCCGGATCCGGATGCggataat   
 ((((((((((((((((.((((((((......((...(((((.((((((...((((......))))...)))))).))))).)).))))).))).))))))))))))))))
```

---

## locus\_id: 250814

family\_id: 246

### **Targets:**

At5g52840(NM\_124662.2
): NADH-ubiquinone oxidoreductase-related

Location in genome: in an IGR, 5044 upstream of At3g43580, 23164 downstream of At3g43570

### Alignment between mature miRNA and predicted targets

```
        Extended mature miRNA:       gcctgaagcagctgggatctctgctg
        Target(rc):NM_124662.2       gggTGTAGCAGCTGGGATCTCTGttt
```

### Precursor sequence and structure. Mature sequence in capital letters

```
ggttgggccTGAAGCAGCTGGGATCTCTGctgggtgaactggcagatcctgagatggactcatggaagggggccggtgagctacagaaagatctgggtcagcctgttgacatgccaagct   
 ((((.(((.((.((((((((((((((...((((((..((((((...(((((((.....)))).))).....))))))..))).)))..))))))....))).)))))..)).))).))))
```

---

## locus\_id: 231926

family\_id: 245

### **Targets:**

At3g13380(NM\_112183.2
): leucine-rich repeat family protein / protein kinase family protein

Location in genome: in an IGR, 21613 upstream of At3g16650, 4035 downstream of At3g16570

### Alignment between mature miRNA and predicted targets

```
        Extended mature miRNA:       cttaccattggaaagtgctctaaggc
        Target(rc):NM_112183.2       tgaACCATTGGAAAATGCTCTAAccg
```

### Precursor sequence and structure. Mature sequence in capital letters

```
atgggccttacagcaccgccaatggtaagaagatcttccaaggtttctacactgtgctatattcatggacatcatgtacgtgctcttACCATTGGAAAGTGCTCTAAggcccat   
 ((((((((((.(((((..((((((((((((.(((((....)))))..((((..(((((((....)))).)))..)))).....))))))))))))...))))).))))))))))
```

---

## locus\_id: 201991

family\_id: 244

### **Targets:**

At5g28140(NM\_122697.1
): hypothetical protein

Location in genome: in an IGR, 500 upstream of At3g31540, 61608 downstream of At3g31910

### Alignment between mature miRNA and predicted targets

```
        Extended mature miRNA:       agtttgtttagttggctcggttcctacg
        Target(rc):NM_122697.1       aaaTTGCTTGGTTGGCTCGGTTCCTtgc
```

### Precursor sequence and structure. Mature sequence in capital letters

```
gtcttggaacgacttccgagccaaatatacccgacagatatttccgaggcgttctcggagtTTGTTTAGTTGGCTCGGTTCCTacgagac   
 ((((((....((...(((((((((...((...(((((((...((((((.....))))))))))))))).))))))))).))...))))))
```

---

## locus\_id: 131901

family\_id: 243

### **Targets:**

At5g26230(NM\_122524.2
): expressed protein

Location in genome: Contained by At2g31590 (NM\_128716: . hypothetical protein) in an intron

### Alignment between mature miRNA and predicted targets

```
        Extended mature miRNA:       tttccggcgacggagctccggcgagg
        Target(rc):NM_122524.2       tcaCCGACGACGGAGCTGCGGCGgtt
```

### Precursor sequence and structure. Mature sequence in capital letters

```
gcgaaggtttCCGGCGACGGAGCTCCGGCGagggttttccggcaagggagttccggtggtgggtttcggcgatggaattccggc   
 .((..((..((((.((.(((((((((.((..((.....)).))...))))))))).)).))))..))..))..((....))...
```

---

## locus\_id: 131897

family\_id: 242

### **Targets:**

At2g27990(NM\_128358.2
): homeodomain-containing protein

Location in genome: Contained by At2g31590 (NM\_128716: . hypothetical protein) in an intron

### Alignment between mature miRNA and predicted targets

```
        Extended mature miRNA:       aagggagttccggtggtgggtttcgg
        Target(rc):NM_128358.2       gtcGGAGCTCCGGTGGTGAGTTTaga
```

### Precursor sequence and structure. Mature sequence in capital letters

```
ccggcgaaggtttccggcgacggagctccggcgagggttttccggcaagGGAGTTCCGGTGGTGGGTTTcggcgatgg   
 .((.(((((...((((.((.(((((((((.((..((.....)).))...))))))))).)).))))))))).))....
```

---

## locus\_id: 114143

family\_id: 241

### **Targets:**

At1g51730(NM\_104051.3
): RWD domain-containing protein

Location in genome: in an IGR, 10512 upstream of At2g07690, 32299 downstream of At2g07740

### Alignment between mature miRNA and predicted targets

```
        Extended mature miRNA:       caccaagttggctcgctcaggtggat
        Target(rc):NM_104051.3       catCAACTTGGCTCGCTCAAGTGcaa
```

### Precursor sequence and structure. Mature sequence in capital letters

```
agttcggatgtgaggagtgggacgaaacttggtcaccaagtcggctttgggacgaaacttggtcacCAAGTTGGCTCGCTCAGGTGgattcggct   
 (((.(((((.((..(((((((....((((((((.(((((((((.........)))..)))))).))))))))..)))))))...)).))))))))
```

---

## locus\_id: 140324

family\_id: 240

### **Targets:**

At2g29260(NM\_128482.2
): tropinone reductase, putative / tropine dehydrogenase, putative

Location in genome: Contained by At2g44735 (NM\_180092: . expressed protein) in an intron on the reverse strand

### Alignment between mature miRNA and predicted targets

```
        Extended mature miRNA:       gagcggttcgctaagtccaccaacgc
        Target(rc):NM_128482.2       tctCGGTTCGCCAAGTCGACCAAgag
```

### Precursor sequence and structure. Mature sequence in capital letters

```
ggcaagagCGGTTCGCTAAGTCCACCAAcgccacctaccggtcccgtataacacggctcacgttagcctccaaccatgatgactacggtggcttggtggcttcatcagccgctttttct   
 ((.((((((((((....(((.(((((((.((((((....((((.(((.......((((......)))).......)))..))))..))))))))))))))))....)))))))))).))
```

---

## locus\_id: 122433

family\_id: 239

### **Targets:**

At1g60040(NM\_104696.1
): MADS-box family protein

Location in genome: in an IGR, 15392 upstream of At2g18480, 4528 downstream of At2g18510

### Alignment between mature miRNA and predicted targets

```
        Extended mature miRNA:       ttctgaagtggtggtagtcgagaaaa
        Target(rc):NM_104696.1       gtaTGAAGTGGTGGTAGTCTTGAggc
```

### Precursor sequence and structure. Mature sequence in capital letters

```
atcgttcttcTGAAGTGGTGGTAGTCGAGAaaacgtatttatatgtaggatttcatatatatacgttttctcgattacttcttcagtagaatgat   
 ((((((((.((((((....(((((((((((((((((((.((((((........)))))).))))))))))))))))))).)))))).))))))))
```

---

## locus\_id: 170335

family\_id: 238

### **Targets:**

At3g45490(NM\_114418.1
): hypothetical protein

Location in genome: in an IGR, 9019 upstream of At2g36720, 592 downstream of At2g36695

### Alignment between mature miRNA and predicted targets

```
        Extended mature miRNA:       agttgagaaccacaaccgtaagccgtc
        Target(rc):NM_114418.1       caaTCACAACCACAACCGTAAGCCcaa
```

### Precursor sequence and structure. Mature sequence in capital letters

```
tggtcgagtTGAGAACCACAACCGTAAGCCgtcttcgtaccgacggtccccttggtgtgtgattgagctgaggcggttgcgaacgttcttgtcgacca   
 .((((((..((((((((.(((((((.(((((((..((((((((.(....).)))))))).)).)).)))...))))))).)...))))))))))))).
```

---

## locus\_id: 227265

family\_id: 237

### **Targets:**

At3g27720(NM\_113688.1
): zinc finger protein-related

Location in genome: in an IGR, 10831 upstream of At3g09922, 3548 downstream of At3g09900

### Alignment between mature miRNA and predicted targets

```
        Extended mature miRNA:       tagtcgtagtcttcctcttcctcttc
        Target(rc):NM_113688.1       tttTCGTCTTCTTCCTCTTCCTCgtc
```

### Precursor sequence and structure. Mature sequence in capital letters

```
gtcctagTCGTAGTCTTCCTCTTCCTCttcttttccagggacgacattgatttcttactcttcaaaagaagaagaagaagaagacaacacgttagggc   
 (((((((.(((.((((((.(((((.(((((((((..((((..((........))...))))..))))))))).))))).))))))...))))))))))
```

---

## locus\_id: 120024

family\_id: 236

### **Targets:**

At4g30160(NM\_119162.2
): villin, putative

Location in genome: in an IGR, 6325 upstream of At2g15390, 6568 downstream of At2g15420

### Alignment between mature miRNA and predicted targets

```
        Extended mature miRNA:       ctgggccgagcagctttcatctgggcc
        Target(rc):NM_119162.2       ttaGGCGGAGCAGCTTTCATCAGGcct
```

### Precursor sequence and structure. Mature sequence in capital letters

```
gtcgggctgGGCCGAGCAGCTTTCATCTGGgccgacttgatgttgttgggccgagcagctctcatttgggccgagccggac   
 (((.((((.(((((((.((((....((.((.(((((........))))).))))..))))))).....)))).)))).)))
```

---

## locus\_id: 125185

family\_id: 235

### **Targets:**

At1g80050(NM\_106654.2
): adenine phosphoribosyltransferase 2 (APT2)

Location in genome: in an IGR, 11288 upstream of At2g22470, 3617 downstream of At2g22490

### Alignment between mature miRNA and predicted targets

```
        Extended mature miRNA:       ttttggaagctgagagagtcccatac
        Target(rc):NM_106654.2       taaTGGAAGCAGAGAGTGTCCCAcca
```

### Precursor sequence and structure. Mature sequence in capital letters

```
gttttTGGAAGCTGAGAGAGTCCCAtacgtggcgcatcgtggttggctcttttagttttaacaaaagc   
 ((((((((((((((((((((((...((((........))))...)))))))))))))))..)))))))
```

---

## locus\_id: 148184

family\_id: 234

### **Targets:**

At5g47950(NM\_124169.2
): transferase family protein

Location in genome: in an IGR, 9046 upstream of At2g07672, 546 downstream of At2g07749

### Alignment between mature miRNA and predicted targets

```
        Extended mature miRNA:       aagagatcccggacagggaagtcaga
        Target(rc):NM_124169.2       tcaAGATCTCGGACAGGGAAGTTttg
```

### Precursor sequence and structure. Mature sequence in capital letters

```
ttcagtgaagAGATCCCGGACAGGGAAGTCagaagggcagttactcttcggacctagcggccctgctttagctgcacttggtggttccccgctcgggattttatactgga   
 (((((((..(((((((((..(.(((((.(((..((((((((((......((.((....))))......))))))).)))..)))))))).)..))))))))).)))))))
```

---

## locus\_id: 253050

family\_id: 233

### **Targets:**

At2g47240(NM\_130292.2
): long-chain-fatty-acid--CoA ligase family protein / long-chain acyl-CoA synthetase family protein

Location in genome: in an IGR, 17044 upstream of At3g45230, 13756 downstream of At3g45170

### Alignment between mature miRNA and predicted targets

```
        Extended mature miRNA:       ttcgtgagtttagggttcttgaatca
        Target(rc):NM_130292.2       tcaGTGAGTTCAGGGTTCTTGTAata
```

### Precursor sequence and structure. Mature sequence in capital letters

```
ttgtttcGTGAGTTTAGGGTTCTTGAAtcaaagaatcgataaagtaatcgccaatgaatcttcgattcgagaaccctaagctcacgaaacga   
 (((((((((((((((((((((((((((((.((((.(((.....(....).....))).)))).)))))))))))))))))))))))))))))
```

---

## locus\_id: 152436

family\_id: 232

### **Targets:**

At2g45660(NM\_130128.2
): MADS-box protein (AGL20)

Location in genome: in an IGR, 19577 upstream of At2g12610, 3529 downstream of At2g12550

### Alignment between mature miRNA and predicted targets

```
        Extended mature miRNA:       tctaggtggaagctttcgaggctgca
        Target(rc):NM_130128.2       accAGGAGGAAGCTTTCGAGGATaaa
```

### Precursor sequence and structure. Mature sequence in capital letters

```
cagctgttggccttgctgggacctctaaaaggcatggatctcgggatcatgtctAGGTGGAAGCTTTCGAGGCTgcagttg   
 (((((((.(((((((..(((.(.((((..(((((((.(((....))))))))))...)))).)))).))))))))))))))
```

---

## locus\_id: 115171

family\_id: 231

### **Targets:**

At2g42310(NM\_129793.4
): expressed protein

Location in genome: in an IGR, 114984 upstream of At2g10608, 15422 downstream of At2g10850

### Alignment between mature miRNA and predicted targets

```
        Extended mature miRNA:       aacattagagagtggaggcaattgagc
        Target(rc):NM_129793.4       agaAGTAGAGAGTGAAGGCAATTGcgt
```

### Precursor sequence and structure. Mature sequence in capital letters

```
ttccttaacATTAGAGAGTGGAGGCAATTGagcactatcgataggtgccaatttccatactctctgggcgaaggga   
 ((((((....((((((((((..((.(((((.(((((.......)))))))))).)).))))))))))...))))))
```

---

## locus\_id: 128260

family\_id: 230

### **Targets:**

At1g32640(NM\_102998.2
): basic helix-loop-helix (bHLH) protein (RAP-1)

Location in genome: in an IGR, 9203 upstream of At2g26190, 7524 downstream of At2g26240

### Alignment between mature miRNA and predicted targets

```
        Extended mature miRNA:       aagcttcttcgagctggtgcttgaacg
        Target(rc):NM_102998.2       tcaCTTCCTCGAGCTGGTTCTTGAttt
```

### Precursor sequence and structure. Mature sequence in capital letters

```
tcaacttgttcatgcaccttcttgagaagcatagctttttcattattttctaagataaattagctaagCTTCTTCGAGCTGGTGCTTGAacgagtcga   
 ((.(((((((((.(((((..((((((((((.(((((......((((((....))))))...))))).))))).)))))..))))).))))))))).))
```

---

## locus\_id: 141100

family\_id: 229

### **Targets:**

At5g65750(NM\_125972.3
): 2-oxoglutarate dehydrogenase E1 component, putative / oxoglutarate decarboxylase, putative / alpha-ketoglutaric dehydrogenase, putative

Location in genome: in an IGR, 896 upstream of At2g45680, 420 downstream of At2g45690

### Alignment between mature miRNA and predicted targets

```
        Extended mature miRNA:       aagagagtagggtccagctcaggccc
        Target(rc):NM_125972.3       cgaAGAGTTGGGTCCATCTCAGGgat
```

### Precursor sequence and structure. Mature sequence in capital letters

```
gaatggtggggtcgaatagttgggcctagccctcaacaatgtgtggaactgaagAGAGTAGGGTCCAGCTCAGGcccacattc   
 (((((...(((((....((((((((((.((.(((..((..((.....))))..))).)).))))))))))..))))).)))))
```

---

## locus\_id: 203204

family\_id: 228

### **Targets:**

At4g07932(NM\_148273.1
): hypothetical protein

Location in genome: in an IGR, 27105 upstream of At3g32410, 31540 downstream of At3g32902

### Alignment between mature miRNA and predicted targets

```
        Extended mature miRNA:       atcagtttggccgagatgggcgtgtgctcgag
        Target(rc):NM_148273.1       gtgAGTTTGGCCGAGATGGACGTGTGCTCaac
```

### Precursor sequence and structure. Mature sequence in capital letters

```
gcccgcaatgtgcccgtagtgagcccgcagtatgcccgtggtatgctcgcagtgtgcccgtagtaggcagtgtgagcgacctgcggtggcatcAGTTTGGCCGAGATGGGCGTGTGCTCgagc   
 ((.((..(..(((((((..((.(((.((.(.(((((((((((.((((((((...((((.......)))).))))))))))).)))..)))))).))..)))))..)))))))..)...)).))
```

---

## locus\_id: 151546

family\_id: 227

### **Targets:**

At3g03680(NM\_111239.2
): C2 domain-containing protein

Location in genome: in an IGR, 6304 upstream of At2g11620, 30546 downstream of At2g11520

### Alignment between mature miRNA and predicted targets

```
        Extended mature miRNA:       catcgaccctaacctctctccttagg
        Target(rc):NM_111239.2       tccCGACCCTAACATCTCTCCCTtgc
```

### Precursor sequence and structure. Mature sequence in capital letters

```
tgcggcgcatCGACCCTAACCTCTCTCCTTaggccaccaaagccctgggccaccagaggttagggtcggtgcgctgca   
 .(((((((((((((((((((((((..(((..(((.......)))..))).....))))))))))))))))))))))).
```

---

## locus\_id: 262701

family\_id: 226

### **Targets:**

At2g23740(NM\_127937.1
): zinc finger (C2H2 type) family protein

Location in genome: in an IGR, 8132 upstream of At3g60530, 8539 downstream of At3g60500

### Alignment between mature miRNA and predicted targets

```
        Extended mature miRNA:       cttttctgacgataacattggttgcgaa
        Target(rc):NM_127937.1       ccaTTCTGACAACAACATTGGTTGCatt
```

### Precursor sequence and structure. Mature sequence in capital letters

```
gcttTTCTGACGATAACATTGGTTGCgaaaccggttggaaactagtcgaaaatgatcgtcggatcctcgtcgaaaatagtttccgactattttctaacgaatattgttgtcaagc   
 (((....(((((((((.(((.((((.((((..((((((((((((.((((....((((....))))....))))...))))))))))))..)))))))).))).))))))))))))
```

---

## locus\_id: 252624

family\_id: 225

### **Targets:**

At5g56040(NM\_124986.3
): leucine-rich repeat protein kinase, putative

Location in genome: in an IGR, 10199 upstream of At3g44730, 2326 downstream of At3g44720

### Alignment between mature miRNA and predicted targets

```
        Extended mature miRNA:       agccatgcattttaggatctgccgga
        Target(rc):NM_124986.3       tctCATGCATTATAGGATCTGCCcgc
```

### Precursor sequence and structure. Mature sequence in capital letters

```
ggagagttaggtggaagagatttcatatatggtggtaggtggcgtcgaagggagccatcagcCATGCATTTTAGGATCTGCCggattgtcc   
 (((.((((.(((.....(((((..(...((((((((.((((((..(....)..)))))).))))).)))..)..)))))))).)))).)))
```

---

## locus\_id: 233079

family\_id: 224

### **Targets:**

At5g08490(NM\_120934.1
): pentatricopeptide (PPR) repeat-containing protein

Location in genome: in an IGR, 959 upstream of At3g18150, 12917 downstream of At3g18100

### Alignment between mature miRNA and predicted targets

```
        Extended mature miRNA:       ggttttcttggcccatccacttctaatg
        Target(rc):NM_120934.1       acaTTCCTTTGCCCATCCACTTCTAgcc
```

### Precursor sequence and structure. Mature sequence in capital letters

```
caagtatggtTTTCTTGGCCCATCCACTTCTAatgtgttttgtaactaatgcaaaatgtgcattagaagtggattggtcaagggaagcgtacttg   
 ((((((((.((..(((((((.(((((((((((((((((((((((.....))))))))..))))))))))))))).)))))))..)).))))))))
```

---

## locus\_id: 162648

family\_id: 223

### **Targets:**

At3g54190(NM\_115279.2
): expressed protein

Location in genome: in an IGR, 12195 upstream of At2g25060, 16381 downstream of At2g25010

### Alignment between mature miRNA and predicted targets

```
        Extended mature miRNA:       agaccggtgaagcaaatgattgagaa
        Target(rc):NM_115279.2       gttTCGGTGAAGCAAATGATTGAagg
```

### Precursor sequence and structure. Mature sequence in capital letters

```
agctgtggatctccttgctcagcttggctttgaccccaacaatggggcaagaCCGGTGAAGCAAATGATTGAgaagct   
 ((((.(.((((...(((((..(((.((..(((.(((((....))))))))..)))))..)))))..)))).)..))))
```

---

## locus\_id: 178184

family\_id: 222

### **Targets:**

At3g56750(NM\_115534.2
): expressed protein

Location in genome: Contained by At3g01540 (NM\_202477: . DEAD box RNA helicase (DRH1)) in an intron

1 homologs in brassica

### Alignment between mature miRNA and predicted targets

```
        Extended mature miRNA:       gacagaagaagaggcgtcctcctgct
        Target(rc):NM_115534.2       agaAGAAGAAGAAGAGTCCTCCTcga
```

### Precursor sequence and structure. Mature sequence in capital letters

```
ggtccagacAGAAGAAGAGGCGTCCTCCTgctgcaggtttcatcagtaaccttttgccacctgcaaatgggggatggtgcttcttctgacatgagcc   
 ((..((..(((((((((.(.((((((((...(((((((..((..((....))..))..)))))))...)))))))).).)))))))))...))..))
```

---

## locus\_id: 183390

family\_id: 221

### **Targets:**

At1g35230(NM\_103221.2
): arabinogalactan-protein (AGP5)

Location in genome: in an IGR, 1709 upstream of At3g09910, 145 downstream of At3g09920

### Alignment between mature miRNA and predicted targets

```
        Extended mature miRNA:       gaagaggaagaggaagactacgactag
        Target(rc):NM_103221.2       agcGAGGAAGAGGAAAACGACGACgga
```

### Precursor sequence and structure. Mature sequence in capital letters

```
gccctaacgtgttgtcttcttcttcttcttcttttgaagagtaagaaatcaatgtcgtccctggaaaagaaGAGGAAGAGGAAGACTACGACtaggac   
 ..((((...(((.((((((((((((((((((((((..((.(...((........))..).))..)))))))))))))))))))))).)))..))))..
```

---

## locus\_id: 168734

family\_id: 220

### **Targets:**

At3g50270(NM\_114887.2
): transferase family protein

Location in genome: in an IGR, 851 upstream of At2g34210, 3063 downstream of At2g34200

1 homologs in brassica

### Alignment between mature miRNA and predicted targets

```
        Extended mature miRNA:       atgtttgtggtgagctctctgccaaa
        Target(rc):NM_114887.2       ggcTTGGTGGTGACCTCTCTGCCtct
```

### Precursor sequence and structure. Mature sequence in capital letters

```
ttggcagagatctattacttcattcttgcatcatatgcataaatgTTTGTGGTGAGCTCTCTGCCaa   
 .(((((((((.(((((((..((((..(((((...)))))..))))...))))).)).))))))))).
```

---

## locus\_id: 112267

family\_id: 219

### **Targets:**

At5g55230(NM\_124905.2
): microtubule associated protein (MAP65/ASE1) family protein

Location in genome: in an IGR, 3852 upstream of At2g07776, 520 downstream of At2g07777

### Alignment between mature miRNA and predicted targets

```
        Extended mature miRNA:       cttgagcctccttgctcaaacctgcc
        Target(rc):NM_124905.2       tagGAACCTCATTGCTCAAACCTcca
```

### Precursor sequence and structure. Mature sequence in capital letters

```
ggggaaggagtgttatgaaagggaggaagaggaaaagctactcttGAGCCTCCTTGCTCAAACCTgccttcttc   
 ((((((((((.(((.(((.((((((((((((.........)))))...)))))))..)))))))).))))))))
```

---

## locus\_id: 211873

family\_id: 218

### **Targets:**

At5g25790(NM\_122480.1
): tesmin/TSO1-like CXC domain-containing protein

Location in genome: in an IGR, 837 upstream of At3g48250, 227 downstream of At3g48260

### Alignment between mature miRNA and predicted targets

```
        Extended mature miRNA:       tattcacttgattcttctgagctagc
        Target(rc):NM_122480.1       accTCACTCGATTCTTCTGAACTtgg
```

### Precursor sequence and structure. Mature sequence in capital letters

```
gacgtaactagctcagaagagtctgggagaagaaaagaacgcggtgatgataggtcacatcactgtgttcttatTCACTTGATTCTTCTGAGCTagcctcgtc   
 ((((...((((((((((((((((.((..(((...(((((((((((((((........))))))))))))))).))).)).))))))))))))))))...))))
```

---

## locus\_id: 201499

family\_id: 217

### **Targets:**

At4g24320(NM\_118565.1
): hypothetical protein

Location in genome: in an IGR, 22759 upstream of At3g30845, 49879 downstream of At3g31330

2 homologs in brassica

### Alignment between mature miRNA and predicted targets

```
        Extended mature miRNA:       atggttgcagagctcttgcaagctgat
        Target(rc):NM_118565.1       tttGTTGCAGAGCTCTCGCTAGCTttg
```

### Precursor sequence and structure. Mature sequence in capital letters

```
ttagcttgctaatagctcgtaaacaatcatcttcagtcacagcttcacgatctttcttccttctctgacgaagatgGTTGCAGAGCTCTTGCAAGCTga   
 (((((((((.((.(((((.....(((((((((((.((((.((......((....))......)).)))))))))))))))..))))).)))))))))))
```

---

## locus\_id: 131623

family\_id: 216

### **Targets:**

At2g24740(NM\_128034.2
): SET domain-containing protein (SUVH8)

Location in genome: Contained by At2g31190 (NM\_128675: . expressed protein) in an intron

### Alignment between mature miRNA and predicted targets

```
        Extended mature miRNA:       tgtgcctgctttcctcagcagcttcaa
        Target(rc):NM_128034.2       ttgGCCTGGTTTCCTCAGCAGTTTgaa
```

### Precursor sequence and structure. Mature sequence in capital letters

```
ttcttgaaatggctggtctggggaattttgctaaggtatagatatctttcttagaacatctgtGCCTGCTTTCCTCAGCAGCTTcaataggag   
 .(((((....(((((..((((((((....((..((((((((((.(((.....)))..)))))))))))).)))))))))))))....))))).
```

---

## locus\_id: 239755

family\_id: 215

### **Targets:**

At5g18730(NM\_121878.1
): hypothetical protein

Location in genome: in an IGR, 2796 upstream of At3g26820, 7673 downstream of At3g26810

### Alignment between mature miRNA and predicted targets

```
        Extended mature miRNA:       caaagaaacaggcaagtcatccttgg
        Target(rc):NM_121878.1       aacAGAAACAGGCAAGTCTTCCTcaa
```

### Precursor sequence and structure. Mature sequence in capital letters

```
gccaaggagactgcctgatgtcttttgatgagtaaaatggtcattgcttgatataacttatagtcacttgtcaaacatgacactaacccattttactcaaAGAAACAGGCAAGTCATCCTtggc   
 (((((((((((((((((...((((....((((((((((((((((.(.((((((..((.....))....)))))).))))).......))))))))))))))))..))))).)))).))))))))
```

---

## locus\_id: 254842

family\_id: 214

### **Targets:**

At3g13830(NM\_112236.1
): F-box family protein

Location in genome: in an IGR, 8552 upstream of At3g48070, 8653 downstream of At3g48040

### Alignment between mature miRNA and predicted targets

```
        Extended mature miRNA:       tttcttttaggtcgagcttcattggaa
        Target(rc):NM_112236.1       cgcCATTTAGGTCGAGCTTCATTGaac
```

### Precursor sequence and structure. Mature sequence in capital letters

```
atctattttCTTTTAGGTCGAGCTTCATTGgaaccaaatggtggctcatccatctattactaaagctatacagcaggtggacaagccatcatcatgttcctgtgaagctcgatctaaaagactacagat   
 ((((.(..((((((((((((((((((((.(((((...(((((((((..(((((((.........(((....))))))))))..)))))))))...))))).))))))))))))))))))))..).))))
```

---

## locus\_id: 123411

family\_id: 213

### **Targets:**

At5g65925(NM\_148165.2
): expressed protein

Location in genome: Contained by At2g20010 (NM\_127559: . expressed protein) in an intron

### Alignment between mature miRNA and predicted targets

```
        Extended mature miRNA:       agggatgaagaaacgaatcggagacgg
        Target(rc):NM_148165.2       catGATCAAGAAACGAACCGGAGAttt
```

### Precursor sequence and structure. Mature sequence in capital letters

```
tctccgtctccgtcgcttcatagatctctgacttctacagctgcgagtaaggtgaagaaggcgttaggGATGAAGAAACGAATCGGAGAcggaga   
 (((((((((((((((.(((.(..(((((((((((((.((.((.......)).)).))))...))))))))).).))).)))..))))))))))))
```

---

## locus\_id: 259032

family\_id: 212

### **Targets:**

At5g51080(NM\_124487.2
): RNase H domain-containing protein

Location in genome: in an IGR, 1533 upstream of At3g54840, 5149 downstream of At3g54826

### Alignment between mature miRNA and predicted targets

```
        Extended mature miRNA:       atgcagttgtagcgccagagagaaga
        Target(rc):NM_124487.2       gtaCAGCTGCAGCGCCAGAGAGAccg
```

### Precursor sequence and structure. Mature sequence in capital letters

```
ggtcttctcttattggtatacattgttagatctcagattatgCAGTTGTAGCGCCAGAGAGAagatc   
 (((((((((((..((((.((((((((..((((...))))..)))).))))..)))))))))))))))
```

---

## locus\_id: 222700

family\_id: 211

### **Targets:**

At5g43760(NM\_123743.2
): beta-ketoacyl-CoA synthase, putative

Location in genome: in an IGR, 3239 upstream of At3g02680, 4966 downstream of At3g02650

### Alignment between mature miRNA and predicted targets

```
        Extended mature miRNA:       tgtggagaggaagcaagaggatgtgct
        Target(rc):NM_123743.2       cgaGGAGAGGAAGGAGGAGGATGTaga
```

### Precursor sequence and structure. Mature sequence in capital letters

```
tgtGGAGAGGAAGCAAGAGGATGTgcttggttgtggaaatatagggcccttaaaatatattcatcgtattcactcacataacaaaaattccacaagtaagcacatcatcttgcttcctccaca   
 .(((((..((((((((((.((((((((((.((((((((....((....))...(((((.......))))).................)))))))).)))))))))).))))))))))))))).
```

---

## locus\_id: 144789

family\_id: 210

### **Targets:**

At3g24517(NM\_148748.1
): hypothetical protein

Location in genome: in an IGR, 39850 upstream of At2g03980, 30779 downstream of At2g03890

### Alignment between mature miRNA and predicted targets

```
        Extended mature miRNA:       gtagattgaagaagaacggcattatgatctcg
        Target(rc):NM_148748.1       aagGATTGAAGAAAATCGGCATTATGATCacg
```

### Precursor sequence and structure. Mature sequence in capital letters

```
agtaGATTGAAGAAGAACGGCATTATGATCtcgcctctacggagcgaagattattgtagcttcttcaatccgct   
 ((..(((((((((((....(((..((((((((((.((....))))).))))))))))..)))))))))))..))
```

---

## locus\_id: 197522

family\_id: 209

### **Targets:**

At4g27760(NM\_118915.3
): oxidoreductase, forever young (FEY3)

Location in genome: in an IGR, 41617 upstream of At3g28280, 3359 downstream of At3g28330

### Alignment between mature miRNA and predicted targets

```
        Extended mature miRNA:       cgactggtggagtcagtggcaacaca
        Target(rc):NM_118915.3       ccgCTGGTGGAGCCAGTGACAACgca
```

### Precursor sequence and structure. Mature sequence in capital letters

```
gtcgaatgtgtgtcatcatctcctttagccggtttatagagttagccattgactcgaCTGGTGGAGTCAGTGGCAACacaattgat   
 (((((.((((((((((...((((....(((((((....(((((((...))))))))))))))))))...))))).))))).)))))
```

---

## locus\_id: 213883

family\_id: 208

### **Targets:**

At4g25450(NM\_118677.2
): ABC transporter family protein

Location in genome: in an IGR, 1366 upstream of At3g51320, 482 downstream of At3g51330

### Alignment between mature miRNA and predicted targets

```
        Extended mature miRNA:       atacttgttcgtcaagctccacattt
        Target(rc):NM_118677.2       gtgCTTGCTCATCAAGCTCCACAgaa
```

### Precursor sequence and structure. Mature sequence in capital letters

```
ctaacaagttttggagtttgaagaacaaatatgtgggtaaaaggcaaaaaggatgcagatcaataatcttttacctacataCTTGTTCGTCAAGCTCCACAttttggtag   
 (((.((((...((((((((((.((((((.(((((((((((((((.......(((....))).....))))))))))))))).)))))).))))))))))...)))).)))
```

---

## locus\_id: 134740

family\_id: 207

### **Targets:**

At3g52525(NM\_148870.2
): ovate protein, putative

Location in genome: in an IGR, 1938 upstream of At2g36020, 14 downstream of At2g36030

### Alignment between mature miRNA and predicted targets

```
        Extended mature miRNA:       tctgagtctttctcgacggcgacagc
        Target(rc):NM_148870.2       tcaGAATCTTTCTCCACGGCGACgga
```

### Precursor sequence and structure. Mature sequence in capital letters

```
ggatcgtctGAGTCTTTCTCGACGGCGACagcggcggcggagggtgttgccgaggaggaagaggtgtcagagaacc   
 ((.((.(((((.((((((((..((((((((.(..(....)..).))))))))..))))))))....))))))).))
```

---

## locus\_id: 117292

family\_id: 206

### **Targets:**

At2g01810(NM\_126242.1
): PHD finger family protein

Location in genome: in an IGR, 117367 upstream of At2g13126, 2133 downstream of At2g13350

### Alignment between mature miRNA and predicted targets

```
        Extended mature miRNA:       agaaaacgctttgggaaccgcttgtat
        Target(rc):NM_126242.1       tttAAACACTTTGAGAACCGCTTGggc
```

### Precursor sequence and structure. Mature sequence in capital letters

```
cgatcgagaAAACGCTTTGGGAACCGCTTGtatcgcctcgggagatagtcaaccaatttcaatgaagagtcatccagcatgtcccgagccagaatacaccggttgcccaaagcgacctctcgaacg   
 ((.((((((...((((((((((((((..((((((..(((((((.((.((........(((......))).......)))).)))))))...).))))).))))).)))))))))...)))))).))
```

---

## locus\_id: 162594

family\_id: 205

### **Targets:**

At4g03830(NM\_116621.1
): myosin heavy chain-related

Location in genome: in an IGR, 27830 upstream of At2g25060, 744 downstream of At2g25010

### Alignment between mature miRNA and predicted targets

```
        Extended mature miRNA:       atagcttcaggtccttcaatggaggtgt
        Target(rc):NM_116621.1       tcgGCTTCAGCTCCTTCAATGGAGGgat
```

### Precursor sequence and structure. Mature sequence in capital letters

```
ggaagcataGCTTCAGGTCCTTCAATGGAGGtgtggtgaaacagagtttcaagaggggggtatttttgttgaaggaccacacctccattgaaggatatggagctatgcttcc   
 (((((((((((((((.(((((((((((((((((((((..((((((((..(......)..)...))))))).....))))))))))))))))))))).)))))))))))))))
```

---

## locus\_id: 145225

family\_id: 204

### **Targets:**

At3g51950(NM\_115054.2
): zinc finger (CCCH-type) family protein / RNA recognition motif (RRM)-containing protein

Location in genome: in an IGR, 3876 upstream of At2g04220, 106940 downstream of At2g04039

### Alignment between mature miRNA and predicted targets

```
        Extended mature miRNA:       tcacctttcaggtttccattttcact
        Target(rc):NM_115054.2       aatCCTTTCAGGTCTCCATCTTCcaa
```

### Precursor sequence and structure. Mature sequence in capital letters

```
atccatctcaCCTTTCAGGTTTCCATTTTCactcctttataaaggagtgaaaatggtaaccggaaaagagatatggat   
 ((((((.((.(.((((.((((.(((((((((((((((....))))))))))))))).)))).)))).).)).))))))
```

---

## locus\_id: 169771

family\_id: 203

### **Targets:**

At2g36100(NM\_129169.1
): integral membrane family protein

Location in genome: in an IGR, 6937 upstream of At2g35790, 418 downstream of At2g35760

### Alignment between mature miRNA and predicted targets

```
        Extended mature miRNA:       gaggatagactcagccgcaaccgact
        Target(rc):NM_129169.1       cgcGATAGAATCAGCCACAACCGcgg
```

### Precursor sequence and structure. Mature sequence in capital letters

```
ggtggctttggtgatggctttgatgttgcttatgtccgtgttggtttctgccgaggaaacaccaaccatcggacagagGATAGACTCAGCCGCAACCGactttacc   
 (((((..(((((..(((((..((.(((.(((.((((((.((((((((((.....))))..))))))...)))))).)))...))))))))))..)))))..)))))
```

---

## locus\_id: 169601

family\_id: 202

### **Targets:**

At1g64030(NM\_105076.1
): serpin family protein / serine protease inhibitor family protein

Location in genome: in an IGR, 6354 upstream of At2g35605, 1727 downstream of At2g35585

### Alignment between mature miRNA and predicted targets

```
        Extended mature miRNA:       tcgatccaaacgccattcgctgccaat
        Target(rc):NM_105076.1       tcaATCCAAAGGCCATTCGCCGCCgtt
```

### Precursor sequence and structure. Mature sequence in capital letters

```
gtcttttcgATCCAAACGCCATTCGCTGCCaatatcgtcgggccgccactcggggtgctgtcggcgaggatggtggtgacgatcctggagaagac   
 ((((((((((((....((((((((((((.((.((((.(((((......))))))))).)).))))))..)))))).....))))...))))))))
```

---

## locus\_id: 138713

family\_id: 201

### **Targets:**

At2g32295(NM\_179857.1
): EXS family protein / ERD1/XPR1/SYG1 family protein

Location in genome: in an IGR, 266 upstream of At2g41860, 792 downstream of At2g41870

### Alignment between mature miRNA and predicted targets

```
        Extended mature miRNA:       aagaacttccttccatatctctctgtcat
        Target(rc):NM_179857.1       caaAACCACCTTCCATATCTCTCTGTgac
```

### Precursor sequence and structure. Mature sequence in capital letters

```
caagaagaaacagagagattagggagggaaggaatttgtgctttttcaaaaaaagAACTTCCTTCCATATCTCTCTGTcatttttg   
 ((((((...((((((((((...(((((((((...(((.((......))...)))...)))))))))..))))))))))..))))))
```

---

## locus\_id: 111421

family\_id: 200

### **Targets:**

At1g52240(NM\_104103.2
): expressed protein

Location in genome: in an IGR, 345 upstream of At2g06906, 34022 downstream of At2g06925

### Alignment between mature miRNA and predicted targets

```
        Extended mature miRNA:       gactcttgcggttggacaactcaggt
        Target(rc):NM_104103.2       gtaTCTTGCGGTTGGACTCCTCAtag
```

### Precursor sequence and structure. Mature sequence in capital letters

```
gctggacTCTTGCGGTTGGACAACTCAggtgaacgaatttgtttcggaaacctgggttggccgactgcgggcgtctgac   
 ...((((.(((((((((((.((((((((((...((((.....))))...)))))))))).))))))))))).))))...
```

---

## locus\_id: 222812

family\_id: 199

### **Targets:**

At5g06120(NM\_120694.2
): Ran-binding protein, putative

Location in genome: in an IGR, 1560 upstream of At3g02840, 686 downstream of At3g02830

2 homologs in brassica

### Alignment between mature miRNA and predicted targets

```
        Extended mature miRNA:       ctctccatgacaattttgcttcatgg
        Target(rc):NM_120694.2       caaTCCATGACAATTTTGCTTCAatg
```

### Precursor sequence and structure. Mature sequence in capital letters

```
tgcctcTCCATGACAATTTTGCTTCAtggaaatcttctcttccatggagtagatctatggagaggca   
 .(((((((((((.....((((((((((((((........))))))))))))))..))))))))))).
```

---

## locus\_id: 161319

family\_id: 198

### **Targets:**

At3g53180(NM\_115178.4
): glutamine synthetase, putative

Location in genome: in an IGR, 30576 upstream of At2g23640, 770 downstream of At2g23540

### Alignment between mature miRNA and predicted targets

```
        Extended mature miRNA:       gatttcttgaggcttttgataacatggaca
        Target(rc):NM_115178.4       ataTTCTTGAGGCTTTTGAAAGCATGGgac
```

### Precursor sequence and structure. Mature sequence in capital letters

```
gatagatTTCTTGAGGCTTTTGATAACATGGacatatgatctgcatctttgcattttcaagtgcaaagatgcagatcatatgtccatgttaatatcaaagcctcaagaactctatc   
 ((((((.(((((((((((((.((((((((((((((((((((((((((((((((((....))))))))))))))))))))))))))))))...))))))))))))))))).))))))
```

---

## locus\_id: 172007

family\_id: 197

### **Targets:**

At3g47010(NM\_114568.3
): glycosyl hydrolase family 3 protein

Location in genome: in an IGR, 9611 upstream of At2g39190, 6978 downstream of At2g39140

### Alignment between mature miRNA and predicted targets

```
        Extended mature miRNA:       gtggatggcgtatgaggagccatgca
        Target(rc):NM_114568.3       aacAATGGCGTATGAGAAGCCATcac
```

### Precursor sequence and structure. Mature sequence in capital letters

```
gtatgcctggctccctgtatgccatatgctgagcccatcgagtatcgatgacctccgtgGATGGCGTATGAGGAGCCATgcatat   
 ((((((.(((((((..(((((((((.(((.(((..(((((.....)))))..))).))).)))))))))..))))))).))))))
```

---

## locus\_id: 184662

family\_id: 196

### **Targets:**

At3g28030(NM\_113721.1
): UV hypersensitive protein (UVH3) / DNA-repair protein, putative

Location in genome: Contained by At3g11910 (NM\_112024: . ubiquitin-specific protease, putative) in an intron

### Alignment between mature miRNA and predicted targets

```
        Extended mature miRNA:       agttctaccttctcgtcatgcatatg
        Target(rc):NM_113721.1       ataTCTCCCTTCTCGTCACGCATcgc
```

### Precursor sequence and structure. Mature sequence in capital letters

```
atggctggttttgctcctgatgaggaaatagaactttttgaggttggggctggggcctttaagtTCTACCTTCTCGTCATGCATatggccat   
 (((((((....(((...(((((((((..((((((((...((((((........)))))).))))))))..))))))))).)))..)))))))
```

---

## locus\_id: 213882

family\_id: 195

### **Targets:**

At2g31650(NM\_179839.1
): trithorax 1 (ATX-1) (TRX1)

Location in genome: in an IGR, 1362 upstream of At3g51320, 486 downstream of At3g51330

### Alignment between mature miRNA and predicted targets

```
        Extended mature miRNA:       ttgttcgtcaagctccacattttggt
        Target(rc):NM_179839.1       aatTTCATCAAGCTCCACACTTTcag
```

### Precursor sequence and structure. Mature sequence in capital letters

```
ttcactaacaagttttggagtttgaagaacaaatatgtgggtaaaaggcaaaaaggatgcagatcaataatcttttacctacatacttgTTCGTCAAGCTCCACATTTTggtagtgaa   
 .((((((.((((...((((((((((.((((((.(((((((((((((((.......(((....))).....))))))))))))))).)))))).))))))))))...)))).)))))).
```

---

## locus\_id: 255076

family\_id: 194

### **Targets:**

At4g16070(NM\_117700.2
): lipase class 3 family protein

Location in genome: in an IGR, 3797 upstream of At3g48270, 4747 downstream of At3g48240

### Alignment between mature miRNA and predicted targets

```
        Extended mature miRNA:       gaagaggacgaggctagctcagaagaa
        Target(rc):NM_117700.2       catGAGGACGAGGTTACCTCAGAAcga
```

### Precursor sequence and structure. Mature sequence in capital letters

```
gttgggtgaaGAGGACGAGGCTAGCTCAGAAgaatcaagtgaataagaacacagtgatgtgacctatcatcaccgcgttcttttcttctcccagactcttctgagctagttacgtcttcacctaac   
 (((((((((...(((((..((((((((((((((.((..(.(((.((((((.(.((((((........)))))).).))))))...))).)...)).))))))))))))))..))))))))))))))
```

---

## locus\_id: 127400

family\_id: 193

### **Targets:**

At2g26780(NM\_179755.1
): expressed protein

Location in genome: Contained by At2g25170 (NM\_128074: . chromatin remodeling factor CHD3 (PICKLE)) in an intron on the reverse strand

### Alignment between mature miRNA and predicted targets

```
        Extended mature miRNA:       ctgtacacacagtttcagcacatgct
        Target(rc):NM_179755.1       gcaTCCACACAGTTCCAGCACATcgt
```

### Precursor sequence and structure. Mature sequence in capital letters

```
agatccagcatatgctgaaactgtgtgtttatgagaactctgTACACACAGTTTCAGCACATgctcgacct   
 ((.((.(((((.((((((((((((((((....((....))...)))))))))))))))).))))).)).))
```

---

## locus\_id: 149101

family\_id: 192

### **Targets:**

At5g57460(NM\_125128.2
): expressed protein

Location in genome: in an IGR, 17312 upstream of At2g07713, 1854 downstream of At2g07708

1 homologs in brassica

### Alignment between mature miRNA and predicted targets

```
        Extended mature miRNA:       cttcccaccgcgtccttccttgtgta
        Target(rc):NM_125128.2       aagCCCACCGAGTCCTTCCTTCTtct
```

### Precursor sequence and structure. Mature sequence in capital letters

```
gatgccagcggaatgatcggccggacagaggctagggctgcttccttCCCACCGCGTCCTTCCTTGTgtatc   
 ((((((((.((((.((((((..(((..(((((.......)))))..)))..))).))).))))))).)))))
```

---

## locus\_id: 162079

family\_id: 191

### **Targets:**

At3g54040(NM\_115264.3
): photoassimilate-responsive protein-related

Location in genome: in an IGR, 6218 upstream of At2g24520, 3736 downstream of At2g24510

### Alignment between mature miRNA and predicted targets

```
        Extended mature miRNA:       gatggatgagtaaagcttaccggcgcca
        Target(rc):NM_115264.3       agcGGATGAGCAAAGCTTAGCGGCGaaa
```

### Precursor sequence and structure. Mature sequence in capital letters

```
tgatGGATGAGTAAAGCTTACCGGCGccaatgaattatggaacgaagacgatgaattagtagagcttaccttcatcatcgtcgttccataactcaatggctctgttgatctttactcatctttcg   
 .((.((((((((((((.(((.(((.((((.(((.(((((((((((.((.((((((..((.....))....)))))).)).))))))))))).))).)))).))).))).)))))))))))).)).
```

---

## locus\_id: 193546

family\_id: 190

### **Targets:**

At4g03740(NM\_116612.1
): hypothetical protein

Location in genome: in an IGR, 4416 upstream of At3g23440, 13914 downstream of At3g23490

### Alignment between mature miRNA and predicted targets

```
        Extended mature miRNA:       tttctaacgaatattgttgtcaggcgtt
        Target(rc):NM_116612.1          CTAACGAATATTGTTGACAGGCatt
```

### Precursor sequence and structure. Mature sequence in capital letters

```
acgcttttctgacgataaaatttgctgcgaaaccggttggaaactagtcgaaaatgatcgtcggatcctcgtcggaaatagtttccgactattttCTAACGAATATTGTTGTCAGGCgt   
 ((((....((((((((((.(((((.((.((((..((((((((((((.((((....((((....))))....))))...))))))))))))..)))))).))))).))))))))))))))
```

---

## locus\_id: 197521

family\_id: 189

### **Targets:**

At2g30320(NM\_128585.1
): tRNA pseudouridine synthase family protein

Location in genome: in an IGR, 41611 upstream of At3g28280, 3363 downstream of At3g28330

### Alignment between mature miRNA and predicted targets

```
        Extended mature miRNA:       tggtggagtcagtggcaacacaattgat
        Target(rc):NM_128585.1       agaTGGAGCCAGTGGCAAGACAATTctt
```

### Precursor sequence and structure. Mature sequence in capital letters

```
agtcgaatgtgtgtcatcatctcctttagccggtttatagagttagccattgactcgactggTGGAGTCAGTGGCAACACAATTgatt   
 ((((((.((((((((((...((((....(((((((....(((((((...))))))))))))))))))...))))).))))).))))))
```

---

## locus\_id: 249003

family\_id: 188

### **Targets:**

At3g25290(NM\_113435.2
): auxin-responsive family protein

Location in genome: in an IGR, 2172 upstream of At3g42380, 12800 downstream of At3g42340

### Alignment between mature miRNA and predicted targets

```
        Extended mature miRNA:       aagttgtgctaccgctgtagtcagac
        Target(rc):NM_113435.2       agaTTGTGCCACCGCTGTTGTCAcct
```

### Precursor sequence and structure. Mature sequence in capital letters

```
tcaccaaaagTTGTGCTACCGCTGTAGTCAgacggggcattacataagggaatgcccgcagctacaagcggaggggtcagttggtga   
 (((((((...((((.((.((((((((((..(...(((((((.(.....).))))))).).))))).))))).)).).))))))))))
```

---

## locus\_id: 248026

family\_id: 187

### **Targets:**

At3g07100(NM\_111590.2
): protein transport protein Sec24, putative

Location in genome: in an IGR, 7359 upstream of At3g33494, 106466 downstream of At3g33131

### Alignment between mature miRNA and predicted targets

```
        Extended mature miRNA:       gcccgaatggatttggctccacgtttt
        Target(rc):NM_111590.2       cagCAAATGAATTTGGCTCCACGTcac
```

### Precursor sequence and structure. Mature sequence in capital letters

```
taaggcgtggggtcgaagttgggcttgcatggcttgggagaaaacttggtcaccaagtcagctctgccCGAATGGATTTGGCTCCACGTttta   
 (((((((((((((((((.((((((..((.((((((((..((........)).))))))))))...)))))).....)))))))))))))))))
```

---

## locus\_id: 227042

family\_id: 186

### **Targets:**

At2g38410(NM\_129397.2
): VHS domain-containing protein / GAT domain-containing protein

Location in genome: in an IGR, 202 upstream of At3g09550, 4025 downstream of At3g09530

4 homologs in brassica

### Alignment between mature miRNA and predicted targets

```
        Extended mature miRNA:       gaaggaactggaaccaggtatggagaa
        Target(rc):NM_129397.2       tggGGAACTGGAACCAGCTATGGAact
```

### Precursor sequence and structure. Mature sequence in capital letters

```
gagaaGGAACTGGAACCAGGTATGGAgaagggtctggcgacggatccaacgagtcctacagggagtactgtagcggacctgtcgccaactccaaccccaaggaagactctggttctgtccaattc   
 .....(((...((((((((((.(((.(..(((..(((((((((.(((........((((((......)))))).))).)))))))))..)))..).)))......)).)))))))).))).....
```

---

## locus\_id: 225446

family\_id: 185

### **Targets:**

At2g26360(NM\_128193.2
): mitochondrial substrate carrier family protein

Location in genome: Contained by At3g06510 (NM\_111527: . glycosyl hydrolase family 1 protein) in an intron

### Alignment between mature miRNA and predicted targets

```
        Extended mature miRNA:       tggctcatccagcatgtgacgggctt
        Target(rc):NM_128193.2       ttaCTCATCCAGCATGTGTCGTGgtt
```

### Precursor sequence and structure. Mature sequence in capital letters

```
ggtacctggCTCATCCAGCATGTGACGGGcttagactcaaaaaggtcatccgttgcagggctgggctggctttgggtcgcc   
 (((((((((((..((((((.((..(((((....((((......)))).)))))..))..))))))..))))..)))).)))
```

---

## locus\_id: 171564

family\_id: 184

### **Targets:**

At1g58050(NM\_104587.1
): helicase domain-containing protein

Location in genome: in an IGR, 2487 upstream of At2g38330, 4038 downstream of At2g38320

### Alignment between mature miRNA and predicted targets

```
        Extended mature miRNA:       ttttttggcgctatccatcctgagtt
        Target(rc):NM_104587.1       aacTTTGGTGCTATCCACCCTGAcct
```

### Precursor sequence and structure. Mature sequence in capital letters

```
ctgtaaagctcaggagggatagcgccatgatgatcacattcgttatctatttTTTGGCGCTATCCATCCTGAgtttcattg   
 .....((((((((((.(((((((((((.((((((.((....)).))).)))...))))))))))).)))))))))).....
```

---

## locus\_id: 162769

family\_id: 183

### **Targets:**

At1g33700(NM\_103090.3
): expressed protein

Location in genome: Contained by At2g25170 (NM\_128074: . chromatin remodeling factor CHD3 (PICKLE)) in an intron

### Alignment between mature miRNA and predicted targets

```
        Extended mature miRNA:       gtttcagcatatgctggatctacttga
        Target(rc):NM_103090.3       gcaTCAGCATATGCTGCACCTACTggc
```

### Precursor sequence and structure. Mature sequence in capital letters

```
tagttttccaataggtcgagcatgtgctgaaactgtgtgtacagagttctcataaacacacagttTCAGCATATGCTGGATCTACTtgaagacta   
 ((((((((...((((((.((((((((((((((((((((((...((....))....)))))))))))))))))))))).))))))...))))))))
```

---

## locus\_id: 206694

family\_id: 182

### **Targets:**

At5g32590(NM\_122838.1
): myosin heavy chain-related

Location in genome: in an IGR, 48824 upstream of At3g43110, 8849 downstream of At3g43153

### Alignment between mature miRNA and predicted targets

```
        Extended mature miRNA:       cagtggcagctggaggagatgtccaggggg
        Target(rc):NM_122838.1       caaTGGCAGTTGGAGAAGATGTCCAGGagg
```

### Precursor sequence and structure. Mature sequence in capital letters

```
agcctccacaatcgacttggacattgtcttgcgtcgagctgtgatggcccgagcagaagtccctttggataaagaagggggcagTGGCAGCTGGAGGAGATGTCCAGGggggct   
 (((((((.........(((((((((......(.((.((((((.((.((((........((((....))))........)))).)).)))))).)).).))))))))))))))))
```

---

## locus\_id: 133553

family\_id: 181

### **Targets:**

At5g11760(NM\_121214.2
): expressed protein

Location in genome: in an IGR, 11313 upstream of At2g34180, 25150 downstream of At2g34270

1 homologs in brassica

### Alignment between mature miRNA and predicted targets

```
        Extended mature miRNA:       ttgcgaggcaaatctcctttggcagag
        Target(rc):NM_121214.2       gaaCGAGTTAAATCTCCTTTGGCAcca
```

### Precursor sequence and structure. Mature sequence in capital letters

```
gaagcattgCGAGGCAAATCTCCTTTGGCAgaggaaaatgatgagaaaataacatataagggtcatcaacttccactgccaatagaggattcgccctgtaatgcttt   
 (((((((((((.(((.((((..(((((((((.((((..((((((..................))))))..)))).))))))).))..)))).))).)))))))))))
```

---

## locus\_id: 124667

family\_id: 180

### **Targets:**

At2g05360(NM\_126559.1
): hypothetical protein

Location in genome: in an IGR, 2917 upstream of At2g21880, 4888 downstream of At2g21900

### Alignment between mature miRNA and predicted targets

```
        Extended mature miRNA:       aagagatttgtagtccacaaacttgtatta
        Target(rc):NM_126559.1       agcAGATTTGTAGTCGACAAACTTGTActa
```

### Precursor sequence and structure. Mature sequence in capital letters

```
tcttgtaaagAGATTTGTAGTCCACAAACTTGTAttatgccgatttgtaagcagtttgtcggcatagtacaagttggtcgactacaaatctgcttacaaga   
 ((((((((..((((((((((((.((.(((((((((((((((((((((....)))...)))))))))))))))))).)).))))))))))))..))))))))
```

---

## locus\_id: 185907

family\_id: 179

### **Targets:**

At1g69770(NM\_105645.2
): chromomethylase 3 (CMT3)

Location in genome: in an IGR, 871 upstream of At3g13720, 1128 downstream of At3g13730

### Alignment between mature miRNA and predicted targets

```
        Extended mature miRNA:       tctagtgggtggtgatcatataagattg
        Target(rc):NM_105645.2       ttaAGAGGGTGATGATCATATAAGAcat
```

### Precursor sequence and structure. Mature sequence in capital letters

```
acgaatcttgtatgatcactaaccattggaacaataagagtatataatgagtatatcaccatttagttctAGTGGGTGGTGATCATATAAGAttggt   
 ((.((((((((((((((((((.((((((((((...((..(((((((.....))))).))..))..)))))))))).)))))))))))))))))).))
```

---

## locus\_id: 203198

family\_id: 178

### **Targets:**

At1g16080(NM\_101476.2
): expressed protein

Location in genome: in an IGR, 25512 upstream of At3g32410, 33139 downstream of At3g32902

### Alignment between mature miRNA and predicted targets

```
        Extended mature miRNA:       tgggcacctggttgacgagctacggg
        Target(rc):NM_101476.2       cttGCACCTGGATGACGAGCTCCaac
```

### Precursor sequence and structure. Mature sequence in capital letters

```
agattctcatggttggtcgactatgtgaacggtcgtgttctggGCACCTGGTTGACGAGCTACgggggtct   
 ((((((((.(((((.((((((((.(((..(((.......)))..))).)))))))).))))).))))))))
```

---

## locus\_id: 252728

family\_id: 177

### **Targets:**

At5g66850(NM\_126084.2
): protein kinase family protein

Location in genome: in an IGR, 2959 upstream of At3g44790, 6355 downstream of At3g44770

### Alignment between mature miRNA and predicted targets

```
        Extended mature miRNA:       gtcgccccatcaaacacactccaataa
        Target(rc):NM_126084.2       gcaGCCCCTTCAAACTCACTCCAAgga
```

### Precursor sequence and structure. Mature sequence in capital letters

```
gataaaatactggagtgtgtttgatggagtgatagacataagtgtgtttgataacatgcatgtcGCCCCATCAAACACACTCCAAtaatgtc   
 ..........(((((((((((((((((.((((((.......(((((((....))))))).)))))).)))))))))))))))))........
```

---

## locus\_id: 201718

family\_id: 176

### **Targets:**

At3g28550(NM\_113775.2
): proline-rich extensin-like family protein

Location in genome: in an IGR, 3464 upstream of At3g31330, 79216 downstream of At3g31410

### Alignment between mature miRNA and predicted targets

```
        Extended mature miRNA:       ggcctggagagtgctctagtggtggctg
        Target(rc):NM_113775.2       gcgCTGGAGAGTATTCTAGTGGTGGtgg
```

### Precursor sequence and structure. Mature sequence in capital letters

```
ttacagcgctactcaacggtggtgggtggggatctcgctagagctcttctctcccggctgtctcctgctcctgcctaagcgatggcCTGGAGAGTGCTCTAGTGGTGGctgtga   
 (((((((.(((((((.......))))))).....((((((((((....((((((.(((((((.(..((....))....).))))))).)))))).))))))))))..)))))))
```

---

## locus\_id: 211874

family\_id: 175

### **Targets:**

At3g10405(NM\_148703.2
): expressed protein

Location in genome: in an IGR, 914 upstream of At3g48250, 150 downstream of At3g48260

### Alignment between mature miRNA and predicted targets

```
        Extended mature miRNA:       ggtgaagacgtaactagctcagaaga
        Target(rc):NM_148703.2       aaaGAAGACGTAACTAGCCCAAAgat
```

### Precursor sequence and structure. Mature sequence in capital letters

```
gttaggtGAAGACGTAACTAGCTCAGAagagtctgggagaagaaaagaacgcggtgatgataggtcacatcactgtgttcttattcacttgattcttctgagctagcctcgtcctcttcacccaac   
 (((.((((((((((...((((((((((((((((.((..(((...(((((((((((((((........))))))))))))))).))).)).))))))))))))))))...))))...)))))).)))
```

---

## locus\_id: 218300

family\_id: 174

### **Targets:**

At1g80790(NM\_106727.2
): XH/XS domain-containing protein / XS zinc finger domain-containing protein

Location in genome: in an IGR, 789 upstream of At3g58280, 480 downstream of At3g58290

### Alignment between mature miRNA and predicted targets

```
        Extended mature miRNA:       tgaacctcaagagggttgaaccggtt
        Target(rc):NM_106727.2       ttcACCTCAAGAGGATTGAACCTagc
```

### Precursor sequence and structure. Mature sequence in capital letters

```
ttgagccggtttgctgaacctcaagaggattgagccggtttgctgaACCTCAAGAGGGTTGAACCGgtttaa   
 .(((((((((((......((((..((((.((.(((......))).))))))..))))...))))))))))).
```

---

## locus\_id: 120031

family\_id: 173

### **Targets:**

At1g47660(NM\_103659.1
): hypothetical protein

Location in genome: in an IGR, 6845 upstream of At2g15390, 6046 downstream of At2g15420

### Alignment between mature miRNA and predicted targets

```
        Extended mature miRNA:       gtcgggcgagctggtgggggcgtcgtctg
        Target(rc):NM_103659.1       gttGGCCGAGCTGGTGGTGGCGTCGTtgg
```

### Precursor sequence and structure. Mature sequence in capital letters

```
ggtcggcaggagctgcgacaggagctgcggctggaggtgcgacaggaactgcggctggaggtgcgacaggaactgcggctagaggtggcatcgtcGGGCGAGCTGGTGGGGGCGTCGTctgccgagc   
 (.((((((((.(((.(.((...((((.((.(((..(((((.((.....(((((((((.........)))...))))))......)).)))))..))).)))))).)).).)))....)))))))).)
```

---

## locus\_id: 120028

family\_id: 172

### **Targets:**

At1g47660(NM\_103659.1
): hypothetical protein

Location in genome: in an IGR, 6797 upstream of At2g15390, 6092 downstream of At2g15420

### Alignment between mature miRNA and predicted targets

```
        Extended mature miRNA:       caagaggcgcgctggttccgactccttgctc
        Target(rc):NM_103659.1       cacGAGGCGCGATGGTTCTGACTCCTTGttc
```

### Precursor sequence and structure. Mature sequence in capital letters

```
tcgggcgagctggtgggggcgtcgtctgccgagctggtggtccggcaaGAGGCGCGCTGGTTCCGACTCCTTGctcgttcga   
 ((((((((((.(((.((((((.(((((((((..(.....)..))))...)))))))))....)).)))....))))))))))
```

---

## locus\_id: 123604

family\_id: 171

### **Targets:**

At1g27910(NM\_102556.3
): U-box domain-containing protein

Location in genome: in an IGR, 121 upstream of At2g20300, 964 downstream of At2g20310

### Alignment between mature miRNA and predicted targets

```
        Extended mature miRNA:       gcgtcggtgttcgctaacgccatcatca
        Target(rc):NM_102556.3       aacTCGGTGTTCTCTAACTCCATCAaaa
```

### Precursor sequence and structure. Mature sequence in capital letters

```
ggttttgtcgatgtgacggtagcaaacggccaccagttttgtcgtttcgatgtgcgtggccaccacgcgcatacgaaaccgcgTCGGTGTTCGCTAACGCCATCAtcatcgcc   
 (((..((..((((.(.((.((((....(..((((.(...(((.((((((((((((((((...)))))))))).)))))).))).)))))..))))).)))))))..))..)))
```

---

## locus\_id: 147378

family\_id: 170

### **Targets:**

At2g36070(NM\_129166.3
): mitochondrial import inner membrane translocase subunit TIM44, putative

Location in genome: in an IGR, 28333 upstream of At2g06845, 55076 downstream of At2g06690

### Alignment between mature miRNA and predicted targets

```
        Extended mature miRNA:       cagggagaagaggccgctttgtaactc
        Target(rc):NM_129166.3       gctGGAGAAGAGGCTGCTTTGTGAtca
```

### Precursor sequence and structure. Mature sequence in capital letters

```
ttcagGGAGAAGAGGCCGCTTTGTAActcagtttcctctaaaggaggaggagtagggggatcagaacttggtaaatttctctctagaggtggtctcctcgctctgga   
 (((((((.((.(((((((((((...((((..((((((....))))))..))))(((((((.................))))))).))))))))))).)).)))))))
```

---

## locus\_id: 112266

family\_id: 169

### **Targets:**

At5g48850(NM\_124262.2
): male sterility MS5 family protein

Location in genome: in an IGR, 3847 upstream of At2g07776, 525 downstream of At2g07777

### Alignment between mature miRNA and predicted targets

```
        Extended mature miRNA:       gcctccttgctcaaacctgccttctt
        Target(rc):NM_124262.2       tctTCCCTCCTCAAACCTGCCTTgtt
```

### Precursor sequence and structure. Mature sequence in capital letters

```
cggggaaggagtgttatgaaagggaggaagaggaaaagctactcttgagccTCCTTGCTCAAACCTGCCTTcttcg   
 (((((((((((.(((.(((.((((((((((((.........)))))...)))))))..)))))))).)))))))))
```

---

## locus\_id: 206988

family\_id: 168

### **Targets:**

At5g32775(NM\_147987.1
): hypothetical protein

Location in genome: in an IGR, 8344 upstream of At3g43300, 22064 downstream of At3g43320

### Alignment between mature miRNA and predicted targets

```
        Extended mature miRNA:       gactggtcgagtggtttgtcagcttg
        Target(rc):NM_147987.1       tctTGGTCGAGTGGTTTGTCATGatc
```

### Precursor sequence and structure. Mature sequence in capital letters

```
gatgctgctgggatgacaggtcgagtggttggtcgagtaacgctgtcgagtggcagatcgagtggttgctcgagtgacTGGTCGAGTGGTTTGTCAGCttgatc   
 (((...(((((.(.(((.(.((((.((((((.(((((((((.(..((((........))))..)))))))))).)))))).)))).).)))).)))))...)))
```

---

## locus\_id: 244083

family\_id: 167

### **Targets:**

At5g02190(NM\_120297.2
): aspartyl protease family protein

Location in genome: in an IGR, 56814 upstream of At3g30420, 23653 downstream of At3g30380

### Alignment between mature miRNA and predicted targets

```
        Extended mature miRNA:       tttcggatcggttccggatacggataa
        Target(rc):NM_120297.2       ttcCGGGTCGGATCCGGATACGGAacc
```

### Precursor sequence and structure. Mature sequence in capital letters

```
ttcgttatccgtatctggatccggaaatctcggataatcgttttttCGGATCGGTTCCGGATACGGAtaacgga   
 (((((((((((((((((((((((((((...((......))..)))))))).....)))))))))))))))))))
```

---

## locus\_id: 165416

family\_id: 166

### **Targets:**

At4g38660(NM\_120027.2
): thaumatin, putative

Location in genome: in an IGR, 33009 upstream of At2g28920, 4246 downstream of At2g28840

### Alignment between mature miRNA and predicted targets

```
        Extended mature miRNA:       ctttttgtcgtacttggtgttcttagt
        Target(rc):NM_120027.2       ttcTTTGTCGTACTCAGTGTTCTTggt
```

### Precursor sequence and structure. Mature sequence in capital letters

```
gctgagactaagtacggtgtaagagaagcaaatagggaaactgaggaaaccattgctctttgttttctattatttgcttTTTGTCGTACTTGGTGTTCTTagt   
 (((((((((((((((((.....(((((((((((((((((((.(((.((....)).)))...)))))))..)))))))))))).))))))))))...)))))))
```

---

## locus\_id: 149717

family\_id: 165

### **Targets:**

At4g17540(NM\_117860.4
): expressed protein

Location in genome: in an IGR, 7059 upstream of At2g07787, 7699 downstream of At2g07758

47 homologs in brassica

2 homologs in rice

### Alignment between mature miRNA and predicted targets

```
        Extended mature miRNA:       aactggtcgctcctctagttgaagta
        Target(rc):NM_117860.4       catTGGTTGCTCCTCTAGCTGAAttc
```

### Precursor sequence and structure. Mature sequence in capital letters

```
gctccgataacgaaattccgggaggagtcggtagttgggcactggatcccttcggacctggagaacgtgtgacgctgggtaggggtttggtgaaccaacTGGTCGCTCCTCTAGTTGAAgtatcgggc   
 ((.((((((....((((...((((((((.(.(((((((.(((..(((((((....(((..(....((.....)))..))))))))))..)))..))))))).).))))))))))))....))))))))
```

---

## locus\_id: 236630

family\_id: 164

### **Targets:**

At2g28270(NM\_128387.1
): DC1 domain-containing protein

Location in genome: in an IGR, 4539 upstream of At3g22890, 10693 downstream of At3g22870

### Alignment between mature miRNA and predicted targets

```
        Extended mature miRNA:       agtctcgattagatcatgttcgcagtt
        Target(rc):NM_128387.1       ttgCCCAATTAGATCATGTTCGCAtcc
```

### Precursor sequence and structure. Mature sequence in capital letters

```
gaagctgccagcatgatctaattagctttctttatcctttgttgtgtttcatgacgatggttaagagatcagtCTCGATTAGATCATGTTCGCAgtttc   
 ((((((((.((((((((((((((((((.(((((..((.((((..((...))..)))).))...)))))..)).)).)))))))))))))).))))))))
```

---

## locus\_id: 228643

family\_id: 163

### **Targets:**

At2g26730(NM\_128230.2
): leucine-rich repeat transmembrane protein kinase, putative

Location in genome: in an IGR, 7756 upstream of At3g12170, 13449 downstream of At3g12110

### Alignment between mature miRNA and predicted targets

```
        Extended mature miRNA:       cagtgcattccactccgatccagcca
        Target(rc):NM_128230.2       agtTGCATTCGACTCCGACCCAGtta
```

### Precursor sequence and structure. Mature sequence in capital letters

```
gcatggtgatcgcgggtgggatatttccggagattgtactggcggaaaggtagaggtaggtgacgcggccgtcagTGCATTCCACTCCGATCCAGccagt   
 ...(((((((((.((((((((.............(((((((((((...(((...........)).)..))))))))))))))))))))))))..))))..
```

---

## locus\_id: 232776

family\_id: 162

### **Targets:**

At2g07779(NM\_201710.1
): expressed protein

Location in genome: in an IGR, 1387 upstream of At3g17720, 4261 downstream of At3g17710

### Alignment between mature miRNA and predicted targets

```
        Extended mature miRNA:       ggtcttttttccgacccgatttgcagc
        Target(rc):NM_201710.1       ggcCTTTATTCCGACCCGACTTGCgga
```

### Precursor sequence and structure. Mature sequence in capital letters

```
ggattgttgctgcggtgggttggaataagctaagatccatcaaatatacccgtgggtCTTTTTTCCGACCCGATTTGCagccatacc   
 ....(((.((((((.((((((((((......(((((((((...........)))))))))..))))))))))...)))))).)))..
```

---

## locus\_id: 149718

family\_id: 161

### **Targets:**

At1g75930(NM\_106243.2
): family II extracellular lipase 6 (EXL6)

Location in genome: in an IGR, 7011 upstream of At2g07787, 7745 downstream of At2g07758

43 homologs in brassica

1 homologs in rice

### Alignment between mature miRNA and predicted targets

```
        Extended mature miRNA:       taggttacaccttcggaataccccagaa
        Target(rc):NM_106243.2       gaaGTTACACCATCCGAATACCCCAccg
```

### Precursor sequence and structure. Mature sequence in capital letters

```
tcccttcggacctggagaacgtgtgacgctgggtaggggtttggtgaaccaactggtcgctcctctagttgaagtatcgggccccttttcgttgcctagGTTACACCTTCGGAATACCCCAgaaggga   
 (((((((((..((((((...(((((((.((((((((((((((((((...(((((((........)))))))...)))))))))))).......)))))))))))))))))))......)).)))))))
```

---

## locus\_id: 169767

family\_id: 160

### **Targets:**

At1g68110(NM\_105481.2
): epsin N-terminal homology (ENTH) domain-containing protein / clathrin assembly protein-related

Location in genome: in an IGR, 7016 upstream of At2g35790, 339 downstream of At2g35760

### Alignment between mature miRNA and predicted targets

```
        Extended mature miRNA:       agaaggtggctttggtgatggctttg
        Target(rc):NM_105481.2       gggAGGTGGCTTTGATGATAGCTgct
```

### Precursor sequence and structure. Mature sequence in capital letters

```
tggagaagaAGGTGGCTTTGGTGATGGCTttgatgttgcttatgtccgtgttggtttctgccgaggaaacaccaaccatcggacagaggatagactcagccgcaaccgactttaccaaattcttcg   
 .((((((...(((((..(((((..(((((..((.(((.(((.((((((.((((((((((.....))))..))))))...)))))).)))...))))))))))..)))))..)))))...)))))).
```

---

## locus\_id: 224933

family\_id: 159

### **Targets:**

At1g64320(NM\_105106.1
): myosin heavy chain-related

Location in genome: in an IGR, 8562 upstream of At3g05870, 2216 downstream of At3g05840

### Alignment between mature miRNA and predicted targets

```
        Extended mature miRNA:       cttcagctcggttcatgacttcctccat
        Target(rc):NM_105106.1       tagCAACTCGGTTCGTGACTTCCTCtgc
```

### Precursor sequence and structure. Mature sequence in capital letters

```
catcttCAGCTCGGTTCATGACTTCCTCcatagtcacagcgggaggagctgagactgagattgagatg   
 (((((.((((((((((((.(.((.(((((...((....)).))))))))))).)))))).))))))))
```

---

## locus\_id: 197878

family\_id: 158

### **Targets:**

At4g19590(NM\_118079.1
): DNAJ heat shock N-terminal domain-containing protein

Location in genome: in an IGR, 6207 upstream of At3g28710, 6745 downstream of At3g28740

### Alignment between mature miRNA and predicted targets

```
        Extended mature miRNA:       gcgattttggtggttgtttaggcctc
        Target(rc):NM_118079.1       cttGATTTGGTGGTTGTTTAGGCtgg
```

### Precursor sequence and structure. Mature sequence in capital letters

```
aagcgATTTTGGTGGTTGTTTAGGCctcgactaggggctaggctctcatgcgacgcctagatgaccgtttcgaccgctt   
 (((((.((..((((((..(((((((.(((....(((((...)))))....))).)))))))..))))))..)).)))))
```

---

## locus\_id: 235987

family\_id: 157

### **Targets:**

At5g33340(NM\_122858.1
): aspartyl protease family protein

Location in genome: in an IGR, 8916 upstream of At3g22150, 11663 downstream of At3g22104

### Alignment between mature miRNA and predicted targets

```
        Extended mature miRNA:       tggctttgcgattggaggagttgcaa
        Target(rc):NM_122858.1       cgcCTTTGCGATCAGAGGAGTTGaga
```

### Precursor sequence and structure. Mature sequence in capital letters

```
gtggctttgtcataggtggggttgcaacaggcgacttagcgataggtggagttgctacgggtggtgtggcaacgggtggccgtgcgccgggtggCTTTGCGATTGGAGGAGTTGc   
 (..((((..(((........(((((((..(((.(((..(((...(((.(.(((((((((.....)))))))))...).)))...)))..))).)))))))))))))..))))..)
```

---

## locus\_id: 154298

family\_id: 156

### **Targets:**

At4g39590(NM\_120119.2
): kelch repeat-containing F-box family protein

Location in genome: in an IGR, 17284 upstream of At2g14330, 15877 downstream of At2g14255

### Alignment between mature miRNA and predicted targets

```
        Extended mature miRNA:       gtcagaccgacaatccaggagcgagacacc
        Target(rc):NM_120119.2       gtgAGACCGACAATCCAGGATAGAGACttt
```

### Precursor sequence and structure. Mature sequence in capital letters

```
gccactcgtcAGACCGACAATCCAGGAGCGAGACaccggagtcataaagtttatctttttgttttttgttaaaaagatttttgacccgagtgagtctccgtcctggctccggtgtgaagtgtggc   
 (((((.(.(((.((((.....((((((..((((((((((.((((........(((((((((........)))))))))...))))))).))..)))))..))))))...)))).))).).)))))
```

---

## locus\_id: 124763

family\_id: 155

### **Targets:**

At1g67560(NM\_105423.2
): lipoxygenase family protein

Location in genome: in an IGR, 10181 upstream of At2g22040, 1818 downstream of At2g22090

### Alignment between mature miRNA and predicted targets

```
        Extended mature miRNA:       agtacgggcagtgactctatgtgatc
        Target(rc):NM_105423.2       accACGGCCAGTGACTCCATGTGgtg
```

### Precursor sequence and structure. Mature sequence in capital letters

```
agagatgaaagagaagagtgtgaagctgcccgtagttgttactacgaatccttagggcagtACGGGCAGTGACTCTATGTGatcatctct   
 ((((((((..(...(((((.....((((((((((.(((((.(((.(....).)))))))))))))))))).)))))...)..))))))))
```

---

## locus\_id: 203688

family\_id: 152

### **Targets:**

At1g03457(NM\_100228.3
): RNA-binding protein, putative  
At1g03457(NM\_202023.1
): RNA-binding protein, putative

Location in genome: in an IGR, 30619 upstream of At3g33073, 144064 downstream of At3g33080

### Alignment between mature miRNA and predicted targets

```
        Extended mature miRNA:       atcttgggaagttcctgcggggtgaat
        Target(rc):NM_202023.1       ccgTTGGGAAGTTTCTGCGGGGGGcca
        Target(rc):NM_100228.3       ccgTTGGGAAGTTTCTGCGGGGGGcca
```

### Precursor sequence and structure. Mature sequence in capital letters

```
tctgtcggatatctagcagcttctcaggtcctgtgttccattccagtgtgggtgatagtatcTTGGGAAGTTCCTGCGGGGTGaatgacgga   
 (((((((..(((((.(((((((((((((..((((..(((((......)))))..))))...)))))))))...)))).)))))..)))))))
```

---

## locus\_id: 247405

family\_id: 152

### **Targets:**

At1g03457(NM\_100228.3
): RNA-binding protein, putative  
At1g03457(NM\_202023.1
): RNA-binding protein, putative

Location in genome: in an IGR, 190877 upstream of At3g33131, 119125 downstream of At3g33064

### Alignment between mature miRNA and predicted targets

```
        Extended mature miRNA:       atcttgggaagttcctgcggggtgaat
        Target(rc):NM_202023.1       ccgTTGGGAAGTTTCTGCGGGGGGcca
        Target(rc):NM_100228.3       ccgTTGGGAAGTTTCTGCGGGGGGcca
```

### Precursor sequence and structure. Mature sequence in capital letters

```
tctgtcggatatctagcagcttctcaggtcctgtgttccattccagtgtgggtgatagtatcTTGGGAAGTTCCTGCGGGGTGaatgacgga   
 (((((((..(((((.(((((((((((((..((((..(((((......)))))..))))...)))))))))...)))).)))))..)))))))
```

---

## locus\_id: 395449

family\_id: 152

### **Targets:**

At1g03457(NM\_202023.1
): RNA-binding protein, putative  
At1g03457(NM\_100228.3
): RNA-binding protein, putative

Location in genome: in an IGR, 5816 upstream of At5g28340, 778 downstream of At5g28330

### Alignment between mature miRNA and predicted targets

```
        Extended mature miRNA:       atcttgggaagttcctgcggggtgaat
        Target(rc):NM_202023.1       ccgTTGGGAAGTTTCTGCGGGGGGcca
        Target(rc):NM_100228.3       ccgTTGGGAAGTTTCTGCGGGGGGcca
```

### Precursor sequence and structure. Mature sequence in capital letters

```
tctgtcggatatctagcagcttctcaggtcctgtgttccattccagtgtgggtgatagtatcTTGGGAAGTTCCTGCGGGGTGaatgacgga   
 (((((((..(((((.(((((((((((((..((((..(((((......)))))..))))...)))))))))...)))).)))))..)))))))
```

---

## locus\_id: 177437

family\_id: 151

### **Targets:**

At5g39270(NM\_123289.1
): expansin, putative (EXP22)  
At5g39290(NM\_123291.1
): expansin, putative (EXP26)

Location in genome: in an IGR, 950 upstream of At2g47590, 1955 downstream of At2g47580

1 homologs in brassica

### Alignment between mature miRNA and predicted targets

```
        Extended mature miRNA:       tatttgcacgtacttaacttctccaaca
        Target(rc):NM_123289.1       aacTTGCACGTACTTAATATCTCCAgct
        Target(rc):NM_123291.1       aacTTGCACGTACTTAATATCTCCAgct
```

### Precursor sequence and structure. Mature sequence in capital letters

```
catgttggagaagcagggcacgtgcaaaccaacaaacacgaaatccgtctcatttgcttatTTGCACGTACTTAACTTCTCCAacatg   
 (((((((((((((..(((.(((((((((....((((.(((.....)))....))))....))))))))).)))..)))))))))))))
```

---

## locus\_id: 127279

family\_id: 149

### **Targets:**

At4g28580(NM\_119000.2
): magnesium transporter CorA-like family protein (MRS2-6)

Location in genome: in an IGR, 28849 upstream of At2g24970, 3931 downstream of At2g25050

### Alignment between mature miRNA and predicted targets

```
        Extended mature miRNA:       ttattctccatcttcttgatgaccagtt
        Target(rc):NM_119000.2       ctcTTCTCCACCTTCTTGATGACCAtga
```

### Precursor sequence and structure. Mature sequence in capital letters

```
gcattaTTCTCCATCTTCTTGATGACCAgtttgttgttaggctggtcagcatcgataagaatagtgc   
 ((((((((((..(((....((.(((((((((((....))))))))))).))..))).))))))))))
```

---

## locus\_id: 202100

family\_id: 149

### **Targets:**

At4g28580(NM\_119000.2
): magnesium transporter CorA-like family protein (MRS2-6)

Location in genome: in an IGR, 52738 upstream of At3g31540, 9370 downstream of At3g31910

### Alignment between mature miRNA and predicted targets

```
        Extended mature miRNA:       ttattctccatcttcttgatgaccagtt
        Target(rc):NM_119000.2       ctcTTCTCCACCTTCTTGATGACCAtga
```

### Precursor sequence and structure. Mature sequence in capital letters

```
gcattaTTCTCCATCTTCTTGATGACCAgtttgtttttaggctggtcagcatcgataagaatagtgt   
 ((((((((((..(((....((.(((((((((((....))))))))))).))..))).))))))))))
```

---

## locus\_id: 360935

family\_id: 146

### **Targets:**

At5g12090(NM\_121247.1
): protein kinase family protein

Location in genome: in an IGR, 847 upstream of At5g42957, 2187 downstream of At5g42970

### Alignment between mature miRNA and predicted targets

```
        Extended mature miRNA:       acttgtgcgagttggcacgtgtgcga
        Target(rc):NM_121247.1       gcaCGTGGGAGTTGGCACGTGTGaag
```

### Precursor sequence and structure. Mature sequence in capital letters

```
gttcttcgttcgcatgtgcgagttggcacgtgtgtgaagtggcactTGTGCGAGTTGGCACGTGTGcgaagtagc   
 (((((((((..((((((((.(..(.(((((.((((......)))).))))).)..).)))))))))))))).)))
```

---

## locus\_id: 360936

family\_id: 146

### **Targets:**

At5g12090(NM\_121247.1
): protein kinase family protein

Location in genome: in an IGR, 879 upstream of At5g42957, 2151 downstream of At5g42970

### Alignment between mature miRNA and predicted targets

```
        Extended mature miRNA:       gttcgcatgtgcgagttggcacgtgtgtga
        Target(rc):NM_121247.1       accCGCACGTGGGAGTTGGCACGTGTGaag
```

### Precursor sequence and structure. Mature sequence in capital letters

```
gttcttcgttCGCATGTGCGAGTTGGCACGTGTGtgaagtggcacttgtgcgagttggcacgtgtgcgaagtagcacttttgcgaattggagtcc   
 ...(((((((((((.((((.(.((.((((((((((.((.(.(((....))).).)).)))))))))).)).).))))...)))))).)))))...
```

---

## locus\_id: 248355

family\_id: 144

### **Targets:**

At5g06490(NM\_120732.1
): zinc finger (C3HC4-type RING finger) family protein

Location in genome: in an IGR, 5660 upstream of At3g41768, 84486 downstream of At3g33230

### Alignment between mature miRNA and predicted targets

```
        Extended mature miRNA:       ccgtgtccgaggagtggtcgagggttt
        Target(rc):NM_120732.1       tctTGTCCTAGGAGTGGTCGTGGGaga
```

### Precursor sequence and structure. Mature sequence in capital letters

```
gaccgTGTCCGAGGAGTGGTCGAGGGtttgtcgaccaggacgaggaatcgtcgaccgggtccgaggatttgtc   
 (((...((((..(((.(((((((.(((((.(((.......))).))))).)))))).).)))..))))..)))
```

---

## locus\_id: 248385

family\_id: 144

### **Targets:**

At5g06490(NM\_120732.1
): zinc finger (C3HC4-type RING finger) family protein

Location in genome: in an IGR, 2995 upstream of At3g41768, 87151 downstream of At3g33230

### Alignment between mature miRNA and predicted targets

```
        Extended mature miRNA:       ccgtgtccgaggagtggtcgagggttt
        Target(rc):NM_120732.1       tctTGTCCTAGGAGTGGTCGTGGGaga
```

### Precursor sequence and structure. Mature sequence in capital letters

```
tcgaccgTGTCCGAGGAGTGGTCGAGGGtttgtcgaccaggacgaggaatcgtcgaccgggtccgaggatttgtcgaccaggggttgaaatcgtcgaccaggtccgagacttcatcga   
 ((((....(((((.((..(((((((.((((..((((((.((.(((.(((((.(((........))).))))).))).))...)))))))))).)))))))..)))).)))....))))
```

---

## locus\_id: 248468

family\_id: 144

### **Targets:**

At5g06490(NM\_120732.1
): zinc finger (C3HC4-type RING finger) family protein

Location in genome: in an IGR, 52779 upstream of At3g42070, 5154 downstream of At3g41979

### Alignment between mature miRNA and predicted targets

```
        Extended mature miRNA:       ccgtgtccgaggagtggtcgagggttt
        Target(rc):NM_120732.1       tctTGTCCTAGGAGTGGTCGTGGGaga
```

### Precursor sequence and structure. Mature sequence in capital letters

```
tcgaccgTGTCCGAGGAGTGGTCGAGGGtttgtcgaccaggacgaggaatcgtcgaccgggtccgaggatttgtcgaccaggggttgaaatcgtcgaccaggtccgagacttcatcga   
 ((((....(((((.((..(((((((.((((..((((((.((.(((.(((((.(((........))).))))).))).))...)))))))))).)))))))..)))).)))....))))
```

---

## locus\_id: 143936

family\_id: 142

### **Targets:**

At1g30330(NM\_102771.2
): auxin-responsive factor (ARF6)

Location in genome: in an IGR, 3815 upstream of At2g02700, 709 downstream of At2g02690

7 homologs in brassica

### Alignment between mature miRNA and predicted targets

```
        Extended mature miRNA:       tcttgggttcgaggaacgccaagcgc
        Target(rc):NM_102771.2       tggTGGGTTTCAGGAACGCCAAGata
```

### Precursor sequence and structure. Mature sequence in capital letters

```
gtgcgtttggtattcctcgaacccaagacctcaccctttagattaattggtaacagtttgtcctgtgggaaggttgttaaagggtgaggtctTGGGTTCGAGGAACGCCAAGcgcac   
 ((((((((((..(((((((((((((((((((((((((((((..(((((....((((......)))).....))))))))))))))))))))))))))))))))))..))))))))))
```

---

## locus\_id: 391540

family\_id: 142

### **Targets:**

At1g30330(NM\_102771.2
): auxin-responsive factor (ARF6)

Location in genome: in an IGR, 6930 upstream of At5g24165, 17545 downstream of At5g24130

7 homologs in brassica

### Alignment between mature miRNA and predicted targets

```
        Extended mature miRNA:       tcttgggttcgaggaacgccaagcgc
        Target(rc):NM_102771.2       tggTGGGTTTCAGGAACGCCAAGata
```

### Precursor sequence and structure. Mature sequence in capital letters

```
attggtgcgcttggcgttcctcgaacccaagtcaattggtaacagcttgtcctttgggaaagttgttaaaggatgaggtctTGGGTTCGAGGAACGCCAAGcgcaccaat   
 (((((((((((((((((((((((((((((((.(.(((..(((((((((.(((....))))))))))))...)))...).)))))))))))))))))))))))))))))))
```

---

## locus\_id: 31349

family\_id: 141

### **Targets:**

At4g35080(NM\_119674.3
): high-affinity nickel-transport family protein  
At4g35080(NM\_202956.1
): high-affinity nickel-transport family protein

Location in genome: in an IGR, 6952 upstream of At1g48900, 6793 downstream of At1g48930

### Alignment between mature miRNA and predicted targets

```
        Extended mature miRNA:       ggatggattcccaaagcaccgatgcc
        Target(rc):NM_202956.1       tccTTGATTCCCATAGCACCGATaat
        Target(rc):NM_119674.3       tccTTGATTCCCATAGCACCGATaat
```

### Precursor sequence and structure. Mature sequence in capital letters

```
tgatctggcatcggtgctttggggaatccattcttcttcaccacgtattcatagtcctgtataatatcacaaaaataggaTGGATTCCCAAAGCACCGATgccaaagca   
 .....((((((((((((((((((.((((((((((..........(((((.(((......)))))))).........)))))))))))))))))))))))))))).....
```

---

## locus\_id: 337312

family\_id: 140

### **Targets:**

At4g25990(NM\_118732.1
): expressed protein  
At4g25990(NM\_179114.1
): expressed protein

Location in genome: in an IGR, 97 upstream of At5g10890, 1230 downstream of At5g10900

2 homologs in brassica

### Alignment between mature miRNA and predicted targets

```
        Extended mature miRNA:       cgatggatgacggtgtaggagtgaga
        Target(rc):NM_179114.1       ttgTAGATGACGGTGAAGGAGTGttg
        Target(rc):NM_118732.1       ttgTAGATGACGGTGAAGGAGTGttg
```

### Precursor sequence and structure. Mature sequence in capital letters

```
ctccgaTGGATGACGGTGTAGGAGTGagagcaggatgcatccggatcagatgtccgtcgccacggag   
 (((((.(((.(((((((((..((.((.((((.....)).))))..))..))).))))))))))))))
```

---

## locus\_id: 34573

family\_id: 135

### **Targets:**

At1g55750(NM\_104451.3
): transcription factor-related

Location in genome: in an IGR, 8117 upstream of At1g53650, 3057 downstream of At1g53690

### Alignment between mature miRNA and predicted targets

```
        Extended mature miRNA:       agaatgagattgagccaaggatgact
        Target(rc):NM_104451.3       ttgGTGAGATTGAGCCAAGGAGGttt
```

### Precursor sequence and structure. Mature sequence in capital letters

```
aaagaATGAGATTGAGCCAAGGATGacttgccgatgttatcaacaaatcttaactgattttggtgtccggcaagttgaccttggctctgtttccttcttt   
 ((((((.(((((.(((((((((.((((((((((....((((((..((((......))))))))))..)))))))))).))))))))).))))).))))))
```

---

## locus\_id: 87833

family\_id: 135

### **Targets:**

At1g55750(NM\_104451.3
): transcription factor-related

Location in genome: in an IGR, 6987 upstream of At1g53700, 2463 downstream of At1g53680

1 homologs in brassica

### Alignment between mature miRNA and predicted targets

```
        Extended mature miRNA:       aaatgagattgagccaaggatgactt
        Target(rc):NM_104451.3       tggTGAGATTGAGCCAAGGAGGTtta
```

### Precursor sequence and structure. Mature sequence in capital letters

```
gaaaaTGAGATTGAGCCAAGGATGActtgccgattttctcaacgaatcttactgattatggtatccggcaagttgactttggctctgtttccttc   
 ......(((((.(((((((((.((((((((((...(((.....)))...((((......))))..)))))))))).))))))))).)))))....
```

---

## locus\_id: 382055

family\_id: 131

### **Targets:**

At1g72990(NM\_105957.1
): glycosyl hydrolase family 35 protein

Location in genome: in an IGR, 883 upstream of At5g08190, 15449 downstream of At5g08141

### Alignment between mature miRNA and predicted targets

```
        Extended mature miRNA:       cgatgaaccgctgcctccagcgactc
        Target(rc):NM_105957.1       tggTGATCCACTGCCTCCAGCGAaag
```

### Precursor sequence and structure. Mature sequence in capital letters

```
gaggcaaacgctggatgcagaggtttatcgatctattcatgcttttacatttttaatgtgttcacaggaagagatcgaTGAACCGCTGCCTCCAGCGActctc   
 (((.....(((((((.((((.(((((((((((((.(((.((....(((((.....)))))....)).))).))))))))))))).)))).))))))).)))..
```

---

## locus\_id: 390808

family\_id: 131

### **Targets:**

At1g72990(NM\_105957.1
): glycosyl hydrolase family 35 protein

Location in genome: in an IGR, 1203 upstream of At5g23070, 12972 downstream of At5g23030

### Alignment between mature miRNA and predicted targets

```
        Extended mature miRNA:       cgatgaaccgctgcctccagcgactt
        Target(rc):NM_105957.1       tggTGATCCACTGCCTCCAGCGAaag
```

### Precursor sequence and structure. Mature sequence in capital letters

```
agagcaagcagcgctggatgcagaggtttatcgatcgattcttgcttttgtaaacaaaaataaatattcacaggaattgatcgaTGAACCGCTGCCTCCAGCGActtcactct   
 ((((.(((...(((((((.((((.((((((((((((((((((((..(((((........)))))......)))))))))))))))))))).)))).))))))).)))..))))
```

---

## locus\_id: 336441

family\_id: 123

### **Targets:**

At1g67030(NM\_105372.2
): zinc finger (C2H2 type) family protein (ZFP6)

Location in genome: in an IGR, 27305 upstream of At5g09380, 3976 downstream of At5g09460

10 homologs in brassica

### Alignment between mature miRNA and predicted targets

```
        Extended mature miRNA:       gttatccggctccggatccgggtccgt
        Target(rc):NM_105372.2       cggATCCTGATCCGGATCCGGGTCttg
```

### Precursor sequence and structure. Mature sequence in capital letters

```
ttgttATCCGGCTCCGGATCCGGGTCcgtctgatccataaatttgttatccgtattcagatccgtttagtccggatatccgatttttcggagcggatcacggatccggatccggatattcgcgg   
 ....(((((((.(((((((((((((((((((((.....(((((.(.((((((.(((.(((....)))))).)))))).).))))).))))).)))))).)))))))))).))))))).......
```

---

## locus\_id: 382602

family\_id: 123

### **Targets:**

At2g24780(NM\_128037.1
): hypothetical protein

Location in genome: in an IGR, 1442 upstream of At5g09450, 890 downstream of At5g09440

### Alignment between mature miRNA and predicted targets

```
        Extended mature miRNA:       aatatccggatccggatccgtgatcc
        Target(rc):NM_128037.1       aggATCCGCATCCGGATCCGTAAacc
```

### Precursor sequence and structure. Mature sequence in capital letters

```
ccgcgaatATCCGGATCCGGATCCGTGAtccgctccgaaaaatcggatatccggactaaacggatctgaatacggataacaaatttatggatcagacggacccggatccggagccgg   
 (((.(....(((((((((((.((((((((((..(((((....)))))..((((.......))))((((....))))............)))))..))))).))))))))))).))))
```

---

## locus\_id: 25939

family\_id: 121

### **Targets:**

At5g20870(NM\_122094.1
): glycosyl hydrolase family 17 protein  
At5g20870(NM\_122094.1
): glycosyl hydrolase family 17 protein

Location in genome: in an IGR, 165802 upstream of At1g38131, 48872 downstream of At1g38630

### Alignment between mature miRNA and predicted targets

```
        Extended mature miRNA:       cggtcaatccctcggtggcggatgga
        Target(rc):NM_122094.1       accTGAATTCCTCGGTGGCGGATtcg
```

### Precursor sequence and structure. Mature sequence in capital letters

```
ggatcggTCAATCCCTCGGTGGCGGATggatttcttcaacagatgactggtggatcgatcaatccccacctgggagcgatcgatcc   
 (((((((((..((((..(((((.((((.((((..((((.(((....))).))))..)))).))))))))).))))..)))))))))
```

---

## locus\_id: 25969

family\_id: 121

### **Targets:**

At5g20870(NM\_122094.1
): glycosyl hydrolase family 17 protein

Location in genome: in an IGR, 169554 upstream of At1g38131, 45120 downstream of At1g38630

### Alignment between mature miRNA and predicted targets

```
        Extended mature miRNA:       cggtcaatccctcggtggcggatgga
        Target(rc):NM_122094.1       accTGAATTCCTCGGTGGCGGATtcg
```

### Precursor sequence and structure. Mature sequence in capital letters

```
ggatcggTCAATCCCTCGGTGGCGGATggatttcttcaacagatgactggtggatcgatcgatccccacctgggagcgatcgatcc   
 (((((((((..((((..(((((.((((.((((..((((.(((....))).))))..)))).))))))))).))))..)))))))))
```

---

## locus\_id: 357437

family\_id: 119

### **Targets:**

At2g38860(NM\_129443.1
): proteaseI (pfpI)-like protein (YLS5)  
At2g38860(NM\_179972.1
): proteaseI (pfpI)-like protein (YLS5)

Location in genome: in an IGR, 1148 upstream of At5g38280, 12191 downstream of At5g38310

### Alignment between mature miRNA and predicted targets

```
        Extended mature miRNA:       agctacggttgggtgaaatgcaacac
        Target(rc):NM_129443.1       cgtTGCGGTTGGGTGAAACGCAAtga
        Target(rc):NM_179972.1       cgtTGCGGTTGGGTGAAACGCAAtga
```

### Precursor sequence and structure. Mature sequence in capital letters

```
acccccaagcTACGGTTGGGTGAAATGCAAcacagatggtgcctgggttgatgtagctccgaactgtggcgttggatgggt   
 (((((((((((((((((.((.(..(((((((.(((.......))).)))).)))..).)).))))))))).))))..))))
```

---

## locus\_id: 151914

family\_id: 118

### **Targets:**

At5g58000(NM\_125185.2
): phosphatase-related

Location in genome: in an IGR, 15482 upstream of At2g12100, 53726 downstream of At2g11890

### Alignment between mature miRNA and predicted targets

```
        Extended mature miRNA:       gttgttgatgcagtgactcattgaggta
        Target(rc):NM_125185.2       tgaGTTGATGCAGAGACTCGTTGAGata
```

### Precursor sequence and structure. Mature sequence in capital letters

```
cttaccttgagggtggtggatggatcgccagagaagagagctctgagttGTTGATGCAGTGACTCATTGAGgtaag   
 (((((((..(((((.(((.((.(((.(((((((.......))))).)).))).)).)).).)))).)..)))))))
```

---

## locus\_id: 246653

family\_id: 118

### **Targets:**

At5g58000(NM\_125185.2
): phosphatase-related

Location in genome: in an IGR, 37890 upstream of At3g32394, 35618 downstream of At3g32330

### Alignment between mature miRNA and predicted targets

```
        Extended mature miRNA:       gttgttgatgcagtgactcattgaggta
        Target(rc):NM_125185.2       tgaGTTGATGCAGAGACTCGTTGAGata
```

### Precursor sequence and structure. Mature sequence in capital letters

```
cttaccttgagggtggtggatggatagccagagaagagagctctgagttGTTGATGCAGTGACTCATTGAGgtaag   
 (((((((..(((((.(((.((.(((((((((((.......))))).)))))).)).)).).)))).)..)))))))
```

---

## locus\_id: 334232

family\_id: 116

### **Targets:**

At2g32250(NM\_201851.1
): far-red impaired responsive protein, putative  
At2g32250(NM\_128784.3
): far-red impaired responsive protein, putative

Location in genome: in an IGR, 151 upstream of At5g05510, 6293 downstream of At5g05550

### Alignment between mature miRNA and predicted targets

```
        Extended mature miRNA:       ccggttgaccgattagccggttagctt
        Target(rc):NM_128784.3       tctGTTAACCGATTAACCGGTTAGaca
        Target(rc):NM_201851.1       tctGTTAACCGATTAACCGGTTAGaca
```

### Precursor sequence and structure. Mature sequence in capital letters

```
ggctaaccggctgagtgattagctggttatctgattatctggttaaccgGTTGACCGATTAGCCGGTTAGct   
 ((((((((((((((.((.(((((((((((.((........)).))))))))))).)).))))))))))))))
```

---

## locus\_id: 162692

family\_id: 114

### **Targets:**

At3g19670(NM\_112857.2
): FF domain-containing protein / WW domain-containing protein

Location in genome: in an IGR, 7586 upstream of At2g25110, 6517 downstream of At2g25080

### Alignment between mature miRNA and predicted targets

```
        Extended mature miRNA:       ttgcctttgtgtgctcactctcttctgtc
        Target(rc):NM_112857.2       cttCCTTTGTGTGCTCCCTCGCTTCTtct
```

### Precursor sequence and structure. Mature sequence in capital letters

```
gaatctgacagaaagagcagtgagcacgcaagagaagcaagtgcaatgatatgcaaattgCCTTTGTGTGCTCACTCTCTTCTgtcagtttc   
 (((.(((((((((.(((.((((((((((((((....((((.((((......))))..)))).)))))))))))))))))))))))))).)))
```

---

## locus\_id: 337349

family\_id: 114

### **Targets:**

At4g31877(NM\_202928.1
): expressed protein

Location in genome: in an IGR, 2464 upstream of At5g10940, 2597 downstream of At5g10950

3 homologs in brassica

### Alignment between mature miRNA and predicted targets

```
        Extended mature miRNA:       ggttgcttttgcgtgctcactctcttttt
        Target(rc):NM_202928.1       aagTGCCTTTGTGTGCTCACTCTCTTctg
```

### Precursor sequence and structure. Mature sequence in capital letters

```
tgacagaagagagtgagcacacaaaggggaagttgtataaaagttttgtatatggtTGCTTTTGCGTGCTCACTCTCTTtttgtca   
 .(((((((((((((((((((.((((((.((...(((((((.....)))))))...)).)))))).)))))))))))).))))))).
```

---

## locus\_id: 393204

family\_id: 114

### **Targets:**

At4g31877(NM\_202928.1
): expressed protein

Location in genome: in an IGR, 7061 upstream of At5g26160, 4553 downstream of At5g26140

1 homologs in brassica

1 homologs in rice

### Alignment between mature miRNA and predicted targets

```
        Extended mature miRNA:       aagctatgtgtgctcactctctatcc
        Target(rc):NM_202928.1       tgcCTTTGTGTGCTCACTCTCTTctg
```

### Precursor sequence and structure. Mature sequence in capital letters

```
ggtgacagaagagagtgagcacacatggtggctttcttgcatatttgaaggttccatgcttgaagCTATGTGTGCTCACTCTCTAtccgtcacc   
 ((((((.((((((((((((((((((((((....(((..((((.............))))..))))))))))))))))))))))).)).))))))
```

---

## locus\_id: 393205

family\_id: 114

### **Targets:**

At2g23350(NM\_127899.2
): polyadenylate-binding protein, putative / PABP, putative

Location in genome: in an IGR, 7057 upstream of At5g26160, 4557 downstream of At5g26140

1 homologs in rice

### Alignment between mature miRNA and predicted targets

```
        Extended mature miRNA:       tatgtgtgctcactctctatccgtca
        Target(rc):NM_127899.2       gcaGCGTGCTCACTCTCTATCTGgtc
```

### Precursor sequence and structure. Mature sequence in capital letters

```
ggtgacagaagagagtgagcacacatggtggctttcttgcatatttgaaggttccatgcttgaagctatGTGTGCTCACTCTCTATCCGtcacc   
 ((((((.((((((((((((((((((((((....(((..((((.............))))..))))))))))))))))))))))).)).))))))
```

---

## locus\_id: 39260

family\_id: 113

### **Targets:**

At1g60110(NM\_104703.1
): jacalin lectin family protein

Location in genome: in an IGR, 13692 upstream of At1g61200, 12018 downstream of At1g61255

### Alignment between mature miRNA and predicted targets

```
        Extended mature miRNA:       atggtcggattcatcatcccgcttatag
        Target(rc):NM_104703.1       tttGTCGGATCCATCATCCCACTTAgat
```

### Precursor sequence and structure. Mature sequence in capital letters

```
agaaaacataagtgggatgacggatctgaccatgatggtgtttcgatccctggacaataactacatcatacataaatttctgcaacaccatcatgGTCGGATTCATCATCCCGCTTAtagcctct   
 .......(((((((((((((.(((((((((((((((((((((.(((................................)).).))))))))))))))))))))).))))))))))))).......
```

---

## locus\_id: 233846

family\_id: 112

### **Targets:**

At3g19330(NM\_202611.1
): polyadenylate-binding protein-related / PABP-related  
At3g19330(NM\_112820.2
): polyadenylate-binding protein-related / PABP-related  
At3g19330(NM\_202610.1
): polyadenylate-binding protein-related / PABP-related

Location in genome: in an IGR, 20329 upstream of At3g19290, 234 downstream of At3g19240

### Alignment between mature miRNA and predicted targets

```
        Extended mature miRNA:       gttgtcctctcgcataagctcaaaaa
        Target(rc):NM_202610.1       gtaGTCCTCTCTCATAAGCTCAGgat
        Target(rc):NM_202611.1       gtaGTCCTCTCTCATAAGCTCAGgat
        Target(rc):NM_112820.2       gtaGTCCTCTCTCATAAGCTCAGgat
```

### Precursor sequence and structure. Mature sequence in capital letters

```
ggtcctgttGTCCTCTCGCATAAGCTCAAaaaaccactgatacaaataggttgaggagcttacgtttggggacgaaggatc   
 ((((((.((((((((..((.(((((((....((((.............))))...))))))).))..))))))))))))))
```

---

## locus\_id: 248359

family\_id: 111

### **Targets:**

At4g17070(NM\_117811.2
): expressed protein

Location in genome: in an IGR, 5546 upstream of At3g41768, 84601 downstream of At3g33230

### Alignment between mature miRNA and predicted targets

```
        Extended mature miRNA:       cgggtccgaggattcgtcgaccagga
        Target(rc):NM_117811.2       agaGTCCGAGGATTCGTTGACGAaga
```

### Precursor sequence and structure. Mature sequence in capital letters

```
ttgtcgaccaggacgaggaatcgtcgaccgggtccgaggatttgtcgaccaggggttgaaatcatcgaccaggtccgagacttcatcgaccggGTCCGAGGATTCGTCGACCAggacgatga   
 (..(((.((.((.(((.(((((.((((((.((((.((.((..(.((((((...((((((.....)))))).))).))).)..)).)))))).))).))).))))).))).)).)).)))..)
```

---

## locus\_id: 248388

family\_id: 111

### **Targets:**

At4g17070(NM\_117811.2
): expressed protein

Location in genome: in an IGR, 2881 upstream of At3g41768, 87266 downstream of At3g33230

### Alignment between mature miRNA and predicted targets

```
        Extended mature miRNA:       cgggtccgaggattcgtcgaccagga
        Target(rc):NM_117811.2       agaGTCCGAGGATTCGTTGACGAaga
```

### Precursor sequence and structure. Mature sequence in capital letters

```
ttgtcgaccaggacgaggaatcgtcgaccgggtccgaggatttgtcgaccaggggttgaaatcgtcgaccaggtccgagacttcatcgaccggGTCCGAGGATTCGTCGACCAggacgatga   
 (..(((.((.((.(((.(((((.((((((.((((.((.((..(.((((((...((((((.....)))))).))).))).)..)).)))))).))).))).))))).))).)).)).)))..)
```

---

## locus\_id: 209045

family\_id: 109

### **Targets:**

At2g14000(NM\_126972.1
): hypothetical protein

Location in genome: in an IGR, 48764 upstream of At3g44520, 587 downstream of At3g44580

### Alignment between mature miRNA and predicted targets

```
        Extended mature miRNA:       ggtaacaaggtactgctcgttaggtttga
        Target(rc):NM_126972.1       aggAACAAGGTACTGCTCGTTATGTTcga
```

### Precursor sequence and structure. Mature sequence in capital letters

```
tagtgtttgaacctaacgagcagtactttgttcctaacccttctgagaccgtgttagattagtagacaagtgacagtctaacactaggtAACAAGGTACTGCTCGTTAGGTTtgaacacta   
 (((((((..(((((((((((((((((((((((((((...((....))...((((((((((.((.........)))))))))))))))).)))))))))))))))))))))))..)))))))
```

---

## locus\_id: 252331

family\_id: 109

### **Targets:**

At2g14000(NM\_126972.1
): hypothetical protein

Location in genome: in an IGR, 3807 upstream of At3g44590, 154 downstream of At3g44570

### Alignment between mature miRNA and predicted targets

```
        Extended mature miRNA:       aaagtactgctcgttaggttcaaacactagg
        Target(rc):NM_126972.1       aagGTACTGCTCGTTATGTTCGAACACTggg
```

### Precursor sequence and structure. Mature sequence in capital letters

```
gaacctagtgttcaaacctaacgagcagtaccttgttacctagtgttagactgtcacttgtctactaatctaacacggtctcagaagggttaggaacaaaGTACTGCTCGTTAGGTTCAAACACTaggttc   
 ((((((((((((..(((((((((((((((((.(((((.((((((((((((..((.........))...))))))))...(((....))).))))))))).)))))))))))))))))..))))))))))))
```

---

## locus\_id: 271458

family\_id: 107

### **Targets:**

At4g32915(NM\_119445.2
): expressed protein

Location in genome: in an IGR, 318029 upstream of At4g06526, 26962 downstream of At4g06599

### Alignment between mature miRNA and predicted targets

```
        Extended mature miRNA:       tggttctcctctcatagctggctccctc
        Target(rc):NM_119445.2       catTTCTGCTCTAATAGCTGGCTCCaca
```

### Precursor sequence and structure. Mature sequence in capital letters

```
ggacatgagcctgcataggatgcggcggaggcggaggtggatagtgaggcggaactaccatatccggttcacgctgtggagcattcacctctggTTCTCCTCTCATAGCTGGCTCCctcc   
 (((...(((((.((.((.((.(.((.((((.((((((((((..(((((.((((.........)))).)))))(((....))).)))))))))).)))))).))).)))).)))))..)))
```

---

## locus\_id: 151726

family\_id: 104

### **Targets:**

At1g03940(NM\_100275.2
): transferase family protein

Location in genome: in an IGR, 32224 upstream of At2g11890, 23773 downstream of At2g11810

### Alignment between mature miRNA and predicted targets

```
        Extended mature miRNA:       ccatggcatggaagaaggtgagactt
        Target(rc):NM_100275.2       aggGGGCATCGAAGAAGGTGAGAgga
```

### Precursor sequence and structure. Mature sequence in capital letters

```
gtgatggccaTGGCATGGAAGAAGGTGAGActtgcctgaatcgtgaatcaaaacctccatgattatcactacaagtcgtacctttttcctagccatgataatcac   
 (((((...((((((..(((((((((((.((((((..(((((((((............))))))..)))...)))))).)))))))))))..))))))...)))))
```

---

## locus\_id: 277415

family\_id: 104

### **Targets:**

At1g03940(NM\_100275.2
): transferase family protein

Location in genome: in an IGR, 25138 upstream of At4g11090, 3392 downstream of At4g11140

### Alignment between mature miRNA and predicted targets

```
        Extended mature miRNA:       catggcatggaagaaggtgagacttg
        Target(rc):NM_100275.2       gggGGCATCGAAGAAGGTGAGAGgaa
```

### Precursor sequence and structure. Mature sequence in capital letters

```
gatggccatGGCATGGAAGAAGGTGAGACttgcatgaatcgtgaatcaaaacctccatgattatcactgcaagtcgtacctttttcctagccatgataatc   
 (((...((((((..(((((((((((.(((((((((((((((((............))))))..))).)))))))).)))))))))))..))))))...)))
```

---

## locus\_id: 20495

family\_id: 103

### **Targets:**

At5g33370(NM\_122861.2
): GDSL-motif lipase/hydrolase family protein

Location in genome: in an IGR, 2044 upstream of At1g32210, 15907 downstream of At1g32240

### Alignment between mature miRNA and predicted targets

```
        Extended mature miRNA:       tccggattcgaatacccgcggataac
        Target(rc):NM_122861.2       taaGGATTCCAATACCCGCGGAGgcg
```

### Precursor sequence and structure. Mature sequence in capital letters

```
tccgtcgcacccggttattcggttttctggatcggatcacggatccGGATTCGAATACCCGCGGATaacgga   
 (((((....(((((.(((((((...((((((((.(....).)))))))).))))))).))).))...)))))
```

---

## locus\_id: 162473

family\_id: 103

### **Targets:**

At5g33370(NM\_122861.2
): GDSL-motif lipase/hydrolase family protein

Location in genome: in an IGR, 335 upstream of At2g24790, 1349 downstream of At2g24765

### Alignment between mature miRNA and predicted targets

```
        Extended mature miRNA:       tccggattcgaatacccgcggataac
        Target(rc):NM_122861.2       taaGGATTCCAATACCCGCGGAGgcg
```

### Precursor sequence and structure. Mature sequence in capital letters

```
atccagatccgccggactcgtatccagatccgccggactcgtatccggatccgtcgcacctaattattcggttttccggatcggatcacggatccGGATTCGAATACCCGCGGATaacggat   
 ((((..((((((.((..(((.((((.((((((...((..((.(((((((.(((...............)))...)))))))))..)).)))))).)))).)))...)).))))))...))))
```

---

## locus\_id: 29702

family\_id: 100

### **Targets:**

At1g27580(NM\_102522.1
): F-box family protein

Location in genome: in an IGR, 908 upstream of At1g47370, 2907 downstream of At1g47380

### Alignment between mature miRNA and predicted targets

```
        Extended mature miRNA:       gctgcttttggtcgctgaatccagcg
        Target(rc):NM_102522.1       ctgGCTTTTGGCCGCTGAATCAAatt
```

### Precursor sequence and structure. Mature sequence in capital letters

```
tgtcgctggttcaacgaccaaaagtagcgaccagcgaccgcaatttttgatcgctgaaatttttagcgatcagtcgctggtttcagcgattagtcgctGCTTTTGGTCGCTGAATCCAgcgaca   
 .((((((((((((.(((((((((((((((((..((....))......(((((((((((((...((((((....)))))))))))))))))))))))))))))))))))).)))).)))))))).
```

---

## locus\_id: 78525

family\_id: 100

### **Targets:**

At1g27580(NM\_102522.1
): F-box family protein

Location in genome: in an IGR, 5046 upstream of At1g37000, 4032 downstream of At1g36980

### Alignment between mature miRNA and predicted targets

```
        Extended mature miRNA:       gctgcttttggtcgctgaatccagcg
        Target(rc):NM_102522.1       ctgGCTTTTGGCCGCTGAATCAAatt
```

### Precursor sequence and structure. Mature sequence in capital letters

```
gcttgtcgctggttcagcgatcaaaagtagcgacagtcgccagggaccagcgaccgtaattttttgtcgttaaaatttttagcgattagtcgctGCTTTTGGTCGCTGAATCCAgcgacatgc   
 ((.(((((((((((((((((((((((((((((((((((((.(((((..((((((...........))))))....))))).)))))).)))))))))))))))))))))).))))))))).))
```

---

## locus\_id: 317413

family\_id: 99

### **Targets:**

At1g48410(NM\_179453.1
): argonaute protein (AGO1)  
At1g48410(NM\_103737.2
): argonaute protein (AGO1)

Location in genome: in an IGR, 27780 upstream of At4g19450, 13248 downstream of At4g19360

### Alignment between mature miRNA and predicted targets

```
        Extended mature miRNA:       ttcgcttggtgcaggtcgggaaccaa
        Target(rc):NM_179453.1       gtaGCTTGATGCAGCTCGGGAACtga
        Target(rc):NM_103737.2       gtaGCTTGATGCAGCTCGGGAACtga
```

### Precursor sequence and structure. Mature sequence in capital letters

```
tcggattcGCTTGGTGCAGGTCGGGAACcaattcggctgacacagcctcgtgacttttaaacctttattggtttgtgagcagggattggatcccgccttgcatcaactgaatcgga   
 ((.((((((.((((((((((.(((((.((((((((((((...)))))...((.(((.((((((......)))))).))))).))))))).))))).)))))))))).)))))).))
```

---

## locus\_id: 371738

family\_id: 98

### **Targets:**

At5g59130(NM\_125303.1
): subtilase family protein

Location in genome: in an IGR, 2035 upstream of At5g58800, 16488 downstream of At5g58860

### Alignment between mature miRNA and predicted targets

```
        Extended mature miRNA:       gtggtgctggctgcaacggttaagat
        Target(rc):NM_125303.1       gctGTGCTGGCTGCAACAGTTAAcag
```

### Precursor sequence and structure. Mature sequence in capital letters

```
tgtgGTGCTGGCTGCAACGGTTAAgatccacggtgctacgctcacggttggattcggaccattgttaccagctgactgca   
 .(..(((((((..((((.((((..(((((((.(((.......))).).))))))..)))).))))..)))))..))..).
```

---

## locus\_id: 202769

family\_id: 97

### **Targets:**

At2g22670(NM\_179697.2
): auxin-responsive protein / indoleacetic acid-induced protein 8 (IAA8)  
At2g22670(NM\_127832.2
): auxin-responsive protein / indoleacetic acid-induced protein 8 (IAA8)

Location in genome: in an IGR, 42530 upstream of At3g32200, 142918 downstream of At3g32400

### Alignment between mature miRNA and predicted targets

```
        Extended mature miRNA:       gcagcgttggcagctggtgcatgggca
        Target(rc):NM_179697.2       tgtGCCTTGGCAGCTGGTGCATTGttc
        Target(rc):NM_127832.2       tgtGCCTTGGCAGCTGGTGCATTGttc
```

### Precursor sequence and structure. Mature sequence in capital letters

```
gcatgcgcatgcctagtattcaacgcggctaaacgagcagcattagcagcagctaaacaggcaGCGTTGGCAGCTGGTGCATGGgcagc   
 ((.(((.((((((((((..(((((((.(((.....(((.((.......)).))).....))).)))))))..))))).))))).)))))
```

---

## locus\_id: 268523

family\_id: 95

### **Targets:**

At1g72080(NM\_105866.1
): hypothetical protein

Location in genome: in an IGR, 21516 upstream of At4g04070, 140410 downstream of At4g04330

### Alignment between mature miRNA and predicted targets

```
        Extended mature miRNA:       accgccacgtccacgtccacgacctc
        Target(rc):NM_105866.1       acgCCCACGTCCACGACCACGACtac
```

### Precursor sequence and structure. Mature sequence in capital letters

```
gccggtaccGCCACGTCCACGTCCACGACctctaccattgttgttgtgcccattgtagttgttgtggttgttgtcgcggtagccggt   
 (((((((((((.(((...(((.(((((((..((((....((......)).....))))..))))))).)))))).))))).))))))
```

---

## locus\_id: 268524

family\_id: 95

### **Targets:**

At1g66260(NM\_105297.2
): RNA and export factor-binding protein, putative

Location in genome: in an IGR, 21518 upstream of At4g04070, 140408 downstream of At4g04330

### Alignment between mature miRNA and predicted targets

```
        Extended mature miRNA:       gtaccgccacgtccacgtccacgacc
        Target(rc):NM_105297.2       ggtCCACCACGTCCACGTCCACTctt
```

### Precursor sequence and structure. Mature sequence in capital letters

```
gccggtaCCGCCACGTCCACGTCCACGacctctaccattgttgttgtgcccattgtagttgttgtggttgttgtcgcggtagccggt   
 (((((((((((.(((...(((.(((((((..((((....((......)).....))))..))))))).)))))).))))).))))))
```

---

## locus\_id: 142317

family\_id: 94

### **Targets:**

At1g56000(NM\_104478.3
): amine oxidase-related

Location in genome: in an IGR, 4052 upstream of At2g47570, 3371 downstream of At2g47600

2 homologs in brassica

### Alignment between mature miRNA and predicted targets

```
        Extended mature miRNA:       tttgcacgtgccctgcttctccaacat
        Target(rc):NM_104478.3          GCACGTACCCTGCTTCTCCAAttt
```

### Precursor sequence and structure. Mature sequence in capital letters

```
ctcatgttggagaagttaagtacgtgcaaataagcaaatgagacggatttcgtgtttgttggtttGCACGTGCCCTGCTTCTCCAAcatggag   
 .(((((((((((((((...((((((((((((.(((((((...(((.....)))))))))).))))))))))))...)))))))))))))))..
```

---

## locus\_id: 348182

family\_id: 94

### **Targets:**

At1g56000(NM\_104478.3
): amine oxidase-related

Location in genome: in an IGR, 10681 upstream of At5g27790, 10221 downstream of At5g27840

1 homologs in brassica

### Alignment between mature miRNA and predicted targets

```
        Extended mature miRNA:       ttcgcacgtgccctgcttctccatca
        Target(rc):NM_104478.3          GCACGTACCCTGCTTCTCCAatt
```

### Precursor sequence and structure. Mature sequence in capital letters

```
gacacgtgttggagtagtagaacacgtgcgaaaatatgatcaacaagtaccgatcgatttcatttgtgttcGCACGTGCCCTGCTTCTCCAtcaagtgtt   
 (((((.((.(((((.(((((..((((((((((.((((((((..........)))))........))).))))))))))..))))).))))).)).)))))
```

---

## locus\_id: 257133

family\_id: 93

### **Targets:**

At1g27020(NM\_102464.2
): expressed protein

Location in genome: in an IGR, 1772 upstream of At3g51640, 358 downstream of At3g51630

### Alignment between mature miRNA and predicted targets

```
        Extended mature miRNA:       agagcaaagggccatcgccaatagtt
        Target(rc):NM_102464.2       ttgGCAAAGGGCCATTGCCAATAccg
```

### Precursor sequence and structure. Mature sequence in capital letters

```
gagaGCAAAGGGCCATCGCCAATAgttccacttgtgcttgtggtactcttcggatggttcctatgcggctc   
 (((.(((.(((((((((.(.((.(((.((((........)))).))).)).)))))).)))).)))..)))
```

---

## locus\_id: 257146

family\_id: 93

### **Targets:**

At1g27020(NM\_102464.2
): expressed protein

Location in genome: in an IGR, 1839 upstream of At3g51650, 318 downstream of At3g51640

### Alignment between mature miRNA and predicted targets

```
        Extended mature miRNA:       agagcaaagggccatcgccaatagtt
        Target(rc):NM_102464.2       ttgGCAAAGGGCCATTGCCAATAccg
```

### Precursor sequence and structure. Mature sequence in capital letters

```
gagaGCAAAGGGCCATCGCCAATAgttccacttgtgcttgtggtactcttcggatggttcctatgcggctc   
 (((.(((.(((((((((.(.((.(((.((((........)))).))).)).)))))).)))).)))..)))
```

---

## locus\_id: 55822

family\_id: 90

### **Targets:**

At3g01660(NM\_111032.2
): expressed protein

Location in genome: Contained by At1g05500 (NM\_100429: . C2 domain-containing protein) in an intron

1 homologs in brassica

### Alignment between mature miRNA and predicted targets

```
        Extended mature miRNA:       ttgcgaggatgacggtggaggattcc
        Target(rc):NM_111032.2       ggcCGAGGATGATGGTCGAGGATcgg
```

### Precursor sequence and structure. Mature sequence in capital letters

```
gctgcttttgCGAGGATGACGGTGGAGGATtccaggaagcttttgcctcctgagttttatccttcctgggttgtcttctccgagcgtc   
 ...((((..(.(((((..((((.(((((((.((((((.((....)).))))).)....)))))))))..))..))))).).))))...
```

---

## locus\_id: 55823

family\_id: 90

### **Targets:**

At1g49750(NM\_103862.2
): leucine-rich repeat family protein

Location in genome: Contained by At1g05500 (NM\_100429: . C2 domain-containing protein) in an intron

1 homologs in brassica

### Alignment between mature miRNA and predicted targets

```
        Extended mature miRNA:       tgcgaggatgacggtggaggattcca
        Target(rc):NM_103862.2       ggtGAAGGTGACGGTGGAGGATTgtt
```

### Precursor sequence and structure. Mature sequence in capital letters

```
gcttttgcGAGGATGACGGTGGAGGATTccaggaagcttttgcctcctgagttttatccttcctgggttgtcttctccgagc   
 ((((..(.(((((..((((.(((((((.((((((.((....)).))))).)....)))))))))..))..))))).).))))
```

---

## locus\_id: 251897

family\_id: 89

### **Targets:**

At4g17190(NM\_202836.1
): farnesyl pyrophosphate synthetase 2 (FPS2) / FPP synthetase 2 / farnesyl diphosphate synthase 2  
At4g17190(NM\_117823.2
): farnesyl pyrophosphate synthetase 2 (FPS2) / FPP synthetase 2 / farnesyl diphosphate synthase 2

Location in genome: Contained by At3g44250 (NM\_114293: . cytochrome P450 family protein) in an intron on the reverse strand

### Alignment between mature miRNA and predicted targets

```
        Extended mature miRNA:       tttgaggaaagtctttttctccttcacgg
        Target(rc):NM_117823.2       tgaGAGGAAAGTCTCTTTCTCCGTCAagt
        Target(rc):NM_202836.1       tgaGAGGAAAGTCTCTTTCTCCGTCAagt
```

### Precursor sequence and structure. Mature sequence in capital letters

```
agatttGAGGAAAGTCTTTTTCTCCTTCAcggctagcatcaacaaaatcgcattggtggaagttttgtccaaaagcaagtctacaaatgatgctagccgtgaaggagaaaaggactttcctcaaatct   
 (((((((((((((((((((((((((((((((((((((((((.(((.......)))(((((.(((((.....)))))...)))))...)))))))))))))))))))))))))))))))))))))))))
```

---

## locus\_id: 201721

family\_id: 88

### **Targets:**

At4g36540(NM\_179177.1
): basic helix-loop-helix (bHLH) family protein  
At4g36540(NM\_119817.2
): basic helix-loop-helix (bHLH) family protein

Location in genome: in an IGR, 3512 upstream of At3g31330, 79170 downstream of At3g31410

### Alignment between mature miRNA and predicted targets

```
        Extended mature miRNA:       ctagagctcttctctcccggctgtct
        Target(rc):NM_179177.1       tttGAGCTCTTCTCTCCCATCTGagc
        Target(rc):NM_119817.2       tttGAGCTCTTCTCTCCCATCTGagc
```

### Precursor sequence and structure. Mature sequence in capital letters

```
tcgctaGAGCTCTTCTCTCCCGGCTGtctcctgctcctgcctaagcgatggcctggagagtgctctagtgg   
 .(((((((((....((((((.(((((((.(..((....))....).))))))).)))))).))))))))).
```

---

## locus\_id: 366535

family\_id: 87

### **Targets:**

At5g18510(NM\_121856.1
): hypothetical protein

Location in genome: in an IGR, 3282 upstream of At5g50500, 1528 downstream of At5g50510

### Alignment between mature miRNA and predicted targets

```
        Extended mature miRNA:       cacgagccatcgatggagctagacac
        Target(rc):NM_121856.1       acaGAGCCATCGATGGAGGTAGAaaa
```

### Precursor sequence and structure. Mature sequence in capital letters

```
gtatccggctccattgatgactagtgttgtttcatggacttcatgctaattcattgttcacaggagtaacacGAGCCATCGATGGAGCTAGAcac   
 ((.((..((((((((((((.((.((((((((((.(((((...(((......))).)))))..)))))))))).)).))))))))))))..)).))
```

---

## locus\_id: 366588

family\_id: 87

### **Targets:**

At5g18510(NM\_121856.1
): hypothetical protein

Location in genome: in an IGR, 7492 upstream of At5g50600, 1529 downstream of At5g50620

### Alignment between mature miRNA and predicted targets

```
        Extended mature miRNA:       cacgagccatcgatggagctagacac
        Target(rc):NM_121856.1       acaGAGCCATCGATGGAGGTAGAaaa
```

### Precursor sequence and structure. Mature sequence in capital letters

```
gtatccggctccattgatgactagtgttgtttcatggacttcatgctaattcattgttcacaggagtaacacGAGCCATCGATGGAGCTAGAcac   
 ((.((..((((((((((((.((.((((((((((.(((((...(((......))).)))))..)))))))))).)).))))))))))))..)).))
```

---

## locus\_id: 333459

family\_id: 84

### **Targets:**

At5g67180(NM\_126118.2
): AP2 domain-containing transcription factor, putative

Location in genome: in an IGR, 3495 upstream of At5g04270, 14971 downstream of At5g04300

1 homologs in brassica

1 homologs in rice

### Alignment between mature miRNA and predicted targets

```
        Extended mature miRNA:       tatgagaatcttgatgatgctgcatc
        Target(rc):NM_126118.2       ggaGAGAATCCTGATGATGCTGCcat
```

### Precursor sequence and structure. Mature sequence in capital letters

```
ttgtaggcgcagcaccattaagattcacatggaaattgataaataccctaaattagggttttgatatgtatatGAGAATCTTGATGATGCTGCatcaacaa   
 .(((.(..((((((.(((((((((((.((((...((...((((.((((((...))))))))))..))...)))).))))))))))).))))))..).))).
```

---

## locus\_id: 418146

family\_id: 84

### **Targets:**

At4g36920(NM\_119856.2
): floral homeotic protein APETALA2 (AP2)

Location in genome: in an IGR, 1486 upstream of At5g59510, 1186 downstream of At5g59500

### Alignment between mature miRNA and predicted targets

```
        Extended mature miRNA:       tgggaatcttgatgatgctgcatcagca
        Target(rc):NM_119856.2       agaGAATCCTGATGATGCTGCAGCAttt
```

### Precursor sequence and structure. Mature sequence in capital letters

```
gtagtcgcagatgcagcaccattaagattcacaagagatgtggttccctttgctttcgcctctcgatccgcagaaaagggttccttatcgagtggGAATCTTGATGATGCTGCATCAgcaaatac   
 ......((.(((((((((.(((((((((((.((...((((.((..((((((((..(((.....)))...)))...)))))..)).))))...)).))))))))))).))))))))).))......
```

---

## locus\_id: 107901

family\_id: 83

### **Targets:**

At5g11350(NM\_121173.2
): endonuclease/exonuclease/phosphatase family protein

Location in genome: in an IGR, 51689 upstream of At2g02570, 6077 downstream of At2g02710

### Alignment between mature miRNA and predicted targets

```
        Extended mature miRNA:       tcctcgaacccaagacctcacccttt
        Target(rc):NM_121173.2       gctTTGAACCCAAGACCTCAACCatc
```

### Precursor sequence and structure. Mature sequence in capital letters

```
ttggcgttccTCGAACCCAAGACCTCACCCtttaacaaccttcccacaggacaaactgttaccaattaatctaaagggtgaggtcttgggttcgaggaataccaa   
 .(((..((((((((((((((((((((((((((((...........((((......))))............))))))))))))))))))))))))))))..))).
```

---

## locus\_id: 143934

family\_id: 83

### **Targets:**

At5g11350(NM\_121173.2
): endonuclease/exonuclease/phosphatase family protein

Location in genome: in an IGR, 3889 upstream of At2g02700, 635 downstream of At2g02690

### Alignment between mature miRNA and predicted targets

```
        Extended mature miRNA:       ctcgaacccaagacctcaccctttag
        Target(rc):NM_121173.2       tttGAACCCAAGACCTCAACCATcta
```

### Precursor sequence and structure. Mature sequence in capital letters

```
ggtattcctcGAACCCAAGACCTCACCCTTtagattaattggtaacagtttgtcctgtgggaaggttgttaaagggtgaggtcttgggttcgaggaacgcc   
 ((..(((((((((((((((((((((((((((((..(((((....((((......)))).....))))))))))))))))))))))))))))))))))..))
```

---

## locus\_id: 87783

family\_id: 80

### **Targets:**

At5g09440(NM\_120981.2
): phosphate-responsive protein, putative

Location in genome: in an IGR, 13639 upstream of At1g53660, 13829 downstream of At1g53600

### Alignment between mature miRNA and predicted targets

```
        Extended mature miRNA:       acttgcggtgaaggcgacggtggaat
        Target(rc):NM_120981.2       ggcTGCGGTAAAGCCGACGGTGGcgg
```

### Precursor sequence and structure. Mature sequence in capital letters

```
ttccttgtacgttttccccccttgtgccgaagacgagggaggctttttcttgtcgattttggccactTGCGGTGAAGGCGACGGTGGaa   
 .(((((((.(((((((.((.(..((((((((((((((..((...))..))))))...))))).)))..).)).))))))))))).))).
```

---

## locus\_id: 40841

family\_id: 77

### **Targets:**

At2g33770(NM\_179887.1
): ubiquitin-conjugating enzyme family protein  
At2g33770(NM\_179887.1
): ubiquitin-conjugating enzyme family protein  
At2g33770(NM\_179887.1
): ubiquitin-conjugating enzyme family protein

Location in genome: in an IGR, 25072 upstream of At1g62960, 2310 downstream of At1g63010

3 homologs in rice

### Alignment between mature miRNA and predicted targets

```
        Extended mature miRNA:       acctgccaaaggagagttgccctgaa
        Target(rc):NM_179887.1       tttTGCCAAAGAAGATTTGCCCTaaa
```

### Precursor sequence and structure. Mature sequence in capital letters

```
tagttttagggcgcctctccattggcaggtcctttacttccaaatatacacatacatatatgaatatcgaaaatttccgatgatcgatttataaatgaccTGCCAAAGGAGAGTTGCCCTgaaactg   
 (((((((((((((.((((((.((((((((((.((((.......(((((........)))))((.(((((........))))).))......)))).)))))))))).)))))).)))))))))))))
```

---

## locus\_id: 70989

family\_id: 77

### **Targets:**

At2g33770(NM\_179887.1
): ubiquitin-conjugating enzyme family protein  
At2g33770(NM\_179887.1
): ubiquitin-conjugating enzyme family protein  
At2g33770(NM\_179887.1
): ubiquitin-conjugating enzyme family protein  
At2g33770(NM\_179887.1
): ubiquitin-conjugating enzyme family protein  
At2g33770(NM\_179887.1
): ubiquitin-conjugating enzyme family protein

Location in genome: in an IGR, 3135 upstream of At1g29270, 960 downstream of At1g29260

5 homologs in brassica

2 homologs in rice

### Alignment between mature miRNA and predicted targets

```
        Extended mature miRNA:       atctgccaaaggagatttgccctgta
        Target(rc):NM_179887.1       aatTGCCAAAGGAGATATGCCCTaga
```

### Precursor sequence and structure. Mature sequence in capital letters

```
gcattacagggtaagatctctattggcaggaaaccattacttagatctttgcatctctttatgcattgcttttaattagtgagttatcTGCCAAAGGAGATTTGCCCTgtaattc   
 ..(((((((((((.(((((((.((((((((.((((((((.(((((.(..(((((......)))))..)..))))).))))).))).)))))))).))))))))))))))))))..
```

---

## locus\_id: 168728

family\_id: 77

### **Targets:**

At2g33770(NM\_179887.1
): ubiquitin-conjugating enzyme family protein  
At2g33770(NM\_179887.1
): ubiquitin-conjugating enzyme family protein

Location in genome: in an IGR, 2339 upstream of At2g34210, 1575 downstream of At2g34200

2 homologs in brassica

1 homologs in rice

### Alignment between mature miRNA and predicted targets

```
        Extended mature miRNA:       tcctctgccaaaggagatttgcctcg
        Target(rc):NM_179887.1       actTTTGCCAAAGAAGATTTGCCcta
```

### Precursor sequence and structure. Mature sequence in capital letters

```
gcattacagggcgaatcctctattggcagtggaagttgatgacccttatatgttattttctcatcattttccTCTGCCAAAGGAGATTTGCCtcgcaatgc   
 (((((.(..((((((((.(((.(((((((.(((((.((((((....((.....)).....)))))).))))).))))))).)))))))))))..).)))))
```

---

## locus\_id: 168735

family\_id: 77

### **Targets:**

At2g33770(NM\_179887.1
): ubiquitin-conjugating enzyme family protein  
At2g33770(NM\_179887.1
): ubiquitin-conjugating enzyme family protein  
At2g33770(NM\_179887.1
): ubiquitin-conjugating enzyme family protein  
At2g33770(NM\_179887.1
): ubiquitin-conjugating enzyme family protein  
At2g33770(NM\_179887.1
): ubiquitin-conjugating enzyme family protein

Location in genome: in an IGR, 835 upstream of At2g34210, 3079 downstream of At2g34200

2 homologs in brassica

2 homologs in rice

### Alignment between mature miRNA and predicted targets

```
        Extended mature miRNA:       ctctgccaaaggagatttgcccggta
        Target(rc):NM_179887.1       taaTGCCAAAGGAGATTTGCTCGaac
```

### Precursor sequence and structure. Mature sequence in capital letters

```
tacagggcaagatcaccattggcagagatctattacttcattcttgcatcatatgcataaatgtttgtggtgagctctcTGCCAAAGGAGATTTGCCCGgta   
 (((.(((((.((((.((.((((((((((.(((((((..((((..(((((...)))))..))))...))))).)).)))))))))).)).))))))))).)))
```

---

## locus\_id: 419659

family\_id: 77

### **Targets:**

At2g33770(NM\_179887.1
): ubiquitin-conjugating enzyme family protein  
At2g33770(NM\_179887.1
): ubiquitin-conjugating enzyme family protein  
At2g33770(NM\_179887.1
): ubiquitin-conjugating enzyme family protein

Location in genome: in an IGR, 2113 upstream of At5g62165, 1305 downstream of At5g62160

1 homologs in brassica

### Alignment between mature miRNA and predicted targets

```
        Extended mature miRNA:       acttgccaaaggagagttgccctgtc
        Target(rc):NM_179887.1       aaaTGCCAAAGGAGATTTGCCCAacg
```

### Precursor sequence and structure. Mature sequence in capital letters

```
agcagtaatagggcatctttctattggcaggcgacttggctatttgtatcttttgtgttcttgactattggctatgtcactTGCCAAAGGAGAGTTGCCCTgtcactgct   
 ((((((.((((((((.((((((.((((((((.(((.((((((...((................))...)))))).))).)))))))).)))))).)))))))).))))))
```

---

## locus\_id: 116413

family\_id: 73

### **Targets:**

At5g22690(NM\_122175.2
): disease resistance protein (TIR-NBS-LRR class), putative

Location in genome: in an IGR, 1960 upstream of At2g12405, 56050 downstream of At2g12480

### Alignment between mature miRNA and predicted targets

```
        Extended mature miRNA:       gccagctcgggatcgatcgattccag
        Target(rc):NM_122175.2       ttaAGCTCGGGATCGAGCGATTGact
```

### Precursor sequence and structure. Mature sequence in capital letters

```
ccagctcgggatcgatcgatcctaggtggggatcgatcgatccaacactcattcgtcgaagaaaatcgtccgccAGCTCGGGATCGATCGATTCcaggtctgg   
 (((((..((((((((((((((((((.((((((.((((((((.............)))))......)))))).))).)).))))))))))))))))..).))))
```

---

## locus\_id: 398557

family\_id: 73

### **Targets:**

At5g07920(NM\_120874.2
): diacylglycerol kinase 1 (DGK1)

Location in genome: in an IGR, 54047 upstream of At5g33280, 18080 downstream of At5g33230

### Alignment between mature miRNA and predicted targets

```
        Extended mature miRNA:       cagctcgggatcgatcgttcccgttc
        Target(rc):NM_120874.2       ataCTCGGGATAGATCATTCCCGgtt
```

### Precursor sequence and structure. Mature sequence in capital letters

```
tccgccagCTCGGGATCGATCGTTCCCGttctagcagacgattttacttcgtggatgagttttggatcgatcgatcccgaactgggga   
 (((.((((.(((((((((((((.(((.(..((..(..((((.......)))).)...))..).))).))))))))))))).)))))))
```

---

## locus\_id: 25094

family\_id: 69

### **Targets:**

At1g79670(NM\_179577.1
): wall-associated kinase, putative  
At1g79670(NM\_106616.3
): wall-associated kinase, putative

Location in genome: in an IGR, 26512 upstream of At1g36763, 62866 downstream of At1g36950

### Alignment between mature miRNA and predicted targets

```
        Extended mature miRNA:       aagctaagtgggaagctggagcagctc
        Target(rc):NM_106616.3       aatCAAAGAGGGAAGCTGGAGCAGtca
        Target(rc):NM_179577.1       aatCAAAGAGGGAAGCTGGAGCAGtca
```

### Precursor sequence and structure. Mature sequence in capital letters

```
ctcggaaagCTAAGTGGGAAGCTGGAGCAGctcaagggaagctcggggtagctcggacagctcaaagggaagctggaccagctcgggagactgctagctagctccactagctcgacttagctggaaggg   
 (((....(((((((((((.((.((((((((((...((....((((....((((.(..(((((........)))))..).))))))))...))...)))).))))))))..))).))))))))....)))
```

---

## locus\_id: 287438

family\_id: 68

### **Targets:**

At3g09100(NM\_111745.2
): mRNA capping enzyme family protein  
At3g09100(NM\_202534.1
): mRNA capping enzyme family protein

Location in genome: Contained by At4g23410 (NM\_118470: . senescence-associated family protein) in an intron on the reverse strand

### Alignment between mature miRNA and predicted targets

```
        Extended mature miRNA:       cgacgtcggtggtttgcagcatccag
        Target(rc):NM_202534.1       atcCATCGGTGGTTAGCAGCATCata
        Target(rc):NM_111745.2       atcCATCGGTGGTTAGCAGCATCata
```

### Precursor sequence and structure. Mature sequence in capital letters

```
ggtcttgttgtatcaccgtgtccgtgttgtaaacgcacgaCGTCGGTGGTTTGCAGCATCcagact   
 ((((((((((((((((((((((.((((.......)))))))).)))))))..))))))...)))))
```

---

## locus\_id: 263961

family\_id: 64

### **Targets:**

At1g52150(NM\_104096.2
): homeobox-leucine zipper family protein / lipid-binding START domain-containing protein  
At1g52150(NM\_179464.1
): homeobox-leucine zipper family protein / lipid-binding START domain-containing protein

Location in genome: in an IGR, 3492 upstream of At3g61900, 18232 downstream of At3g61870

2 homologs in brassica

3 homologs in rice

### Alignment between mature miRNA and predicted targets

```
        Extended mature miRNA:       cgtcggaccaggcttcattcccccca
        Target(rc):NM_104096.2       atcCGGACCAGGCTTCATTCCAGgca
        Target(rc):NM_179464.1       atcCGGACCAGGCTTCATTCCAGgca
```

### Precursor sequence and structure. Mature sequence in capital letters

```
ttgaggggactgttgtctggctcgaggactcttattctaatacaatctcatttgaatacattcagatctgatgattgattagggttttagtgtcgtCGGACCAGGCTTCATTCCCCccaa   
 .((.(((((.((..((((((..(((.(((.((.((((((((.((((((((((((((....))))))..))).)))))))))))))...)).))).)))..))))))..)).))))).)).
```

---

## locus\_id: 407523

family\_id: 64

### **Targets:**

At1g52150(NM\_104096.2
): homeobox-leucine zipper family protein / lipid-binding START domain-containing protein

Location in genome: in an IGR, 2681 upstream of At5g43610, 884 downstream of At5g43600

2 homologs in brassica

3 homologs in rice

### Alignment between mature miRNA and predicted targets

```
        Extended mature miRNA:       tgtcggaccaggcttcattcccctca
        Target(rc):NM_104096.2       atcCGGACCAGGCTTCATTCCAGgca
```

### Precursor sequence and structure. Mature sequence in capital letters

```
aagttcaggtgaatgatgcctggctcgagaccattcaatctcatgatctcatgattataacgatgatgatgatgatgtCGGACCAGGCTTCATTCCCCtcaactt   
 (((((.(((.((((((.((((((..(((.(.((((..(((((((((((....))))))...)).)))...)))).).)))..)))))).)))))).))).)))))
```

---

## locus\_id: 420573

family\_id: 64

### **Targets:**

At1g52150(NM\_104096.2
): homeobox-leucine zipper family protein / lipid-binding START domain-containing protein

Location in genome: in an IGR, 1447 upstream of At5g63720, 2085 downstream of At5g63710

3 homologs in brassica

3 homologs in rice

### Alignment between mature miRNA and predicted targets

```
        Extended mature miRNA:       tctcggaccaggcttcattcccctca
        Target(rc):NM_104096.2       atcCGGACCAGGCTTCATTCCAGgca
```

### Precursor sequence and structure. Mature sequence in capital letters

```
gggtttagaggaatgttgtttggctcgaggtcatggagagtaattcgttaacccaactcaaaactctaaatgattctCGGACCAGGCTTCATTCCCCtcaacct   
 (((((.((.((((((..((((((..(((((((((..(((((.....(((.....))).....)))))..)))).)))))..))))))..)))))).)).)))))
```

---

## locus\_id: 285344

family\_id: 63

### **Targets:**

At1g48520(NM\_103748.2
): glutamyl-tRNA(Gln) amidotransferase B family protein  
At1g48520(NM\_202262.1
): glutamyl-tRNA(Gln) amidotransferase B family protein  
At1g48520(NM\_202261.1
): glutamyl-tRNA(Gln) amidotransferase B family protein

Location in genome: in an IGR, 7932 upstream of At4g20930, 17262 downstream of At4g21010

### Alignment between mature miRNA and predicted targets

```
        Extended mature miRNA:       tgacggaatctagtttcgacaacttt
        Target(rc):NM_202262.1       accCGGAATCTAGTTTCGACAATgca
        Target(rc):NM_202261.1       accCGGAATCTAGTTTCGACAATgca
        Target(rc):NM_103748.2       accCGGAATCTAGTTTCGACAATgca
```

### Precursor sequence and structure. Mature sequence in capital letters

```
cgttttgaCGGAATCTAGTTTCGACAACtttggtttcgtcggacgagagattaagccgagctccacgctcgcaccgttacgacggaatcaatagtttcgacaactttggtttcgtcggacg   
 (((((.(((((((((.((((((((.((((((((((((((((..(....).....((.((((.....)))))).......)))))))))))).)))))))).))))..))))))))))))))
```

---

## locus\_id: 201719

family\_id: 61

### **Targets:**

At3g14120(NM\_112268.2
): expressed protein  
At3g14120(NM\_180250.1
): expressed protein

Location in genome: in an IGR, 3472 upstream of At3g31330, 79209 downstream of At3g31410

### Alignment between mature miRNA and predicted targets

```
        Extended mature miRNA:       aagcgatggcctggagagtgctctagt
        Target(rc):NM_112268.2       gctCGATGGCCTGGAGAGTCCTATttt
        Target(rc):NM_180250.1       gctCGATGGCCTGGAGAGTCCTATttt
```

### Precursor sequence and structure. Mature sequence in capital letters

```
cgctagagctcttctctcccggctgtctcctgctcctgcctaagCGATGGCCTGGAGAGTGCTCTagtg   
 (((((((((....((((((.(((((((.(..((....))....).))))))).)))))).)))))))))
```

---

## locus\_id: 116412

family\_id: 60

### **Targets:**

At2g40980(NM\_129663.3
): expressed protein  
At2g40980(NM\_129663.3
): expressed protein

Location in genome: in an IGR, 1950 upstream of At2g12405, 56060 downstream of At2g12480

### Alignment between mature miRNA and predicted targets

```
        Extended mature miRNA:       gatcgatcgattccaggtctggatcg
        Target(rc):NM_129663.3       accCTATCGATTCCAGGTCTGGAgat
```

### Precursor sequence and structure. Mature sequence in capital letters

```
agaaatcgtctgccagctcgggatcgatcgatcctaggtggggatcgatcgatccaacactcattcgtcgaagaaaatcgtccgccagctcgggatCGATCGATTCCAGGTCTGGAtcggtcaat   
 ....((((....(((((..((((((((((((((((((.((((((.((((((((.............)))))......)))))).))).)).))))))))))))))))..).))))..))))....
```

---

## locus\_id: 25771

family\_id: 59

### **Targets:**

At3g31350(NM\_113998.2
): hypothetical protein

Location in genome: in an IGR, 53277 upstream of At1g38131, 161394 downstream of At1g38630

### Alignment between mature miRNA and predicted targets

```
        Extended mature miRNA:       gatcgatcgatccccacctgggatcggtc
        Target(rc):NM_113998.2       gagCGACCGATCCCCACATGGGATCGatc
```

### Precursor sequence and structure. Mature sequence in capital letters

```
agggttggatCGATCGATCCCCACCTGGGATCGgtcgatccctcgctggctgatagatttcttcaacagatgattggtggatcgatcgatccccacctaggagcgatcgatcccgttct   
 ((..(.((((((((((.(((......((((((((((((((((((((((..(((.((....))))).))).)))...).)))))))))))))))......))).)))))))))).)..))
```

---

## locus\_id: 25855

family\_id: 59

### **Targets:**

At3g31350(NM\_113998.2
): hypothetical protein

Location in genome: in an IGR, 98930 upstream of At1g38131, 115741 downstream of At1g38630

### Alignment between mature miRNA and predicted targets

```
        Extended mature miRNA:       gatcgatcgatccccacctgggatcggtc
        Target(rc):NM_113998.2       gagCGACCGATCCCCACATGGGATCGatc
```

### Precursor sequence and structure. Mature sequence in capital letters

```
agggttggatCGATCGATCCCCACCTGGGATCGgtcgatccctcgctggctgatagatttcttcaacagatgattggtggatcgatcgatccccacctaggagcgatcgatcccgttct   
 ((..(.((((((((((.(((......((((((((((((((((((((((..(((.((....))))).))).)))...).)))))))))))))))......))).)))))))))).)..))
```

---

## locus\_id: 310681

family\_id: 58

### **Targets:**

At1g68400(NM\_105511.2
): leucine-rich repeat transmembrane protein kinase, putative

Location in genome: Contained by At4g11480 (NM\_117219: . protein kinase family protein) in an intron

### Alignment between mature miRNA and predicted targets

```
        Extended mature miRNA:       cgatagccgcttcaccgcaactttgg
        Target(rc):NM_105511.2       tttTAACCGCTTCACCGCAACCTcat
```

### Precursor sequence and structure. Mature sequence in capital letters

```
gaagcttcgaTAGCCGCTTCACCGCAACTTtggtttcattcgtcagtaattgtaatattgtatatttgtagggaatgttaccaaacgaaacagaagttgcggtgaagcggctatcgagtaattc   
 ...(((.((((((((((((((((((((((((.(((((......(((((.......)))))..........((........))....))))).))))))))))))))))))))))))))).....
```

---

## locus\_id: 204872

family\_id: 56

### **Targets:**

At3g43160(NM\_114184.1
): expressed protein

Location in genome: in an IGR, 96563 upstream of At3g33520, 36363 downstream of At3g42050

### Alignment between mature miRNA and predicted targets

```
        Extended mature miRNA:       cgtcctggtcgacgaatcctcggacccg
        Target(rc):NM_114184.1       accCCTGGTCGATGAATCCTCGAACacg
```

### Precursor sequence and structure. Mature sequence in capital letters

```
tcgtCCTGGTCGACGAATCCTCGGACccggtcgatgaagtctcggacctggtcgatgatttcaacccctggtcgacaaatcctcggacccggtcgacgattcctcgtcctggtcga   
 (((.((.((.(((.(((((.(((.(((.((((((.((.(((...((((.(((.((.....)).)))...)))))))...)).)).)))).)))))).))))).))).)).)).)))
```

---

## locus\_id: 204949

family\_id: 56

### **Targets:**

At3g43160(NM\_114184.1
): expressed protein

Location in genome: in an IGR, 99228 upstream of At3g33520, 33698 downstream of At3g42050

### Alignment between mature miRNA and predicted targets

```
        Extended mature miRNA:       cgtcctggtcgacgaatcctcggacccg
        Target(rc):NM_114184.1       accCCTGGTCGATGAATCCTCGAACacg
```

### Precursor sequence and structure. Mature sequence in capital letters

```
tcgtCCTGGTCGACGAATCCTCGGACccggtcgatgaagtctcggacctggtcgacgatttcaacccctggtcgacaaatcctcggacccggtcgacgattcctcgtcctggtcga   
 (((.((.((.(((.(((((.(((.(((.((((((.((.(((...((((.(((.((.....)).)))...)))))))...)).)).)))).)))))).))))).))).)).)).)))
```

---

## locus\_id: 142662

family\_id: 55

### **Targets:**

At2g28780(NM\_128437.2
): expressed protein

Location in genome: in an IGR, 168 upstream of At2g01020, 82 downstream of At2g01010

### Alignment between mature miRNA and predicted targets

```
        Extended mature miRNA:       tgcccgccggatccgtggtttcgcgt
        Target(rc):NM_128437.2       ccgCCGCCAGAGCCGTGGTTTCGgct
```

### Precursor sequence and structure. Mature sequence in capital letters

```
cgacccgcgaaccaaagatcaccactctcggtgggccggtttcttagccgattccttgcCCGCCGGATCCGTGGTTTCGcgtatcg   
 (((..(((((((((..((((........((((((((.((..((......))..))..))))))))))))..))).))))))..)))
```

---

## locus\_id: 248418

family\_id: 55

### **Targets:**

At2g28780(NM\_128437.2
): expressed protein

Location in genome: in an IGR, 168 upstream of At3g41979, 82 downstream of At3g41768

### Alignment between mature miRNA and predicted targets

```
        Extended mature miRNA:       tgcccgccggatccgtggtttcgcgt
        Target(rc):NM_128437.2       ccgCCGCCAGAGCCGTGGTTTCGgct
```

### Precursor sequence and structure. Mature sequence in capital letters

```
cgacccgcgaaccaaagatcaccactctcggtgggccggtttcttagccgattccttgcCCGCCGGATCCGTGGTTTCGcgtatcg   
 (((..(((((((((..((((........((((((((.((..((......))..))..))))))))))))..))).))))))..)))
```

---

## locus\_id: 232477

family\_id: 54

### **Targets:**

At4g00610(NM\_116286.1
): DNA-binding storekeeper protein-related

Location in genome: in an IGR, 19448 upstream of At3g17350, 5734 downstream of At3g17300

### Alignment between mature miRNA and predicted targets

```
        Extended mature miRNA:       tgcgacatccgacgatttcttcgccgt
        Target(rc):NM_116286.1       tgtGACACCCGACGGTTTCTTCGCtgg
```

### Precursor sequence and structure. Mature sequence in capital letters

```
tcgacggcgaagaaatcaccggagatgcgactcccaacggtttctttgaagtggatgcGACATCCGACGATTTCTTCGCcgttga   
 (((((((((((((((((..((((..((((..((((..(((.....)))..).)))))))...))))..)))))))))))))))))
```

---

## locus\_id: 172817

family\_id: 53

### **Targets:**

At1g67890(NM\_105459.2
): protein kinase family protein

Location in genome: Contained by At2g40440 (NM\_129605: . BTB/POZ domain-containing protein) in an intron on the reverse strand

3 homologs in brassica

### Alignment between mature miRNA and predicted targets

```
        Extended mature miRNA:       aatccccacggctgcaaacaccttaa
        Target(rc):NM_105459.2       gaaCCCCACGGCTCCAATCACCTgca
```

### Precursor sequence and structure. Mature sequence in capital letters

```
ccacttataggtgggccagtggtaggaaggatttcgggctgagggtgaaattcccaataatcccgggttcgaatCCCCACGGCTGCAAACACCTtaagtgg   
 (((((((.(((((.((.(((.((.((..(((((.((((((..((((.............))))..))))))))))))))).)))))...))))))))))))
```

---

## locus\_id: 416389

family\_id: 53

### **Targets:**

At1g67890(NM\_105459.2
): protein kinase family protein

Location in genome: in an IGR, 23906 upstream of At5g56460, 12981 downstream of At5g56365

3 homologs in brassica

### Alignment between mature miRNA and predicted targets

```
        Extended mature miRNA:       aatccccacggctgcaaacaccttaa
        Target(rc):NM_105459.2       gaaCCCCACGGCTCCAATCACCTgca
```

### Precursor sequence and structure. Mature sequence in capital letters

```
ccactcataggtgggccagtggtaggaaggatttcgggctgagggtgaaattcccaataatcccgggttcgaatCCCCACGGCTGCAAACACCTtaagtgg   
 (((((...(((((.((.(((.((.((..(((((.((((((..((((.............))))..))))))))))))))).)))))...)))))..)))))
```

---

## locus\_id: 188393

family\_id: 51

### **Targets:**

At2g24150(NM\_127976.2
): expressed protein

Location in genome: in an IGR, 1330 upstream of At3g17310, 833 downstream of At3g17320

### Alignment between mature miRNA and predicted targets

```
        Extended mature miRNA:       tcgcatctccggtgatttcttcgccgtcg
        Target(rc):NM_127976.2       cacCATCGCCGGAGATTTCTTCGCCGatc
```

### Precursor sequence and structure. Mature sequence in capital letters

```
tttctcaacggcgaagaaatcgtcggatgtcgcatccacttcaaagaaaccgttgggagtcgCATCTCCGGTGATTTCTTCGCCGtcgatggag   
 .(((((.((((((((((((((..(((((((.((..(((...............)))..)).)))..))))..)))))))))))))).)).))).
```

---

## locus\_id: 188394

family\_id: 51

### **Targets:**

At2g32820(NM\_128842.1
): hypothetical protein

Location in genome: in an IGR, 1334 upstream of At3g17310, 832 downstream of At3g17320

### Alignment between mature miRNA and predicted targets

```
        Extended mature miRNA:       gtcgcatctccggtgatttcttcgcc
        Target(rc):NM_128842.1       ccaACATCTCCGGTGAGTTCTTCttc
```

### Precursor sequence and structure. Mature sequence in capital letters

```
tcaacggcgaagaaatcgtcggatgtcgcatccacttcaaagaaaccgttgggagtcGCATCTCCGGTGATTTCTTCgccgtcga   
 ((.((((((((((((((..(((((((.((..(((...............)))..)).)))..))))..)))))))))))))).))
```

---

## locus\_id: 107902

family\_id: 49

### **Targets:**

At1g68845(NM\_148641.1
): hypothetical protein

Location in genome: in an IGR, 51763 upstream of At2g02570, 6002 downstream of At2g02710

### Alignment between mature miRNA and predicted targets

```
        Extended mature miRNA:       tgcagcacatcaaccctaacctctttt
        Target(rc):NM_148641.1       taaACAACATCAACCCTAACCTCTacc
```

### Precursor sequence and structure. Mature sequence in capital letters

```
ggcgctgcAGCACATCAACCCTAACCTCTtttcagtgggcccttctagcccaccaggtaggttattggtgcgcttggcgtt   
 .((((((.(((.((((((...(((((((((....((((((.......)))))).))).)))))))))))).))))))))).
```

---

## locus\_id: 401398

family\_id: 49

### **Targets:**

At1g32180(NM\_102951.1
): cellulose synthase family protein

Location in genome: Contained by At5g35850 (NM\_122976: . ) in an intron on the reverse strand

### Alignment between mature miRNA and predicted targets

```
        Extended mature miRNA:       gctgcagcgcatcaaccctaacctctc
        Target(rc):NM_102951.1       gagGCAGCGCATTAACCCGAACCTtaa
```

### Precursor sequence and structure. Mature sequence in capital letters

```
gcgctGCAGCGCATCAACCCTAACCTctcttatgtggcccctctagcccacatgtaggttattggtgcgcttcgcgt   
 ((((.(.((((((((((...((((((....(((((((.(......).))))))).)))))))))))))))).)))))
```

---

## locus\_id: 92544

family\_id: 46

### **Targets:**

At2g37340(NM\_201890.1
): splicing factor RSZ33 (RSZ33)  
At2g37340(NM\_201891.1
): splicing factor RSZ33 (RSZ33)  
At2g37340(NM\_179949.2
): splicing factor RSZ33 (RSZ33)

Location in genome: in an IGR, 4303 upstream of At1g61240, 1662 downstream of At1g61215

### Alignment between mature miRNA and predicted targets

```
        Extended mature miRNA:       aaacaccatcatggtcagatccgtca
        Target(rc):NM_179949.2       tacCACCGTCACGGTCAGATCCGact
        Target(rc):NM_201890.1       tacCACCGTCACGGTCAGATCCGact
        Target(rc):NM_201891.1       tacCACCGTCACGGTCAGATCCGact
```

### Precursor sequence and structure. Mature sequence in capital letters

```
gcgggatgatgaatccgaccatgatggtgttgcagaaatttatgtatgatgtagttattgtccagggatcgaaaCACCATCATGGTCAGATCCGtcatcccac   
 ..(((((((((.(((.(((((((((((((((...((..((..((.(..(((....)))..).))..))))..))))))))))))))).))).)))))))))..
```

---

## locus\_id: 222701

family\_id: 45

### **Targets:**

At5g58360(NM\_125221.1
): ovate family protein

Location in genome: in an IGR, 3144 upstream of At3g02680, 5062 downstream of At3g02650

### Alignment between mature miRNA and predicted targets

```
        Extended mature miRNA:       taagcacatcatcttgcttcctccac
        Target(rc):NM_125221.1       cagGCACATCATCAAGCTTCCTCaca
```

### Precursor sequence and structure. Mature sequence in capital letters

```
ttgtggagaggaagcaagaggatgtgcttggttgtggaaatatagggcccttaaaatatattcatcgtattcactcacataacaaaaattccacaagtaaGCACATCATCTTGCTTCCTCcacaa   
 .((((((..((((((((((.((((((((((.((((((((....((....))...(((((.......))))).................)))))))).)))))))))).)))))))))))))))).
```

---

## locus\_id: 20922

family\_id: 44

### **Targets:**

At1g02850(NM\_202017.1
): glycosyl hydrolase family 1 protein  
At1g02850(NM\_202016.1
): glycosyl hydrolase family 1 protein  
At1g02850(NM\_100166.2
): glycosyl hydrolase family 1 protein  
At1g02850(NM\_179247.2
): glycosyl hydrolase family 1 protein

Location in genome: in an IGR, 17049 upstream of At1g32690, 7469 downstream of At1g32750

### Alignment between mature miRNA and predicted targets

```
        Extended mature miRNA:       cttcaatctagacctaacattgggta
        Target(rc):NM_202016.1       tggCAATCTAGACCCAACATTGGtct
        Target(rc):NM_179247.2       tggCAATCTAGACCCAACATTGGtct
        Target(rc):NM_100166.2       tggCAATCTAGACCCAACATTGGtct
        Target(rc):NM_202017.1       tggCAATCTAGACCCAACATTGGtct
```

### Precursor sequence and structure. Mature sequence in capital letters

```
tttgtagcttCAATCTAGACCTAACATTGGgtattaaagcttctatatatatagaagctctgataccacatgttagttttagatcaaagtttcaaa   
 .(((.(((((..(((((((.(((((((..(((((((.(((((((((....))))))))).)))))))..))))))).)))))))..))))).))).
```

---

## locus\_id: 364755

family\_id: 43

### **Targets:**

At3g21170(NM\_113011.1
): F-box family protein

Location in genome: in an IGR, 1261 upstream of At5g48270, 68 downstream of At5g48280

### Alignment between mature miRNA and predicted targets

```
        Extended mature miRNA:       caacaataagtcatcgtagtgaaagactaa
        Target(rc):NM_113011.1       tagCAATAAGCCATCGCAGTGAAAGACgtg
```

### Precursor sequence and structure. Mature sequence in capital letters

```
cattttgttgtctgtaactgcgatggcttagtgttgtgcgttaccaaagacaacacaagccttatgttttctttggtaacacaaCAATAAGTCATCGTAGTGAAAGACtaacggtg   
 ....(((((((((...((((((((((((((.((((((..(((((((((((.((((.........)))).))))))))))))))))).))))))))))))))...)))).)))))..
```

---

## locus\_id: 364756

family\_id: 43

### **Targets:**

At3g49520(NM\_114812.1
): F-box family protein

Location in genome: in an IGR, 1266 upstream of At5g48270, 66 downstream of At5g48280

### Alignment between mature miRNA and predicted targets

```
        Extended mature miRNA:       cacaacaataagtcatcgtagtgaaag
        Target(rc):NM_114812.1       catAACAATAAGCCATCGCAGTGAtag
```

### Precursor sequence and structure. Mature sequence in capital letters

```
gttgtctgtaactgcgatggcttagtgttgtgcgttaccaaagacaacacaagccttatgttttctttggtaacacAACAATAAGTCATCGTAGTGAaagactaac   
 (((((((...((((((((((((((.((((((..(((((((((((.((((.........)))).))))))))))))))))).))))))))))))))...)))).)))
```

---

## locus\_id: 371732

family\_id: 40

### **Targets:**

At5g58830(NM\_125273.2
): subtilase family protein

Location in genome: in an IGR, 1990 upstream of At5g58800, 16533 downstream of At5g58860

### Alignment between mature miRNA and predicted targets

```
        Extended mature miRNA:       gttggattcggaccattgttaccagc
        Target(rc):NM_125273.2       gtcGGATTCGGACCAGCGTTACCggc
```

### Precursor sequence and structure. Mature sequence in capital letters

```
gtgctggctgcaacggttaagatccacggtgctacgctcacggttGGATTCGGACCATTGTTACCagctgac   
 ..(((((..((((.((((..(((((((.(((.......))).).))))))..)))).))))..)))))....
```

---

## locus\_id: 204927

family\_id: 39

### **Targets:**

At3g43160(NM\_114184.1
): expressed protein  
At3g43160(NM\_114184.1
): expressed protein

Location in genome: in an IGR, 98469 upstream of At3g33520, 34458 downstream of At3g42050

### Alignment between mature miRNA and predicted targets

```
        Extended mature miRNA:       ttcctcgtcctggtcgacgattcctcg
        Target(rc):NM_114184.1       tctCTCGTCCTGGTTGAGGATTCCgcc
```

### Precursor sequence and structure. Mature sequence in capital letters

```
ctggtcgacgaatcctcggacccggtcgatgaagtctcggacctggtcgacgatttcaacccctggtcgacaaatcctcggacccggtcgacgattcCTCGTCCTGGTCGACGATTCCtcggacccg   
 ..((((((.(((((.(((.(((.((.(((.(((.(((((.(((.((((((.((((((.(((...))).))..)))).)).)))).)))))).))))).))).)).)))))).))))).)).))))..
```

---

## locus\_id: 205064

family\_id: 39

### **Targets:**

At3g43160(NM\_114184.1
): expressed protein  
At3g43160(NM\_114184.1
): expressed protein

Location in genome: in an IGR, 108878 upstream of At3g33520, 24049 downstream of At3g42050

### Alignment between mature miRNA and predicted targets

```
        Extended mature miRNA:       ttcctcgtcctggtcgacgattcctcg
        Target(rc):NM_114184.1       tctCTCGTCCTGGTTGAGGATTCCgcc
```

### Precursor sequence and structure. Mature sequence in capital letters

```
ctggtcgacgaatcctcggacccggtcgatgaagtctcggacctggtcgacgatttcaacccctggtcgacaaatcctcggacccggtcgacgattcCTCGTCCTGGTCGACGATTCCtcggacccg   
 ..((((((.(((((.(((.(((.((.(((.(((.(((((.(((.((((((.((((((.(((...))).))..)))).)).)))).)))))).))))).))).)).)))))).))))).)).))))..
```

---

## locus\_id: 264979

family\_id: 39

### **Targets:**

At3g43160(NM\_114184.1
): expressed protein

Location in genome: in an IGR, 2678 downstream of At4g00010

### Alignment between mature miRNA and predicted targets

```
        Extended mature miRNA:       cgaattctttgtcctggtcgaggattccatc
        Target(rc):NM_114184.1       accATTCTTCGTCCTAGTCGAGGATTCCgcc
```

### Precursor sequence and structure. Mature sequence in capital letters

```
tggtcgacgaATTCTTTGTCCTGGTCGAGGATTCCatcacctggtagacgaatcctcgaacccgatagattttcctcgacca   
 .((((((.(((...((((((..((((((((((((..((........)).))))))))).))).))))))..))).)))))).
```

---

## locus\_id: 21222

family\_id: 38

### **Targets:**

At1g34310(NM\_103153.2
): transcriptional factor B3 family protein / auxin-responsive factor AUX/IAA-related

Location in genome: in an IGR, 56589 upstream of At1g33060, 16336 downstream of At1g33240

### Alignment between mature miRNA and predicted targets

```
        Extended mature miRNA:       gggattaaatgctcgatgtcccatggtgaaac
        Target(rc):NM_103153.2       ggcATTAAATGTTCGATGTCCCAGGGTGAcac
```

### Precursor sequence and structure. Mature sequence in capital letters

```
tgatgaagggATTAAATGCTCGATGTCCCATGGTGAaactttgtcaggtccttcatttttacctgataaggtttcaacatggaatatcgagcatttaatgctttcatca   
 .((((((((.((((((((((((((((.(((((.((((((((((((((((...........)))))))))))))))).))))).)))))))))))))))).)))))))).
```

---

## locus\_id: 21223

family\_id: 38

### **Targets:**

At1g34170(NM\_103140.2
): transcriptional factor B3 family protein

Location in genome: in an IGR, 56595 upstream of At1g33060, 16330 downstream of At1g33240

### Alignment between mature miRNA and predicted targets

```
        Extended mature miRNA:       gatgaagggattaaatgctcgatgtcccatgg
        Target(rc):NM_103140.2       gacGAAGGAATTAGATGCTCGATGTCCCAagg
```

### Precursor sequence and structure. Mature sequence in capital letters

```
gaaatttgatGAAGGGATTAAATGCTCGATGTCCCAtggtgaaactttgtcaggtccttcatttttacctgataaggtttcaacatggaatatcgagcatttaatgctttcatcaattatttc   
 (((((((((((((((.((((((((((((((((.(((((.((((((((((((((((...........)))))))))))))))).))))).)))))))))))))))).))))))))))..)))))
```

---

## locus\_id: 365579

family\_id: 37

### **Targets:**

At1g68020(NM\_105472.2
): glycosyl transferase family 20 protein / trehalose-phosphatase family protein  
At1g68020(NM\_202376.1
): glycosyl transferase family 20 protein / trehalose-phosphatase family protein

Location in genome: in an IGR, 6422 upstream of At5g49300, 100 downstream of At5g49310

### Alignment between mature miRNA and predicted targets

```
        Extended mature miRNA:       acaatgtggattccaacaggaagtgg
        Target(rc):NM_105472.2       cccATGTGGATTCCAACAGGTAGaat
        Target(rc):NM_202376.1       cccATGTGGATTCCAACAGGTAGaat
```

### Precursor sequence and structure. Mature sequence in capital letters

```
aaacaATGTGGATTCCAACAGGAAGtggaacttggttctctaacagctactcatcatattggaatcaagttccacttcctgttagagaaccaagttccacttcctgttggaatccacattgttt   
 ((((((((((((((((((((((((((((((((((((((((((((((.............((((((...))))))....))))))))))))))))))))))))))))))))))))))))))))))
```

---

## locus\_id: 411484

family\_id: 37

### **Targets:**

At1g68020(NM\_202376.1
): glycosyl transferase family 20 protein / trehalose-phosphatase family protein  
At1g68020(NM\_105472.2
): glycosyl transferase family 20 protein / trehalose-phosphatase family protein

Location in genome: in an IGR, 32180 upstream of At5g49370, 7934 downstream of At5g49290

### Alignment between mature miRNA and predicted targets

```
        Extended mature miRNA:       acaatgtggattccaacaggaagtgg
        Target(rc):NM_105472.2       cccATGTGGATTCCAACAGGTAGaat
        Target(rc):NM_202376.1       cccATGTGGATTCCAACAGGTAGaat
```

### Precursor sequence and structure. Mature sequence in capital letters

```
aaacaATGTGGATTCCAACAGGAAGtggaacttggttctctaacaggaagtggaacttgattccaatatgatgagtagctgttagagaaccaagttccacttcctgttggaatccacattgttt   
 ((((((((((((((((((((((((((((((((((((((((((((((....(((((.....))))).(((.....))).))))))))))))))))))))))))))))))))))))))))))))))
```

---

## locus\_id: 12302

family\_id: 35

### **Targets:**

At3g16570(NM\_112530.2
): rapid alkalinization factor (RALF) family protein

Location in genome: in an IGR, 11646 upstream of At1g20370, 6885 downstream of At1g20400

### Alignment between mature miRNA and predicted targets

```
        Extended mature miRNA:       gaaatgggccggttgatcactgaggcc
        Target(rc):NM_112530.2       aatATGCGCCGGTTGATCTCTGAGtcc
```

### Precursor sequence and structure. Mature sequence in capital letters

```
gggcttcggcctgctgggccggctcgagttctaaagtgatggtcgggctgggtcgttattccttgaaATGGGCCGGTTGATCACTGAGgccc   
 (((((((((..((.(.((((((((((..(((...(((((((..(......)..)))))))....))).)))))))))).).)))))))))))
```

---

## locus\_id: 369298

family\_id: 35

### **Targets:**

At3g16570(NM\_112530.2
): rapid alkalinization factor (RALF) family protein

Location in genome: in an IGR, 1114 upstream of At5g54690, 122 downstream of At5g54700

### Alignment between mature miRNA and predicted targets

```
        Extended mature miRNA:       gaaatgggccggttgatcactgaggcc
        Target(rc):NM_112530.2       aatATGCGCCGGTTGATCTCTGAGtcc
```

### Precursor sequence and structure. Mature sequence in capital letters

```
gggcttcggcctggtgggccggctcgagttctaaagtgatggtcggggctgggtcgttattccttgaaATGGGCCGGTTGATCACTGAGgccc   
 (((((((((..((((.((((((((((..(((...(((((((..(.......)..)))))))....))).)))))))))).)))))))))))))
```

---

## locus\_id: 115927

family\_id: 34

### **Targets:**

At1g74330(NM\_106093.1
): protein kinase, putative

Location in genome: in an IGR, 45330 upstream of At2g11775, 1297 downstream of At2g11830

### Alignment between mature miRNA and predicted targets

```
        Extended mature miRNA:       agtctcaccttcttccatgccatggc
        Target(rc):NM_106093.1       aacCTCACCTTCTTCAATGCCACaat
```

### Precursor sequence and structure. Mature sequence in capital letters

```
gtgattatcatggctaggaaaaaggtacgacttgtagtgataatcatggaggttttgattcacgattcaggcaagtCTCACCTTCTTCCATGCCATggccatcac   
 (((((..(((((((..((((.(((((..(((((((..(((..(((.((((........)))).)))))).)))))))..))))).))))..)))))))..)))))
```

---

## locus\_id: 310417

family\_id: 34

### **Targets:**

At2g26880(NM\_128245.2
): MADS-box family protein

Location in genome: in an IGR, 8544 upstream of At4g11150, 6956 downstream of At4g11130

### Alignment between mature miRNA and predicted targets

```
        Extended mature miRNA:       acgattcatgcaagtctcaccttcttcca
        Target(rc):NM_128245.2       tctATCCATGCAAGTTTCACCTTCTTtct
```

### Precursor sequence and structure. Mature sequence in capital letters

```
atggctaggaaaaaggtacgacttgcagtgataatcatggaggttttgattcacgATTCATGCAAGTCTCACCTTCTTccatgccat   
 (((((..((((.(((((..((((((((.(((..(((.((((........)))).))))))))))))))..))))).))))..)))))
```

---

## locus\_id: 310419

family\_id: 34

### **Targets:**

At1g74330(NM\_106093.1
): protein kinase, putative

Location in genome: in an IGR, 8536 upstream of At4g11150, 6967 downstream of At4g11130

### Alignment between mature miRNA and predicted targets

```
        Extended mature miRNA:       aagtctcaccttcttccatgccatgg
        Target(rc):NM_106093.1       aaaCCTCACCTTCTTCAATGCCAcaa
```

### Precursor sequence and structure. Mature sequence in capital letters

```
tgattatcatggctaggaaaaaggtacgacttgcagtgataatcatggaggttttgattcacgattcatgcaagTCTCACCTTCTTCCATGCCAtggccatca   
 .(((..(((((((..((((.(((((..((((((((.(((..(((.((((........)))).))))))))))))))..))))).))))..)))))))..))).
```

---

## locus\_id: 30784

family\_id: 33

### **Targets:**

At1g64580(NM\_105133.1
): pentatricopeptide (PPR) repeat-containing protein

Location in genome: in an IGR, 8583 upstream of At1g48260, 2124 downstream of At1g48270

### Alignment between mature miRNA and predicted targets

```
        Extended mature miRNA:       attgattaatgcagtgaaagtgactaa
        Target(rc):NM_105133.1       atcGATCAACGCAGTGAAAGTGACaac
```

### Precursor sequence and structure. Mature sequence in capital letters

```
tgcattGATTAATGCAGTGAAAGTGACtaaaccagggttgattactgtttccgcttcatcatatgaagaacaaaaaaaatcggaaccccgatgtagtcactttcaatgcattgatcgacgca   
 .((.((((((((((((.((((((((((((...(.(((((((((....(((...((((((...))))))...)))...))))..))))).)...)))))))))))).)))))))))))).)).
```

---

## locus\_id: 65312

family\_id: 32

### **Targets:**

At5g13780(NM\_121381.2
): GCN5-related N-acetyltransferase, putative

Location in genome: in an IGR, 3356 upstream of At1g20380, 13694 downstream of At1g20350

1 homologs in brassica

### Alignment between mature miRNA and predicted targets

```
        Extended mature miRNA:       gggtatactgccaatagagatctgttaggg
        Target(rc):NM_121381.2       tacTATATTGCCAATAGAGATCTGCTAtct
```

### Precursor sequence and structure. Mature sequence in capital letters

```
aagattttcttacagtcatctttggcattctgtccacctccttctatacatatatgcatgtgtatatatatatgcgtttcgtgtgaaagaaggaggtgggTATACTGCCAATAGAGATCTGTTAgggcttctt   
 ((((..((((.((((((.(((((((((.(.(..((((((((((((.(((((..((((((((((....))))))))))...)))))..))))))))))))..).).)))))).))))).)))).))))..))))
```

---

## locus\_id: 103294

family\_id: 32

### **Targets:**

At5g13780(NM\_121381.2
): GCN5-related N-acetyltransferase, putative

Location in genome: in an IGR, 2373 upstream of At1g76140, 4829 downstream of At1g76130

1 homologs in brassica

### Alignment between mature miRNA and predicted targets

```
        Extended mature miRNA:       ggcatactgccaatagagatctgttaggg
        Target(rc):NM_121381.2       actATATTGCCAATAGAGATCTGCTAtct
```

### Precursor sequence and structure. Mature sequence in capital letters

```
ctcttacagagatctttggcattctgtccacctcctctctctatatttatgtgtaataagtgtacgtatctacggtgtgtttcgtaagaggaggtgggcATACTGCCAATAGAGATCTGTTAggg   
 ((((.(((((..(((((((((.(.(((((((((((((....(((((((((.....)))))))))......(((((......))))).))))))))))))).).)))))).)))..))))).))))
```

---

## locus\_id: 272830

family\_id: 30

### **Targets:**

At3g44370(NM\_114305.1
): expressed protein

Location in genome: in an IGR, 84 upstream of At4g07380, 4728 downstream of At4g07400

### Alignment between mature miRNA and predicted targets

```
        Extended mature miRNA:       gagaggttagggttgatgcgctgcag
        Target(rc):NM_114305.1       ggtAGGTTAGGGTTGATGCGCTGgat
```

### Precursor sequence and structure. Mature sequence in capital letters

```
gagAGGTTAGGGTTGATGCGCTGcagcaccgactgggtcccactggacgggttggtactgtgattttggtgagacgagtcatagcctctc   
 (((((((((((.(((...((((((((.(((((((.(.(((....)))).))))))).))))......))))...))).)).)))))))))
```

---

## locus\_id: 345384

family\_id: 30

### **Targets:**

At3g44370(NM\_114305.1
): expressed protein

Location in genome: in an IGR, 425 upstream of At5g24155, 1641 downstream of At5g24160

2 homologs in brassica

### Alignment between mature miRNA and predicted targets

```
        Extended mature miRNA:       aggggttagggtcgatgcgctgcagag
        Target(rc):NM_114305.1       gtaGGTTAGGGTTGATGCGCTGGAtgg
```

### Precursor sequence and structure. Mature sequence in capital letters

```
ggtgaggGGTTAGGGTCGATGCGCTGCAgagctaacaggatgccacgtggcagagagtggccacgtgtgcgggtctcacgaggcgggttgttacactaacttctggacc   
 (((.((((((((((..((((.((((...(((...((....(((((((((((........)))))))).))).)))))....)))).))))...).))))))))).).))
```

---

## locus\_id: 271465

family\_id: 29

### **Targets:**

At5g19570(NM\_121962.1
): expressed protein

Location in genome: in an IGR, 318109 upstream of At4g06526, 26884 downstream of At4g06599

### Alignment between mature miRNA and predicted targets

```
        Extended mature miRNA:       cataggatgcggcggaggcggaggtg
        Target(rc):NM_121962.1       cgaAGAATGTGGCGGAGGCGGAGagg
```

### Precursor sequence and structure. Mature sequence in capital letters

```
gagcctgcatAGGATGCGGCGGAGGCGGAGgtggatagtgaggcggaactaccatatccggttcacgctgtggagcattcacctctggttctcctctcatagctggctc   
 (((((.((.((.((.(.((.((((.((((((((((..(((((.((((.........)))).)))))(((....))).)))))))))).)))))).))).)))).)))))
```

---

## locus\_id: 5070

family\_id: 28

### **Targets:**

At1g64170(NM\_105090.2
): cation/hydrogen exchanger, putative (CHX16)

Location in genome: Contained by At1g09190 (NM\_100789: . pentatricopeptide (PPR) repeat-containing protein) in an intron

1 homologs in brassica

### Alignment between mature miRNA and predicted targets

```
        Extended mature miRNA:       gagcgtaagctgctgcgtcttctccac
        Target(rc):NM_105090.2       aacCTTAAGCTGCTGCATCTTCTCgcc
```

### Precursor sequence and structure. Mature sequence in capital letters

```
gtggtagagaggagcagaggaggtttaatggaaatcgagCGTAAGCTGCTGCGTCTTCTCcacggccac   
 (((((.(((((((((((....((((((.(.(....).)...)))))).)))).)))))))....)))))
```

---

## locus\_id: 160644

family\_id: 27

### **Targets:**

At1g16610(NM\_101523.3
): arginine/serine-rich protein, putative (SR45)  
At1g16610(NM\_202115.1
): arginine/serine-rich protein, putative (SR45)

Location in genome: in an IGR, 11240 upstream of At2g22520, 12243 downstream of At2g22480

### Alignment between mature miRNA and predicted targets

```
        Extended mature miRNA:       gtcagctgagctttctcgtcatcactt
        Target(rc):NM_202115.1       tagAGCTGAGCTTTCTCAGCATCAgct
        Target(rc):NM_101523.3       tagAGCTGAGCTTTCTCAGCATCAgct
```

### Precursor sequence and structure. Mature sequence in capital letters

```
agtcAGCTGAGCTTTCTCGTCATCActtattagtaaatatagtctctatatttttgatgagtgatgattggaaatttcgttgact   
 (((((((.(((.(((((.(((((((((((((((.((((((((...))))))))))))))))))))))).))))).))))))))))
```

---

## locus\_id: 132313

family\_id: 25

### **Targets:**

At1g40230(NM\_103389.1
): hypothetical protein

Location in genome: in an IGR, 4193 upstream of At2g32140, 11005 downstream of At2g32180

### Alignment between mature miRNA and predicted targets

```
        Extended mature miRNA:       ggccgcaggcggcagcggctgcggtt
        Target(rc):NM_103389.1       tggCGCAAGCGGCAGCGGCTGTGtcg
```

### Precursor sequence and structure. Mature sequence in capital letters

```
ggcCGCAGGCGGCAGCGGCTGCGgttgcggcagcggcaacggtaacactgccgcaggttgttgttcgtttcgtcgccgccaacgttgccgcagccgctgtcgcaaccgcaggtt   
 .(((((..(((((((((((((((((.(((...(((((.((((.(((((.(((...))).).))))....)))).)))))...))).)))))))))))))))))....)).))).
```

---

## locus\_id: 362417

family\_id: 23

### **Targets:**

At2g17870(NM\_127341.3
): cold-shock DNA-binding family protein

Location in genome: in an IGR, 5769 upstream of At5g44890, 1862 downstream of At5g44930

### Alignment between mature miRNA and predicted targets

```
        Extended mature miRNA:       ggtcaagcacgaccacgactaccgag
        Target(rc):NM_127341.3       taaCAAGCACGACCACCACCACCacc
```

### Precursor sequence and structure. Mature sequence in capital letters

```
aagcagctcggtagtcgtggtcgtgctggatccattattgagctttggtCAAGCACGACCACGACTACCgagctgctt   
 (((((((((((((((((((((((((((.((.(((...........))))).)))))))))))))))))))))))))))
```

---

## locus\_id: 279310

family\_id: 22

### **Targets:**

At3g59230(NM\_115785.1
): F-box family protein

Location in genome: in an IGR, 2467 upstream of At4g13560, 17499 downstream of At4g13590

### Alignment between mature miRNA and predicted targets

```
        Extended mature miRNA:       tcgagcacatgacatccagatagaagctttgt
        Target(rc):NM_115785.1       tcaAGCACATGACAACCAGAAAGAAGCTTagc
```

### Precursor sequence and structure. Mature sequence in capital letters

```
taatttttcgAGCACATGACATCCAGATAGAAGCTTtgttaaacgtcatcaaagtcaagatttggtataaacgtttaacaaacttctatttggatgtcatgtgctcgaagagtta   
 (((((((((((((((((((((((((((((((((.((((((((((((.((((((.......))))))....)))))))))))))))))))))))))))))))))))))))))))))
```

---

## locus\_id: 279311

family\_id: 22

### **Targets:**

At3g59230(NM\_115785.1
): F-box family protein

Location in genome: in an IGR, 2476 upstream of At4g13560, 17496 downstream of At4g13590

### Alignment between mature miRNA and predicted targets

```
        Extended mature miRNA:       ttttcgagcacatgacatccagatag
        Target(rc):NM_115785.1       tccTCAAGCACATGACAACCAGAaag
```

### Precursor sequence and structure. Mature sequence in capital letters

```
ctaattttTCGAGCACATGACATCCAGAtagaagctttgttaaacgtcatcaaagtcaagatttggtataaacgtttaacaaacttctatttggatgtcatgtgctcgaagagttag   
 ((((((((((((((((((((((((((((((((((.((((((((((((.((((((.......))))))....))))))))))))))))))))))))))))))))))))))))))))))
```

---

## locus\_id: 24096

family\_id: 19

### **Targets:**

At5g05200(NM\_120602.2
): ABC1 family protein  
At5g05200(NM\_120602.2
): ABC1 family protein

Location in genome: in an IGR, 35286 upstream of At1g35910, 33553 downstream of At1g36020

### Alignment between mature miRNA and predicted targets

```
        Extended mature miRNA:       aatagagtttcctcaaaaatagaggaaaa
        Target(rc):NM_120602.2       caaAGAGTTTCCTCAAATATAGAGGAgac
```

### Precursor sequence and structure. Mature sequence in capital letters

```
cttctaaaatAGAGTTTCCTCAAAAATAGAGGAaaaaatagagatgaattgtagagatctctatttatagagacaaaaagtaaatatctctattttttctctattatagaggaaactctattttagagg   
 (((((((((((((((((((((...(((((((.((((((((((((((...........((((((....))))))...........)))))))))))))))))))))...)))))))))))))))))))))
```

---

## locus\_id: 335860

family\_id: 18

### **Targets:**

(NM\_123475.2
): expressed protein  
At5g41100(NM\_180781.1
): expressed protein

Location in genome: in an IGR, 1560 upstream of At5g08180, 6237 downstream of At5g08210

### Alignment between mature miRNA and predicted targets

```
        Extended mature miRNA:       gtgagagtcgctggaggcagcggttc
        Target(rc):NM_123475.2       atcAGAGACGCTGGAGTCAGCGGaag
        Target(rc):NM_180781.1       atcAGAGACGCTGGAGTCAGCGGaag
```

### Precursor sequence and structure. Mature sequence in capital letters

```
aagaagagtgAGAGTCGCTGGAGGCAGCGGttcatcgatctcttcctgtgaacacattaaaaatgtaaaagcatgaatagatcgataaacctctgcatccagcgtttgcctcttgtatctttctt   
 ..((((((..((((.(((((((.((((.((((.((((((((.(((.(((....((((.....))))....))).))).)))))))).)))).)))).))))))).....))))..).)))))...
```

---

## locus\_id: 344631

family\_id: 18

### **Targets:**

At5g19590(NM\_121964.2
): expressed protein

Location in genome: in an IGR, 2177 upstream of At5g23060, 1934 downstream of At5g23080

### Alignment between mature miRNA and predicted targets

```
        Extended mature miRNA:       aggaaagagtgaagtcgctggaggcag
        Target(rc):NM_121964.2       ggaAAAGAGAGAAGTCGCCGGAGGttt
```

### Precursor sequence and structure. Mature sequence in capital letters

```
atgaggAAAGAGTGAAGTCGCTGGAGGcagcggttcatcgatcaattcctgtgaatatttatttttgtttacaaaagcaagaatcgatcgataaacctctgcatccagcgctgcttgctcttcat   
 (((((((..((((..((.(((((((.((((.((((.(((((((.((((.((((((((........)))))))).......)))).))))))).)))).)))).)))))))))))))..)))))))
```

---

## locus\_id: 74205

family\_id: 17

### **Targets:**

At1g62930(NM\_104971.1
): pentatricopeptide (PPR) repeat-containing protein  
At1g63130(NM\_104991.2
): pentatricopeptide (PPR) repeat-containing protein

Location in genome: in an IGR, 8832 upstream of At1g32600, 308 downstream of At1g32580

### Alignment between mature miRNA and predicted targets

```
        Extended mature miRNA:       ttgtttatgagagtattataagtcactacatt
        Target(rc):NM_104971.1       cccTTTATAAGAGTATTGTAAGTCACTACgtt
        Target(rc):NM_104991.2       cccTTTATAAGAGTATTGTAAGTCACTACgtt
```

### Precursor sequence and structure. Mature sequence in capital letters

```
ttgTTTATGAGAGTATTATAAGTCACTACatttggtaagcaaagtgttgtttctcaaacgaagtgacttatgataatctcatgaatgg   
 (..(((((((((.((((((((((((((.(.((((..((((((....))))))..)))).).)))))))))))))).)))))))))..)
```

---

## locus\_id: 325366

family\_id: 15

### **Targets:**

At4g10560(NM\_117124.1
): DC1 domain-containing protein

Location in genome: in an IGR, 9717 upstream of At4g30990, 5939 downstream of At4g30960

1 homologs in brassica

### Alignment between mature miRNA and predicted targets

```
        Extended mature miRNA:       gacagaagagagtgagcacatgcagg
        Target(rc):NM_117124.1       catAAAAAAGAGTGAGCACATGCtta
```

### Precursor sequence and structure. Mature sequence in capital letters

```
aactgacAGAAGAGAGTGAGCACATGCaggcactgttatgtgtctataactttgcgtgtgcgtgctcacctctctttctgtcagtt   
 (((((((((((((((((((((((..(((.(((..((((((....))))))..))).)))..))))))).))))).)))))))))))
```

---

## locus\_id: 393202

family\_id: 15

### **Targets:**

At3g15270(NM\_112390.2
): squamosa promoter-binding protein-like 5 (SPL5)

Location in genome: in an IGR, 7126 upstream of At5g26160, 4488 downstream of At5g26140

1 homologs in brassica

### Alignment between mature miRNA and predicted targets

```
        Extended mature miRNA:       gatggtgacagaagagagtgagcaca
        Target(rc):NM_112390.2       gtaGATGACAGAAGAGAGAGAGCggg
```

### Precursor sequence and structure. Mature sequence in capital letters

```
gatGGTGACAGAAGAGAGTGAGCacacatggtggctttcttgcatatttgaaggttccatgcttgaagctatgtgtgctcactctctatccgtcacccccttc   
 ...((((((.((((((((((((((((((((((....(((..((((.............))))..))))))))))))))))))))))).)).))))))......
```

---

## locus\_id: 272325

family\_id: 14

### **Targets:**

At1g08135(NM\_179279.1
): cation/hydrogen exchanger (CHX6b)

Location in genome: in an IGR, 97948 upstream of At4g06634, 24120 downstream of At4g06672

### Alignment between mature miRNA and predicted targets

```
        Extended mature miRNA:       tagactcttgtggttggacgactcgg
        Target(rc):NM_179279.1        tcACTCTTGTTGTTGGACGACTaac
```

### Precursor sequence and structure. Mature sequence in capital letters

```
gctagACTCTTGTGGTTGGACGACTcgggcatacgaattcatttcggaaaccttggttggccgactgcgggcgtctacgt   
 (((((((.((((..(((((.(((((.(((....((((.....))))....))).))))).)))))..)))).))))).))
```

---

## locus\_id: 353282

family\_id: 14

### **Targets:**

At1g08135(NM\_179279.1
): cation/hydrogen exchanger (CHX6b)

Location in genome: in an IGR, 4366 upstream of At5g34780, 8373 downstream of At5g34820

### Alignment between mature miRNA and predicted targets

```
        Extended mature miRNA:       tagactcttgtggttggacgactcgg
        Target(rc):NM_179279.1        tcACTCTTGTTGTTGGACGACTaac
```

### Precursor sequence and structure. Mature sequence in capital letters

```
gctagACTCTTGTGGTTGGACGACTcgggcatacgaatttgtttcggaaacctgggttggccgactgcgggcgtctaac   
 ..(((((.((((..(((((.(((((((((....((((.....))))....))))))))).)))))..)))).)))))..
```

---

## locus\_id: 397026

family\_id: 14

### **Targets:**

At1g08135(NM\_179279.1
): cation/hydrogen exchanger (CHX6b)

Location in genome: in an IGR, 17421 upstream of At5g29050, 1539 downstream of At5g29037

### Alignment between mature miRNA and predicted targets

```
        Extended mature miRNA:       tagactcttgtggttggacgactcgg
        Target(rc):NM_179279.1        tcACTCTTGTTGTTGGACGACTaac
```

### Precursor sequence and structure. Mature sequence in capital letters

```
tagACTCTTGTGGTTGGACGACTcgggcatacgaatttgttttggaaacgtgggttgaccgactgcgggcgtcta   
 (((((.((((..(((((.(((((((.(....((((.....))))....).))))))).)))))..)))).)))))
```

---

## locus\_id: 161161

family\_id: 11

### **Targets:**

At3g13820(NM\_112235.1
): F-box family protein

Location in genome: in an IGR, 4793 upstream of At2g23320, 897 downstream of At2g23310

### Alignment between mature miRNA and predicted targets

```
        Extended mature miRNA:       aaaaatatctacctgattaatggacggaatga
        Target(rc):NM_112235.1       aggAATATCGACCTGATTAATGCACGGAAcga
```

### Precursor sequence and structure. Mature sequence in capital letters

```
acttcgtttttcggtcgttaaattaggtaaatatttttgaccatatcgaaatacaaagtcatccacatttagatgtggtcaaaAATATCTACCTGATTAATGGACGGAAtgacaaagt   
 ((((.((((((((..((((((.(((((((.(((((((((((((((((.((((..............)))).))))))))))))))))).)))))))))))))..))))).))).))))
```

---

## locus\_id: 161162

family\_id: 11

### **Targets:**

At3g13820(NM\_112235.1
): F-box family protein

Location in genome: in an IGR, 4789 upstream of At2g23320, 904 downstream of At2g23310

### Alignment between mature miRNA and predicted targets

```
        Extended mature miRNA:       tctacctgattaatggacggaatgacaaa
        Target(rc):NM_112235.1       tcgACCTGATTAATGCACGGAACGACgta
```

### Precursor sequence and structure. Mature sequence in capital letters

```
gatgacttcgtttttcggtcgttaaattaggtaaatatttttgaccatatcgaaatacaaagtcatccacatttagatgtggtcaaaaatatctACCTGATTAATGGACGGAATGACaaagtcatt   
 ((((((((.((((((((..((((((.(((((((.(((((((((((((((((.((((..............)))).))))))))))))))))).)))))))))))))..))))).))).))))))))
```

---

## locus\_id: 366174

family\_id: 10

### **Targets:**

At5g39200(NM\_123282.1
): hypothetical protein  
At5g39140(NM\_123276.1
): hypothetical protein  
At5g39170(NM\_123279.1
): hypothetical protein

Location in genome: in an IGR, 6037 upstream of At5g50000, 31425 downstream of At5g50120

### Alignment between mature miRNA and predicted targets

```
        Extended mature miRNA:       tgaaccggtatctggtccggtttctaa
        Target(rc):NM_123276.1       attACCGGTTTTTGGTCCGGTTTCcac
        Target(rc):NM_123279.1       attACCGGTTTTTGGTCCGGTTTCcac
        Target(rc):NM_123282.1       attACCGGTTTTTGGTCCGGTTTCcac
```

### Precursor sequence and structure. Mature sequence in capital letters

```
tgttttaaaaaccggaccggtccggtcctgcagaccaagaatcgttcatgtggctgaaattgggatgaACCGGTATCTGGTCCGGTTTCtaaaaca   
 .((((((.((((((((((((.(((((....((..((((...(((..(....)..)))..))))..)).)))))...)))))))))))).)))))).
```

---

## locus\_id: 333080

family\_id: 9

### **Targets:**

At2g20610(NM\_201760.1
): aminotransferase, putative  
At2g20610(NM\_127622.2
): aminotransferase, putative

Location in genome: in an IGR, 26663 upstream of At5g03630, 11788 downstream of At5g03690

### Alignment between mature miRNA and predicted targets

```
        Extended mature miRNA:       ataaccccgatggttatcctcaccact
        Target(rc):NM_127622.2       tcgACTCCGATGGTTATCCTCATCcag
        Target(rc):NM_201760.1       tcgACTCCGATGGTTATCCTCATCcag
```

### Precursor sequence and structure. Mature sequence in capital letters

```
gtggtgtcgggggaccgttagggaatgatagaccgtgaccccattcacgggcaatctctaatattcataACCCCGATGGTTATCCTCACCac   
 ((((((..((..((((((..(((.((((((((((((((......))))))......))))....))))..)))..))))))..)).))))))
```

---

## locus\_id: 368659

family\_id: 8

### **Targets:**

At2g25830(NM\_128143.2
): YebC-related

Location in genome: in an IGR, 3773 upstream of At5g53780, 4409 downstream of At5g53810

### Alignment between mature miRNA and predicted targets

```
        Extended mature miRNA:       gagaagggacgagaaagtgttggttat
        Target(rc):NM_128143.2       ggaAAGAGACGAGAGAGTGTTGGTcat
```

### Precursor sequence and structure. Mature sequence in capital letters

```
gttttgagAAGGGACGAGAAAGTGTTGGTtatcgcatcgaaaatggatcggaatgttagtttatgtgtgctattgcatatatatgcgataaccaacactttctcgtcccttctcaaaac   
 (((((((((((((((((((((((((((((((((((.((((.......))))..........(((((((((....))))))))).)))))))))))))))))))))))))))))))))))
```

---

## locus\_id: 414702

family\_id: 8

### **Targets:**

At2g02980(NM\_126350.2
): pentatricopeptide (PPR) repeat-containing protein

Location in genome: in an IGR, 2451 upstream of At5g53800, 401 downstream of At5g53790

### Alignment between mature miRNA and predicted targets

```
        Extended mature miRNA:       tgttttgagaagggacgagaaagtgttgg
        Target(rc):NM_126350.2       agcTTTGAGAAGAGACGGGAAAGTGTagt
```

### Precursor sequence and structure. Mature sequence in capital letters

```
tgtTTTGAGAAGGGACGAGAAAGTGTtggttatcgcatatatatgcaatagcacacataaactaacattccgatccattttcgatgcgataaccaacactttctcgtcccttctcaaaacg   
 .(((((((((((((((((((((((((((((((((((((.....(((....))).................(((.......)))))))))))))))))))))))))))))))))))))))).
```

---

## locus\_id: 414703

family\_id: 8

### **Targets:**

At2g25830(NM\_128143.2
): YebC-related

Location in genome: in an IGR, 2447 upstream of At5g53800, 407 downstream of At5g53790

### Alignment between mature miRNA and predicted targets

```
        Extended mature miRNA:       gagaagggacgagaaagtgttggttat
        Target(rc):NM_128143.2       ggaAAGAGACGAGAGAGTGTTGGTcat
```

### Precursor sequence and structure. Mature sequence in capital letters

```
tgttttgagAAGGGACGAGAAAGTGTTGGTtatcgcatatatatgcaatagcacacataaactaacattccgatccattttcgatgcgataaccaacactttctcgtcccttctcaaaacg   
 .(((((((((((((((((((((((((((((((((((((.....(((....))).................(((.......)))))))))))))))))))))))))))))))))))))))).
```

---

## locus\_id: 186526

family\_id: 7

### **Targets:**

At5g12840(NM\_180480.1
): CCAAT-binding transcription factor (CBF-B/NF-YA) family protein  
(NM\_121287.3
): CCAAT-binding transcription factor (CBF-B/NF-YA) family protein

Location in genome: in an IGR, 1984 upstream of At3g14380, 5715 downstream of At3g14400

### Alignment between mature miRNA and predicted targets

```
        Extended mature miRNA:       ttgagccaaggatgacttgccggtttaaa
        Target(rc):NM_180480.1       aaaAGCCAAGAATGATTTGCCGGTTTgag
        Target(rc):NM_121287.3       aaaAGCCAAGAATGATTTGCCGGTTTgag
```

### Precursor sequence and structure. Mature sequence in capital letters

```
aatggaattgAGCCAAGGATGACTTGCCGGTTTaaacccaaccggtttatgaccattgatttggtctcattcacaatctgttgattcgtgtctggcaagttgaccttggctctgcttcgtt   
 ((((((...(((((((((.(((((((((((..((((((.....)))))).(((((......))))).....(((((((....)))).))).))))))))))).)))))))))...))))))
```

---

## locus\_id: 392209

family\_id: 7

### **Targets:**

At3g22640(NM\_113163.2
): cupin family protein

Location in genome: in an IGR, 3443 upstream of At5g24830, 2219 downstream of At5g24820

### Alignment between mature miRNA and predicted targets

```
        Extended mature miRNA:       gccaaggatgacttgccggaacgttg
        Target(rc):NM_113163.2       tgaATGGATGACCTGCCGGAACGata
```

### Precursor sequence and structure. Mature sequence in capital letters

```
aatgcagccAAGGATGACTTGCCGGAACGttgttaaccatgcatatgaataatgtgatgattaattatgtgatgaacatatttctggcaagttgtccttcggctacatt   
 ((((.(((((((((..(((((((((((...((((...(((.(((((((.((((......)))).))))))))))))))...)))))))))))..))))).)))).))))
```

---

## locus\_id: 264854

family\_id: 6

### **Targets:**

At5g37020(NM\_123060.2
): auxin-responsive factor (ARF8)

Location in genome: in an IGR, 5825 upstream of At3g63400, 188 downstream of At3g63370

1 homologs in rice

### Alignment between mature miRNA and predicted targets

```
        Extended mature miRNA:       gtgaagctgccagcatgatctatctt
        Target(rc):NM_123060.2       tacAAGCTGCCAGCCTGATCTAAggg
```

### Precursor sequence and structure. Mature sequence in capital letters

```
ggaacaagtgAAGCTGCCAGCATGATCTATctttggttaagagatgaatgtggaaacatattgcttaaacccaagctaggtcatgctctgacagcctcactccttcc   
 ((((..(((((.(((((.(((((((((((.(((.((((..(((...((((((....)))))).))).)))).))).)))))))))))..).)))).)))))..))))
```

---

## locus\_id: 163525

family\_id: 5

### **Targets:**

At3g51570(NM\_115016.1
): disease resistance protein (TIR-NBS-LRR class), putative

Location in genome: in an IGR, 4862 upstream of At2g26230, 417 downstream of At2g26210

### Alignment between mature miRNA and predicted targets

```
        Extended mature miRNA:       cgactcgttcaagcaccagctcgaaga
        Target(rc):NM_115016.1       tagCTCGTTCAAGCACCATTTCGAttc
```

### Precursor sequence and structure. Mature sequence in capital letters

```
catcgaCTCGTTCAAGCACCAGCTCGAagaagcttagctaatttatcttagaaaataatgaaaaagctatgcttctcaagaaggtgcatgaacaagttgatg   
 ((((((((.(((((.(((((..((.((.(((((.(((((..(((((..(.....)..)))))..))))).))))))).))..))))).))))).))))))))
```

---

## locus\_id: 177438

family\_id: 4

### **Targets:**

At5g05030(NM\_120585.2
): expressed protein

Location in genome: in an IGR, 942 upstream of At2g47590, 1963 downstream of At2g47580

2 homologs in brassica

### Alignment between mature miRNA and predicted targets

```
        Extended mature miRNA:       cgtacttaacttctccaacatgagctct
        Target(rc):NM_120585.2       taaACTTAACTTCTCCAACACGCGCcga
```

### Precursor sequence and structure. Mature sequence in capital letters

```
gggtgagaatctccatgttggagaagcagggcacgtgcaaaccaacaaacacgaaatccgtctcatttgcttatttgcacgtACTTAACTTCTCCAACATGAGCtcttcaccc   
 ((((((..(.(((.((((((((((((..(((.(((((((((....((((.(((.....)))....))))....))))))))).)))..))))))))))))))).)..))))))
```

---

## locus\_id: 177439

family\_id: 4

### **Targets:**

At1g61100(NM\_104791.2
): disease resistance protein (TIR class), putative

Location in genome: in an IGR, 938 upstream of At2g47590, 1967 downstream of At2g47580

2 homologs in brassica

### Alignment between mature miRNA and predicted targets

```
        Extended mature miRNA:       cttaacttctccaacatgagctcttcac
        Target(rc):NM_104791.2       agaAACTTCTCCCTCATGAGCTCTTttt
```

### Precursor sequence and structure. Mature sequence in capital letters

```
gggtgagaatctccatgttggagaagcagggcacgtgcaaaccaacaaacacgaaatccgtctcatttgcttatttgcacgtacttAACTTCTCCAACATGAGCTCTTcaccc   
 ((((((..(.(((.((((((((((((..(((.(((((((((....((((.(((.....)))....))))....))))))))).)))..))))))))))))))).)..))))))
```

---

## locus\_id: 91579

family\_id: 1

### **Targets:**

At3g13830(NM\_112236.1
): F-box family protein

Location in genome: in an IGR, 5827 upstream of At1g60090, 7946 downstream of At1g60060

### Alignment between mature miRNA and predicted targets

```
        Extended mature miRNA:       aatctctctgttgtgaagtcaaacat
        Target(rc):NM_112236.1       aaaCTCTCCGTCGTGAAGTCAAAaca
```

### Precursor sequence and structure. Mature sequence in capital letters

```
atgtcgaaatCTCTCTGTTGTGAAGTCAAAcatgagtatgaattaacattaatggatctttttgattcataatactcatgtttggtttcacagtagatagatatcgacgt   
 (((((((.((((.(((((((((((..(((((((((((((............(((((((.....))))))).)))))))))))))..))))))))))).)))).)))))))
```

---

## locus\_id: 30670

family\_id: 153

### **Targets:**

At1g13020(NM\_101172.2
): eukaryotic translation initiation factor, putative (EIF4B5)  
At3g26400(NM\_113547.1
): eukaryotic translation initiation factor 4B, putative/ eIF-4B, putative

Location in genome: in an IGR, 1206 upstream of At1g48090, 9921 downstream of At1g48110

### Alignment between mature miRNA and predicted targets

```
        Extended mature miRNA:       taattggtcttggtttcaccaaatttaa
        Target(rc):NM_113547.1       cctTTGGTCTCGGTTTCACCACATTatt
        Target(rc):NM_101172.2       cctTTGGTCTCGGTTTCACCACATTctc
```

### Precursor sequence and structure. Mature sequence in capital letters

```
gtctcttaactttgatgaaacctaggcaattgtctcttagttaagagataaTTGGTCTTGGTTTCACCAAATTtaagagac   
 (((((((((.((((.(((((((.(((((((((((((((....))))))))))).)))).))))))).)))).)))))))))
```

---

## locus\_id: 101406

family\_id: 153

### **Targets:**

At1g13020(NM\_101172.2
): eukaryotic translation initiation factor, putative (EIF4B5)  
At3g26400(NM\_113547.1
): eukaryotic translation initiation factor 4B, putative/ eIF-4B, putative

Location in genome: in an IGR, 1273 upstream of At1g72910, 538 downstream of At1g72900

### Alignment between mature miRNA and predicted targets

```
        Extended mature miRNA:       taattggtcttggtttcaccaaatttaa
        Target(rc):NM_113547.1       cctTTGGTCTCGGTTTCACCACATTatt
        Target(rc):NM_101172.2       cctTTGGTCTCGGTTTCACCACATTctc
```

### Precursor sequence and structure. Mature sequence in capital letters

```
gtctcttaaatttgatgaaacttaggcaaattgtctcttagttaagagataaTTGGTCTTGGTTTCACCAAATTtaagagac   
 ((((((((((((((.(((((((.((((.(((((((((((....))))))))))).)))).))))))).))))))))))))))
```

---

## locus\_id: 364394

family\_id: 150

### **Targets:**

At4g17486(NM\_117853.2
): expressed protein  
At3g48210(NM\_114691.1
): expressed protein

Location in genome: in an IGR, 11285 upstream of At5g47760, 58 downstream of At5g47810

### Alignment between mature miRNA and predicted targets

```
        Extended mature miRNA:       tggttgagcaggagcttgagctctgc
        Target(rc):NM_114691.1       gctTTGAGCAGTGGCTTGAGCTCgct
        Target(rc):NM_117853.2       cacTTGAACATGAGCTTGAGCTCaat
```

### Precursor sequence and structure. Mature sequence in capital letters

```
catggctcatgcttgttgctgaaccgttttcttaagcacataagagagagtggTTGAGCAGGAGCTTGAGCTCtg   
 ((.((((((.((((.(((((.((((((((((((..........)))))))))))).))))))))).)))))).))
```

---

## locus\_id: 167444

family\_id: 147

### **Targets:**

At5g60530(NM\_125446.2
): late embryogenesis abundant protein-related / LEA protein-related  
At5g18280(NM\_121833.2
): apyrase (APY2)

Location in genome: in an IGR, 998 upstream of At2g32160, 438 downstream of At2g32150

### Alignment between mature miRNA and predicted targets

```
        Extended mature miRNA:       cagtgttaccgttgccgctgccgcaa
        Target(rc):NM_125446.2       catTGTTACCGTTGCCTTTGCCGCcat
        Target(rc):NM_121833.2       gtaTCTTACCGTTGCCGTTGCCGgta
```

### Precursor sequence and structure. Mature sequence in capital letters

```
ctgcggttgcgacagcggctgcggcaacgttggcggcgacgaaacgaacaacaacctgcggcagTGTTACCGTTGCCGCTGCCGcaaccgcag   
 (((((((((((.(((((((.((((.((((...((.(((..................))).))..)))).)))).))))))).)))))))))))
```

---

## locus\_id: 132833

family\_id: 143

### **Targets:**

At1g60680(NM\_104751.3
): aldo/keto reductase family protein  
At1g65620(NM\_105235.3
): LOB domain protein 6 / lateral organ boundaries domain protein 6 (LBD6) / asymmetric leaves2 (AS2)

Location in genome: in an IGR, 3355 upstream of At2g32980, 10671 downstream of At2g33020

### Alignment between mature miRNA and predicted targets

```
        Extended mature miRNA:       gagtggtggaggagagagatgacgtga
        Target(rc):NM_104751.3       gcaTGGTGGAGGAGAGCGATGGCGTcag
        Target(rc):NM_105235.3       tgaTGTTGGAGGAGAGAGATGACGccg
```

### Precursor sequence and structure. Mature sequence in capital letters

```
tcactagtctctctctatttctctgctctcgcaggagagggatatatttgaaagctctgtggagTGGTGGAGGAGAGAGATGACGtga   
 ((((..((((((((((..(((.(..(((.(((((.((.................))))))))))..).)))))))))))))...))))
```

---

## locus\_id: 394354

family\_id: 139

### **Targets:**

At5g61430(NM\_125536.2
): no apical meristem (NAM) family protein  
(NM\_180458.1
): no apical meristem (NAM) family protein  
At5g07680(NM\_120850.2
): no apical meristem (NAM) family protein  
At3g04980(NM\_111370.1
): DNAJ heat shock N-terminal domain-containing protein

Location in genome: in an IGR, 3129 upstream of At5g27810, 8875 downstream of At5g27800

2 homologs in brassica

### Alignment between mature miRNA and predicted targets

```
        Extended mature miRNA:       acacttgatggagaagcagggcacgt
        Target(rc):NM_111370.1       tgtCTGGATGGAGAAGCAGCGCAagc
        Target(rc):NM_180458.1          ttgGTTGGAGAAGCAGGGCACGTaaa
        Target(rc):NM_125536.2          ttgGTTGGAGAAGCAGGGCACGTaga
        Target(rc):NM_120850.2          ttgGTTGGAGAAGCAGGGCACGTaaa
```

### Precursor sequence and structure. Mature sequence in capital letters

```
gagtaacaCTTGATGGAGAAGCAGGGCAcgtgcgaacacaaatgaaatcgatcggtacttgttgatcatattttcgcacgtgttctactactccaacacgtgtctctc   
 (((..((((.((.(((((.((.((((((((((((((..(....).....((((((......)))))).....)))))))))))))).)).))))).)).))))..)))
```

---

## locus\_id: 363784

family\_id: 138

### **Targets:**

At1g77850(NM\_106434.1
): transcriptional factor B3 family protein  
At2g28350(NM\_128394.3
): auxin-responsive factor (ARF10)

Location in genome: in an IGR, 18412 upstream of At5g46800, 2843 downstream of At5g46860

### Alignment between mature miRNA and predicted targets

```
        Extended mature miRNA:       ttatgcctggctccctgtatgccacg
        Target(rc):NM_128394.3       gctTGCCTGGCTCCCTGTATTCCtgc
        Target(rc):NM_106434.1       tatTGCCTGGCTCCCTGCATGCCAgca
```

### Precursor sequence and structure. Mature sequence in capital letters

```
gttaTGCCTGGCTCCCTGTATGCCacgagtggataccgattttggttttaaaatcggctgccggtggcgtacaaggagtcaagcatgac   
 (((((((.(((((((.((((((((((..(..(...(((((((((....))))))))))..)..)))))))))).))))))).)))))))
```

---

## locus\_id: 398770

family\_id: 137

### **Targets:**

At4g08098(NM\_148283.2
): hypothetical protein  
At3g32180(NM\_114040.1
): hypothetical protein

Location in genome: in an IGR, 10455 upstream of At5g33320, 7135 downstream of At5g33303

### Alignment between mature miRNA and predicted targets

```
        Extended mature miRNA:       cgttgcctctcatcgcataagcggcggtg
        Target(rc):NM_148283.2       cgcTGCCTCTCATCGCACATGCGGtgg
        Target(rc):NM_114040.1       cgcTGCCTCTCATCACATAGGCGGCGatg
```

### Precursor sequence and structure. Mature sequence in capital letters

```
catagcccttaccttcctctcgatgcgggcaattcgggccttgagcgtttgcctcttgaggcccaaccgtTGCCTCTCATCGCATAAGCGGCGgtg   
 (((.(((((((.........(((((.(((((((..((((((..((.(.....).))..))))))....)))))).).)))))..)))).))).)))
```

---

## locus\_id: 194370

family\_id: 136

### **Targets:**

At3g57560(NM\_115616.2
): aspartate/glutamate/uridylate kinase family protein  
At1g03475(NM\_100230.3
): coproporphyrinogen III oxidase, putative / coproporphyrinogenase, putative / coprogen oxidase, putative

Location in genome: in an IGR, 3021 upstream of At3g24390, 2170 downstream of At3g24420

### Alignment between mature miRNA and predicted targets

```
        Extended mature miRNA:       gtggtgatgtcagtgccaccaccgaa
        Target(rc):NM_115616.2        ggtTGATGTCAGGACCACCACCGtga
        Target(rc):NM_100230.3       ggaGTGAAATCAGTGCCACCACCaaa
```

### Precursor sequence and structure. Mature sequence in capital letters

```
ggagtcccttacggtggtcagcaacaacaaaatgcatcacgacgatcagatcacgtgGTGATGTCAGTGCCACCACCgaagtctcc   
 ((((...(((.(((((((..(((.........((((((((.(((.........))).)))))).)).))).)))))))))).))))
```

---

## locus\_id: 285825

family\_id: 135

### **Targets:**

At2g19500(NM\_127508.2
): FAD-binding domain-containing protein / cytokinin oxidase family protein  
(NM\_180532.1
): cytokinin oxidase, putative (CKX5)

Location in genome: in an IGR, 10208 upstream of At4g21570, 9127 downstream of At4g21630

### Alignment between mature miRNA and predicted targets

```
        Extended mature miRNA:       agaatgaggttgagccaaggatgactt
        Target(rc):NM_180532.1       acgAAGAGGTTGAGCCAAGGGTGAggc
        Target(rc):NM_127508.2        cgtAGAGGTTAAGCCAAGGATGAgga
```

### Precursor sequence and structure. Mature sequence in capital letters

```
agaatagagaATGAGGTTGAGCCAAGGATGActtgccgggtttttttaccaatgaatctaattaactgattctggtgtccggcaagttgaccttggctctgtttccttctcttct   
 (((..((((((.((((..(((((((((.((((((((((((......(((((..(((((.........)))))))))))))))))))))).)))))))))..)))).)))))))))
```

---

## locus\_id: 402290

family\_id: 134

### **Targets:**

At1g04090(NM\_100290.2
): expressed protein  
At5g43950(NM\_123762.2
): expressed protein

Location in genome: in an IGR, 10270 upstream of At5g36905, 357 downstream of At5g36900

### Alignment between mature miRNA and predicted targets

```
        Extended mature miRNA:       ggatgaggaggctacggtaaccaccg
        Target(rc):NM_123762.2       cctTGAGGAGGCTGCGGTAACCAgaa
        Target(rc):NM_100290.2       cctTCAGGAGGCTGCGGTAACCAgaa
```

### Precursor sequence and structure. Mature sequence in capital letters

```
tggggaTGAGGAGGCTACGGTAACCAccgctccttagagacagggtcataatgccgtatttcctcacccta   
 (((((.((((((((.((((((((((....(((....)))....))).....))))))))))))))))))))
```

---

## locus\_id: 127193

family\_id: 132

### **Targets:**

At2g16630(NM\_127215.3
): proline-rich family protein  
At1g09750(NM\_100847.2
): chloroplast nucleoid DNA-binding protein-related

Location in genome: in an IGR, 13090 upstream of At2g24970, 19692 downstream of At2g25050

### Alignment between mature miRNA and predicted targets

```
        Extended mature miRNA:       ccattgaaggacctgaagctatgctt
        Target(rc):NM_127215.3        ctgTGCAGGACCTGAAGCAATGCTgca
        Target(rc):NM_100847.2       aagTAGAAGGACCTGAAGCTAGGgag
```

### Precursor sequence and structure. Mature sequence in capital letters

```
acacggaagcatagctccatatccttcaatggaggtgtggtccttcaacaaaaatacccccctcttgaaactctgtttcaccacacctccaTTGAAGGACCTGAAGCTATGcttccttgt   
 (((.((((((((((((.((..((((((((((((((((((((.........................(((((...)))))))))))))))))))))))))..)).)))))))))))).)))
```

---

## locus\_id: 84011

family\_id: 130

### **Targets:**

At1g62670(NM\_104944.1
): pentatricopeptide (PPR) repeat-containing protein  
At1g64580(NM\_105133.1
): pentatricopeptide (PPR) repeat-containing protein

Location in genome: in an IGR, 9336 upstream of At1g48280, 16846 downstream of At1g48230

### Alignment between mature miRNA and predicted targets

```
        Extended mature miRNA:       cattgaaagtgactacatcggggttccg
        Target(rc):NM_104944.1       cgcTGAAAGTGAATACATCAGGGTTgat
        Target(rc):NM_105133.1       cagTGAAAGTGACAACATCAGGGTTtat
```

### Precursor sequence and structure. Mature sequence in capital letters

```
aatgcatTGAAAGTGACTACATCGGGGTTccgattttttttgttcttcatatgatgaagcggaaacagtaatcaaccctggtttagtcactttcactgcatt   
 ((((((.((((((((((((.(((((((((..((((....(((((((((((...))))))....))))).))))))))))))).)))))))))))).))))))
```

---

## locus\_id: 71306

family\_id: 126

### **Targets:**

At5g37840(NM\_123143.2
): expressed protein  
At1g66480(NM\_105319.2
): expressed protein

Location in genome: in an IGR, 11 upstream of At1g29710, 9485 downstream of At1g29670

### Alignment between mature miRNA and predicted targets

```
        Extended mature miRNA:       tgttctccgtcaccggagttttacca
        Target(rc):NM_105319.2       ctcTCGCCGTCACCGGAGTTTTAatt
        Target(rc):NM_123143.2      tcaCTCGCCGTCACCGGAGTTTTgag
```

### Precursor sequence and structure. Mature sequence in capital letters

```
tttgtgtTCTCCGTCACCGGAGTTTTAccatcgtgaaatggtgcgattgtggtgtgggaagctcaga   
 ((((.(((..(((.(((((.((((.((((((......)))))).)))).))))))))..))).))))
```

---

## locus\_id: 177435

family\_id: 125

### **Targets:**

At2g16950(NM\_127248.3
): importin beta-2 subunit family protein  
At1g74160(NM\_106076.2
): expressed protein

Location in genome: in an IGR, 1015 upstream of At2g47590, 1890 downstream of At2g47580

1 homologs in brassica

### Alignment between mature miRNA and predicted targets

```
        Extended mature miRNA:       gaatctccatgttggagaagcagggcac
        Target(rc):NM_106076.2       gttTCTCCATCTTGGAGAAGCCGGGtcc
        Target(rc):NM_127248.3       cccTCTCCATGTTGAAGAATCAGGcct
```

### Precursor sequence and structure. Mature sequence in capital letters

```
gggtgagaaTCTCCATGTTGGAGAAGCAGGGcacgtgcaaaccaacaaacacgaaatccgtctcatttgcttatttgcacgtacttaacttctccaacatgagctcttcaccc   
 ((((((..(.(((.((((((((((((..(((.(((((((((....((((.(((.....)))....))))....))))))))).)))..))))))))))))))).)..))))))
```

---

## locus\_id: 148381

family\_id: 117

### **Targets:**

At4g11211(NM\_202805.1
): expressed protein  
At1g79630(NM\_106612.3
): protein phosphatase 2C family protein / PP2C family protein  
At1g79630(NM\_202451.1
): protein phosphatase 2C family protein / PP2C family protein

Location in genome: in an IGR, 1431 upstream of At2g07748, 2902 downstream of At2g07746

### Alignment between mature miRNA and predicted targets

```
        Extended mature miRNA:       agatgttgaggcttccatggctatat
        Target(rc):NM_202451.1       attGGTTGAGACTTCCATGGCTActg
        Target(rc):NM_202805.1       tatTGATGAAGCTTCCATGGCTAaaa
        Target(rc):NM_106612.3        ttgGTTGAGACTTCCATGGCTACtga
```

### Precursor sequence and structure. Mature sequence in capital letters

```
gcagaagaTGTTGAGGCTTCCATGGCTAtattctggactcctggtactagagtgaagatggccatggagactgaggactcttcgc   
 ((.(((((.(((..((..((((((((((((((((((((.....)).)))))))....)))))))))))..))...))))))))))
```

---

## locus\_id: 26477

family\_id: 115

### **Targets:**

At5g33240(NM\_122848.1
): hypothetical protein  
At1g42740(NM\_148529.1
): hypothetical protein

Location in genome: in an IGR, 6355 upstream of At1g41820, 12098 downstream of At1g41830

### Alignment between mature miRNA and predicted targets

```
        Extended mature miRNA:       ctggtgtcgatcgacactggtgtctagt
        Target(rc):NM_148529.1       gcaGTGACGGTCGACACTGGTGTCTggt
        Target(rc):NM_122848.1       agaGTGTCGATCGACACAGGTGTggt
```

### Precursor sequence and structure. Mature sequence in capital letters

```
gcaaggctggactgggttggtgtcgatcgacacacaattgatgtaggttgacactgtcgggttctatagtgtctgtgttggctgGTGTCGATCGACACTGGTGTCTagtctgc   
 (((.((((((((....(..((((((((((((((.((.(..(..((....((((((((.(....).))))))))))..)..).))))))))))))))))..).)))))))))))
```

---

## locus\_id: 39261

family\_id: 113

### **Targets:**

At2g25980(NM\_128157.3
): jacalin lectin family protein  
At1g57570(NM\_104554.2
): jacalin lectin family protein

Location in genome: in an IGR, 13699 upstream of At1g61200, 12013 downstream of At1g61255

### Alignment between mature miRNA and predicted targets

```
        Extended mature miRNA:       ccatcatggtcggattcatcatcccg
        Target(rc):NM_128157.3       tttTCATGGTCGGCTCCATCATCCCatt
        Target(rc):NM_104554.2       tcgTCATGGTCGGATCCATCATCaaa
```

### Precursor sequence and structure. Mature sequence in capital letters

```
ataagtgggatgacggatctgaccatgatggtgtttcgatccctggacaataactacatcatacataaatttctgcaacaccaTCATGGTCGGATTCATCATCccgcttat   
 (((((((((((((.(((((((((((((((((((((.(((................................)).).))))))))))))))))))))).)))))))))))))
```

---

## locus\_id: 92546

family\_id: 110

### **Targets:**

At1g60110(NM\_104703.1
): jacalin lectin family protein  
At5g38550(NM\_123216.2
): jacalin lectin family protein

Location in genome: in an IGR, 4292 upstream of At1g61240, 1673 downstream of At1g61215

### Alignment between mature miRNA and predicted targets

```
        Extended mature miRNA:       tggtcagatccgtcatcccacttatg
        Target(rc):NM_104703.1      tttGTCGGATCCATCATCCCACTTAgat
        Target(rc):NM_123216.2       tgtTCAGATCCATCATCCCACTCccg
```

### Precursor sequence and structure. Mature sequence in capital letters

```
agaggctataagcgggatgatgaatccgaccatgatggtgttgcagaaatttatgtatgatgtagttattgtccagggatcgaaacaccatcatggTCAGATCCGTCATCCCACTTatgttttct   
 (((((.((((((.(((((((((.(((.(((((((((((((((...((..((..((.(..(((....)))..).))..))))..))))))))))))))).))).))))))))).)))))).)))))
```

---

## locus\_id: 287437

family\_id: 108

### **Targets:**

At2g19580(NM\_127516.3
): senescence-associated protein-related  
At2g23810(NM\_179714.2
): senescence-associated family protein

Location in genome: Contained by At4g23410 (NM\_118470: . senescence-associated family protein) in an intron on the reverse strand

1 homologs in brassica

### Alignment between mature miRNA and predicted targets

```
        Extended mature miRNA:       ggtggtttgcagcatccagactgttt
        Target(rc):NM_127516.3    gttGGTGGTTTGCAGCAGCCGGACTGgag
        Target(rc):NM_179714.2       gaaGGTTTGCAGCAACCAGACTGaag
```

### Precursor sequence and structure. Mature sequence in capital letters

```
ggtaacaatcagggtcttgttgtatcaccgtgtccgtgttgtaaacgcacgacgtcggtGGTTTGCAGCATCCAGACTGtttcc   
 ((.((((.((.((((.((((...(((((((((((.((((.......)))))))).)))))))..)))).)))).)).)))).))
```

---

## locus\_id: 271459

family\_id: 107

### **Targets:**

At1g18580(NM\_101716.2
): glycosyltransferase family protein 8  
At5g65070(NM\_125906.1
): MADS-box protein (MAF4)

Location in genome: in an IGR, 318036 upstream of At4g06526, 26956 downstream of At4g06599

### Alignment between mature miRNA and predicted targets

```
        Extended mature miRNA:       cacctctggttctcctctcatagctgg
        Target(rc):NM_125906.1       aaaCTCTGGTTCTCCTCTCTCAGCagc
        Target(rc):NM_101716.2           ataGGTTGTCCTCTCATAGCTGCttt
```

### Precursor sequence and structure. Mature sequence in capital letters

```
gagcctgcataggatgcggcggaggcggaggtggatagtgaggcggaactaccatatccggttcacgctgtggagcattcacCTCTGGTTCTCCTCTCATAGCtggctc   
 (((((.((.((.((.(.((.((((.((((((((((..(((((.((((.........)))).)))))(((....))).)))))))))).)))))).))).)))).)))))
```

---

## locus\_id: 201887

family\_id: 106

### **Targets:**

At3g58870(NM\_115749.2
): expressed protein  
At3g13440(NM\_112189.3
): expressed protein

Location in genome: in an IGR, 7676 upstream of At3g31430, 38822 downstream of At3g31540

### Alignment between mature miRNA and predicted targets

```
        Extended mature miRNA:       ggtggtctctcggttgtggacgctgagaga
        Target(rc):NM_115749.2       gtgGGTTTCTCGGTTGTGGACGCCGAGgag
        Target(rc):NM_112189.3       gggGGTCTCTTGGTTATGGACGCTGAGggt
```

### Precursor sequence and structure. Mature sequence in capital letters

```
gcagcggtGGTCTCTCGGTTGTGGACGCTGAGagacgcggcgagtttctcaggctcatccgttgatctagtcgctgc   
 (((((((.((((...(((..(((..(.(((((((((.......))))))))))..))))))..))))...)))))))
```

---

## locus\_id: 355409

family\_id: 105

### **Targets:**

At5g11490(NM\_121187.2
): adaptin family protein  
At5g47390(NM\_124110.2
): myb family transcription factor

Location in genome: in an IGR, 4852 upstream of At5g36100, 63 downstream of At5g36120

### Alignment between mature miRNA and predicted targets

```
        Extended mature miRNA:       ccgcaggtagttgcggctgcggcaga
        Target(rc):NM_124110.2       agcCAGGTAGTTGCGGCTGCGGTtgc
        Target(rc):NM_121187.2         tggGGTAGTTGCGGATGTGGCAGggc
```

### Precursor sequence and structure. Mature sequence in capital letters

```
cgtcgccgCAGGTAGTTGCGGCTGCGGCagacgcatatgaaattgttcgttttgttgccgccagtacttgcggtaagacg   
 ((((((((((((((.(.(((((.(((..(((((.(((......))).)))))))).))))).).))))))))))..))))
```

---

## locus\_id: 243612

family\_id: 102

### **Targets:**

At5g33340(NM\_122858.1
): aspartyl protease family protein  
At3g04920(NM\_111364.2
): 40S ribosomal protein S24 (RPS24A)

Location in genome: in an IGR, 10132 upstream of At3g30180, 54013 downstream of At3g29970

### Alignment between mature miRNA and predicted targets

```
        Extended mature miRNA:       tctggatcttacttcttctcggcatcgac
        Target(rc):NM_122858.1      ttgTGGATCTTGCTTCTTCTCAGCATCGAtag
        Target(rc):NM_111364.2       tcaGGATCTCACTTCTTCTTGGCATCacc
```

### Precursor sequence and structure. Mature sequence in capital letters

```
agtggagagcttgacgccagaggaggtgatggagctcaaggacggctctGGATCTTACTTCTTCTCGGCATCgacttggct   
 (((.(((...((((.(((((((((((((((((((((.......)))))))....))))))))))).))).))))))).)))
```

---

## locus\_id: 223347

family\_id: 101

### **Targets:**

At4g12810(NM\_117350.1
): F-box family protein  
At1g67160(NM\_105386.1
): F-box family protein

Location in genome: in an IGR, 5672 upstream of At3g03750, 8918 downstream of At3g03710

### Alignment between mature miRNA and predicted targets

```
        Extended mature miRNA:       ttcggaaagaggatgatccatggagttgatg
        Target(rc):NM_117350.1       tttGGAAATAGGATGAGCCATGGAGTTGttg
        Target(rc):NM_105386.1       tttGGAAAAAGAATGATCCATGGAGTTGttg
```

### Precursor sequence and structure. Mature sequence in capital letters

```
gaggatcatacaacttacatgaatcgttgtttctttcaacaaattcgttcGGAAAGAGGATGATCCATGGAGTTGatggtcctc   
 (((((((((.((((((.((((.((((((..(((((((.((......))...))))))))))))).)))))))))))))))))))
```

---

## locus\_id: 371737

family\_id: 98

### **Targets:**

At5g59190(NM\_125309.1
): subtilase family protein  
At5g58840(NM\_125274.1
): subtilase family protein

Location in genome: in an IGR, 2029 upstream of At5g58800, 16494 downstream of At5g58860

### Alignment between mature miRNA and predicted targets

```
        Extended mature miRNA:       ctggctgcaacggttaagatccacgg
        Target(rc):NM_125274.1       cttGCTGCAACTGTCAAGATCCAtgg
        Target(rc):NM_125309.1   accGCTGGCTGCAACGGTTATCATCCAtgg
```

### Precursor sequence and structure. Mature sequence in capital letters

```
tgtggtgctgGCTGCAACGGTTAAGATCCAcggtgctacgctcacggttggattcggaccattgttaccagctgactgca   
 .(..(((((((..((((.((((..(((((((.(((.......))).).))))))..)))).))))..)))))..))..).
```

---

## locus\_id: 39263

family\_id: 91

### **Targets:**

At5g04230(NM\_120505.2
): phenylalanine ammonia-lyase 3 (PAL3)  
At2g37330(NM\_129289.3
): expressed protein

Location in genome: in an IGR, 13769 upstream of At1g61200, 11943 downstream of At1g61255

### Alignment between mature miRNA and predicted targets

```
        Extended mature miRNA:       tgacggatctgaccatgatggtgttt
        Target(rc):NM_129289.3       agtCGGATCTGACCGTGACGGTGgta
        Target(rc):NM_120505.2         ttcGATCTGACCAGGATGGTGCTTgag
```

### Precursor sequence and structure. Mature sequence in capital letters

```
agtgggatgaCGGATCTGACCATGATGGTGtttcgatccctggacaataactacatcatacataaatttctgcaacaccatcatggtcggattcatcatcccgct   
 ((((((((((.(((((((((((((((((((((.(((................................)).).))))))))))))))))))))).))))))))))
```

---

## locus\_id: 72283

family\_id: 86

### **Targets:**

At1g31670(NM\_102902.1
): copper amine oxidase, putative  
At4g12220(NM\_117292.1
): hypothetical protein

Location in genome: in an IGR, 6454 upstream of At1g30825, 16756 downstream of At1g30800

### Alignment between mature miRNA and predicted targets

```
        Extended mature miRNA:       ggtgacgtggtggtgactagaagagc
        Target(rc):NM_117292.1       tcgGATGTGGTGGTGACTAGAACtac
        Target(rc):NM_102902.1     tgcCTGACGTGGTGGTGACTTGAcat
```

### Precursor sequence and structure. Mature sequence in capital letters

```
ggcgaaggtGACGTGGTGGTGACTAGAAGagccatgtctggtgtggcgcctagttagcgtcgtgtcggtacatcgtcagctccacatgtcaactaatcgct   
 (((((((.(((((((.(((.(.((.((.((....(((((((..((((((.......))))))..)))).))))).))))).)))))))))).))..)))))
```

---

## locus\_id: 303857

family\_id: 85

### **Targets:**

At5g34860(NM\_122875.1
): hypothetical protein  
At1g41750(NM\_103401.1
): hypothetical protein

Location in genome: in an IGR, 1990 upstream of At4g06479, 41809 downstream of At4g05640

### Alignment between mature miRNA and predicted targets

```
        Extended mature miRNA:       agtgacacccgtcgccacgccatttc
[truncated: 61,872 more chars]
